# Metabolic Profiling of Serum for Osteoarthritis Biomarkers

**DOI:** 10.1155/2022/1800812

**Published:** 2022-07-28

**Authors:** Ziqian Xiao, Zhenyang Zhang, Shanbin Huang, Jerome Rumdon Lon, Shuilin Xie

**Affiliations:** ^1^School of Biology and Biological Engineering, South China University of Technology, Guangzhou, China; ^2^School of Physical Education, South China University of Technology, Guangzhou, China; ^3^Institute of Synthetic Biology, Shenzhen Institute of Advanced Technology, Shenzhen, China

## Abstract

Osteoarthritis is a prevalent aging disease in the world, and in recent years it has shown a trend toward younger age, which is becoming a major health problem in the world and seriously endangers the health of the elderly. However, the etiology and pathogenesis of osteoarthritis are still unclear, causing great trouble for treatment. To screen out candidate biomarkers that could be used for the identification of osteoarthritis and explore the pathogenesis of osteoarthritis, we performed an untargeted metabolomics analysis of nine New Zealand rabbit serum samples by LC-MS/MS, including three normal serum samples (control group) and six osteoarthritis serum samples (case group). Finally, 44 differential metabolites were identified, and the ROC analysis results indicated that a total of 36 differential metabolites could be used as candidate biomarkers. Further metabolic pathway enrichment analysis was performed on these differential metabolites, and we found that a total of 17 metabolic pathways were affected, which may provide directions for the study of osteoarthritis mechanisms.

## 1. Introduction

Osteoarthritis (OA) is a prevalent degenerative disease, and the incidence rate increases with age. In the current research, the specific pathogenesis of OA has not yet been investigated. Patients with OA have a slow onset in the early stages, with no significant systemic symptoms. It takes up to two years from the onset of pain to the choice to go to the hospital, and more than 90% of these patients only go to the hospital after they develop knee pain. There are two main types of conventional methods to examine OA. One is imaging, which includes X-ray film examination, irradiation CT, and MRI; the other is laboratory tests, including hematocrit blood tests, thermal agglutination tests, and the examination of the joint fluid in the joint cavity [1]. Imaging is relatively easy, but the probability of misdiagnosis is very high and is often confused with ankylosing spondylitis resulting in medical misdiagnosis. Laboratory inspections are more numerous, complex, and challenging to detect.

Considering that a large number of biological processes are involved in arthritis, further characterization of the disease mechanisms is needed, which can be used for earlier diagnosis, intervention, or treatment. Peng et al. designed a new fluorescence turn-on ADAMTS-4-D-Au probe for detecting ADAMTS-4 activity, which could be used for the early diagnosis of cartilage-damage diseases [2]. Leung et al. developed a deep learning prediction model on knee radiographs, which can accurately predict OA progression in some patients [3]. For the diagnosis of OA, many traditional methods have many defects, and new detection methods are rarely reported. Therefore, we focus on metabolomics. Metabolomics is an emerging field that looks for specific metabolic pathways through the study of biomarkers to determine the causes of various diseases from the perspective of metabolite analysis, where lifestyle, diet, disease, and genetics can affect multiple metabolite concentrations simultaneously [4]. Some studies in recent years have attempted to identify biomarkers of arthritis through metabolomics and have identified many potential targets in urine, synovial fluid, and serum. However, specific biomarkers have not yet been identified, so the search for biomarkers through metabolomics is gradually evolving into a breakthrough direction [5, 6]. For example, before this study, Maerz et al. performed a serum metabolomics analysis after anterior cruciate ligament injury in rats. They conducted a preliminary study on inflammation and immune disorders in traumatic OA [7]. Some metabolomics analyses for OA showed that OA affects the metabolism of amino acids [8–10]. Many biomarkers that could be used for the early diagnosis of OA have been reported in many studies [10–12]. These corroborate the merits of metabolomics studies for understanding the disease mechanism of arthritis.

In recent years, the modified Hulth method and intraarticular injection method are common methods to construct OA models. In this study, New Zealand white rabbits were used as the experimental model, three as the control group, and six were constructed as the experimental group through the modified Hulth method and intra-articular injection method. The modified Hulth method is a classic OA modeling method, which leads to OA by destructing the bone and joint tissue. The intraarticular injection method can degrade chondroitin sulfate of the cartilage matrix, which increases the amount of free water inside the cartilage and affects the cartilage elasticity, compressive resistance, and integrity of cartilage so that stresses in the normal range can also cause cartilage damage, and eventually leading to the occurrence of typical OA [13]. Therefore, we collected rabbit blood samples from the constructed OA models to prepare serum for metabolomics analysis.

In this project, liquid chromatography-tandem mass spectrometry (LC-MS/MS) was used to identify the metabolites and compare the levels of metabolites between the two groups. The differential metabolites were screened by multivariate statistical analysis and univariate analysis, and they were exhibited by clustered heat map and volcano plot. The diagnostic ability of the screened differential metabolites was judged by ROC analysis to select candidate biomarkers for OA. The differential metabolites were finally subjected to pathway enrichment analysis to determine the metabolic pathways affected by OA.

## 2. Materials and Methods

### 2.1. Sample Information

Nine New Zealand white rabbit serum samples used for metabolomics analysis were purchased from Guangzhou Huateng Biomedical Technology Co., Ltd. The samples were divided into control group and case group. The control group includes three rabbit serum samples without operation named control-1, control-2, and control-3, and the case group includes six OA rabbit serum samples named case-1, case-2, case-3, case-4, case-5, and case-6. All the samples were stored in a refrigerator at − 80 °C.

The model construction method could be referred to the following article [14]. Nine male six-month-old New Zealand white rabbits were used for animal experiments. All the rabbits were raised in separate cages and were able to eat and drink freely. The ambient temperature was maintained at 20 °C~25 °C, and the humidity was maintained at 40%~60%. The simulated natural light was 12 hours a day. The animal experiment and research process followed the 3R principle and has been approved by the experimental animal ethics committee of Guangzhou Huateng Biological Medicine Technology Co., Ltd. (htsw201003).

The model construction methods are as follows: the modified Hulth method and the type II collagenase method were used to construct 3 rabbit knee OA models, respectively. The modified Hulth method is to cut off the medial collateral ligament and anterior cruciate ligament, remove the medial meniscus, and construct the knee OA model through the complete rupture of the anterior cruciate ligament. The type II collagenase method is to inject 0.5 ml (4 mg/ml) of type II collagenase solution into the articular cavity through the medial depression of the patellar ligament. All operations require strict aseptic operation environment.

One week after surgery, the animals in each group were driven to exercise for 30 minutes every day, and the International OA Lequesne MG Index [15] was used as the standard for model evaluation, which lasted for 6 weeks. The scoring was performed twice a week, and a total score greater than or equal to 3 was considered successful modeling. Blood was collected from the ear margin vein 6 weeks after the operation. The blood was collected in procoagulation tubes, and the serum was collected by centrifuging at 650 rcf for 10 min after standing at 4 °C for 30 min.

### 2.2. Extraction of Metabolites

Serum was stored in − 80 °C refrigerator before sample preparation. 100 *μ*L samples were extracted by directly adding 300 *μ*L of precooled methanol and acetonitrile (2:1, v/v, Thermo Fisher Scientific, USA). After vortexing (QL-901, Kylin-bell Lab Instruments Co., Ltd., China) for 1 min and incubating at − 20 °C for 2 hours, the samples were centrifuged at 14800 rcf for 10 min at 4 °C, and the supernatants were then transferred for vacuum freeze drying (Maxi Vacbeta, GENE COMPANY). The metabolites were resuspended in 150 *μ*L of 50% methanol and centrifuged at 14800 rcf for 10 min at 4 °C; then, the supernatants were transferred to autosampler vials for LC-MS analysis.

### 2.3. LC-MS/MS Analysis Conditions

Metabolites separation was performed on a Waters 2D UPLC (Waters, USA) with a Waters ACQUITY UPLC BEH C18 column (1.7 *μ*m, 2.1 mm × 100 mm, Waters, USA), and the column temperature was maintained at 45 °C. The mobile phase in positive ion mode is as follows: 0.1% formic acid solution (50144-50 ml, DIMKA, USA) (A) to 0.1% formic acid methanol solution (B). The mobile phase in negative ion mode is as follows: 10 mM ammonium formate solution (17843-250G, Honeywell Fluka, USA )(A) to 10 mM ammonium formate in 95% methanol (B). The gradient conditions were as follows: 0-1 min, 2% B; 1-9 min, 2%-98% B; 9-12 min, 98% B; 12-12.1 min, 98% B to 2% B; and 12.1-15 min, 2% B. The flow rate was set at 0.35 mL/min, and the injection volume was 5 *μ*L.

Primary and secondary mass spectrometry data were collected by Q Exactive (Thermo Fisher Scientific, USA). The mass spectrometric settings for positive and negative ionization modes were as follows: The full scan range was 70–1050 m/z; spray voltages, 3.80 kV/3.20 kV; capillary temp, 320 °C; Aux gas heater temp, 350 °C; sheath gas flow rate, 40 arbitrary units; Aux gas flow rate, 10 arbitrary units; and runtime, 13 min.

### 2.4. Data Processing

Raw data from mass spectrometry was imported into Compound Discoverer 3.1 (Thermo Fisher Scientific, USA) for data processing, including peak extraction, peak alignment, and metabolite identification , and information about compound molecular weight, retention time, peak area and identification results were exported. Metabolites were identified by using the BGI self-built standard library database, HMDB database, KEGG database, mzCloud database, and Chemspider database. The basis for identification includes precursor mass tolerance <5 ppm, fragment mass tolerance <10 ppm, and RT tolerance <0.2 min. The results exported from the Compound Discoverer 3.1 were imported into metaX [16] for data preprocessing, statistical analysis, and metabolite analysis. Data was normalized using the probabilistic quotient normalization method (PQN [17]) to obtain the relative peak area. Multivariate statistical analysis and univariate analysis were performed on metaX [16]. The distribution and separation trends of the two groups were observed by multivariate statistical analysis, and then the fold change (FC) with *p* value was obtained by univariate analysis.

### 2.5. Screening and Analysis of the Differential Metabolites

Fold change analysis of metabolites was performed between the two groups, and *t*-test was further performed to determine whether the differences in metabolites between groups were significant. Screening for differential metabolites was based on the results of univariate analysis, the differential metabolite screening conditions were as follows: fold change ≥ 1.2 or ≤ 0.83 and *P* value < 0.05, and metabolites meeting these conditions could be considered significantly different. For clustering analysis of differential metabolites, data was log 2 transformed and *Z*-score normalized; hierarchical clustering was used for clustering algorithms, and Euclidean distance was used for distance calculation. Through the cluster analysis, the variation trends of differential metabolites can be seen, and the metabolites with the same variation trends can be identified. After that, the screened metabolites were visual displayed by volcano plots. The filtered differential metabolites were imported into databases for identification, the identified differential metabolites were subjected to ROC analysis, and the differential metabolites with AUC > 0.9 could be tentatively used as biomarkers for OA. The identified differential metabolites were finally imported into the KEGG database for metabolic pathway enrichment analysis.

## 3. Results

### 3.1. Serum Metabolites Analysis

**Figure 1:**
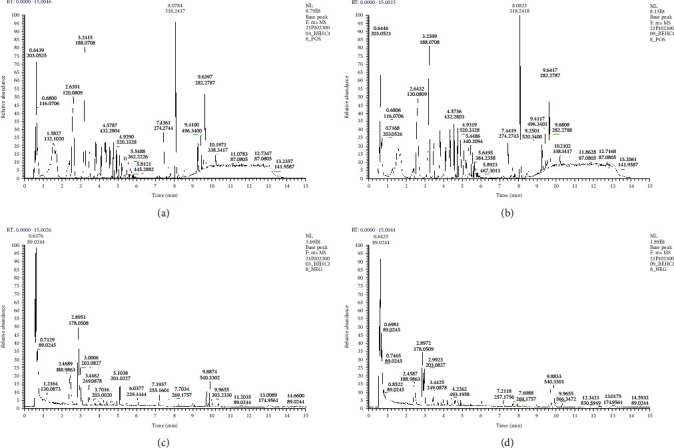
Base peak ion chromatogram. (a) (ESI+) BPC-control. (b) (ESI+) BPC-case. (c) (ESI-) BPC-control. (d) (ESI-) BPC-case.

Herein, we selected a sample from each group for base peak ion chromatogram (BPC) inspection. The BPC chart is shown in Figure 1. It shows that the samples have good peak shape and large peak capacity.

**Figure 2:**
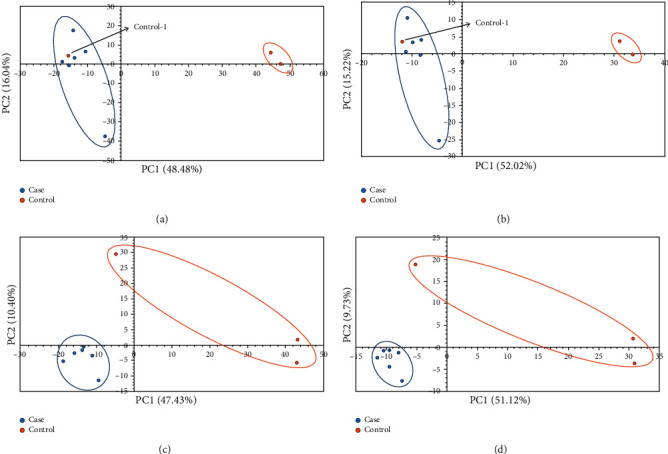
PCA and PLS-DA score plots. (a) (ESI+) PCA. (b) (ESI-) PCA. (c) (ESI+) PLS-DA. (d) (ESI-) PLS-DA. The abscissa is the first principal component PC1, and the ordinate is the second principal component PC2. The number is the score of the principal component, which represents the percentage of the explanation on overall variance of the specific principal component.

We performed the multivariate statistical analysis and univariate analysis on the processed data by metaX [16]. The PCA and PLS-DA [18, 19] score plots are shown in Figure 2. The PCA analysis shows that the scores of the two principal components in the positive ion mode are PC1 = 48.48% and PC2 = 16.04% and the scores in the negative ion mode are PC1 = 52.02% and PC2 = 15.22%. It can be seen that there is an abnormal point in the control group and this point comes from the sample control-1, indicating that the sample has individual differences. Further PLS-DA [18, 19] analysis was performed on the two groups. Through the supervised statistical analysis, the differences between the groups were expanded, and the differences within the groups were reduced. From the score plots, it can be seen that the control group is mainly distributed on the right side, while the OA group is mainly clustered on the left. The two have a good separation, which indicates that our data can be used for further analysis.

### 3.2. Screening of Candidate Biomarkers

**Figure 3:**
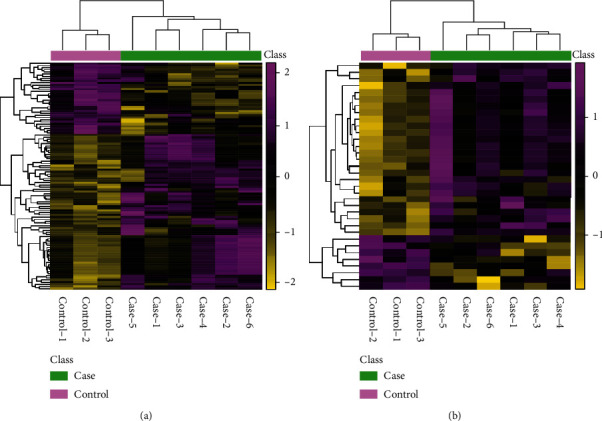
Clustering heat map. (a) (ESI+) Clustering heat map. (b) (ESI-) Clustering heat map. Each row represents a differential metabolite, each column represents a sample, color is the amount expressed, and yellow to purple indicates the amount expressed from low to high.

**Figure 4:**
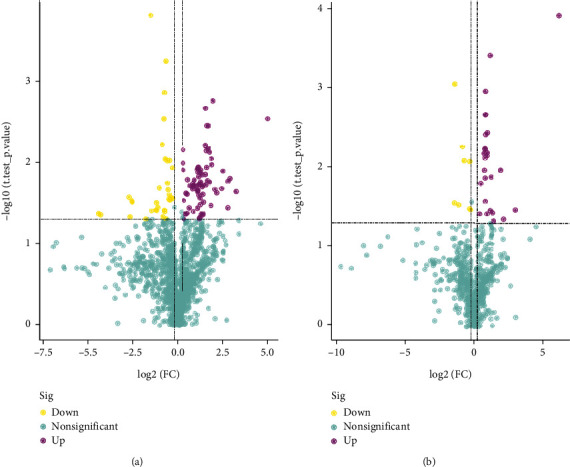
Volcano plots. (a) (ESI+) Volcano plot. (b) (ESI-) Volcano plot. Horizontal axis is log_2_ (FC) and vertical axis is -log_10_ (*P* value). The horizontal and vertical dashed lines were selected as the filtering conditions: *P* < 0.05, and log_2_ (FC) > 1.2 or < 0.83. The differential metabolites in the top left and the top right part were obtained after filtering, yellow representing downregulation and purple representing upregulation.

After data preprocessing, 1632 and 636 features were detected in positive and negative ion mode, respectively, and detailed information is shown in Table S1 and Table S2. In order to screen out differential metabolites, fold change analysis and *t*-test were performed. The screening conditions of differential metabolites were set as fold change ≥ 1.2 or ≤ 0.83 and *p* value < 0.05. The statistics of screening results are shown in Table 1. The cluster heat maps (Figure 3) show that all differential metabolites are divided into two clusters of co-regulated metabolites. In the positive ion mode, 33 metabolites in the upper cluster are significantly downregulated, and 72 metabolites in the lower cluster are upregulated. In contrast, in the negative ion mode, 26 metabolites in the upper cluster are upregulated, and 8 metabolites in the lower cluster are downregulated. The specific calculation results of cluster analysis are shown in Table S3 and Table S4. The upregulated metabolites include fructoselysine, otonecine, and carmustine, and the downregulated metabolites include ɛ, ɛ, ɛ trimethyllysine, tranexamic acid, and triethylamine. Draw the differential metabolites into volcano plots (Figure 4) for visual display, in which purple is the upregulated differential metabolite, yellow is the downregulated differential metabolite, and cyan is the metabolite with no obvious difference.

We further identified the screened differential metabolites through the HMDB database, KEGG database, etc. A total of 35 differential metabolites can be identified in positive ion mode, and 9 differential metabolites can be identified in negative ion mode. ROC analysis was performed on these 44 differential metabolites to evaluate their diagnostic ability. The metabolites with the area under the curve (AUC) > 0.9 can be used as candidate biomarkers. ROC analysis results are shown in Table 2, and data about the relative peak area of each metabolite used for ROC analysis are shown in Table S5. Finally, we determined 36 metabolites as candidate biomarkers of OA. The relevant information is shown in Table 2. These candidate biomarkers include ɛ, ɛ, ɛ trimethyllysine, ascorbic acid, otonecine, and tranexamic acid. All these metabolites have good diagnostic ability for OA.

### 3.3. Pathway Analysis of Differential Metabolites

**Figure 5:**
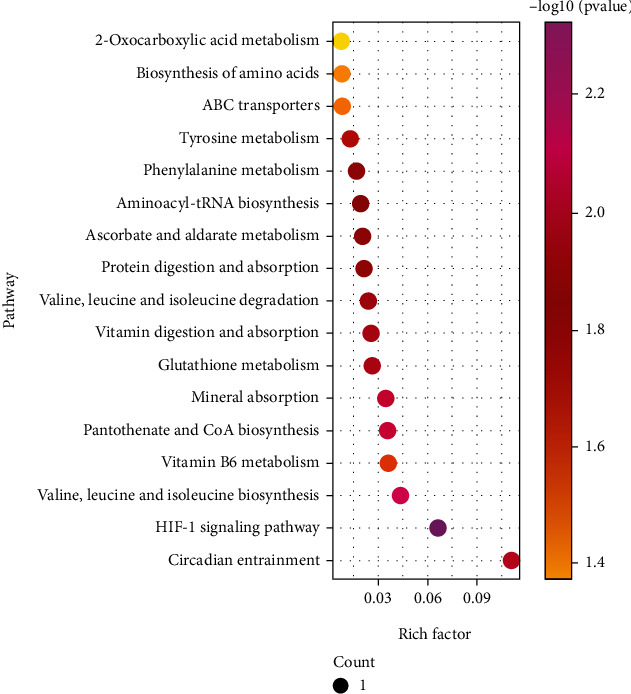
Bubble plots for metabolic pathway enrichment analysis. Horizontal axis is the enrichment factor and vertical axis is the pathway name. Dot size represents the number of differential metabolites annotated to that pathway.

The filtered differential metabolites were imported into the KEGG database for pathway enrichment analysis, from which we found that 17 metabolic pathways were affected. Circadian rhythm and vitamin B6 metabolism were detected in positive ion mode, and the metabolic pathways seen in negative ion mode included the HIF-1 signaling pathway, pantothenate and CoA biosynthesis, mineral absorption, and glutathione metabolism. The total enrichment results are displayed in the bubble plot for metabolic pathway enrichment analysis (Figure 5) and the metabolic pathway enrichment results' table (Table 3), from which it can be seen that the metabolic pathway with the most significant enrichment factor is circadian rhythm. Circadian rhythm is a regular cycle established by adapting various physiological functions to changes in the external environment, reflecting that circadian rhythm has a particular impact on the development of OA. Kc et al. [20] have established circadian rhythm disordered mouse models and found that the disorder of the biological clock system leads to pathological changes in the knee joint, suggesting that circadian rhythm disorder will induce OA development. The report has also shown that the autonomous clock in chondrocytes regulates key pathways involved in OA [21], which can provide more new ideas for treating OA by investigating the molecular mechanisms between circadian rhythm and OA development. At the same time, it can be seen that the metabolism of multiple amino acids is affected, including valine, leucine, isoleucine biosynthesis and degradation, phenylalanine metabolism, and tyrosine metabolism. Meanwhile, upregulated metabolite L-(+)-valine affects several metabolic pathways, including valine, leucine, isoleucine biosynthesis, pantothenate and CoA biosynthesis, mineral absorption, and protein digestion and absorption. Many studies have reported the relationship between amino acid metabolism and OA [22, 23], and it can be seen that OA has an essential impact on the metabolism of relevant amino acids.

## 4. Discussion

Osteoarthritis is a gradually progressive chronic disease. The degenerative damage and reactive hyperplasia of articular cartilage caused by OA are related to many factors, such as increasing age, obesity, and strain. OA is often diagnosed by clinical diagnosis and imaging methods, and standard imaging methods include radiographs, magnetic resonance imaging (MRI), ultrasound (US), and optical coherence tomography (OCT) [24]. However, the diagnosis of OA is often not confirmed until late in the disease, while approximately half of those identified by imaging methods do not have associated symptoms or disability, and the clinical relevance of some radiological features is not completely clear [25, 26]. Biomarkers play an essential role in the diagnosis and stage judgment of the disease, and it is vital to study the OA-related biomarkers in the early diagnosis of OA. Based on this, we hope to screen out the OA-related biomarkers and provide a basis for the early diagnosis of OA.

This project performed an untargeted metabolomics analysis of nine New Zealand rabbit serum samples based on LC-MS/MS to extract and compare serum metabolites between control and OA groups. We identified 44 differential metabolites, which were further subjected to ROC analysis. A total of 36 differential metabolites were screened to serve as candidate biomarkers to provide some ideas for the early diagnosis of OA. Through the pathway enrichment analysis of differential metabolites, we found that 17 metabolic pathways were affected, which were worth further study.

The association between OA and circadian rhythms seems to be expected. In fact, several studies have shed light on the potential relationship between rheumatoid arthritis and circadian rhythms [27], primarily due to the body's hormone levels varying widely at different times of the day. Circadian rhythms originate from the central pacemaker in the brain's suprachiasmatic nucleus (SCN) and are regulated by light-sensitive retinal nerves [28]. This rhythm plays an essential role in regulating endocrine and immune functions. Some previous studies have shown that melatonin secretion significantly increases in patients with rheumatoid arthritis during the night, while endogenous cortisol synthesis is subsequently activated to counteract the cascade of symptoms [29]. This rhythmic fluctuation may play a role in the pathophysiology of rheumatoid arthritis. Previous studies have found that direct inhibition of pro-inflammatory factors such as melatonin contributes to clinical improvement in rheumatoid arthritis [30]. Some studies revealed that RA risk increases with an unhealthy daily routine [31, 32]. In addition, OA and rheumatoid arthritis are chronic inflammatory diseases. The immune system's excessive response to OA at night uses up a lot of energy [33, 34]. This disrupts the balance of energy expenditure maintained by the circadian rhythm, which can lead to discomfort beyond the symptoms of arthritis itself [35]. In conclusion, the relationship between chronic inflammation and dysfunctional circadian rhythms in rheumatoid arthritis and OA appears to be complex, with many potential interactions.

Compared with other studies, we also identified that OA upregulated HIF-1 signaling pathway [36, 37]. Hypoxia-inducible factor 1 (HIF-1) can detect environmental oxygen content and adapt to hypoxic environment by activating genes related to oxygen homeostasis [38]. Hypoxia has been proved to be one of the main factors inducing OA, and HIF-1 *α* subunit has good stability under hypoxic conditions and can protect chondrocytes [36, 39]. Hu et al. found that HIF-1*α* can alleviate chondrocyte apoptosis and senescence through mitophagy under hypoxic conditions to improve the symptoms of OA [37]. In addition, HIF-1*α* can promote the formation of chondrocytes, maintain chondrocyte viability, and support chondrocytes to adapt to hypoxic environment by mediating a series of reactions to protect articular cartilage [40]. In short, HIF-1 is closely related to OA.

The metabolism of ascorbic acid was also affected. The senescence of chondrocytes is an important factor in the production of OA, and the oxygen free radicals can degrade collagen and proteoglycans, which are considered to be the main inducers of chondrocyte senescence [41, 42]. Ascorbic acid has strong reducibility, so the increase of ascorbic acid in the serum of the case group is likely to be a self-protection mechanism. Studies have also shown that ascorbic acid plays an important role in collagen synthesis and can stimulate the expression of collagen and aggrecan in articular cartilage [43, 44]. Liao et al. found that it could effectively delay cartilage degeneration by injecting ascorbic acid-ferric chloride mixture into the articular cavity of OA rats [45]. These all show the close link between ascorbic acid and OA and provide ideas for the treatment of OA.

Our results showed that OA significantly impacts amino acid metabolism, especially on branched-chain amino acid (BCAA) metabolism, which has been confirmed by previous reports [8, 10]. BCAA, including valine, leucine, and isoleucine, play an important role in promoting muscle growth and releasing hormones. Zhai et al. first studied the serum-based metabolomics of human OA and found that the ratio of BCAA to histidine has potential clinical use as a biomarker of OA [8]. Although our study did not show changes in histidine content, these studies can reflect the close relationship between BCAA and OA. BCAA is related to the increasing number of major pro-inflammatory cytokines involved in the pathophysiology of OA, which can lead to the degradation of the articular cartilage matrix [46]. It has also been reported that BCAA reflects the relationship between obesity and OA and obesity is an essential factor in the occurrence of OA [47]. In addition, this project showed that valine could be used as a potential biomarker for OA, which had also been reported in a previous study [48]. This project also reported some new biomarkers, which have a specific significance for the development of new diagnostic methods for OA. In conclusion, the relationship between amino acid metabolism and OA has always been a hot topic.

There are some limitations to this study. OA is easily confused with other types of arthritis, such as rheumatoid arthritis. We did not investigate the potential relationship between OA and other arthritic diseases; further validation of whether the screened biomarkers are also able to identify other types of arthritis is needed. Second, the sample size in this study is small, so we could not make a further interpretation of the screened biomarkers. Meanwhile, we obtained the differential metabolites by database matching, and no further validation was done by comparing with standards, which needs to be done in the future. This study used New Zealand rabbits as the research object, and the genetic information of this species is different from that of humans. Future research needs to be closer to the clinical aspect.

## 5. Conclusion

In summary, untargeted metabolomics analysis was performed on nine serum samples by LC-MS/MS. This study finally identified 44 differential metabolites, and 36 of the 44 metabolites were served as candidate biomarkers for OA by ROC analysis. A total of 17 metabolic pathways were affected due to OA, including circadian rhythm, amino acid metabolism, and HIF-1 signaling pathway. These reveal the potential mechanism of OA and provide new research directions for future therapeutics and development of new drugs in the field of OA.

## Figures and Tables

**Table 1:** Differential metabolite statistics.

Mode	Group	Total number of differential metabolites	Up	Down
pos	case_control	105	72	33
neg	case_control	34	26	8

**Table 2:** List of candidate biomarkers.

Name	Fold-change	*P* value	Direction	AUC
ESI+				
ɛ, ɛ, ɛ trimethyllysine	0.3427	0.0002	Down	1
Otonecine	2.2536	0.0188	Up	0.944
Tranexamic acid	0.0472	0.0431	Down	1
Triethylamine	0.668	0.0219	Down	1
Carmustine	2.8701	0.0022	Up	1
Epiguanine	0.5722	0.003	Down	0.944
Bayer e 39 soluble	6.8063	0.0173	Up	1
Etilevodopa	9.1913	0.023	Up	1
Leu-val	0.6167	0.0006	Down	1
Melatonin	7.3363	0.016	Up	0.944
1,4-naphthoquinone	2.597	0.0428	Up	0.944
Arg-asp	3.3794	0.0177	Up	0.944
Istamycin a1	1.4538	0.0165	Up	1
Deferoxamine	2.3565	0.0185	Up	0.944
Codonocarpine	1.5971	0.0196	Up	0.944
Sophoranone	2.3365	0.0478	Up	1
Anecortave	3.1962	0.0036	Up	1
Compactin	2.1724	0.0118	Up	0.944
Quinoline	0.4259	0.0316	Down	1
12‑deoxyphorbol 20-acetate 13-(2-methylbutanoate)	2.8926	0.0189	Up	1
Khellin	0.6024	0.0093	Down	0.944
Epelsiban	3.0074	0.0036	Up	1
Nevirapine	0.1551	0.047	Down	0.944
(1r,5r)-3,3,5-trimethylcyclohexyl 5-oxo-l-prolinate	6.7929	0.0362	Up	0.944
Theaspirane	0.3567	0.0397	Down	0.944
2475675	0.1698	0.0309	Down	1
Drostanolone propionate	0.1679	0.0303	Down	1
ESI-				
Ascorbic acid	2.2196	0.0004	Up	1
L-(+)-valine	2.2286	0.0345	Up	1
2‑hydroxy-1,2-diphenylethyl hydrogen sulfate	1.3409	0.038	Up	1
4-carboxy-2-(tyrosylamino)butanoate	7.7855	0.0339	Up	0.944
Gentisic acid	0.3758	0.0009	Down	1
Benzoic acid	1.9018	0.038	Up	0.944
N-(5-amino-2-[(2,6-diamino-2,6-dideoxyhexopyranosyl)oxy] -3-{[3-o-(2,6-diamino-2,6-dideoxyhexopyranosyl)-beta-d- ribofuranosyl]oxy}-4-hydroxycyclohexyl)acetamide	1.7462	0.0136	Up	1
D-tryptophyl-d-alanyl-d-allothreonylglycyl-d-histidyl- l-phenylalanyl-d-methioninamide	2.6554	0.0467	Up	0.944
Probucol	2.3401	0.0132	Up	1

**Table 3:** Affected metabolic pathways in rabbit serum.

Pathway	RichFacor	KEGG.Name	KEGG.ID	*P* value	Direction
ESI-					
HIF-1 signaling pathway	1/15	Ascorbic acid	C00072	0.0049	Up
Valine, leucine, and isoleucine biosynthesis	1/23	L-(+)-valine	C00183	0.0074	Up
Pantothenate and CoA biosynthesis	1/28	L-(+)-valine	C00183	0.0091	Up
Mineral absorption	1/29	L-(+)-valine	C00183	0.0094	Up
Glutathione metabolism	1/38	Ascorbic acid	C00072	0.0123	Up
Vitamin digestion and absorption	1/39	Ascorbic acid	C00072	0.0126	Up
Valine, leucine, and isoleucine degradation	1/42	L-(+)-valine	C00183	0.0136	Up
Protein digestion and absorption	1/47	L-(+)-valine	C00183	0.0152	Up
Ascorbate and aldarate metabolism	1/49	Ascorbic acid	C00072	0.0158	Up
Aminoacyl-tRNA biosynthesis	1/52	L-(+)-valine	C00183	0.0168	Up
Phenylalanine metabolism	1/60	Benzoic acid	C00180	0.0193	Up
Tyrosine metabolism	1/78	Gentisic acid	C00628	0.0251	Down
ABC transporters	1/124	L-(+)-valine	C00183	0.0396	Up
Biosynthesis of amino acids	1/128	L-(+)-valine	C00183	0.0408	Up
2-Oxocarboxylic acid metabolism	1/134	L-(+)-valine	C00183	0.0427	Up
ESI+					
Circadian entrainment	1/9	Melatonin	C01598	0.0111	Up
Vitamin B6 metabolism	1/28	4-pyridoxate	C00847	0.0343	Down

## Data Availability

The data used to support the findings of this study are available from the corresponding author upon request.

## Supplementary Materials

Supplementary 1 Table S1. Metabolites detected in positive ion mode.

Supplementary 2 Table S2. Metabolites detected in negative ion mode.

Supplementary 3 Table S3. Cluster analysis results in positive ion mode.

Supplementary 4 Table S4. Cluster analysis results in negative ion mode.

Supplementary 5 Table S5. Data used for ROC analysis.
