# Supplementary material for: Metabolic Profiling of Serum for Osteoarthritis Biomarkers
Source: Dis Markers. 2022 Jul 28;2022:1800812. doi: 10.1155/2022/1800812 (PMC9356247; doi:10.1155/2022/1800812)
Supplement: Supplementary 1 — Table S1. Metabolites detected in positive ion mode. [file 1800812.f1.pdf]

Table S1 Metabolites detected in positive ion mode

Compound.ID	Fold-change	t.test_p.value	Name	Formula	Molecular Weight	Retention time	KEGG.ID	mzCloud.ID..ChemSpider.ID	HMDB.ID	DeltaMass..Da.	DeltaMass..ppm.	mzVault.Best.Match	mzCloud.Best.Match	ChemSpider.Results	level	Super.class	Class	Sub.class	Family	Pathway	label
0.596_597.9938	0.779	0.0569		C10 H25 N4 O15 P3 S2	597.9938	0.596								0	level5						other
0.598_717.8311	0.5349	0.0954		C19 H5 N4 O17 P3 S2	717.8311	0.598								0	level5						other
0.598_853.8058	0.4974	0.0696			853.8058	0.598								0	level5						other
0.598_921.7956	0.493	0.0947			921.7956	0.598								0	level5						other
0.599_785.8181	0.5369	0.0845		C21 H4 N6 O18 P2 S3	785.8181	0.599								0	level5						other
0.6_581.8556	0.5512	0.0583		C9 H5 N4 O18 P3 S	581.8556	0.6								0	level5						other
0.6_649.8432	0.5427	0.0546		C16 H5 N4 O15 P3 S2	649.8432	0.6								0	level5						other
0.6_845.8201	0.5535	0.0908			845.8201	0.6								0	level5						other
0.601_445.8803	0.581	0.0535		C5 H9 N2 O12 P3 S2	445.8803	0.601								0	level5						other
0.601_513.8679	0.5567	0.0568		C11 H5 N2 O14 P3 S	513.8679	0.601								0	level5						other
0.602_377.8928	0.5832	0.0389		C6 H5 O13 P3	377.8928	0.602								0	level5						down
0.603_335.008	0.7174	0.0542		C11 H16 N O3 P3 S	335.008	0.603								0	level5						other
0.604_309.9054	0.6123	0.0878		C3 H5 O11 P3	309.9054	0.604								0	level5						other
0.606_241.918	0.6118	0.0688		C4 H2 O8 S2	241.918	0.606								0	level5						other
0.606_257.8919	0.4777	0.0209		C3 H2 Cl N2 O4 P3	257.8919	0.606								0	level5						down
0.607_173.9308	0.6179	0.0496		C3 H N2 O P3	173.9308	0.607								1	level5						down
0.609_188.1527	0.3427	0.0002	Nepsilon,nepsilon,nepsilon-trimethyllysine	C9 H20 N2 O2	188.1527	0.609	C03793	BGI429	HMDB0001325	0.0002	1.0349	98.2	97.1	0	level1	Organic acids and derivatives	Carboxylic acids and derivatives	Amino acids, peptides, and analogues	Amino acids, peptides, and analogues	map00310 Lysine degradation; map01100 Metabolic pathways;	down
0.61_105.9433	0.5894	0.0407			105.9433	0.61								0	level5						down
0.629_231.8894	0.6777	0.1093		C3 H Cl O6 S2	231.8894	0.629								0	level5						other
0.659_89.0477	0.6154	0.0559	B-alanine	C3 H7 N O2	89.0477	0.659	C00099	MReference-599	HMDB0000056	0	0.4641	43.4	85.6	0	level2	Organic acids and derivatives	Carboxylic acids and derivatives	Amino acids, peptides, and analogues	Amino acids, peptides, and analogues	map00240 Pyrimidine metabolism; map00410 beta-Alanine metabolism; map00640 Propanoate metabolism; map00770 Pantothenate and CoA biosynthesis; map01100 Metabolic pathways; map04080 Neuroactive ligand-receptor interaction; map04974 Protein digestion and absorption;	other
0.664_174.0503	1.5453	0.0363		C11 H10 S	174.0503	0.664								0	level5						up
0.702_175.121	0.0507	0.0444		C8 H17 N O3	175.121	0.702								0	level5						down
0.708_157.1104	0.0472	0.0431	Tranexamic acid	C8 H15 N O2	157.1104	0.708	C12535	S10482000	HMDB0014447	0.0001	0.8531			2	level4	Organic acids and derivatives	Carboxylic acids and derivatives	Amino acids, peptides, and analogues	Amino acids, peptides, and analogues	NULL	down
0.712_101.1206	0.668	0.0219	Triethylamine	C6 H15 N	101.1206	0.712	C14691	S8158	HMDB0032539	0.0001	1.1836			0	level4	Organic nitrogen compounds	Organonitrogen compounds	Amines	Amines	NULL	down
0.721_213.007	2.8701	0.0022	Carmustine	C5 H9 Cl2 N3 O2	213.007	0.721	C06873	S2480	HMDB0014407	-0.0002	-0.7559			0	level4	Organic acids and derivatives	Organic carbonic acids and derivatives	Ureas	Organic acids	NULL	up
0.726_191.0253	1.8048	0.0201		C6 H9 N O4 S	191.0253	0.726								3	level5						up
0.774_125.0148	0.3015	0.0649	Taurine	C2 H7 N O3 S	125.0148	0.774	C00245	S1091	HMDB0000251	0.0001	0.7688	30.6		6	level4	Organic acids and derivatives	Organic sulfonic acids and derivatives	Organosulfonic acids and derivatives	Organic acids	map00120 Primary bile acid biosynthesis; map00430 Taurine and hypotaurine metabolism; map00920 Sulfur metabolism; map01100 Metabolic pathways; map02010 ABC transporters; map04080 Neuroactive ligand-receptor interaction;	other
1.008_136.0386	0.2705	0.0585	Hypoxanthine	C5 H4 N4 O	136.0386	1.008	C00262	MReference-441	HMDB0000157	0.0001	0.7569		95.5	0	level1	Organoheterocyclic compounds	Imidazopyrimidines	Purines and purine derivatives	Purines and derivatives	map00230 Purine metabolism; map01100 Metabolic pathways;	other
1.235_83.0372	0.3637	0.1646		C4 H5 N O	83.0372	1.235								13	level5						other
1.236_228.9674	0.2734	0.1172		C7 H3 N O6 S	228.9674	1.236								4	level5						other
2.215_338.1478	6.8063	0.0173	Bayer e 39 soluble	C16 H22 N2 O6	338.1478	2.215	C19333	S55158		0	-0.0443			8	level4					NULL	up
3.057_225.1002	9.1913	0.023	Etilevodopa	C11 H15 N O4	225.1002	3.057		S148944		0.0001	0.6202			2	level4						up
3.162_230.163	0.6167	0.0006	Leu-val	C11 H22 N2 O3	230.163	3.162		S312585	HMDB0028942	-0.0001	-0.303			0	level4	Organic acids and derivatives	Carboxylic acids and derivatives	Amino acids, peptides, and analogues	Amino acids, peptides, and analogues		down
3.194_159.0717	2.9563	0.0073		C7 H13 N O S	159.0717	3.194								0	level5						up
3.279_214.0742	0.3661	0.0505		C12 H10 N2 O2	214.0742	3.279								0	level5						other
3.286_244.1358	3.6067	0.0091		C10 H20 N4 O S	244.1358	3.286								0	level5						up
3.819_327.1526	0.6464	0.0097		C12 H25 N O9	327.1526	3.819								0	level5						down
3.827_279.1681	1.3312	0.0752		C12 H25 N O6	279.1681	3.827								0	level5						other

4.05_368.2524	2.8452	0.0063		C16 H36 N2 O7	368.2524	4.05								0	level5						up
4.112_371.179	0.703	0.0097		C18 H31 N O3 P2	371.179	4.112								0	level5						down
4.119_379.1053	1.2343	0.065	S-lactoylglutathione	C13 H21 N3 O8 S	379.1053	4.119	C03451	S389032	HMDB0001066	0.0003	0.8897			0	level4	Organic acids and derivatives	Carboxylic acids and derivatives	Amino acids, peptides, and analogues	Amino acids, peptides, and analogues	map00620 Pyruvate metabolism; map01100 Metabolic pathways;	other
4.272_284.1584	2.1569	0.0911		C10 H24 N2 O7	284.1584	4.272								0	level5						other
4.455_242.1267	0.4487	0.036		C11 H18 N2 O4	242.1267	4.455								0	level5						down
4.506_417.2575	1.4538	0.0165	Istamycin a1	C18 H35 N5 O6	417.2575	4.506	C17987	S30791529		-0.0012	-2.825			0	level4					NULL	up
4.54_555.2563	0.6646	0.0517		C24 H47 N O7 P2 S	555.2563	4.54								0	level5						other
4.608_589.1976	2.0789	0.0124		C38 H27 N3 O4	589.1976	4.608								0	level5						up
4.609_577.2753	2.1426	0.023		C36 H39 N3 O2 S	577.2753	4.609								3	level5						up
4.609_589.7004	1.8302	0.0193			589.7004	4.609								0	level5						up
4.62_385.2313	1.2079	0.0071		C20 H37 N O2 P2	385.2313	4.62								0	level5						up
4.621_311.1944	1.6564	0.0594		C13 H29 N O7	311.1944	4.621								0	level5						other
4.662_221.09	2.4164	0.0939	Shrimp shell	C8 H15 N O6	221.09	4.662		S1376695	HMDB0062641	0.0001	0.4144			0	level4	Organic oxygen compounds	Organooxygen compounds	Carbohydrates and carbohydrate conjugates	Carbohydrates		other
4.663_328.1845	2.163	0.0976		C12 H28 N2 O8	328.1845	4.663								0	level5						other
4.664_283.1266	2.37	0.0991		C10 H21 N O8	283.1266	4.664								0	level5						other
4.687_340.2096	1.3546	0.1314		C16 H28 N4 O4	340.2096	4.687								0	level5						other
4.688_357.2362	1.3614	0.1355		C16 H31 N5 O4	357.2362	4.688								0	level5						other
4.697_455.2732	2.5904	0.0194		C21 H37 N5 O6	455.2732	4.697								5	level5						up
4.835_429.2575	1.2774	0.1453		C18 H39 N O10	429.2575	4.835								6	level5						other
4.885_324.1532	4.881	0.0534	Valaciclovir	C13 H20 N6 O4	324.1532	4.885	C07184	S54770	HMDB0014716	-0.0014	-4.4189			0	level4	Organic acids and derivatives	Carboxylic acids and derivatives	Amino acids, peptides, and analogues	Amino acids, peptides, and analogues	map04976 Bile secretion;	other
4.93_279.1093	1.2043	0.0104		C8 H18 N5 O4 P	279.1093	4.93								0	level5						up
4.947_515.857	2.5285	0.0626		C19 H2 O10 P2 S2	515.857	4.947								0	level5						other
4.967_327.1529	2.2014	0.0859		C12 H25 N O9	327.1529	4.967								0	level5						other
4.969_372.2107	2.1419	0.0673		C23 H32 O2 S	372.2107	4.969								0	level5						other
5.02_473.2838	1.2079	0.1123		C24 H45 N O4 P2	473.2838	5.02								0	level5						other
5.021_271.6629	2.2103	0.0116	[similar to: idarubicin; δ mass: -225.5057 da]		271.6629	5.021								0	level5						up
5.101_604.3073	0.5339	0.0062		C25 H54 N2 O8 P2 S	604.3073	5.101								0	level5						down
5.121_368.1794	4.7566	0.0588		C14 H28 N2 O9	368.1794	5.121								0	level5						other
5.165_604.3791	23.3861	0.0568		C27 H52 N6 O9	604.3791	5.165								0	level5						other
5.182_517.3102	1.2168	0.0126		C22 H47 N O12	517.3102	5.182								0	level5						up
5.191_416.237	2.1449	0.0542		C17 H32 N6 O6	416.237	5.191								0	level5						other
5.193_371.1791	2.252	0.0792		C14 H29 N O10	371.1791	5.193								0	level5						other
5.306_131.0406	2.9811	0.0117		C5 H9 N O S	131.0406	5.306								0	level5						up
5.316_398.2376	1.1528	0.1819		C16 H38 N4 O3 S2	398.2376	5.316								0	level5						other
5.38_429.1847	1.9322	0.0784		C16 H31 N O12	429.1847	5.38								0	level5						other
5.384_533.3416	1.3742	0.0241		C27 H53 N O5 P2	533.3416	5.384								0	level5						up
5.391_460.2633	2.3365	0.0478	Sophoranone	C30 H36 O4	460.2633	5.391	C08719	S390366		0.0019	4.2223			0	level4	Flavonoids	Flavonoids	Flavanones	Flavonoids	NULL	up
5.392_415.2054	2.3218	0.0826		C17 H29 N5 O7	415.2054	5.392								0	level5						other
5.453_414.2469	0.6903	0.0287		C18 H38 O10	414.2469	5.453								0	level5						down
5.492_386.2086	3.1962	0.0036	Anecortave	C23 H30 O5	386.2086	5.492		S99892		-0.0008	-1.9459			0	level4						up
5.494_131.0406	3.361	0.0076		C5 H9 N O S	131.0406	5.494								0	level5						up
5.497_442.264	1.243	0.0725		C17 H38 N4 O9	442.264	5.497								0	level5						other
5.546_473.2111	1.9663	0.0639		C18 H35 N O13	473.2111	5.546								0	level5						other
5.549_368.2285	30.346	0.003		C13 H33 N6 O4 P	368.2285	5.549								0	level5						up
5.556_502.2994	1.2789	0.0707		C22 H46 O12	502.2994	5.556								0	level5						other
5.557_504.2898	2.4829	0.0637		C30 H40 N4 O S	504.2898	5.557								0	level5						other
5.559_459.2317	2.2496	0.0891		C18 H37 N O12	459.2317	5.559								0	level5						other
5.653_486.2905	1.1982	0.1992	(5z,8z,11z,14z)-n-[2-(4-sulfamoylphenyl)ethyl]-5,8,11,14-icosatetraenamide	C28 H42 N2 O3 S	486.2905	5.653		S4446554		-0.0011	-2.3264			0	level4						other
5.655_131.0406	3.0897	0.017		C5 H9 N O S	131.0406	5.655								4	level5						up
5.698_503.2582	2.6026	0.0442		C21 H37 N5 O9	503.2582	5.698								0	level5						up
5.7_548.3162	2.4108	0.0706		C31 H48 O6 S	548.3162	5.7								0	level5						other
5.701_520.2848	3.822	0.0018		C20 H44 N2 O13	520.2848	5.701								0	level5						up
5.729_129.058	0.4259	0.0316	Quinoline	C9 H7 N	129.058	5.729	C06413	MReference-8753	HMDB0033731	0.0001	0.9532		83.7	0	level2	Organoheterocyclic compounds	Quinolines and derivatives	null	Quinolines and derivatives	NULL	down
5.789_474.2613	2.8926	0.0189	12-deoxyphorbol 20-acetate 13-(2-methylbutanoate)	C27 H38 O7	474.2613	5.789	C09086	S171403		-0.0005	-1.0127			0	level4	Terpenoids	Diterpenoids (C20)	Tiglianes	Terpenoids	NULL	up
5.826_547.2842	2.3631	0.078		C22 H45 N O14	547.2842	5.826								0	level5						other
5.827_193.0597	3.7746	0.1578	Pkf118-310	C7 H7 N5 O2	193.0597	5.827	C16789	S59912		-0.0003	-1.3527			1	level4					NULL	other
5.827_592.3421	2.5474	0.0655	Plazomicin	C25 H48 N6 O10	592.3421	5.827		S26390008		-0.0011	-1.8888			3	level4						other
5.907_518.2876	3.0074	0.0036	Epelsiban	C30 H38 N4 O4	518.2876	5.907		S9809717		-0.0017	-3.3632			0	level4						up
5.908_131.0406	3.6568	0.0108		C5 H9 N O S	131.0406	5.908								0	level5						up

5.925_622.3161	2.0775	0.0806		C22 H53 N6 O8 P3	622.3161	5.925								0	level5						other
5.935_591.3103	2.4637	0.0689	Fluphenazine depot	C32 H44 F3 N3 O2 S	591.3103	5.935	C07956	S3271		-0.0004	-0.6211			2	level4					NULL	other
6.014_562.3137	3.1875	0.0068		C26 H51 N4 O3 P S2	562.3137	6.014								0	level5						up
6.034_635.3367	2.4776	0.061		C27 H49 N5 O12	635.3367	6.034								1	level5						other
6.123_696.3896	2.5323	0.0569		C27 H61 N4 O14 P	696.3896	6.123								0	level5						other
6.125_679.3629	2.5262	0.0618	Thymopentin	C30 H49 N9 O9	679.3629	6.125		S397640		-0.0024	-3.5889			0	level4						other
6.205_740.4167	2.38	0.0838		C30 H64 N2 O18	740.4167	6.205								0	level5						other
6.207_723.3898	2.4134	0.0798		C29 H62 N3 O15 P	723.3898	6.207								0	level5						other
6.275_433.1908	4.1482	0.0957		C16 H36 N O8 P S	433.1908	6.275								0	level5						other
6.281_767.416	2.5033	0.0819		C36 H67 N O12 P2	767.416	6.281								0	level5						other
6.281_784.4426	2.4516	0.0832		C31 H69 N4 O16 P	784.4426	6.281								0	level5						other
6.322_131.0406	5.2908	0.0211		C5 H9 N O S	131.0406	6.322								0	level5						up
6.35_425.225	2.5088	0.0527		C16 H38 N5 O2 P3	425.225	6.35								0	level5						other
6.35_828.4695	2.5658	0.0703		C38 H74 N2 O13 P2	828.4695	6.35								0	level5						other
6.391_477.2172	4.2967	0.0872		C18 H40 N O9 P S	477.2172	6.391								0	level5						other
6.489_521.2435	4.4342	0.0851		C18 H39 N3 O14	521.2435	6.489								0	level5						other
6.574_565.2697	4.1095	0.0885	Coelichelin	C21 H39 N7 O11	565.2697	6.574	C15719	S2497530		-0.0011	-1.8729			0	level4					NULL	other
6.65_609.2958	3.9573	0.0898	Ergocristine	C35 H39 N5 O5	609.2958	6.65	C09164	S28873		0.0007	1.1714			0	level4	Alkaloids	Alkaloids derived from tryptophan and anthranilic acid	Indole alkaloids	Alkaloids	NULL	other
6.716_653.3226	3.9592	0.1114	Ro-32-2175	C37 H43 N5 O6	653.3226	6.716	C15661	S405782		0.0012	1.8727			3	level4						other
6.833_430.2528	2.4805	0.0858		C21 H40 N2 O3 P2	430.2528	6.833								0	level5						other
6.905_474.2791	2.5139	0.0713	Fluocortolone caproate	C28 H39 F O5	474.2791	6.905		S16735815		0.0009	1.9106			0	level4						other
6.969_518.3054	2.474	0.0743	(6e)-5-hydroxy-4-{[(1r,2s,5s)-2-hydroxy-5-isopropenyl-2-methylcyclopentyl]methyl}-6-[hydroxy(phenyl)methylene]-2,2-bis(3-methyl-2-buten-1-yl)-4-cyclohexene-1,3-dione	C33 H42 O5	518.3054	6.969		S4444886		0.0022	4.1946			0	level4						other
7.024_562.3316	2.5148	0.0576		C23 H50 N2 O13	562.3316	7.024								0	level5						other
7.074_606.3582	2.358	0.0808		C26 H50 N6 O10	606.3582	7.074								0	level5						other
7.161_694.4106	2.4479	0.0591		C29 H62 N2 O16	694.4106	7.161								0	level5						other
7.509_225.1367	2.9357	0.1694	2616	C12 H19 N O3	225.1367	7.509	C07129	S5210	HMDB0015009	0.0002	0.7671			0	level4	Benzenoids	Phenols	Benzenediols	Phenols and derivatives	NULL	other
7.512_195.1261	0.409	0.0988	4-(2-aminopropoxy)-3,5-dimethylphenol	C11 H17 N O2	195.1261	7.512		S84217	HMDB0060954	0.0002	1.1286			0	level4	Benzenoids	Phenols	4-alkoxyphenols	Phenols and derivatives		other
7.571_462.2984	3.8451	0.2129	Podecdysone b	C27 H42 O6	462.2984	7.571	C08832	S390409		0.0002	0.4465			0	level4	Terpenoids	Steroids	Cholestane	Terpenoids	NULL	other
7.676_122.0736	0.7041	0.0774	Phenylethyl alcohol	C8 H10 O	122.0736	7.676	C05853	S5830	HMDB0033944	0.0004	3.1563			0	level4	Benzenoids	Benzene and substituted derivatives	null	Benzene and derivatives	map00360 Phenylalanine metabolism;	other
7.83_473.278	0.0094	0.0971		C28 H35 N5 O2	473.278	7.83								0	level5						other
7.977_299.1522	2.8756	0.1265	Hydrocodone	C18 H21 N O3	299.1522	7.977	C08024	MReference-3246	HMDB0015091	0	0.115		48.4	0	level3	Alkaloids and derivatives	Morphinans	null	Alkaloids and derivatives	NULL	other
8.085_452.2928	0.1468	0.1685		C30 H36 N4	452.2928	8.085								0	level5						other
8.097_424.2977	0.0926	0.182	(1r,3s,5z)-5-[(2e)-2-{(1r,3as,7ar)-1-[(2r,3e,5e,7e)-9-hydroxy-3,5,7-nonatrien-2-yl]-7a-methyloctahydro-4h-inden-4-ylidene}ethylidene]-4-methylene-1,3-cyclohexanediol	C28 H40 O3	424.2977	8.097		S7826400		0	-0.0682			3	level4						other
8.097_470.3033	0.0382	0.1758	(2r,4ar,6bs,12as)-1-hydroxy-1,2,6b,9,9,12a-hexamethyl-10,13-dioxo-1,3,4,5,6,6a,6b,7,8,8a,9,10,11,12,12a,12b,13,14b-octadecahydro-4a(2h)-picenecarboxylic acid	C29 H42 O5	470.3033	8.097		S35014633	HMDB0038683	0.0001	0.132			0	level4	Organic oxygen compounds	Organooxygen compounds	Carbonyl compounds	Carbonyl compounds		other
8.18_176.1566	0.0084	0.1089	Heptylbenzene	C13 H20	176.1566	8.18		S13492	HMDB0061825	0.0001	0.809			1	level4	Benzenoids	Benzene and substituted derivatives	null	Benzene and derivatives		other
8.341_256.2191	0.3766	0.0593		C19 H28	256.2191	8.341								0	level5						other
8.44_475.3664	0.5828	0.0014		C23 H50 N5 O3 P	475.3664	8.44								0	level5						down
8.473_338.282	0.106	0.0987	Glycidyl oleate	C21 H38 O3	338.282	8.473	C19426	S4510739		-0.0001	-0.1919			0	level4					NULL	other

8.477_306.2559	0.1059	0.1503	8z,11z,14z-eicosatrienoic acid	C20 H34 O2	306.2559	8.477	C03242	MReference-349	HMDB0002925	0	-0.0127		77.8	0	level2	Lipids and lipid-like molecules	Fatty Acyls	Fatty acids and conjugates	Fatty acyls[FA]	map00591 Linoleic acid metabolism; map01040 Biosynthesis of unsaturated fatty acids; map01100 Metabolic pathways;	other
8.477_364.259	0.0915	0.0989		C16 H37 N4 O3 P	364.259	8.477								0	level5						other
8.555_342.2559	0.1698	0.0309	2475675	C23 H34 O2	342.2559	8.555		S4908583		0	-0.036			0	level4						down
8.555_360.2665	0.1679	0.0303	Drostanolone propionate	C23 H36 O3	360.2665	8.555		S194604		0	0.0318		52.1	0	level4						down
8.555_400.259	0.1497	0.0272		C20 H36 N2 O6	400.259	8.555								0	level5						down
8.583_448.319	5.6783	0.1243	Digitogenin	C27 H44 O5	448.319	8.583	C08896	S390462		0.0002	0.3483			0	level4	Steroids	27-Carbon atoms	Spirostan derivatives [Fig]	Sterol lipids[ST]	NULL	other
8.74_308.2714	0.0761	0.1336	Ethyl linoleate	C20 H36 O2	308.2714	8.74		S4445379		-0.0001	-0.2915			0	level4						other
9.053_450.2982	2.9594	0.1256		C27 H38 N4 O2	450.2982	9.053								6	level5						other
9.339_517.7534	2.2741	0.1123			517.7534	9.339								3	level5						other
9.416_275.1275	0.7291	0.0812	Alanyltryptophan	C14 H17 N3 O3	275.1275	9.416		S231456	HMDB0013209	0.0005	1.8089			0	level4	Organic acids and derivatives	Carboxylic acids and derivatives	Amino acids, peptides, and analogues	Amino acids, peptides, and analogues		other
0.595_530.0068	0.7525	0.0278		C9 H20 N6 O12 P2 S2	530.0068	0.595								10	level5						down
0.599_633.8687	0.717	0.1422			633.8687	0.599								0	level5						other
0.599_777.8316	0.584	0.0894			777.8316	0.599								0	level5						other
0.646_139.0609	1.7646	0.0184			139.0609	0.646								0	level5						up
0.657_212.999	0.4339	0.1579		C3 H10 N3 O2 P3	212.999	0.657								0	level5						other
0.657_270.9574	0.3736	0.1042		C6 H8 Cl N O5 P2	270.9574	0.657								0	level5						other
0.661_147.0532	0.7123	0.1088	L-glutamic acid	C5 H9 N O4	147.0532	0.661	C00025	MReference-470	HMDB0000148	0.0001	0.4215	81.6	90	5	level1	Organic acids and derivatives	Carboxylic acids and derivatives	Amino acids, peptides, and analogues	Amino acids	map00220 Arginine biosynthesis; map00250 Alanine, aspartate and glutamate metabolism; map00330 Arginine and proline metabolism; map00340 Histidine metabolism; map00430 Taurine and hypotaurine metabolism; map00471 D-Glutamine and D-glutamate metabolism; map00480 Glutathione metabolism; map00524 Neomycin, [illegible]	other
0.667_259.9664	0.4655	0.1281		C8 H7 Cl N2 O2 P2	259.9664	0.667								3	level5						other
0.667_317.9251	0.3326	0.0626		C4 H7 Cl N6 O S4	317.9251	0.667								13	level5						other
0.671_191.0171	0.3536	0.1854		C6 H10 N O2 P S	191.0171	0.671								0	level5						other
0.674_126.043	0.2083	0.0888	4-imidazoleacetic acid	C5 H6 N2 O2	126.043	0.674	C02835	BGI122	HMDB0002024	0.0001	0.8464	94.7	85.7	0	level1	Organoheterocyclic compounds	Azoles	Imidazoles	Imidazoles	map00340 Histidine metabolism; map01100 Metabolic pathways;	other
0.674_145.1103	1.3897	0.0532	Acetylcholine	C7 H15 N O2	145.1103	0.674	C01996	MReference-885	HMDB0000895	0.0001	0.3845		62.4	8	level2	Organic nitrogen compounds	Organonitrogen compounds	Quaternary ammonium salts	Quaternary ammonium salts	map00564 Glycerophospholipid metabolism; map04024 cAMP signaling pathway; map04080 Neuroactive ligand-receptor interaction; map04721 Synaptic vesicle cycle; map04725 Cholinergic synapse; map04742 Taste transduction; map04810 Regulation of actin cytoskeleton; map04911 Insulin secretion; map04970 Salivary secretion; map04971 Gastric acid [illegible]	other
0.675_202.1428	0.701	0.072	N,n-dimethylarginine	C8 H18 N4 O2	202.1428	0.675	C03626	S110375	HMDB0001539	-0.0002	-0.8982			15	level4	Organic acids and derivatives	Carboxylic acids and derivatives	Amino acids, peptides, and analogues	Amino acids, peptides, and analogues	NULL	other
0.699_185.1053	2.2536	0.0188	Otonecine	C9 H15 N O3	185.1053	0.699	C10356	S4445054		0.0001	0.6956			0	level4	Alkaloids	Alkaloids derived from ornithine	Pyrrolizidine alkaloids	Alkaloids	NULL	up
0.729_317.9623	1.3732	0.2039		C5 H11 Cl N6 S4	317.9623	0.729								1	level5						other
0.742_131.98	2.285	0.0492		C4 H5 O P S	131.98	0.742								0	level5						up
0.797_294.0589	0.2066	0.1268		C10 H14 O10	294.0589	0.797								0	level5						other
1.021_165.0651	0.5722	0.003	Epiguanine	C6 H7 N5 O	165.0651	1.021	C02242	S10883	HMDB0000897	0.0001	0.3862			13	level4	Organoheterocyclic compounds	Imidazopyrimidines	Purines and purine derivatives	Purines and derivatives	NULL	down
1.122_235.9909	1.7758	0.0585	5433281	C11 H6 Cl2 N2	235.9909	1.122	C14268	S82824		0.0001	0.2638			0	level4					NULL	other

1.214_181.074	1.8445	0.0981	L-tyrosine	C9 H11 N O3	181.074	1.214	C00082	MReference-2255	HMDB0000158	0.0001	0.7123	95.3	96.4	5	level1	Organic acids and derivatives	Carboxylic acids and derivatives	Amino acids, peptides, and analogues	Amino acids	map00130 Ubiquinone and other terpenoid-quinone biosynthesis; map00350 Tyrosine metabolism; map00360 Phenylalanine metabolism; map00400 Phenylalanine, tyrosine and tryptophan biosynthesis; map00730 Thiamine metabolism; map00970 Aminoacyl-tRNA biosynthesis; map01100 Metabolic pathways; map01210 2-Oxocarboxylic acid metabolism; [illegible]	other
1.236_129.0427	0.3665	0.1448	Pyroglutamate	C5 H7 N O3	129.0427	1.236	C01879	BGI491	HMDB0000267	0.0001	0.5196	93.5	87.3	1	level1	Peptides	Amino acids	Other amino acids [Fig]	Amino acids	map00480 Glutathione metabolism; map01100 Metabolic pathways;	other
1.346_134.048	2.2987	0.0982		C7 H6 N2 O	134.048	1.346								6	level5						other
1.874_151.0495	2.4621	0.1683	Guanine	C5 H5 N5 O	151.0495	1.874	C00242	MReference-436	HMDB0000132	0.0001	0.6682		97.1	0	level2	Organoheterocyclic compounds	Imidazopyrimidines	Purines and purine derivatives	Purines and derivatives	map00230 Purine metabolism; map01100 Metabolic pathways;	other
2.822_189.0395	1.8749	0.0167		C6 H11 N3 S2	189.0395	2.822							43.5	0	level5						up
3.164_256.1423	3.7193	0.1295	Pulcherriminic acid	C12 H20 N2 O4	256.1423	3.164	C20515	S10519216		0	-0.0044			6	level4					NULL	other
3.236_187.1314	3.2962	0.1532		C8 H17 N3 O2	187.1314	3.236								0	level5						other
3.303_131.0372	0.4273	0.0889		C8 H5 N O	131.0372	3.303								0	level5						other
3.392_330.1791	5.623	0.1265		C15 H26 N2 O6	330.1791	3.392								0	level5						other
3.403_186.0795	2.059	0.1014	4'-(imidazol-1-yl)acetophenone	C11 H10 N2 O	186.0795	3.403		MReference-284		0.0002	1.0722		32.1	3	level3						other
3.424_235.1421	2.1691	0.0222		C10 H21 N O5	235.1421	3.424								0	level5						up
3.476_232.1215	7.3363	0.016	Melatonin	C13 H16 N2 O2	232.1215	3.476	C01598	S872	HMDB0001389	0.0003	1.4211			0	level4	Organoheterocyclic compounds	Indoles and derivatives	Indoles	Indoles	map00380 Tryptophan metabolism; map01100 Metabolic pathways; map04080 Neuroactive ligand-receptor interaction; map04713 Circadian entrainment;	up
3.49_238.1417	1.1321	0.0748		C10 H22 O6	238.1417	3.49								0	level5						other
3.632_165.0427	0.3015	0.1214	Am9627000	C8 H7 N O3	165.0427	3.632	C02803	S21106581		0.0001	0.9008			6	level4					NULL	other
3.763_279.1681	2.4638	0.0435		C12 H25 N O6	279.1681	3.763								1	level5						up
3.763_324.2259	2.549	0.0201		C15 H28 N6 O2	324.2259	3.763								0	level5						up
3.835_335.0788	1.2947	0.0566	S-formylglutathione	C11 H17 N3 O7 S	335.0788	3.835	C01031	S164320	HMDB0001550	0.0001	0.3483		64.7	3	level4	Organic acids and derivatives	Carboxylic acids and derivatives	Amino acids, peptides, and analogues	Amino acids, peptides, and analogues	map01100 Metabolic pathways;	other
4.051_323.1944	2.3895	0.0177		C14 H29 N O7	323.1944	4.051								2	level5						up
4.129_243.0896	2.8325	0.1601	Alonimid	C14 H13 N O3	243.0896	4.129		S16932		0	0.0247			5	level4						other
4.224_158.0369	2.597	0.0428	1,4-naphthoquinone	C10 H6 O2	158.0369	4.224	C02617	S8215		0.0002	1.0321			0	level4					map00980 Metabolism of xenobiotics by cytochrome P450;	up
4.26_253.0798	1.9218	0.0996		C8 H15 N O8	253.0798	4.26								0	level5						other
4.273_239.1006	2.2691	0.114	2,3,4,5,6-pentahydroxy-n-(2-hydroxyethyl)hexanamide	C8 H17 N O7	239.1006	4.273		S196803	HMDB0032293	0.0001	0.3575			3	level4	Organic nitrogen compounds	Organonitrogen compounds	Amines	Amines		other
4.295_367.2207	2.6019	0.0247		C16 H33 N O8	367.2207	4.295								0	level5						up
4.493_289.1395	3.3794	0.0177	Arg-asp	C10 H19 N5 O5	289.1395	4.493		S7993183		0.0009	3.0263			1	level4						up
4.508_411.247	2.5445	0.0144		C19 H33 N5 O5	411.247	4.508								0	level5						up
4.569_259.1784	3.384	0.2087	Hexanoylcarnitine	C13 H25 N O4	259.1784	4.569		MReference-883	HMDB0000705	0.0001	0.2098		42.9	0	level3						other
4.613_553.7362	2.0203	0.0194			553.7362	4.613								4	level5						up
4.764_489.3139	2.0665	0.1742		C22 H43 N5 O7	489.3139	4.764								0	level5						other
4.818_210.1258	0.6823	0.0803	Jasmonic acid	C12 H18 O3	210.1258	4.818	C08491	MReference-457	HMDB0032797	0.0002	0.8373		62.3	0	level2	Lipids and lipid-like molecules	Fatty Acyls	Lineolic acids and derivatives	Fatty acyls[FA]	map00592 alpha-Linolenic acid metabolism; map01100 Metabolic pathways;	other
4.85_310.1852	1.2713	0.0894		C11 H26 N4 O6	310.1852	4.85								2	level5						other
4.869_499.2996	2.4049	0.0125		C23 H41 N5 O7	499.2996	4.869								0	level5						up
4.947_516.1914	2.0885	0.1259	Muraglitazar	C29 H28 N2 O7	516.1914	4.947		S178524		0.0017	3.3108			0	level4						other
5.022_543.3258	2.3485	0.0134		C25 H45 N5 O8	543.3258	5.022								14	level5						up
5.023_560.3523	2.3565	0.0185	Deferoxamine	C25 H48 N6 O8	560.3523	5.023	C06940	S2867	HMDB0014884	-0.001	-1.8593			0	level4	Organic acids and derivatives	Carboxylic acids and derivatives	Carboxylic acid derivatives	Organic acids	NULL	up
5.081_546.3254	1.1151	0.1429		C24 H50 O13	546.3254	5.081								0	level5						other
5.102_399.2469	1.5016	0.1289		C17 H37 N O9	399.2469	5.102								1	level5						other

5.16_293.6759	2.1606	0.0133	[similar to: idarubicin; δ mass: -203.4926 da]		293.6759	5.16								1	level5						up
5.161_587.3518	2.2383	0.0152		C30 H55 N O6 P2	587.3518	5.161								0	level5						up
5.184_465.2244	1.5971	0.0196	Codonocarpine	C26 H31 N3 O5	465.2244	5.184	C10582	S4445131		-0.0019	-4.1633			0	level4					NULL	up
5.236_480.2046	4.2111	0.1201		C18 H32 N4 O11	480.2046	5.236								4	level5						other
5.242_158.1096	4.4217	0.1311		C12 H14	158.1096	5.242								1	level5						other
5.285_324.2024	2.071	0.0221		C11 H29 N6 O3 P	324.2024	5.285								0	level5						up
5.38_446.2112	1.9164	0.0808		C16 H34 N2 O12	446.2112	5.38								0	level5						other
5.547_490.2376	1.8848	0.0762	2-(2,4-dihydroxyphenyl)-3-[(2e)-3,7-dimethyl-2,6-octadien-1-yl]-5,7-dihydroxy-6-(3-methyl-2-buten-1-yl)-4h-chromen-4-one	C30 H34 O6	490.2376	5.547		S8430495	HMDB0030630	0.0021	4.2101			0	level4	Phenylpropanoids and polyketides	Flavonoids	Flavones	Flavonoids		other
5.554_722.4309	1.1914	0.0473		C33 H62 N4 O13	722.4309	5.554								5	level5						other
5.555_388.1742	1.119	0.0851	12-o-î²-d-glucopyranosyloxyjasmonic acid	C18 H28 O9	388.1742	5.555	C08558	S4444622		0.0009	2.2649			4	level4	FA Fatty acyls	FA13 Fatty acyl glycosides	FA1301 Fatty acyl glycosides of mono- and disaccharides	Fatty acyls[FA]	NULL	other
5.559_540.2972	1.4263	0.0246		C26 H44 N4 O6 S	540.2972	5.559								0	level5						up
5.601_390.2416	2.1724	0.0118	Compactin	C23 H34 O5	390.2416	5.601	C13963	S58262		0.001	2.5644			6	level4						up
5.691_517.2374	2.028	0.111		C21 H35 N5 O10	517.2374	5.691								1	level5						other
5.691_534.264	1.9959	0.1183	Pyropheophorbide a	C33 H34 N4 O3	534.264	5.691	C18064	S10381282		0.001	1.7915			0	level4	Marine biotoxins	Others	Others	Marine biotoxins	map00860 Porphyrin and chlorophyll metabolism;	other
5.815_578.29	1.9839	0.1015		C21 H47 N4 O12 P	578.29	5.815								0	level5						other
5.816_561.2634	2.039	0.0971	Indometacin farnesil	C34 H40 Cl N O4	561.2634	5.816		S4445378		-0.0012	-2.1353			0	level4						other
5.884_260.0685	0.6024	0.0093	Khellin	C14 H12 O5	260.0685	5.884	C09010	S3696		0	-0.0206			6	level4	Polyketides	Pyrones	Chromones	Polyketides[PK]		down
5.957_345.1384	3.8886	0.1368		C10 H23 N3 O10	345.1384	5.957								0	level5						other
6.017_131.0406	4.1268	0.0216		C5 H9 N O S	131.0406	6.017								0	level5						up
6.028_666.3429	1.9822	0.088		C27 H60 N2 O10 P2 S	666.3429	6.028								0	level5						other
6.131_389.1646	3.926	0.1098		C13 H31 N3 O6 S2	389.1646	6.131								0	level5						other
6.28_403.2119	2.2296	0.0819		C13 H35 N5 O5 P2	403.2119	6.28								2	level5						other
6.975_276.1362	0.4818	0.1374	2-(1-hydroxy-2,4,6-trimethyl-3-oxo-2,3-dihydro-1h-inden-5-yl)ethyl acetate	C16 H20 O4	276.1362	6.975		S4475195	HMDB0030764	0	0.0996			3	level4	Benzenoids	Indanes	Indanones	Benzene and derivatives		other
6.976_164.0839	0.4473	0.1282	4-phenylbutyric acid	C10 H12 O2	164.0839	6.976	C21793	MReference-3074	HMDB0000543	0.0002	0.9632		89.2	5	level2	Benzenoids	Benzene and substituted derivatives	null	Benzene and derivatives	NULL	other
7.095_268.1311	0.148	0.0932	Ripazepam	C15 H16 N4 O	268.1311	7.095	C19518	S30896		-0.0013	-4.8185			2	level4					NULL	other
7.215_266.1155	0.1551	0.047	Nevirapine	C15 H14 N4 O	266.1155	7.215	C07263	S4308	HMDB0014383	-0.0012	-4.6405			8	level4	Organic nitrogen compounds	Organonitrogen compounds	Amines	Amines	NULL	down
7.461_253.1681	6.7929	0.0362	(1r,5r)-3,3,5-trimethylcyclohexyl 5-oxo-l-prolinate	C14 H23 N O3	253.1681	7.461		S8035531		0.0003	1.1314			0	level4						up
7.906_457.3404	0.0248	0.084		C25 H47 N O6	457.3404	7.906								0	level5						other
7.941_194.1672	0.3567	0.0397	Theaspirane	C13 H22 O	194.1672	7.941		S55810	HMDB0036823	0.0002	0.8437			8	level4	Organoheterocyclic compounds	Tetrahydrofurans	null	Tetrahydrofurans		down
8.248_228.1878	0.2114	0.1363		C17 H24	228.1878	8.248								0	level5						other
8.248_242.2034	0.2241	0.1494		C18 H26	242.2034	8.248								0	level5						other
8.248_437.3506	0.2762	0.1635		C26 H47 N O4	437.3506	8.248								2	level5						other
8.248_806.5684	0.0851	0.1245		C39 H79 N6 O9 P	806.5684	8.248								3	level5						other
8.251_414.2746	0.332	0.1636		C22 H34 N6 O2	414.2746	8.251								0	level5						other
8.363_373.3093	0.2757	0.0575		C21 H44 N O2 P	373.3093	8.363								5	level5						other
8.485_390.277	0.1943	0.0613	Bis(2-ethylhexyl) phthalate	C24 H38 O4	390.277	8.485	C03690	S21106505		0	-0.058			6	level4					NULL	other
8.485_435.335	0.1415	0.0851		C20 H46 N5 O3 P	435.335	8.485								2	level5						other
8.493_412.2588	0.2041	0.0832		C20 H37 N4 O3 P	412.2588	8.493								0	level5						other
8.744_414.3135	1.8893	0.1325	Diosgenin	C27 H42 O3	414.3135	8.744	C08898	S89870		0.0001	0.1642			2	level4	Steroids	27-Carbon atoms	Spirostan derivatives [Fig]	Sterol lipids[ST]		other
8.754_377.2332	0.6799	0.1156		C19 H32 N5 O P	377.2332	8.754								0	level5						other
9.066_422.3187	0.2505	0.1429		C29 H42 O2	422.3187	9.066								0	level5						other
9.066_458.3398	0.2271	0.1124	18-acetoxy-1alpha-hydroxyvitamin d3	C29 H46 O4	458.3398	9.066		S7826457		0.0002	0.4134			0	level4						other
9.097_440.3292	0.2719	0.1005	Estradiol undecylate	C29 H44 O3	440.3292	9.097		S18055		0.0002	0.4357			17	level4						other
9.098_263.2613	0.5502	0.0471		C18 H33 N	263.2613	9.098								0	level5						down
9.323_239.0923	0.6269	0.1149		C8 H18 N O5 P	239.0923	9.323								0	level5						other
9.324_1012.6486	0.644	0.1409		C56 H95 N4 O6 P3	1012.6486	9.324								0	level5						other
9.425_522.7941	0.6663	0.0742		C4 H Cl N3 O17 P3 S	522.7941	9.425								0	level5						other
9.426_1012.6488	0.7978	0.0568		C44 H99 N6 O13 P3	1012.6488	9.426								1	level5						other
9.684_342.1867	0.6527	0.0183		C20 H27 N2 O P	342.1867	9.684								2	level5						down
9.866_465.3219	0.3287	0.0806		C25 H43 N3 O5	465.3219	9.866								0	level5						other

0.597_973.8079	0.7069	0.2169			973.8079	0.597								0	level5						other
0.598_326.044	0.8016	0.0639	6,8,10-trihydroxy-3a,12c-dihydro-7h-furo[3',2':4,5]furo[2,3-c]xanthen-7-one	C17 H10 O7	326.044	0.598		S74854141	HMDB0140672	0.0014	4.214			0	level4	Phenylpropanoids and polyketides	Sterigmatocystins	null	Polyketides[PK]		other
0.598_701.8562	0.7094	0.1762			701.8562	0.598								0	level5						other
0.598_769.8439	0.7067	0.1846			769.8439	0.598								0	level5						other
0.598_837.8314	0.694	0.1688			837.8314	0.598								0	level5						other
0.598_905.8193	0.682	0.1547			905.8193	0.598								0	level5						other
0.6_565.8812	0.7275	0.1474		C5 H8 N6 O18 P2 S2	565.8812	0.6								0	level5						other
0.6_770.8461	0.7199	0.1395		C26 H4 N3 O18 P3 S	770.8461	0.6								0	level5						other
0.601_429.9063	0.7554	0.1462		C6 H5 N6 O9 P3 S	429.9063	0.601								0	level5						other
0.601_497.8939	0.7448	0.1605		C5 H3 N6 O18 P S	497.8939	0.601								0	level5						other
0.602_361.9189	0.7546	0.1398		C4 H3 N4 O12 P S	361.9189	0.602								0	level5						other
0.603_293.9314	0.7494	0.1419		C3 H8 N2 O6 P2 S2	293.9314	0.603								0	level5						other
0.607_157.9568	0.7741	0.1514			157.9568	0.607								8	level5						other
0.607_225.9441	0.7639	0.1077	(2e)-2,5-dichloro-4-oxo-2-hexenedioic acid	C6 H4 Cl2 O5	225.9441	0.607	C12835	S4445367	HMDB0060363	0.0005	2.4113			10	level4	Organic acids and derivatives	Keto acids and derivatives	Medium-chain keto acids and derivatives	Organic acids	map01100 Metabolic pathways;	other
0.636_133.9957	0.7631	0.0685		C4 H7 O P S	133.9957	0.636								0	level5						other
0.645_146.9967	0.4371	0.1304	[similar to: l-glutamic acid; δmass: -0.0565 da]		146.9967	0.645								7	level5						other
0.659_145.0739	2.8427	0.1784	4-acetamidobutanoate	C6 H11 N O3	145.0739	0.659	C02946	BGI113	HMDB0003681	0	0.1679	24.9		0	level3	Organic acids and derivatives	Carboxylic acids and derivatives	Amino acids, peptides, and analogues	Amino acids, peptides, and analogues	map00330 Arginine and proline metabolism; map01100 Metabolic pathways;	other
0.66_222.0852	2.3527	0.0277		C7 H14 N2 O6	222.0852	0.66								0	level5						up
0.666_202.0089	0.4285	0.1055		C3 H2 N6 O5	202.0089	0.666								0	level5						other
0.667_251.1004	3.5499	0.0341	2'-deoxyadenosine	C10 H13 N5 O3	251.1004	0.667								0	level5						up
0.691_265.0675	0.513	0.1296		C8 H18 N3 O P3	265.0675	0.691								0	level5						other
0.703_130.1107	1.8274	0.1649	Oryzalin metabolite	C6 H14 N2 O	130.1107	0.703	C19279	S11632		0.0001	0.8218			2	level4					NULL	other
0.711_143.1422	0.0871	0.152	1-methyl-4-(2-aminoethyl)piperazine	C7 H17 N3	143.1422	0.711		S63468	HMDB0062673	-0.0001	-0.5316			0	level4	Organoheterocyclic compounds	Diazinanes	Piperazines	Piperazines		other
0.723_180.0263	0.6083	0.305		C9 H10 P2	180.0263	0.723								0	level5						other
0.729_141.0402	1.4586	0.2264			141.0402	0.729								0	level5						other
0.729_290.0626	3.076	0.073		C9 H17 N4 O P3	290.0626	0.729								9	level5						other
0.737_323.0955	3.3983	0.2292		C10 H17 N3 O9	323.0955	0.737								0	level5						other
0.777_191.9545	2.7232	0.1248		C8 H Cl N2 S	191.9545	0.777								2	level5						other
0.804_169.0174	2.2151	0.1792			169.0174	0.804								0	level5						other
1.018_232.9749	0.6267	0.2267		C5 H5 N3 O4 P2	232.9749	1.018								0	level5						other
1.02_204.98	0.5391	0.1592		C4 H5 N3 O3 P2	204.98	1.02								0	level5						other
1.272_122.0369	1.7623	0.0214	4-hydroxybenzaldehyde	C7 H6 O2	122.0369	1.272	C00633	BGI119	HMDB0011718	0.0001	0.6736	92.2	91.2	2	level2	Organic oxygen compounds	Organooxygen compounds	Carbonyl compounds	Carbonyl compounds	map01100 Metabolic pathways;	up
1.298_548.209	0.3179	0.0772		C18 H37 N4 O13 P	548.209	1.298								0	level5						other
1.642_144.0246	4.3526	0.1417		C6 H8 O2 S	144.0246	1.642								0	level5						other
1.926_183.0533	0.6968	0.0254	4-pyridoxate	C8 H9 N O4	183.0533	1.926	C00847	BGI126	HMDB0000017	0.0001	0.7165	98.6	93.5	0	level1	Organoheterocyclic compounds	Pyridines and derivatives	Pyridinecarboxylic acids and derivatives	Pyridine and derivatives	map00750 Vitamin B6 metabolism; map01100 Metabolic pathways;	down
2.577_174.1092	0.6096	0.1549			174.1092	2.577								0	level5						other
2.624_110.0738	0.7384	0.0875	Trans,trans-2,4-heptadienal	C7 H10 O	110.0738	2.624							62.8	0	level5						other
2.701_170.1533	2.3685	0.0333		C8 H18 N4	170.1533	2.701								0	level5						up
2.745_189.0396	1.8073	0.0403		C6 H11 N3 S2	189.0396	2.745							43.5	0	level5						up
2.775_170.1532	1.9796	0.0672		C8 H18 N4	170.1532	2.775								1	level5						other
2.894_133.0529	0.6859	0.1424	2-oxindole	C8 H7 N O	133.0529	2.894	C12312	MReference-616	HMDB0061918	0.0001	0.6929		74.9	0	level2	Organoheterocyclic compounds	Indoles and derivatives	Indolines	Indole and derivatives		other
3.116_329.1588	2.6247	0.1613	Valclavam	C14 H23 N3 O6	329.1588	3.116		S389926		0.0001	0.2232			0	level4						other
3.163_216.1408	2.8504	0.0143		C9 H20 N4 S	216.1408	3.163								0	level5						up
3.163_84.0688	5.1972	0.0527	Al9450000	C4 H8 N2	84.0688	3.163		S21171402	HMDB0061875	0.0001	0.974			0	level4	Organic nitrogen compounds	Organonitrogen compounds	Amines	Amines		other
3.21_149.0781	0.7898	0.1551			149.0781	3.21								0	level5						other
3.211_135.0988	0.7779	0.103			135.0988	3.211								4	level5						other
3.211_163.116	0.7615	0.1131			163.116	3.211								2	level5						other
3.211_191.0884	0.7752	0.1057			191.0884	3.211								0	level5						other
3.211_209.121	0.7877	0.1241			209.121	3.211								0	level5						other
3.212_190.0824	0.7826	0.1239			190.0824	3.212								0	level5						other
3.242_204.1705	0.2433	0.2301		C7 H20 N6 O	204.1705	3.242								0	level5						other
3.421_297.0896	2.0456	0.193	5'-s-methyl-5'-thioadenosine	C11 H15 N5 O3 S	297.0896	3.421	C00170	MReference-253	HMDB0001173	0.0001	0.2182	78.3	86.6	0	level2	Nucleosides, nucleotides, and analogues	5'-deoxyribonucleosides	5'-deoxy-5'-thionucleosides	Nucleic acids and analogues	map00270 Cysteine and methionine metabolism; map01100 Metabolic pathways;	other

3.438_191.0583	0.223	0.1671	5-hydroxyindoleacetate	C10 H9 N O3	191.0583	3.438	C05635	BGI131	HMDB0000763	0.0001	0.5709	95.8	90	0	level1	Organoheterocyclic compounds	Indoles and derivatives	Indolyl carboxylic acids and derivatives	Indole and derivatives	map00380 Tryptophan metabolism; map01100 Metabolic pathways; map04726 Serotonergic synapse;	other
3.439_131.0372	0.2011	0.1982		C8 H5 N O	131.0372	3.439								0	level5						other
3.439_159.0322	0.2277	0.1516		C9 H5 N O2	159.0322	3.439								5	level5						other
3.439_219.0533	0.2278	0.1636	8-methoxykynurenic acid	C11 H9 N O4	219.0533	3.439	C05830	S68710	HMDB0060426	0.0001	0.5482			1	level4	Organoheterocyclic compounds	Quinolines and derivatives	Quinoline carboxylic acids	Quinoline carboxylic acids	map00380 Tryptophan metabolism;	other
3.44_205.0377	0.2106	0.1692	Xanthurenic acid	C10 H7 N O4	205.0377	3.44	C02470	MReference-789	HMDB0000881	0.0002	0.9213	39.6	73.8	0	level1	Organoheterocyclic compounds	Quinolines and derivatives	Quinoline carboxylic acids	Quinoline carboxylic acids	map00380 Tryptophan metabolism;	other
3.44_220.0611	0.2015	0.1795		C9 H18 P2 S	220.0611	3.44								0	level5						other
3.449_87.0144	1.9215	0.1129		C3 H5 N S	87.0144	3.449								0	level5						other
3.582_260.21	3.17	0.1739		C13 H28 N2 O3	260.21	3.582								0	level5						other
3.692_310.1528	4.9045	0.0743		C16 H26 N2 S2	310.1528	3.692							72.1	5	level5						other
3.772_437.1933	1.4265	0.0423		C17 H32 N3 O8 P	437.1933	3.772								1	level5						up
3.788_209.1053	3.1782	0.175	Propoxur	C11 H15 N O3	209.1053	3.788	C14334	S4775		0.0001	0.3384			0	level4					NULL	other
3.79_207.0533	2.7969	0.1917	4-(2-aminophenyl)-2,4-dioxobutanoic acid	C10 H9 N O4	207.0533	3.79	C01252	S459	HMDB0000978	0.0001	0.5666			0	level4	Organic oxygen compounds	Organooxygen compounds	Carbonyl compounds	Carbonyl compounds	map00380 Tryptophan metabolism; map01100 Metabolic pathways;	other
3.819_324.168	6.0986	0.0911	Tricetamide	C16 H24 N2 O5	324.168	3.819		S61082		-0.0005	-1.5984			3	level4						other
3.832_282.0398	1.5208	0.6083		C5 H12 N6 O4 P2	282.0398	3.832								5	level5						other
3.834_168.0435	1.1317	0.1214			168.0435	3.834								1	level5						other
3.834_282.1674	1.0857	0.2078	Mm3670000	C12 H26 O7	282.1674	3.834		S16521	HMDB0061822	-0.0004	-1.5027			2	level4	Organic oxygen compounds	Organooxygen compounds	Ethers	Ethers		other
3.884_260.1129	10.2136	0.0756	Gln-asn	C9 H16 N4 O5	260.1129	3.884		S16568296		0.0009	3.3617			0	level4						other
3.886_128.0409	10.1853	0.0517	2-thiazolylethylamine	C5 H8 N2 S	128.0409	3.886	C17927	S79076		0.0001	0.7161			4	level4					NULL	other
3.933_284.1008	5.6235	0.0807	(2r,3s,4s,5r)-2-(6-hydroxy-2-imino-3,7-dihydropurin-9-yl)-5-(hydroxymethyl)tetrahydrofuran-3,4-diol	C10 H14 N5 O5	284.1008	3.933		S30778620	HMDB0061067	0.0014	4.765			0	level4	Nucleosides, nucleotides, and analogues	Purine nucleosides	null	Purines and derivatives		other
3.946_188.0872	1.2935	0.0868		C9 H16 O2 S	188.0872	3.946								3	level5						other
4.169_415.278	0.5948	0.0689		C19 H37 N5 O5	415.278	4.169								0	level5						other
4.28_301.1889	3.4104	0.1556	Tegaserod	C16 H23 N5 O	301.1889	4.28		S10609889		-0.0013	-4.4471			2	level4						other
4.358_244.1787	0.6756	0.1342	Leu-leu	C12 H24 N2 O3	244.1787	4.358	C11332	S85053		0	0.1775			0	level4					NULL	other
4.444_296.1834	1.2467	0.146		C13 H28 O7	296.1834	4.444								0	level5						other
4.577_220.1312	1.2343	0.1092		C10 H20 O5	220.1312	4.577								0	level5						other
4.616_176.0799	1.6104	0.0992	Ala-ser	C6 H12 N2 O4	176.0799	4.616		S87421		0.0002	1.0795			4	level4						other
4.62_412.141	1.6383	0.155		C25 H20 N2 O4	412.141	4.62								1	level5						other
4.62_548.2084	1.9858	0.0544		C18 H37 N4 O13 P	548.2084	4.62								2	level5						other
4.645_180.1264	2.5457	0.1849	Rilmenidine	C10 H16 N2 O	180.1264	4.645	C11120	S61963		0.0001	0.5878			1	level4					NULL	other
4.645_208.1213	2.5216	0.2	Pilocarpine	C11 H16 N2 O2	208.1213	4.645	C07474	S5699	HMDB0015217	0.0002	0.799			1	level4	Alkaloids and derivatives	null	null	Alkaloids and derivatives		other
4.649_297.1059	1.8735	0.1157	Nelarabine	C11 H15 N5 O5	297.1059	4.649		S2280207	HMDB0015401	-0.0014	-4.7921			1	level4	Nucleosides, nucleotides, and analogues	Purine nucleosides	null	Purines and derivatives		other
4.649_314.1325	1.8347	0.1231		C11 H18 N6 O5	314.1325	4.649								3	level5						other
4.653_205.1077	1.856	0.1182			205.1077	4.653								0	level5						other
4.679_562.5668	3.9816	0.1603	[similar to: fumonisin b1; δmass: -158.8217 da]		562.5668	4.679								0	level5						other
4.947_497.2138	2.9613	0.1464		C22 H37 N5 O2 P2 S	497.2138	4.947								0	level5						other
4.947_515.5219	1.7485	0.2474	[similar to: (+/-)5(6)-dihet; δmass: 177.2762 da]		515.5219	4.947								1	level5						other
4.95_87.0144	3.8572	0.1099		C3 H5 N S	87.0144	4.95								1	level5						other
4.955_341.1322	1.7442	0.1051	6-(alpha-d-glucosaminyl)-1d-myo-inositol	C12 H23 N O10	341.1322	4.955	C15658	S21395593	HMDB0011668	0	-0.0865			13	level4	Organic oxygen compounds	Organooxygen compounds	Carbohydrates and carbohydrate conjugates	Carbohydrates	NULL	other
4.955_358.1588	1.6759	0.113		C12 H26 N2 O10	358.1588	4.955								0	level5						other
4.981_364.1917	1.3677	0.0433		C18 H28 N4 O2 S	364.1917	4.981								7	level5						up
5.012_131.0372	0.0527	0.1335		C8 H5 N O	131.0372	5.012								6	level5						other
5.084_290.189	0.8576	0.1203	Octinoxate	C18 H26 O3	290.189	5.084		S4511170	HMDB0061861	0.0009	2.9316			0	level4	Phenylpropanoids and polyketides	Cinnamic acids and derivatives	Cinnamic acid esters	Polyketides[PK]		other
5.084_445.2891	1.2229	0.1675	D-lysyl-l-valyl-d-valyl-l-allothreonine	C20 H39 N5 O6	445.2891	5.084		S30776665	HMDB0012936	-0.0009	-2.0627			0	level4	Organic acids and derivatives	Carboxylic acids and derivatives	Amino acids, peptides, and analogues	Amino acids, peptides, and analogues		other
5.111_354.2115	1.1514	0.1913		C13 H30 N4 O7	354.2115	5.111								0	level5						other
5.182_407.1403	2.1257	0.1026	Pagoclone	C23 H22 Cl N3 O2	407.1403	5.182		S116335		0.0003	0.6935			0	level4						other
5.183_402.1848	1.8186	0.0877	Clocinizine	C26 H27 Cl N2	402.1848	5.183		S4525064	HMDB0240242	-0.0014	-3.5492			1	level4	Benzenoids	Benzene and substituted derivatives	Diphenylmethanes	Benzene and derivatives		other
5.242_202.1359	4.2398	0.1404	Jasmonal	C14 H18 O	202.1359	5.242	C12288	S1361549	HMDB0031313	0.0001	0.4362			15	level4	Phenylpropanoids and polyketides	Cinnamaldehydes	null	Polyketides[PK]	NULL	other
5.246_248.1412	4.0649	0.1256	Amiloxate	C15 H20 O3	248.1412	5.246		S1266578		0	-0.0907			4	level4						other
5.285_631.378	2.1364	0.0366		C26 H60 N5 O6 P3	631.378	5.285								0	level5						up

5.332_561.3363	1.1568	0.1249		C24 H51 N O13	561.3363	5.332								10	level5						other
5.336_324.68	0.8949	0.1646	[similar to: (+/-)5(6)-dihet; δmass: -13.5657 da]		324.68	5.336								0	level5						other
5.463_605.3629	1.1772	0.048		C27 H51 N5 O10	605.3629	5.463								4	level5						other
5.56_476.2584	2.0205	0.1254	Betamethasone 17-valerate	C27 H37 F O6	476.2584	5.56		S15673		0.001	2.0199			21	level4						other
5.575_649.389	1.3851	0.0542		C26 H62 N5 O7 P3	649.389	5.575								3	level5						other
5.659_584.3232	1.3638	0.0569		C27 H52 O11 S	584.3232	5.659								6	level5						other
5.729_189.0791	0.427	0.0395	Indole-3-methyl acetate	C11 H11 N O2	189.0791	5.729	C20635	BGI325	HMDB0029738	0.0001	0.7162	84.3	82.9	14	level2	Organoheterocyclic compounds	Indoles and derivatives	Indolyl carboxylic acids and derivatives	Indole and derivatives	NULL	down
5.735_412.2548	1.1797	0.1308	[similar to: (+/-)5(6)-dihet; δmass: 74.0091 da]		412.2548	5.735								0	level5						other
5.816_449.2362	1.1091	0.279		C19 H35 N3 O9	449.2362	5.816								5	level5						other
5.816_871.5368	1.1888	0.0983		C41 H84 N3 O10 P3	871.5368	5.816								1	level5						other
6.649_108.094	0.7848	0.0282	4-vinylcyclohexene	C8 H12	108.094	6.649	C19310	S7218		0.0002	1.405			0	level4					NULL	down
7.347_271.1784	0.2313	0.1693		C14 H25 N O4	271.1784	7.347								1	level5						other
7.442_273.1452	0.341	0.0547			273.1452	7.442								1	level5						other
7.678_120.0214	0.6637	0.0593		C7 H4 O2	120.0214	7.678						29.7		0	level5						other
7.801_194.0957	1.8117	0.2901	[similar to: salvinorin b; δmass: -196.0721 da]		194.0957	7.801								0	level5						other
7.962_303.219	0.7944	0.131	Samandarone	C19 H29 N O2	303.219	7.962	C19951	S16736009		-0.0008	-2.6972			1	level4	Venoms	Others	Salamandra venoms	Venoms	NULL	other
8.248_194.1309	0.1963	0.1443	Hexylresorcinol	C12 H18 O2	194.1309	8.248		S21106121	HMDB0032567	0.0002	1.0008			1	level4	Benzenoids	Phenols	Benzenediols	Phenols and derivatives		other
8.248_409.3191	0.2609	0.1741		C25 H39 N5	409.3191	8.248								0	level5						other
8.25_214.1722	0.2513	0.1444		C16 H22	214.1722	8.25								0	level5						other
8.254_146.1096	0.1889	0.1666	3-methyl-1-phenyl-2-butene	C11 H14	146.1096	8.254		S19372	HMDB0061808	0.0001	0.3783			1	level4	Benzenoids	Benzene and substituted derivatives	null	Benzene and derivatives		other
8.324_409.3192	0.1542	0.2219		C24 H43 N O4	409.3192	8.324						77.8		1	level5						other
8.337_299.2824	0.3406	0.1805	D-sphingosine	C18 H37 N O2	299.2824	8.337	C00319	MReference-418	HMDB0000252	0	-0.1548		37.2	0	level3	Organic nitrogen compounds	Organonitrogen compounds	Amines	Amines	map00600 Sphingolipid metabolism; map01100 Metabolic pathways; map04071 Sphingolipid signaling pathway; map04210 Apoptosis; map04217 Necroptosis;	other
8.41_302.1858	0.4564	0.062		C15 H22 N6 O	302.1858	8.41							67	18	level5						other
8.477_431.3035	0.3054	0.064		C26 H41 N O4	431.3035	8.477						74.6		1	level5						other
8.477_449.3142	0.3079	0.0509	Glycochenodeoxycholate	C26 H43 N O5	449.3142	8.477	C05466	BGI629	HMDB0000637	0.0001	0.1926	46.7		9	level3	ST Sterol Lipids	ST04 Bile acids and derivatives	ST0402 C26 bile acids, alcohols, and derivatives	Sterol lipids[ST]	map00120 Primary bile acid biosynthesis; map04976 Bile secretion; map04979 Cholesterol metabolism;	other
8.581_264.209	0.3452	0.0631	2-(4-nonylphenoxy)ethanol	C17 H28 O2	264.209	8.581		S7414		0.0001	0.1904			0	level4						other
8.626_357.9727	2.8083	0.2318		C7 H11 Cl N6 O3 S3	357.9727	8.626								0	level5						other
8.753_188.087	1.3912	0.0929		C9 H16 O2 S	188.087	8.753								0	level5						other
8.761_596.8132	1.365	0.1407		C5 H6 N5 O17 P3 S3	596.8132	8.761								0	level5						other
8.761_641.3395	1.5132	0.0533	(3a,5b,7a,12a)-24-[(carboxymethyl)amino]-1,12-dihydroxy-24-oxocholan-3-yl-b-d-glucopyranosiduronic acid	C32 H51 N O12	641.3395	8.761		S58145726	HMDB0002472	-0.0016	-2.4957			0	level4	Organic nitrogen compounds	Organonitrogen compounds	Oximes	Oximes		other
8.917_342.217	0.3258	0.0548		C18 H26 N6 O	342.217	8.917								0	level5						other
8.918_365.2929	0.2816	0.0499	N-linoleoyl-4-aminobutyric acid	C22 H39 N O3	365.2929	8.918		S4942643	HMDB0062334	-0.0001	-0.1546			3	level4	Organic acids and derivatives	Carboxylic acids and derivatives	Amino acids, peptides, and analogues	Amino acids, peptides, and analogues		down
8.986_320.2349	0.2132	0.0852	(+/-)11(12)-eet	C20 H32 O3	320.2349	8.986		MReference-9807		-0.0002	-0.6803		62.7	3	level2						other
9.098_328.2402	0.1204	0.1055	Docosahexaenoic acid	C22 H32 O2	328.2402	9.098	C06429	MReference-6801	HMDB0002183	-0.0001	-0.2144		74.1	5	level2	Lipids and lipid-like molecules	Fatty Acyls	Fatty acids and conjugates	Fatty acyls[FA]	map01040 Biosynthesis of unsaturated fatty acids;	other

9.098_379.2488	0.5941	0.0746	D-erythro-sphingosine 1-phosphate	C18 H38 N O5 P	379.2488	9.098	C06124	MReference-1213	HMDB0000277	0	0.0637		49	5	level3	Lipids and lipid-like molecules	Sphingolipids	Phosphosphingolipids	Sphingolipids[SP]	map00600 Sphingolipid metabolism; map01100 Metabolic pathways; map04020 Calcium signaling pathway; map04071 Sphingolipid signaling pathway; map04072 Phospholipase D signaling pathway; map04080 Neuroactive ligand-receptor interaction; map04371 Apelin signaling pathway; map04666 Fc gamma R-mediated [illegible]	other
9.103_475.3664	0.4176	0.0388		C23 H50 N5 O3 P	475.3664	9.103								0	level5						down
9.324_183.0662	0.572	0.1583		C5 H14 N O4 P	183.0662	9.324								35	level5						other
9.371_478.3061	0.1714	0.1783		C31 H42 O4	478.3061	9.371								2	level5						other
9.42_183.0662	0.7302	0.1416		C5 H14 N O4 P	183.0662	9.42								0	level5						other
9.42_522.2925	0.6006	0.0608	[similar to: dl-dipalmitoylphosphatidylcholine; δ mass: -211.2697 da]		522.2925	9.42								2	level5						other
9.618_481.3532	0.473	0.1792	1-hexadecyllysophosphatidylcholine	C24 H52 N O6 P	481.3532	9.618	C13903	S3845		0	-0.0279			0	level4	GP Glycerophospholipids	GP01 Glycerophosphocholines	GP0106 Monoalkylglycerophosphocholines	Glycerophospholipids[GP]	NULL	other
9.627_463.3057	0.5152	0.0725		C22 H45 N3 O5 S	463.3057	9.627								5	level5						other
9.693_348.2037	0.7107	0.0294	Roxane	C19 H28 N2 O4	348.2037	9.693		S4926	HMDB0015695	-0.0012	-3.4798			0	level4	Organoheterocyclic compounds	Piperidines	Benzylpiperidines	Benzylpiperidines		down
9.694_529.3509	0.4161	0.1851		C22 H53 N5 O5 P2	529.3509	9.694								5	level5						other
9.723_529.372	0.6321	0.2624		C29 H58 N O P3	529.372	9.723								28	level5						other
9.87_300.204	0.6723	0.1033		C15 H28 N2 O4	300.204	9.87								3	level5						other
0.531_231.9157	1.6009	0.2197			231.9157	0.531								0	level5						other
0.538_178.974	0.7812	0.0119		C2 H2 N3 O5 P	178.974	0.538								1	level5						down
0.545_109.0015	1.3982	0.1738			109.0015	0.545								20	level5						other
0.565_294.1427	3.4365	0.08		C13 H27 O3 P S	294.1427	0.565								8	level5						other
0.568_308.1583	2.6622	0.0311	Fructoselysine	C12 H24 N2 O7	308.1583	0.568	C16488	S8015298		0	-0.0642			3	level4						up
0.596_462.0187	0.8223	0.0996		C19 H16 N2 O6 P2 S	462.0187	0.596								1	level5						other
0.605_330.1398	3.1086	0.0602	Oxomemazine	C18 H22 N2 O2 S	330.1398	0.605		S18281	HMDB0240230	-0.0004	-1.3373			0	level4	Organoheterocyclic compounds	Benzothiazines	Phenothiazines	Phenothiazines		other
0.622_351.8903	0.7855	0.1543		C2 H6 Cl O14 P S	351.8903	0.622								0	level5						other
0.634_221.8605	0.7721	0.2155			221.8605	0.634								3	level5						other
0.643_103.0997	0.5141	0.1813	Choline	C5 H13 N O	103.0997	0.643	C00114	MReference-886	HMDB0000097	0	0.05		90.6	0	level1	Organic nitrogen compounds	Organonitrogen compounds	Quaternary ammonium salts	Quaternary ammonium salts	map00260 Glycine, serine and threonine metabolism; map00564 Glycerophospholipid metabolism; map01100 Metabolic pathways; map02010 ABC transporters; map04725 Cholinergic synapse; map04976 Bile secretion; map05231 Choline metabolism in cancer;	other
0.652_142.024	1.1655	0.1337		C2 H2 N6 O2	142.024	0.652								5	level5						other
0.653_336.1644	4.4241	0.0238		C16 H26 N4 P2	336.1644	0.653								0	level5						up
0.658_155.0695	0.6895	0.0896	L-histidine	C6 H9 N3 O2	155.0695	0.658	C00135	MReference-473	HMDB0000177	0	0.2514	92.6	96	0	level1	Organic acids and derivatives	Carboxylic acids and derivatives	Amino acids, peptides, and analogues	Amino acids	map00340 Histidine metabolism; map00410 beta-Alanine metabolism; map00970 Aminoacyl-tRNA biosynthesis; map01100 Metabolic pathways; map01230 Biosynthesis of amino acids; map02010 ABC transporters; map04974 Protein digestion and absorption; map05230 Central carbon metabolism in cancer;	other
0.659_262.0774	2.2577	0.0254		C7 H15 N6 O P S	262.0774	0.659								1	level5						up
0.667_180.0264	0.4566	0.1645		C5 H8 O7	180.0264	0.667								0	level5						other
0.667_433.842	0.3157	0.2047		C2 H N2 O18 P3	433.842	0.667								3	level5						other
0.668_158.044	0.4614	0.195	Allantoin	C4 H6 N4 O3	158.044	0.668	C01551	S199	HMDB0000462	0.0001	0.361	0.6		0	level4	Organoheterocyclic compounds	Azoles	Imidazoles	Imidazoles	NULL	other

0.669_308.1218	2.3186	0.2835		C11 H20 N2 O8	308.1218	0.669								0	level5						other
0.674_175.0333	0.6775	0.1076		C3 H5 N5 O4	175.0333	0.674								3	level5						other
0.674_337.1482	2.5282	0.1172			337.1482	0.674								0	level5						other
0.679_281.111	5.5333	0.013	(2s)-3-hydroxy-2-({[(3s,4s,5r)-2,3,4-trihydroxy-5-(hydroxymethyl)tetrahydro-2-furanyl]methyl}amino)butanoic acid (non-preferred name)	C10 H19 N O8	281.111	0.679		S35014480	HMDB0037843	-0.0001	-0.4138			0	level4	Organic oxygen compounds	Organooxygen compounds	Carbohydrates and carbohydrate conjugates	Carbohydrates		up
0.688_260.1736	0.34	0.243	Carisoprodol	C12 H24 N2 O4	260.1736	0.688	C07927	S2478	HMDB0014539	0	-0.1509			0	level4	Organic acids and derivatives	Carboxylic acids and derivatives	Amino acids, peptides, and analogues	Amino acids, peptides, and analogues	NULL	other
0.689_137.0452	1.32	0.3024			137.0452	0.689								3	level5						other
0.694_266.0514	0.8235	0.2432		C5 H11 N6 O5 P	266.0514	0.694								0	level5						other
0.702_232.1788	0.1595	0.2836			232.1788	0.702								0	level5						other
0.721_253.8163	0.8346	0.1402			253.8163	0.721								0	level5						other
0.727_147.0896	0.8484	0.1767	Afegostat	C6 H13 N O3	147.0896	0.727		S394649		0.0001	0.5357			0	level4						other
0.742_101.0477	1.8018	0.1257	Acpc	C4 H7 N O2	101.0477	0.742	C01234	S520	HMDB0036458	0	0.448	91.8		0	level4	Organic acids and derivatives	Carboxylic acids and derivatives	Amino acids, peptides, and analogues	Amino acids, peptides, and analogues	map00270 Cysteine and methionine metabolism; map01100 Metabolic pathways;	other
0.773_307.8715	2.776	0.1828		C3 H3 O9 P3 S	307.8715	0.773								0	level5						other
0.774_249.9128	2.5178	0.17		C3 H7 Cl N2 O S4	249.9128	0.774								0	level5						other
0.787_256.1397	3.8336	0.1372			256.1397	0.787								1	level5						other
0.947_168.0284	0.3977	0.208	Uric acid	C5 H4 N4 O3	168.0284	0.947	C00366	MReference-753	HMDB0000289	0.0001	0.4496		91.7	0	level2	Organoheterocyclic compounds	Imidazopyrimidines	Purines and purine derivatives	Purines and derivatives	map00230 Purine metabolism; map01100 Metabolic pathways; map04976 Bile secretion;	other
1.009_179.9277	0.6541	0.1803		C4 H Cl O4 S	179.9277	1.009								0	level5						other
1.015_233.9829	0.6109	0.2189	4-hydroxy-3-(sulfooxy)benzoic acid	C7 H6 O7 S	233.9829	1.015		S35786939	HMDB0124993	-0.0005	-2.1844			15	level4	Organic acids and derivatives	Organic sulfuric acids and derivatives	Arylsulfates	Organic acids		other
1.02_176.9851	0.5235	0.1945		C3 H3 N3 O4 S	176.9851	1.02								0	level5						other
1.043_279.1318	3.2567	0.1139	(2s)-3-methyl-2-({[(3s,4s,5r)-2,3,4-trihydroxy-5-(hydroxymethyl)tetrahydro-2-furanyl]methyl}amino)butanoic acid (non-preferred name)	C11 H21 N O7	279.1318	1.043		S35014481	HMDB0037844	0	-0.0576			1	level4	Organic acids and derivatives	Carboxylic acids and derivatives	Amino acids, peptides, and analogues	Amino acids, peptides, and analogues		other
1.134_334.9702	1.2463	0.0296			334.9702	1.134								0	level5						up
1.199_311.1039	5.3358	0.0555	(2s)-4-(methylsulfanyl)-2-({[(3s,4s,5r)-2,3,4-trihydroxy-5-(hydroxymethyl)tetrahydro-2-furanyl]methyl}amino)butanoic acid	C11 H21 N O7 S	311.1039	1.199		S9035182	HMDB0037841	0	0.0734			0	level4	Organic acids and derivatives	Carboxylic acids and derivatives	Amino acids, peptides, and analogues	Amino acids, peptides, and analogues		other
1.354_343.1268	2.6762	0.2431	(2s)-3-(4-hydroxyphenyl)-2-({[(3s,4s,5r)-2,3,4-trihydroxy-5-(hydroxymethyl)tetrahydro-2-furanyl]methyl}amino)propanoic acid (non-preferred name)	C15 H21 N O8	343.1268	1.354		S35014482	HMDB0037845	0.0001	0.2474			1	level4	Organic acids and derivatives	Carboxylic acids and derivatives	Amino acids, peptides, and analogues	Amino acids, peptides, and analogues		other
1.62_195.0897	5.4091	0.1575	Metirosine	C10 H13 N O3	195.0897	1.62	C07921	S390103	HMDB0014903	0.0001	0.5331			0	level4	Phenylpropanoids and polyketides	Phenylpropanoic acids	null	Phenylpropanoic acids	NULL	other
1.63_131.0525	2.2284	0.0673	[similar to: microcystin lf; δmass: -854.4636 da]		131.0525	1.63								2	level5						other
1.717_134.0713	0.6396	0.1287			134.0713	1.717								3	level5						other
1.876_283.0917	2.4553	0.1803	8-hydroxy-deoxyguanosine	C10 H13 N5 O5	283.0917	1.876		S66049	HMDB0003333	0	0.0678			0	level4	Nucleosides, nucleotides, and analogues	Purine nucleosides	Purine 2'-deoxyribonucleosides	Purines and derivatives		other
2.4_100.0525	1.6813	0.0782	5-valerolactone	C5 H8 O2	100.0525	2.4	C02240	BGI138		0.0001	1.0833	84.9	60.6	0	level2					NULL	other
2.604_150.0542	0.5521	0.1914	Dezaguanine	C6 H6 N4 O	150.0542	2.604		S50312		0.0001	0.3503			0	level4						other
2.627_127.1179	0.4833	0.0706			127.1179	2.627								0	level5						other
3.033_311.123	0.7144	0.1651	N2-dimethylguanosine	C12 H17 N5 O5	311.123	3.033		S83878	HMDB0004824	0	0.1477			1	level4	Nucleosides, nucleotides, and analogues	Purine nucleosides	null	Purines and derivatives		other
3.139_267.1008	2.4276	0.111	Prinomide	C15 H13 N3 O2	267.1008	3.139	C10958	S54416		0.0001	0.2233		36	4	level4					NULL	other
3.207_209.2019	0.1601	0.2021			209.2019	3.207								0	level5						other

3.208_225.1162	0.7436	0.1214	Navenone a	C15 H15 N O	225.1162	3.208	C17019	S9119354		0.0008	3.5897			2	level4					NULL	other
3.21_120.0768	0.7436	0.1805	[similar to: 2-hydroxyhippuric acid; δ mass: -74.9763 da]		120.0768	3.21								1	level5						other
3.21_147.0988	0.7869	0.1529			147.0988	3.21								0	level5						other
3.245_186.9925	2.0476	0.0978	[similar to: dl-tryptophan; δ mass: -17.0974 da]		186.9925	3.245								5	level5						other
3.318_336.1323	2.2041	0.1803		C17 H16 N6 O2	336.1323	3.318								6	level5						other
3.323_217.9879	1.6446	0.1553	Sulfosalicylic acid	C7 H6 O6 S	217.9879	3.323	C16199	S7046	HMDB0011725	-0.0006	-2.8301			0	level4	Benzenoids	Benzene and substituted derivatives	Benzenesulfonic acids and derivatives	Benzene and derivatives		other
3.438_290.9831	0.193	0.1692		C8 H11 N3 O P2 S2	290.9831	3.438								1	level5						other
3.438_334.0954	0.1081	0.1832		C20 H10 N6	334.0954	3.438								9	level5						other
3.44_190.0505	0.3361	0.1855			190.0505	3.44								0	level5						other
3.44_221.0689	0.2063	0.184	Methyl 2,3-dihydro-3-hydroxy-2-oxo-1h-indole-3-acetate	C11 H11 N O4	221.0689	3.44		S35014708	HMDB0038991	0.0001	0.5702			0	level4	Organoheterocyclic compounds	Indoles and derivatives	Indolyl carboxylic acids and derivatives	Indole and derivatives		other
3.459_200.1316	3.0251	0.181	Medetomidine	C13 H16 N2	200.1316	3.459		S61868		0.0002	1.1665			2	level4						other
3.468_238.0427	2.1052	0.4945		C8 H15 O4 P S	238.0427	3.468								0	level5						other
3.469_288.0822	0.8138	0.1107		C10 H19 N4 P3	288.0822	3.469								0	level5						other
3.477_102.0318	0.7955	0.1436	Acetoacetate	C4 H6 O3	102.0318	3.477	C00164	BGI142	HMDB0000060	0.0001	1.0748	20.5		0	level3	Organic acids	Carboxylic acids [Fig]	3-Oxocarboxylic acids	Organic acids	map00072 Synthesis and degradation of ketone bodies; map00280 Valine, leucine and isoleucine degradation; map00310 Lysine degradation; map00350 Tyrosine metabolism; map00640 Propanoate metabolism; map00650 Butanoate metabolism; map01100 Metabolic pathways;	other
3.477_252.121	0.8139	0.1637	His-pro	C11 H16 N4 O3	252.121	3.477		S10608298		-0.0012	-4.9196			1	level4						other
3.494_244.1496	1.134	0.0413		C13 H24 O2 S	244.1496	3.494								11	level5						other
3.511_130.0566	1.9276	0.26		C5 H10 N2 S	130.0566	3.511								2	level5						other
3.811_341.2046	0.8155	0.1595		C14 H31 N O8	341.2046	3.811								0	level5						other
3.822_250.1414	0.8512	0.1963		C11 H22 O6	250.1414	3.822								2	level5						other
3.822_296.1468	0.8536	0.211		C12 H24 O8	296.1468	3.822								3	level5						other
3.863_277.1063	2.2087	0.2326		C13 H15 N3 O4	277.1063	3.863								1	level5						other
4.008_132.0722	1.7172	0.1308	N,n'-diethylthiourea	C5 H12 N2 S	132.0722	4.008	C19400	S2016737		0.0001	0.8173			2	level4					NULL	other
4.074_297.1787	1.2183	0.2067		C13 H23 N5 O3	297.1787	4.074								11	level5						other
4.107_228.0933	2.5562	0.1533	Chrysene	C18 H12	228.0933	4.107	C14222	S8817		-0.0006	-2.6582			1	level4					NULL	other
4.119_326.194	1.0762	0.2752	Heptaethylene glycol	C14 H30 O8	326.194	4.119		S72017	HMDB0061835	0	-0.0864			0	level4	Organic oxygen compounds	Organooxygen compounds	Ethers	Ethers		other
4.199_189.1002	1.8224	0.1957	(+)-castanospermine	C8 H15 N O4	189.1002	4.199	C02256	S49177		0.0001	0.4978			2	level4	Alkaloids	Alkaloids derived from lysine	Indolizidine alkaloids	Alkaloids	NULL	other
4.295_381.2	1.8096	0.1316		C16 H31 N O9	381.2	4.295								0	level5						other
4.351_429.2572	0.8487	0.0997		C18 H39 N O10	429.2572	4.351								0	level5						other
4.365_238.1418	1.1083	0.3153		C10 H22 O6	238.1418	4.365								1	level5						other
4.402_146.9812	5.6147	0.1301		C4 H5 N O S2	146.9812	4.402								0	level5						other
4.403_192.0391	6.2761	0.1046	Unii:n506b45lbe	C6 H12 N2 O S2	192.0391	4.403		S49470	HMDB0031186	0	-0.0832			0	level4	Organoheterocyclic compounds	Azacyclic compounds	Dithiazinanes	Dithiazinanes		other
4.405_87.0144	5.5895	0.1638		C3 H5 N S	87.0144	4.405								0	level5						other
4.444_313.21	1.2162	0.1763		C14 H27 N5 O3	313.21	4.444								14	level5						other
4.575_414.2466	1.0832	0.1575		C18 H38 O10	414.2466	4.575								0	level5						other
4.793_320.1656	1.2608	0.0975	3195	C18 H25 Cl N2 O	320.1656	4.793		S64525		0	0.0164			2	level4						other
4.797_373.2133	1.2474	0.0499		C16 H31 N5 O3 S	373.2133	4.797								7	level5						up
4.809_131.0406	2.6843	0.0672		C5 H9 N O S	131.0406	4.809								0	level5						other
4.939_166.0631	0.431	0.1969	Ethylparaben	C9 H10 O3	166.0631	4.939		S13846749	HMDB0032573	0.0001	0.7973			2	level4	Benzenoids	Benzene and substituted derivatives	Benzoic acids and derivatives	Benzene and derivatives		other
4.982_417.2395	1.2149	0.1248		C18 H35 N5 O4 S	417.2395	4.982								0	level5						other
5.039_276.0634	0.3142	0.1678	Ywa1	C14 H12 O6	276.0634	5.039		S35013649	HMDB0033649	0	-0.0121			7	level4	Organoheterocyclic compounds	Naphthopyrans	Naphthopyranones	Naphthopyranones		other
5.156_408.2179	1.3284	0.0542		C20 H32 N4 O3 S	408.2179	5.156								1	level5						other
5.213_310.6785	0.8394	0.1812	[similar to: (+/-)5(6)-dihet; δmass: -27.5672 da]		310.6785	5.213								1	level5						other
5.214_242.1442	1.3112	0.087		C13 H23 O2 P	242.1442	5.214								5	level5						other
5.241_489.315	1.1833	0.2122		C22 H43 N5 O7	489.315	5.241								5	level5						other
5.339_336.1618	1.0434	0.4374		C15 H30 O4 P2	336.1618	5.339								0	level5						other
5.34_458.2731	1.1283	0.1238		C21 H38 N4 O7	458.2731	5.34								4	level5						other
5.34_502.2994	1.2101	0.0944		C23 H42 N4 O8	502.2994	5.34								0	level5						other
5.433_347.137	0.3659	0.1964	Nafamostat	C19 H17 N5 O2	347.137	5.433		S4260		-0.0012	-3.5485			1	level4						other

5.433_352.0921	0.3681	0.1763		C17 H23 O2 P3	352.0921	5.433								1	level5						other
5.442_496.2707	1.2538	0.1147	Fexaramine	C32 H36 N2 O3	496.2707	5.442	C15649	S4484057		-0.0019	-3.8584			1	level4					NULL	other
5.448_346.693	0.8969	0.2203	[similar to: (+/-)5(6)-dihet; δmass: 8.4473 da]		346.693	5.448								0	level5						other
5.507_360.2171	2.7125	0.1398			360.2171	5.507								3	level5						other
5.514_302.1706	1.2069	0.1258	Arg-gln	C11 H22 N6 O4	302.1706	5.514		S8033257		0.0004	1.2834			0	level4						other
5.623_212.1526	2.4953	0.2147	Brivaracetam	C11 H20 N2 O2	212.1526	5.623		S8012964		0.0002	0.8055			2	level4						other
5.65_402.2012	1.1398	0.1904		C16 H35 O9 P	402.2012	5.65								0	level5						other
5.709_324.2148	1.1489	0.2254		C15 H32 O7	324.2148	5.709								0	level5						other
5.736_432.2006	1.1419	0.2084	(3r,4s,5s,6s)-6-carboxy-n-[2-(diphenylmethoxy)ethyl]-3,4,5-trihydroxy-n,n-dimethyltetrahydro-2h-pyran-2-aminium (non-preferred name)	C23 H30 N O7	432.2006	5.736		S35031959	HMDB0060897	-0.0017	-3.8303			3	level4	Organic acids and derivatives	Carboxylic acids and derivatives	Amino acids, peptides, and analogues	Amino acids, peptides, and analogues		other
6.093_523.8204	1.1668	0.2323		C13 H3 O13 P3 S2	523.8204	6.093								0	level5						other
6.125_456.2936	1.1528	0.2724		C21 H44 O10	456.2936	6.125								0	level5						other
6.139_459.277	1.34	0.043		C18 H41 N3 O10	459.277	6.139								0	level5						up
6.151_545.8338	1.1658	0.2899	[similar to: (+/-)5(6)-dihet; δmass: 207.5881 da]		545.8338	6.151								0	level5						other
6.208_567.8468	1.1307	0.1694		C15 H7 O14 P3 S2	567.8468	6.208								0	level5						other
6.228_500.32	1.1311	0.2463		C23 H48 O11	500.32	6.228								0	level5						other
6.323_544.3461	1.1757	0.2251		C25 H52 O12	544.3461	6.323								0	level5						other
6.809_162.1046	0.7651	0.0965	Valerophenone	C11 H14 O	162.1046	6.809		MReference-935	HMDB0031208	0.0001	0.7367		53.9	0	level3	Organic oxygen compounds	Organooxygen compounds	Carbonyl compounds	Carbonyl compounds		other
7.076_231.1294	0.813	0.2123		C11 H21 N O2 S	231.1294	7.076								0	level5						other
7.22_252.1338	1.977	0.2307		C10 H16 N6 O2	252.1338	7.22								0	level5						other
7.457_317.2933	0.6646	0.3618	2-amino-1,3,4-octadecanetriol	C18 H39 N O3	317.2933	7.457	C12144	MReference-545	HMDB0004610	0.0003	0.8681		63.1	0	level2	Organic nitrogen compounds	Organonitrogen compounds	Amines	Amines	map00600 Sphingolipid metabolism; map01100 Metabolic pathways;	other
7.544_289.2617	0.5306	0.2105		C16 H35 N O3	289.2617	7.544								0	level5						other
7.676_166.0636	0.7495	0.1515	Apocynin	C9 H10 O3	166.0636	7.676	C11380	MReference-5847		0.0006	3.4097		65.5	0	level2	PK Polyketides	PK12 Flavonoids	PK1202 Flavans, Flavanols and Leucoanthocyanidins	Polyketides[PK]	NULL	other
7.676_194.095	0.7719	0.1916	Butylparaben	C11 H14 O3	194.095	7.676		S6916	HMDB0032575	0.0007	3.516			1	level4	Benzenoids	Benzene and substituted derivatives	Benzoic acids and derivatives	Benzene and derivatives		other
7.697_455.3252	0.6604	0.0909			455.3252	7.697								0	level5						other
7.815_131.9521	1.8353	0.3657			131.9521	7.815								0	level5						other
7.831_459.2625	0.0204	0.1981	Militarinone a	C26 H37 N O6	459.2625	7.831	C12333	S26384876		0.0004	0.8359			0	level4					NULL	other
7.832_303.2776	0.6016	0.1775		C17 H37 N O3	303.2776	7.832								2	level5						other
7.923_465.3092	0.6671	0.1797	Glycocholate	C26 H43 N O6	465.3092	7.923	C01921	BGI295	HMDB0000138	0.0002	0.4008	57.7		0	level3	Steroids	24-Carbon atoms	Cholane derivatives [Fig]	Sterol lipids[ST]	map00120 Primary bile acid biosynthesis; map01100 Metabolic pathways; map04976 Bile secretion; map04979 Cholesterol metabolism;	other
7.924_429.288	0.6211	0.1551	Adaprolol	C26 H39 N O4	429.288	7.924		S54735		0.0001	0.189			2	level4						other
7.936_272.1988	0.3459	0.0937	Pentadecanedioic acid	C15 H28 O4	272.1988	7.936		S141102		0.0001	0.3012			6	level4						other
7.937_254.1883	0.3204	0.0892	Prohydrojasmon	C15 H26 O3	254.1883	7.937	C18538	S16738647		0.0001	0.3403			0	level4					NULL	other
8.077_316.2315	0.8049	0.2191			316.2315	8.077								0	level5						other
8.143_246.1621	0.2896	0.2449	Geroquinol	C16 H22 O2	246.1621	8.143	C10793	S4445151		0.0001	0.5955			2	level4					map00130 Ubiquinone and other terpenoid-quinone biosynthesis;	other
8.165_472.319	0.0432	0.1788		C29 H44 O5	472.319	8.165								0	level5						other
8.251_174.1411	0.2112	0.1881	Ionene	C13 H18	174.1411	8.251		S61372	HMDB0059826	0.0002	1.2201			0	level4	Benzenoids	Tetralins	null	Benzene and derivatives		other
8.251_535.3278	0.1042	0.2293		C26 H50 N O8 P	535.3278	8.251								4	level5						other
8.282_435.3349	0.1796	0.1835		C27 H41 N5	435.3349	8.282								0	level5						other
8.326_388.2976	0.1541	0.2136	Dromostanolone tetrahydropyranyl ether	C25 H40 O3	388.2976	8.326	C15422	S224218		-0.0001	-0.3103			2	level4					NULL	other
8.385_190.0784	0.8854	0.1813	(5e)-7,7-dimethyl-4-oxa-8-thia-2,5-diazanon-5-en-3-one	C7 H14 N2 O2 S	190.0784	8.385	C11015	S7844539		0.0008	4.2795			0	level4					NULL	other
8.59_399.3349	0.3147	0.2244	Palmitoylcarnitine	C23 H45 N O4	399.3349	8.59		MReference-2936		0.0001	0.1463	90.5	91.6	0	level2						other
8.595_435.3349	0.2023	0.2837		C26 H45 N O4	435.3349	8.595								6	level5						other
8.687_251.1522	0.8487	0.036	Lt9970000	C14 H21 N O3	251.1522	8.687	C18912	S39512		0.0001	0.3139			0	level4					NULL	other
8.761_640.8392	1.2846	0.258	Tyropanoic acid	C15 H18 I3 N O3	640.8392	8.761		S5409		-0.0029	-4.5462			12	level4						other

8.778_438.3133	0.2941	0.1954	(1r,3s,5z)-5-{(2e)-2-[(1z,3as,7as)-1-(6-ethyl-6-hydroxy-3-octyn-2-ylidene)-7a-methyloctahydro-4h-inden-4-ylidene]ethylidene}-4-methylene-1,3-cyclohexanediol	C29 H42 O3	438.3133	8.778		S7826440		-0.0001	-0.168			3	level4						other
8.781_456.324	0.266	0.1781	Callystatin a	C29 H44 O4	456.324	8.781	C16891	S4581330		0.0001	0.1702			5	level4					NULL	other
8.841_356.2715	0.2705	0.1749	Ethyl docosahexaenoate	C24 H36 O2	356.2715	8.841	C16185	S8007148		0	0.0263		30.4	0	level4	FA Fatty acyls	FA04 Docosanoids	Null	Fatty acyls[FA]	NULL	other
8.841_409.3193	0.3112	0.1887		C24 H43 N O4	409.3193	8.841						78.2		0	level5						other
8.841_437.3506	0.2563	0.1435		C27 H43 N5	437.3506	8.841								0	level5						other
8.841_806.5678	0.1412	0.177		C46 H74 N6 O6	806.5678	8.841								5	level5						other
8.842_214.1722	0.2537	0.1739		C16 H22	214.1722	8.842								4	level5						other
8.842_228.1879	0.2461	0.2327		C17 H24	228.1879	8.842								0	level5						other
8.842_338.261	0.2586	0.2026	3-(2,4-cyclopentadien-1-ylidene)-5alpha-androstan-17beta-ol	C24 H34 O	338.261	8.842	C14915	S10128377		0.0001	0.2019			0	level4					NULL	other
8.842_414.2746	0.3586	0.1802		C22 H34 N6 O2	414.2746	8.842								0	level5						other
8.842_784.5858	0.1201	0.2061	Deoxycholate	C24 H40 O4	784.5858	8.842	C04483	BGI584	HMDB0000626	392.2932	1000001.297	65.4		0	level2	Steroids	24-Carbon atoms	Cholane derivatives [Fig]	Sterol lipids[ST]	map04976 Bile secretion;	other
8.843_242.2034	0.286	0.1682		C18 H26	242.2034	8.843								3	level5						other
8.843_392.2927	0.2767	0.1964	Ursodeoxycholate	C24 H40 O4	392.2927	8.843	C07880	BGI614	HMDB0000946	0	0.1166	76.8		0	level3	Steroids	24-Carbon atoms	Cholane derivatives [Fig]	Sterol lipids[ST]		other
8.849_430.2381	0.3506	0.1871		C14 H34 N6 O9	430.2381	8.849							46.7	0	level5						other
9.019_344.2327	0.0799	0.1633	Lamtidine	C18 H28 N6 O	344.2327	9.019	C11804	S64990		0.0002	0.6497		45.8	6	level4					NULL	other
9.105_322.2484	0.6577	0.2397		C16 H30 N6 O	322.2484	9.105								3	level5						other
9.165_239.0923	0.6615	0.2289		C8 H18 N O5 P	239.0923	9.165								0	level5						other
9.448_467.3378	0.5473	0.1713		C23 H50 N O6 P	467.3378	9.448								0	level5						other
9.603_467.3014	0.7317	0.0828	Buprenorphine	C29 H41 N O4	467.3014	9.603	C08007	S559124	HMDB0015057	-0.0022	-4.7074			0	level4	Benzenoids	Phenanthrenes and derivatives	null	Benzene and derivatives	NULL	other
9.66_328.2395	0.8706	0.2643	Retinyl acetate	C22 H32 O2	328.2395	9.66		S553599		-0.0008	-2.3313			0	level4						other
9.685_304.2399	0.6315	0.0679	Arachidonic acid	C20 H32 O2	304.2399	9.685	C00219	MReference-2742	HMDB0001043	-0.0003	-0.9957		75.2	3	level2	Lipids and lipid-like molecules	Fatty Acyls	Fatty acids and conjugates	Fatty acyls[FA]	map00590 Arachidonic acid metabolism; map00591 Linoleic acid metabolism; map01040 Biosynthesis of unsaturated fatty acids; map01100 Metabolic pathways; map04216 Ferroptosis; map04217 Necroptosis; map04270 Vascular smooth muscle contraction; map04611 Platelet activation; map04664 Fc epsilon RI signaling pathway; map04666 Fc [illegible]	other
9.714_336.1975	0.7626	0.1938		C18 H29 N2 O2 P	336.1975	9.714							46.3	9	level5						other
9.715_318.1868	0.7672	0.2026		C16 H32 O2 P2	318.1868	9.715							50.2	2	level5						other
9.822_347.2822	0.5648	0.1014	Anandamide	C22 H37 N O2	347.2822	9.822	C11695	S4445241	HMDB0004080	-0.0003	-0.7312			1	level4	Organic nitrogen compounds	Organonitrogen compounds	Amines	Amines	map04080 Neuroactive ligand-receptor interaction; map04714 Thermogenesis; map04723 Retrograde endocannabinoid signaling; map04750 Inflammatory mediator regulation of TRP channels;	other
9.822_352.2375	0.5313	0.0807		C20 H28 N6	352.2375	9.822								33	level5						other
9.922_349.2978	0.578	0.0995	N-homo-?-linolenoylethanolamine	C22 H39 N O2	349.2978	9.922	C13828	S4445443	HMDB0013625	-0.0003	-0.7781			15	level4	Organic nitrogen compounds	Organonitrogen compounds	Amines	Amines	NULL	other
9.924_320.2026	0.5539	0.2297		C17 H28 N4 S	320.2026	9.924								0	level5						other
9.924_326.2196	0.4654	0.235		C17 H30 N2 O4	326.2196	9.924								8	level5						other
0.543_273.1325	2.5613	0.2687		C11 H19 N3 O5	273.1325	0.543								0	level5						other
0.544_127.0121	1.2719	0.253	[similar to: n-acetyl-l-cysteine; δ mass: -36.0182 da]		127.0121	0.544								3	level5						other
0.544_129.0096	1.2592	0.3184	3-amino-6-chloropyridazine	C4 H4 Cl N3	129.0096	0.544		S20342		0.0002	1.5876			2	level4						other
0.581_543.887	1.2914	0.236		C20 H5 N2 O9 P S3	543.887	0.581								2	level5						other
0.581_815.8367	1.3155	0.2062		C24 H10 N4 O17 P2 S4	815.8367	0.581								0	level5						other
0.596_709.8437	0.5792	0.1815			709.8437	0.596								0	level5						other
0.598_166.0606	0.7783	0.2158		C5 H6 N6 O	166.0606	0.598								3	level5						other

0.633_177.0514	0.8959	0.2026			177.0514	0.633								0	level5						other
0.641_234.9131	0.5132	0.2329		C2 H6 Cl N3 S4	234.9131	0.641								0	level5						other
0.647_168.0511	0.8857	0.1582	[similar to: mjn110; δ mass: -293.0398 da]		168.0511	0.647								1	level5						other
0.648_218.0192	0.8007	0.3064		C4 H11 O8 P	218.0192	0.648								0	level5						other
0.651_337.0433	0.5521	0.0589	Famotidine	C8 H15 N7 O2 S3	337.0433	0.651		S3208	HMDB0001919	-0.0016	-4.877			4	level4	Organoheterocyclic compounds	Azoles	Thiazoles	Thiazoles		other
0.657_169.0353	0.3977	0.2197			169.0353	0.657								0	level5						other
0.671_181.0409	0.3165	0.2426	Acamprosate	C5 H11 N O4 S	181.0409	0.671		S64300	HMDB0014797	0	0.0321			0	level4	Organic acids and derivatives	Organic sulfonic acids and derivatives	Organosulfonic acids and derivatives	Organic acids		other
0.684_113.059	1.2422	0.3131	Creatinine	C4 H7 N3 O	113.059	0.684	C00791	MReference - 13	HMDB0000562	0.0001	0.6359		95.2	0	level1	Organic acids and derivatives	Carboxylic acids and derivatives	Amino acids, peptides, and analogues	Amino acids, peptides, and analogues	map00330 Arginine and proline metabolism; map01100 Metabolic pathways;	other
0.687_274.9443	1.439	0.3202		C5 H10 Cl N3 S4	274.9443	0.687								0	level5						other
0.691_201.1115	1.7539	0.3947	5-(n,n-dimethylcarbamimidamido)-2-oxopentanoic acid	C8 H15 N3 O3	201.1115	0.691		S58779677	HMDB0240212	0.0001	0.7025			0	level4	Organic acids and derivatives	Keto acids and derivatives	Short-chain keto acids and derivatives	Organic acids		other
0.695_143.0495	0.1671	0.1775	[similar to: dl-stachydrine; δmass: -0.0452 da]		143.0495	0.695								3	level5						other
0.704_203.1157	1.4718	0.2659	O-acetylcarnitine	C9 H17 N O4	203.1157	0.704	C02571	BGI603		0	-0.2106	95.8	92.6	0	level1					map04931 Insulin resistance;	other
0.721_228.1474	1.2999	0.2428	Prolylleucine	C11 H20 N2 O3	228.1474	0.721		MReference - 559		0	-0.0991		76.2	0	level2						other
0.721_270.9577	0.3624	0.1682		C2 H6 Cl N O12	270.9577	0.721								3	level5						other
0.721_779.4396	0.8961	0.5867			779.4396	0.721								0	level5						other
0.722_783.4335	0.8203	0.3621			783.4335	0.722								3	level5						other
0.725_132.0535	1.3604	0.2065	L-asparagine	C4 H8 N2 O3	132.0535	0.725	C00152	BGI344	HMDB0000168	0	0.1146	7.8		0	level3	Peptides	Amino acids	Common amino acids [Fig]	Amino acids	map00250 Alanine, aspartate and glutamate metabolism; map00970 Aminoacyl-tRNA biosynthesis; map01100 Metabolic pathways; map01230 Biosynthesis of amino acids; map04974 Protein digestion and absorption; map04978 Mineral absorption; map05230 Central carbon metabolism in cancer;	other
0.732_260.0037	1.2421	0.3712		C7 H14 Cl O4 P S	260.0037	0.732								0	level5						other
0.737_240.1474	0.8789	0.2938	3477	C12 H20 N2 O3	240.1474	0.737	C07807	S4679	HMDB0015407	0	-0.0088			0	level4	Organoheterocyclic compounds	Pyridines and derivatives	Hydroxypyridines	Pyridine and derivatives	NULL	other
0.743_78.014	0.754	0.2269	Dimethyl sulfoxide	C2 H6 O S	78.014	0.743	C11143	S659	HMDB0002151	0.0001	1.2129			0	level4	Organosulfur compounds	Sulfoxides	null	Sulfoxides	map00920 Sulfur metabolism; map01100 Metabolic pathways;	other
0.762_180.0026	0.3766	0.2001		C3 H4 N2 O7	180.0026	0.762								0	level5						other
0.77_193.0152	1.8454	0.2482		C4 H7 N3 O4 S	193.0152	0.77								4	level5						other
0.775_171.0332	1.606	0.2861			171.0332	0.775								3	level5						other
0.789_234.1577	3.4943	0.1744		C10 H22 N2 O4	234.1577	0.789								2	level5						other
0.791_282.1556	0.2903	0.3242		C8 H23 N6 O3 P	282.1556	0.791								0	level5						other
0.914_123.0321	2.3188	0.281	Nicotinic acid	C6 H5 N O2	123.0321	0.914	C00253	MReference - 519	HMDB0001488	0.0001	0.8192		91	0	level2	Organoheterocyclic compounds	Pyridines and derivatives	Pyridinecarboxylic acids and derivatives	Pyridine and derivatives	map00760 Nicotinate and nicotinamide metabolism; map01100 Metabolic pathways; map05133 Pertussis;	other
1.224_113.0478	1.2165	0.4988	Ma4000000	C5 H7 N O2	113.0478	1.224	C07275	S63891		0.0001	1.1399	63		0	level4					NULL	other
1.261_118.042	1.1549	0.5245	Coumarone	C8 H6 O	118.042	1.261	C14512	S8868	HMDB0032929	0.0001	0.9477			1	level4	Organoheterocyclic compounds	Benzofurans	null	Benzofurans	NULL	other
1.27_125.0591	0.6909	0.2976	5-methylcytosine	C5 H7 N3 O	125.0591	1.27	C02376	MReference - 263	HMDB0002894	0.0002	1.2478		67.8	4	level1	Organoheterocyclic compounds	Diazines	Pyrimidines and pyrimidine derivatives	Pyrimidines and pyrimidine derivatives	map00240 Pyrimidine metabolism; map01100 Metabolic pathways;	other
1.279_135.0685	1.1492	0.5695	N-benzylformamide	C8 H9 N O	135.0685	1.279	C15561	MReference - 817		0.0001	0.8523	93.9	79.4	8	level2					NULL	other
1.528_134.1134	0.6962	0.3312			134.1134	1.528								2	level5						other
1.571_131.054	0.5084	0.2412			131.054	1.571								0	level5						other
11.11_743.5472	0.3735	0.3234	Dioleoylphosphatidylethanolamine	C41 H78 N O8 P	743.5472	11.11		S4743651		0.0007	0.9745			0	level4						other
11.166_702.5679	0.3381	0.2407	Palmitoyl sphingomyelin	C39 H79 N2 O6 P	702.5679	11.166		MReference - 4865		0.0003	0.4943		86.7	0	level2						other
2.29_143.0584	1.3171	0.4233	Trimethadione	C6 H9 N O3	143.0584	2.29		S5374	HMDB0014491	0.0002	1.0774	84.6		0	level4	Organoheterocyclic compounds	Azolidines	Oxazolidines	Oxazolidines		other
2.322_172.0713	0.5427	0.1421		C4 H8 N6 O2	172.0713	2.322								0	level5						other
2.381_204.0935	1.9157	0.2521	Dihydroaceanthrylene	C16 H12	204.0935	2.381	C19401	S141070		-0.0004	-1.7416			0	level4					NULL	other
2.423_152.0587	3.2659	0.2738	5-nitro-o-toluidine	C7 H8 N2 O2	152.0587	2.423	C16398	S7166		0.0001	0.6366			0	level4						other

2.546_126.043	0.6356	0.2901	Thymine	C5 H6 N2 O2	126.043	2.546	C00178	S1103	HMDB0000262	0.0001	0.8874	17.2		1	level4	Organoheterocyclic compounds	Diazines	Pyrimidines and pyrimidine derivatives	Pyrimidines and pyrimidine derivatives	map00240 Pyrimidine metabolism; map01100 Metabolic pathways;	other
2.651_198.1005	2.262	0.275	Metharbital	C9 H14 N2 O3	198.1005	2.651		S3957	HMDB0014606	0.0001	0.4454			2	level4	Organoheterocyclic compounds	Diazines	Pyrimidines and pyrimidine derivatives	Pyrimidines and pyrimidine derivatives		other
2.83_231.1471	0.5383	0.081	N-(tert-butoxycarbonyl)-l-leucine	C11 H21 N O4	231.1471	2.83	C04301	S75037		0	0.1367			0	level4					NULL	other
2.931_661.2932	0.7383	0.2243		C24 H48 N5 O14 P	661.2932	2.931								9	level5						other
2.972_191.1159	1.7862	0.1371		C8 H17 N O4	191.1159	2.972								17	level5						other
2.997_179.0809	0.8524	0.4031	7-aminomethyl-7-deazaguanine	C7 H9 N5 O	179.0809	2.997	C16675	S166	HMDB0011690	0.0002	0.9131			4	level4	Organoheterocyclic compounds	Pyrrolopyrimidines	Pyrrolo[2,3-d]pyrimidines	Pyrrolo[2,3-d]pyrimidines	map00790 Folate biosynthesis; map01100 Metabolic pathways;	other
3.033_253.1526	0.8963	0.3652		C10 H23 N O6	253.1526	3.033								1	level5						other
3.046_239.1733	0.7445	0.1124	[similar to: (+/-)5(6)-dihet; δmass: -99.0724 da]		239.1733	3.046								8	level5						other
3.088_120.0212	1.9358	0.3119		C7 H4 O2	120.0212	3.088						30.4		3	level5						other
3.089_195.0534	2.0326	0.3007	2-hydroxyhippuric acid	C9 H9 N O4	195.0534	3.089	C07588	MReference-529	HMDB0000840	0.0002	1.0542		90.1	19	level2	Benzenoids	Benzene and substituted derivatives	Benzoic acids and derivatives	Benzene and derivatives	NULL	other
3.128_160.0672	1.1263	0.4995		C6 H12 N2 O S	160.0672	3.128								2	level5						other
3.179_276.1587	1.9647	0.3965	P-coumaroylagmatine	C14 H20 N4 O2	276.1587	3.179	C04498	S4444279	HMDB0033460	0	0.1436			2	level4	Phenylpropanoids and polyketides	Cinnamic acids and derivatives	Hydroxycinnamic acids and derivatives	Polyketides[PK]	map00330 Arginine and proline metabolism; map01100 Metabolic pathways;	other
3.182_447.2636	0.6318	0.0888		C20 H42 N5 P S2	447.2636	3.182								3	level5						other
3.284_194.1056	1.6672	0.2358	6-hydroxypseudooxynicotine	C10 H14 N2 O2	194.1056	3.284	C01297	S388576	HMDB0240264	0.0001	0.4667			0	level4	Organic oxygen compounds	Organooxygen compounds	Carbonyl compounds	Carbonyl compounds	map00760 Nicotinate and nicotinamide metabolism; map01100 Metabolic pathways;	other
3.448_130.9865	1.7071	0.1421		C4 H5 N S2	130.9865	3.448								7	level5						other
3.448_176.0444	1.6066	0.1559		C6 H12 N2 S2	176.0444	3.448								0	level5						other
3.488_150.0894	1.0703	0.1875		C6 H14 O4	150.0894	3.488								0	level5						other
3.616_113.0842	0.876	0.1594	Caprolactam	C6 H11 N O	113.0842	3.616	C06593	MReference-2867	HMDB0062769	0.0001	1.2649		73.6	0	level2	Organoheterocyclic compounds	Lactams	Caprolactams	Caprolactams	map01100 Metabolic pathways;	other
3.688_250.1318	2.6453	0.1957	(+)-lacosamide	C13 H18 N2 O3	250.1318	3.688		S189902		0	0.0107			0	level4						other
3.741_412.1344	0.7527	0.1479	N6-threonylcarbamoyladenosine	C15 H20 N6 O8	412.1344	3.741		S141829		0.0001	0.239			7	level4						other
3.791_253.0951	1.5754	0.2451	N-acetylvanilalanine	C12 H15 N O5	253.0951	3.791		S15185955	HMDB0011716	0	0.1959			0	level4	Organic acids and derivatives	Carboxylic acids and derivatives	Amino acids, peptides, and analogues	Amino acids, peptides, and analogues		other
3.836_288.1757	1.1603	0.2641		C15 H28 O3 S	288.1757	3.836								0	level5						other
3.908_196.1213	1.8188	0.2782	Fasoracetam	C10 H16 N2 O2	196.1213	3.908	C13311	S171980		0.0001	0.6893			1	level4					NULL	other
3.91_179.0948	1.8731	0.2541	Salsolinol	C10 H13 N O2	179.0948	3.91	C09642	BGI510	HMDB0042012	0.0001	0.7429	7.5		1	level3	Alkaloids	Alkaloids derived from tyrosine	Isoquinoline alkaloids	Alkaloids		other
3.921_264.111	0.3132	0.3174	N-phenylacetylglutamine	C13 H16 N2 O4	264.111	3.921	C04148	MReference-541	HMDB0006344	0	0.125		95.4	2	level2	Organic acids and derivatives	Carboxylic acids and derivatives	Amino acids, peptides, and analogues	Amino acids, peptides, and analogues	map00360 Phenylalanine metabolism;	other
3.983_144.0359	2.0539	0.2421		C5 H8 N2 O S	144.0359	3.983								0	level5						other
4.002_136.0525	1.7922	0.1907	2145	C8 H8 O2	136.0525	4.002	C05613	S7422	HMDB0041485	0.0001	0.7966			0	level4	Benzenoids	Benzene and substituted derivatives	Benzyloxycarbonyls	Benzene and derivatives	NULL	other
4.007_522.2332	2.1782	0.3178		C23 H35 N6 O6 P	522.2332	4.007								1	level5						other
4.008_87.0144	1.6494	0.1438		C3 H5 N S	87.0144	4.008								1	level5						other
4.012_148.0672	1.4071	0.291		C5 H12 N2 O S	148.0672	4.012								0	level5						other
4.013_162.0828	1.4924	0.2994		C6 H14 N2 O S	162.0828	4.013								0	level5						other
4.048_337.1737	1.6067	0.1782	Kyotorphin	C15 H23 N5 O4	337.1737	4.048		S2829	HMDB0029099	-0.0013	-3.8395			0	level4	Organic acids and derivatives	Carboxylic acids and derivatives	Amino acids, peptides, and analogues	Amino acids, peptides, and analogues		other
4.107_340.1732	0.8827	0.3279		C14 H28 O9	340.1732	4.107								7	level5						other
4.118_238.1418	1.1076	0.2981		C10 H22 O6	238.1418	4.118								5	level5						other
4.119_190.0568	1.0654	0.2031	[similar to: neopterin; δ mass: -63.0243 da]		190.0568	4.119								5	level5						other
4.154_252.1574	1.3309	0.1396	Hexazinone	C12 H20 N4 O2	252.1574	4.154	C10926	S36542		-0.0013	-4.9674			3	level4					NULL	other
4.161_292.9987	0.392	0.2855		C12 H7 N O6 S	292.9987	4.161								0	level5						other
4.2_143.0947	1.558	0.1928	Stachydrine	C7 H13 N O2	143.0947	4.2	C10172	S103115	HMDB0004827	0.0001	0.7348			9	level4	Organic acids and derivatives	Carboxylic acids and derivatives	Amino acids, peptides, and analogues	Amino acids, peptides, and analogues	NULL	other
4.258_184.1214	2.5404	0.2054	Apronalide	C9 H16 N2 O2	184.1214	4.258		S10264		0.0002	1.12			0	level4						other
4.279_250.1237	1.1656	0.2379	[similar to: l-kynurenine; δmass: 42.0389 da]		250.1237	4.279								8	level5						other
4.315_87.0144	3.0082	0.1514		C3 H5 N S	87.0144	4.315								0	level5						other
4.354_384.1995	0.8759	0.2308		C16 H32 O10	384.1995	4.354								0	level5						other
4.362_370.2203	1.0688	0.3799	Ho-dpeg8-oh	C16 H34 O9	370.2203	4.362		S71135	HMDB0094680	0	0.0116			2	level4	Organic oxygen compounds	Organooxygen compounds	Ethers	Ethers		other
4.365_401.2615	1.6159	0.3507		C21 H39 N O4 S	401.2615	4.365								0	level5						other
4.377_341.2048	1.2446	0.2499		C14 H31 N O8	341.2048	4.377								0	level5						other

4.568_473.2836	0.7996	0.2108		C24 H45 N O4 P2	473.2836	4.568								1	level5						other
4.57_428.2257	0.8591	0.2283		C19 H32 N4 O7	428.2257	4.57								2	level5						other
4.574_538.2299	0.6873	0.1306		C20 H44 O12 P2	538.2299	4.574								1	level5						other
4.76_472.252	0.9241	0.4615	Sulukast	C25 H36 N4 O3 S	472.252	4.76		S4940793		0.0012	2.5848			0	level4						other
4.76_517.3101	0.8776	0.2777		C22 H47 N O12	517.3101	4.76								1	level5						other
4.764_458.2729	1.0759	0.302		C20 H42 O11	458.2729	4.764								0	level5						other
4.769_210.137	2.3741	0.2171	Cyclo(leucylprolyl)	C11 H18 N2 O2	210.137	4.769		S92947		0.0002	0.7656			0	level4						other
4.842_190.0995	1.9531	0.3335	Butylphthalide	C12 H14 O2	190.0995	4.842	C17854	S55293	HMDB0032064	0.0002	0.8545			0	level4	Organoheterocyclic compounds	Benzofurans	Benzofuranones	Benzofuranones	NULL	other
4.9_401.2625	1.2436	0.1981		C18 H35 N5 O5	401.2625	4.9								0	level5						other
4.928_516.2783	0.8768	0.2		C23 H40 N4 O9	516.2783	4.928								0	level5						other
4.977_353.2048	1.4423	0.1468		C15 H31 N O8	353.2048	4.977								11	level5						other
5.026_281.1165	0.6203	0.2736		C16 H15 N3 O2	281.1165	5.026								20	level5						other
5.062_203.1159	1.4272	0.3809	Acetylcarnitine	C9 H17 N O4	203.1159	5.062		S21243783		0.0002	0.753			0	level4						other
5.081_591.3831	1.1079	0.2792		C27 H53 N5 O9	591.3831	5.081								0	level5						other
5.082_414.2468	1.1199	0.3517		C18 H38 O10	414.2468	5.082								1	level5						other
5.156_616.2272	0.8565	0.2069		C28 H39 N6 O4 P3	616.2272	5.156								0	level5						other
5.167_191.0948	1.5542	0.3432	Nl8513000	C11 H13 N O2	191.0948	5.167		S12305	HMDB0001896	0.0001	0.7617			0	level4	Organoheterocyclic compounds	Indoles and derivatives	Indoles	Indoles		other
5.181_253.0163	0.2952	0.29	Furathiazole	C9 H7 N3 O4 S	253.0163	5.181	C19273	S10299		0.0006	2.4412			4	level4					NULL	other
5.305_452.2442	1.3146	0.0843	Diphenoxylate	C30 H32 N2 O2	452.2442	5.305	C07872	S12919	HMDB0015213	-0.0021	-4.7514			0	level4	Benzenoids	Benzene and substituted derivatives	Diphenylacetonitriles	Benzene and derivatives	NULL	other
5.335_332.6917	0.8695	0.242	[similar to: (+/-)5(6)-dihet; δmass: -5.5540 da]		332.6917	5.335								0	level5						other
5.435_312.0998	0.4184	0.2103	4',5,7-trimethoxyflavone	C18 H16 O5	312.0998	5.435		S72029	HMDB0030842	0	0.0133			1	level4	Phenylpropanoids and polyketides	Flavonoids	O-methylated flavonoids	Flavonoids		other
5.438_224.1161	0.614	0.1916	Butalbital	C11 H16 N2 O3	224.1161	5.438		S2387	HMDB0014386	0	0.1797			4	level4	Organoheterocyclic compounds	Diazines	Pyrimidines and pyrimidine derivatives	Pyrimidines and pyrimidine derivatives		other
5.452_458.2731	1.1591	0.2608		C24 H44 O4 P2	458.2731	5.452								1	level5						other
5.519_297.2151	1.1756	0.1121		C14 H27 N5 O2	297.2151	5.519								0	level5						other
5.552_546.3258	1.3668	0.3149		C27 H53 N2 O3 P3	546.3258	5.552								10	level5						other
5.554_383.7438	1.1375	0.2071	[similar to: (+/-)5(6)-dihet; δmass: 45.4981 da]		383.7438	5.554								3	level5						other
5.557_593.3449	1.2317	0.1476		C36 H52 N O2 P S	593.3449	5.557								19	level5						other
5.583_529.3113	1.4309	0.2052		C26 H50 N3 O2 P3	529.3113	5.583								4	level5						other
5.648_390.218	1.1157	0.2701		C18 H34 N2 O5 S	390.218	5.648								0	level5						other
5.651_590.3517	1.3275	0.0914		C36 H50 N2 O3 S	590.3517	5.651								1	level5						other
5.664_637.371	1.2391	0.0948		C31 H61 N O6 P2 S	637.371	5.664								8	level5						other
5.692_286.6681	1.4445	0.1588			286.6681	5.692								0	level5						other
5.694_590.3636	1.5299	0.0634		C25 H54 N2 O13	590.3636	5.694								0	level5						other
5.695_573.3367	1.5361	0.0683		C25 H51 N O13	573.3367	5.695								0	level5						other
5.871_368.241	1.1158	0.3345		C17 H36 O8	368.241	5.871								0	level5						other
6.008_412.2673	1.1593	0.2867		C19 H40 O9	412.2673	6.008								23	level5						other
6.123_413.7604	1.4868	0.2341	[similar to: (+/-)5(6)-dihet; δmass: 75.5147 da]		413.7604	6.123								0	level5						other
6.642_234.1733	0.7582	0.3062	Lignocaine	C14 H22 N2 O	234.1733	6.642	C07073	S3548	HMDB0014426	0.0001	0.3241			1	level4	Benzenoids	Benzene and substituted derivatives	Xylenes	Benzene and derivatives	map00982 Drug metabolism - cytochrome P450;	other
6.646_186.1258	0.8561	0.3484	Queen bee acid	C10 H18 O3	186.1258	6.646		S4472163		0.0002	0.8627			0	level4						other
6.652_245.2355	0.6761	0.2748		C14 H31 N O2	245.2355	6.652								3	level5						other
6.981_266.1995	0.7846	0.1788		C15 H26 N2 O2	266.1995	6.981								0	level5						other
7.223_392.2177	0.5639	0.1509		C18 H28 N6 O4	392.2177	7.223								0	level5						other
7.442_273.267	0.6212	0.3627	Hexadecasphinganine	C16 H35 N O2	273.267	7.442	C13915	S571105		0.0003	0.9942			0	level4	SP Sphingolipids	SP01 Sphingoid bases	SP0104 Sphingoid base homologs and variants	Sphingolipids[SP]	NULL	other
7.528_229.2406	0.4897	0.3105		C14 H31 N O	229.2406	7.528								1	level5						other
7.585_185.2145	0.5191	0.2723		C12 H27 N	185.2145	7.585								6	level5						other
7.61_261.0789	0.8674	0.2865	Flumequine	C14 H12 F N O3	261.0789	7.61		S3257	HMDB0041895	-0.0012	-4.785			3	level4	Organoheterocyclic compounds	Quinolines and derivatives	Quinoline carboxylic acids	Quinoline carboxylic acids		other
7.658_190.1363	0.7678	0.5141	3420	C13 H18 O	190.1363	7.658		S4517997	HMDB0013804	0.0005	2.7266			0	level4	Organic oxygen compounds	Organooxygen compounds	Carbonyl compounds	Carbonyl compounds		other
7.676_94.0422	0.8017	0.2434	Benzenol	C6 H6 O	94.0422	7.676	C00146	S971	HMDB0000228	0.0004	4.0496	71.9		0	level4	Benzenoids	Phenols	1-hydroxy-4-unsubstituted benzenoids	Phenols and derivatives	map00350 Tyrosine metabolism; map01100 Metabolic pathways; map04974 Protein digestion and absorption;	other
7.813_204.0803	1.1983	0.4305	[similar to: 2,3-dinor-8-epi-prostaglandin f2α; δ mass: -122.1290 da]		204.0803	7.813								0	level5						other
7.817_135.948	1.8552	0.2887		C3 H2 Cl O2 P	135.948	7.817								0	level5						other
7.907_352.2226	0.6764	0.3186		C16 H28 N6 O3	352.2226	7.907							65.6	0	level5						other

7.935_222.1621	0.3646	0.1038	2,6-di-tert-butylhydroquinone	C14 H22 O2	222.1621	7.935		S68075	HMDB0040178	0.0001	0.6524			0	level4	Benzenoids	Benzene and substituted derivatives	Phenylpropanes	Benzene and derivatives		other
7.936_162.141	0.3356	0.1414	Hexylbenzene	C12 H18	162.141	7.936		S13487	HMDB0061815	0.0001	0.8035			0	level4	Benzenoids	Benzene and substituted derivatives	null	Benzene and derivatives		other
7.936_204.1516	0.3432	0.0952	(z)-?-amylcinnamyl alcohol	C14 H20 O	204.1516	7.936		S20122674	HMDB0036170	0.0002	1.0395			5	level4	Phenylpropanoids and polyketides	Cinnamyl alcohols	null	Cinnamyl alcohols		other
8.077_111.0673	0.8399	0.3265	[similar to: spectinomycin; δmass: -221.0911 da]		111.0673	8.077								0	level5						other
8.077_317.2345	0.8514	0.3562	4,17?-dimethyl-4-aza-5-androsten-17?-ol-3-one	C20 H31 N O2	317.2345	8.077	C15175	S205466		-0.001	-3.1644			14	level4					NULL	other
8.079_362.2922	0.788	0.3211		C22 H38 N2 O2	362.2922	8.079								0	level5						other
8.08_419.1972	0.7992	0.2238			419.1972	8.08								7	level5						other
8.082_317.0802	0.5321	0.1465		C18 H11 N3 O3	317.0802	8.082								0	level5						other
8.294_412.2589	0.242	0.2132		C23 H43 P3	412.2589	8.294								0	level5						other
8.332_425.3142	0.1045	0.2337		C24 H43 N O5	425.3142	8.332						78.7		0	level5						other
8.332_430.2694	0.1179	0.2455		C30 H38 S	430.2694	8.332								1	level5						other
8.462_447.335	0.6687	0.0794	N-stearoyltyrosine	C27 H45 N O4	447.335	8.462		S2284895		0.0001	0.3348			0	level4						other
8.556_276.209	0.287	0.3402	(e)-parinaric acid	C18 H28 O2	276.209	8.556		S4445965		0.0001	0.2003			1	level4						other
8.597_407.3036	0.3128	0.2985	Cassaidine	C24 H41 N O4	407.3036	8.597	C08669	S4444672		0.0001	0.2062			0	level4	Alkaloids	Alkaloids derived by amination reactions	Terpenoid alkaloids	Alkaloids	NULL	other
8.755_495.261	1.2206	0.2417		C25 H41 N3 O3 S2	495.261	8.755								2	level5						other
8.759_552.7872	1.2571	0.2642	[similar to: (+/-)5(6)-dihet; δmass: 214.5414 da]		552.7872	8.759								0	level5						other
8.775_306.1831	2.3402	0.2133	Di-n-amyl phthalate	C18 H26 O4	306.1831	8.775	C14300	S8243		0	-0.1588			0	level4					NULL	other
8.843_188.1566	0.2521	0.2355		C14 H20	188.1566	8.843								1	level5						other
8.991_453.382	0.7186	0.352		C27 H51 N O4	453.382	8.991								1	level5						other
9.176_590.3516	1.1995	0.463		C23 H56 N6 O5 P2 S	590.3516	9.176								1	level5						other
9.271_996.6192	0.6869	0.2514		C43 H95 N6 O13 P3	996.6192	9.271								0	level5						other
9.527_376.2589	0.5689	0.2143		C17 H37 N4 O3 P	376.2589	9.527								1	level5						other
9.684_358.1591	0.721	0.2261		C17 H22 N6 O S	358.1591	9.684								0	level5						other
9.71_287.2796	0.5565	0.1521			287.2796	9.71								15	level5						other
9.711_598.4275	0.6291	0.1348		C28 H65 N4 O3 P3	598.4275	9.711								0	level5						other
9.715_336.1596	0.7027	0.2273	Plinabulin	C19 H20 N4 O2	336.1596	9.715		S8125252		0.0009	2.764			1	level4						other
9.922_354.2532	0.5162	0.1381		C19 H34 N2 O4	354.2532	9.922								1	level5						other
0.54_212.0536	1.1458	0.4879		C6 H12 O8	212.0536	0.54								5	level5						other
0.543_141.0277	1.23	0.3718		C2 H7 N O6	141.0277	0.543								0	level5						other
0.543_81.0066	1.2985	0.2975			81.0066	0.543								0	level5						other
0.543_87.9831	1.2373	0.3919		C H N2 O P	87.9831	0.543								12	level5						other
0.544_103.9961	1.2305	0.3583	[similar to: n-acetyl-l-cysteine; δ mass: -59.0342 da]		103.9961	0.544								4	level5						other
0.544_118.0118	1.213	0.4022			118.0118	0.544								0	level5						other
0.544_119.0126	1.222	0.404			119.0126	0.544								2	level5						other
0.544_120.0093	1.2206	0.3972		C2 H5 N2 O2 P	120.0093	0.544								11	level5						other
0.544_131.9909	1.2223	0.4188	[similar to: l(+)-ornithine; δ mass: -0.0989 da]		131.9909	0.544								2	level5						other
0.544_150.0281	1.2308	0.3435		C3 H6 N2 O5	150.0281	0.544								0	level5						other
0.544_99.0172	1.259	0.3173			99.0172	0.544								0	level5						other
0.549_199.9659	1.4351	0.367		C3 H5 Cl N2 O4 S	199.9659	0.549								1	level5						other
0.549_94.0531	1.3656	0.2867	4-aminopyridine	C5 H6 N2	94.0531	0.549	C13728	S1664		0	0.0557			0	level4					NULL	other
0.552_217.9764	1.4997	0.3414		C5 H4 Cl N4 O2 P	217.9764	0.552								1	level5						other
0.565_115.0634	1.1342	0.5728	L-proline	C5 H9 N O2	115.0634	0.565	C16435	BGI376		0.0001	0.5165	94.5	89.2	2	level1					NULL	other
0.567_132.0899	1.145	0.5623	D-ornithine	C5 H12 N2 O2	132.0899	0.567	C00515	BGI245	HMDB0003374	0	0.1791	97.1	91.6	6	level1	Organic acids and derivatives	Carboxylic acids and derivatives	Amino acids, peptides, and analogues	Amino acids, peptides, and analogues	map00472 D-Arginine and D-ornithine metabolism; map01100 Metabolic pathways;	other
0.576_203.9498	1.1904	0.4009		C2 H7 O5 P3	203.9498	0.576								0	level5						other
0.578_271.937	1.2366	0.2898		C8 H O9 P	271.937	0.578								0	level5						other
0.58_287.9147	1.5195	0.5937		C4 H O13 P	287.9147	0.58								1	level5						other
0.58_339.9244	1.2275	0.3959		C8 H9 N2 O3 P S4	339.9244	0.58								5	level5						other
0.58_407.9118	1.1874	0.4141		C13 H6 N4 O2 P2 S3	407.9118	0.58								0	level5						other
0.582_475.8993	1.2089	0.3128		C17 H7 N2 O5 P3 S2	475.8993	0.582								1	level5						other
0.582_611.8742	1.2089	0.3466		C17 H11 O15 P3 S2	611.8742	0.582								0	level5						other
0.582_679.8613	1.0772	0.6991		C22 H6 N2 O16 P2 S2	679.8613	0.582								2	level5						other
0.586_109.0641	1.397	0.2652	3,4-diaminopyridine	C5 H7 N3	109.0641	0.586		S5705		0.0001	0.5361			4	level4						other
0.596_300.1335	0.8517	0.3466		C15 H27 P3	300.1335	0.596								0	level5						other
0.629_215.9154	0.8621	0.3594	Mutagen x	C5 H3 Cl3 O3	215.9154	0.629	C19205	S48451		0.0006	2.9686			2	level4					NULL	other

0.636_279.0846	0.808	0.2161		C12 H13 N3 O5	279.0846	0.636								4	level5						other
0.643_155.0349	1.3244	0.4111	N-methylethanolamine phosphate	C3 H10 N O4 P	155.0349	0.643	C01210	S388545	HMDB0060173	0.0002	1.1744			4	level4	Organic acids and derivatives	Organic phosphoric acids and derivatives	Phosphate esters	Organic acids	map00564 Glycerophospholipid metabolism;	other
0.648_381.7596	1.2161	0.3939		C H2 Cl O7 P3 S4	381.7596	0.648								4	level5						other
0.649_153.0403	0.5574	0.3578			153.0403	0.649								14	level5						other
0.655_112.0637	0.6857	0.2645		C5 H8 N2 O	112.0637	0.655								6	level5						other
0.66_260.0037	1.1218	0.3332		C7 H14 Cl O4 P S	260.0037	0.66								0	level5						other
0.671_117.1132	0.8713	0.3455			117.1132	0.671								3	level5						other
0.674_131.0695	0.7029	0.365	Creatine	C4 H9 N3 O2	131.0695	0.674	C00300	MReference-357	HMDB0000064	0	0.306	84.6	88.8	2	level1	Organic acids and derivatives	Carboxylic acids and derivatives	Amino acids, peptides, and analogues	Amino acids, peptides, and analogues	map00260 Glycine, serine and threonine metabolism; map00330 Arginine and proline metabolism; map01100 Metabolic pathways;	other
0.68_218.1632	0.2157	0.4419		C10 H22 N2 O3	218.1632	0.68								0	level5						other
0.68_256.1397	1.8651	0.2782			256.1397	0.68								5	level5						other
0.685_332.9031	1.6583	0.2688		C3 H N3 O12 P2	332.9031	0.685								0	level5						other
0.689_213.0572	1.6625	0.3348		C8 H11 N3 O2 S	213.0572	0.689								1	level5						other
0.69_143.0948	0.131	0.2272	Dl-stachydrine	C7 H13 N O2	143.0948	0.69		MReference-837		0.0001	1.0147		87.8	0	level2						other
0.69_174.1006	1.2392	0.4701	N-acetylornithine	C7 H14 N2 O3	174.1006	0.69		MReference-513		0.0002	1.1021		64.9	0	level2						other
0.691_246.1944	0.033	0.239		C12 H26 N2 O3	246.1944	0.691								0	level5						other
0.694_300.205	0.0124	0.1927		C16 H24 N6	300.205	0.694								1	level5						other
0.698_142.0744	1.2212	0.5619	Piracetam	C6 H10 N2 O2	142.0744	0.698		S4677		0.0002	1.2887			0	level4						other
0.699_201.9678	1.7436	0.9562			201.9678	0.699								0	level5						other
0.719_131.0583	0.6275	0.4585	N-acetyl-l-alanine	C5 H9 N O3	131.0583	0.719		BGI417	HMDB0000766	0.0001	0.6248	74.8	59.2	0	level2	Organic acids and derivatives	Carboxylic acids and derivatives	Amino acids, peptides, and analogues	Amino acids, peptides, and analogues		other
0.72_255.8134	0.9275	0.6009			255.8134	0.72								1	level5						other
0.72_781.4368	0.8455	0.4737			781.4368	0.72								0	level5						other
0.722_607.5603	0.8089	0.439			607.5603	0.722								1	level5						other
0.727_269.7902	0.7518	0.311			269.7902	0.727								4	level5						other
0.741_129.1267	1.3285	0.3162		C6 H15 N3	129.1267	0.741								0	level5						other
0.789_322.1869	0.0252	0.1979		C14 H33 N2 P3	322.1869	0.789								0	level5						other
0.79_308.1712	0.0276	0.2008		C10 H25 N6 O3 P	308.1712	0.79								0	level5						other
0.794_94.0089	0.5256	0.4652	Methylsulfonylmethane	C2 H6 O2 S	94.0089	0.794	C11142	S5978	HMDB0004983	0.0001	0.9273			0	level4	Organosulfur compounds	Sulfonyls	Sulfones	Sulfones	map00920 Sulfur metabolism; map01100 Metabolic pathways;	other
0.796_158.0206	0.8433	0.375			158.0206	0.796								0	level5						other
0.91_112.0274	0.3738	0.2627	Uracil	C4 H4 N2 O2	112.0274	0.91	C00106	MReference-2531	HMDB0000300	0.0001	0.881		90.8	0	level2	Organoheterocyclic compounds	Diazines	Pyrimidines and pyrimidine derivatives	Pyrimidines and pyrimidine derivatives	map00240 Pyrimidine metabolism; map00410 beta-Alanine metabolism; map00770 Pantothenate and CoA biosynthesis; map01100 Metabolic pathways;	other
0.999_122.0481	0.188	0.2204	Nicotinamide	C6 H6 N2 O	122.0481	0.999	C00153	MReference-517	HMDB0001406	0.0001	0.4852		92.4	0	level1	Organoheterocyclic compounds	Pyridines and derivatives	Pyridinecarboxylic acids and derivatives	Pyridine and derivatives	map00760 Nicotinate and nicotinamide metabolism; map01100 Metabolic pathways; map04977 Vitamin digestion and absorption;	other
1.025_103.0456	1.7341	0.2361	2-methylthiazolidine	C4 H9 N S	103.0456	1.025		S29856	HMDB0031682	0.0001	0.6594			15	level4	Organoheterocyclic compounds	Azolidines	Thiazolidines	Thiazolidines		other
1.025_132.0246	1.7022	0.2347	3-methylsulfolene	C5 H8 O2 S	132.0246	1.025		S64103	HMDB0059667	0.0001	0.7315	89.5		9	level4	Organoheterocyclic compounds	Dihydrothiophenes	null	Dihydrothiophenes		other
1.128_152.0335	0.3673	0.2391	Xanthine	C5 H4 N4 O2	152.0335	1.128	C00385	S1151	HMDB0000292	0.0001	0.5823			4	level4	Organoheterocyclic compounds	Imidazopyrimidines	Purines and purine derivatives	Purines and derivatives	map00230 Purine metabolism; map00232 Caffeine metabolism; map01100 Metabolic pathways;	other
1.157_191.0253	1.384	0.334		C6 H9 N O4 S	191.0253	1.157								21	level5						other

1.167_192.0271	1.3862	0.3498	Citric acid	C6 H8 O7	192.0271	1.167	C00158	S305	HMDB0000094	0.0001	0.4774			4	level4	Organic acids and derivatives	Carboxylic acids and derivatives	Tricarboxylic acids and derivatives	Organic acids	map00020 Citrate cycle (TCA cycle); map00250 Alanine, aspartate and glutamate metabolism; map00630 Glyoxylate and dicarboxylate metabolism; map01100 Metabolic pathways; map01200 Carbon metabolism; map01210 2-Oxocarboxylic acid metabolism; map01230 Biosynthesis of amino acids; map04742 Taste transduction; map04922 Glucagon signaling [illegible]	other
1.167_214.009	1.4404	0.3035			214.009	1.167								1	level5						other
1.324_162.043	1.6027	0.3326	Quindoxin	C8 H6 N2 O2	162.043	1.324		S65059	HMDB0032927	0.0001	0.5885			2	level4	Organoheterocyclic compounds	Diazanaphthalenes	Benzodiazines	Benzodiazines		other
1.44_131.0534	0.4851	0.9968			131.0534	1.44								0	level5						other
1.712_131.0539	1.2948	0.6553			131.0539	1.712								0	level5						other
1.904_268.0809	1.6437	0.3105	Inosine	C10 H12 N4 O5	268.0809	1.904	C00294	BGI328	HMDB0000195	0.0001	0.4102	99.1	87.5	0	level1	Nucleosides, nucleotides, and analogues	Purine nucleosides	null	Purines and derivatives	map00230 Purine metabolism; map01100 Metabolic pathways; map02010 ABC transporters;	other
11.296_368.3443	0.5336	0.3027		C27 H44	368.3443	11.296								0	level5						other
2.36_163.0635	0.8783	0.5986	Î³-oxo-3-pyridinebutanal	C9 H9 N O2	163.0635	2.36	C19567	S102987	HMDB0062406	0.0002	1.021			0	level4	Organic oxygen compounds	Organooxygen compounds	Carbonyl compounds	Carbonyl compounds	map00760 Nicotinate and nicotinamide metabolism; map00980 Metabolism of xenobiotics by cytochrome P450; map01100 Metabolic pathways; map05204 Chemical carcinogenesis;	other
2.421_172.0713	0.8474	0.5151		C4 H8 N6 O2	172.0713	2.421								0	level5						other
2.579_127.1004	1.026	0.7948	Lq1825000	C7 H13 N O	127.1004	2.579								2	level5						other
2.593_165.0523	0.631	0.4063	[similar to: l-phenylalanine; δmass: -0.0267 da]		165.0523	2.593								0	level5						other
2.631_157.0826	0.812	0.4386	[similar to: 2,6-di-tert-butyl-1,4-benzoquinone; δmass: -63.0637 da]		157.0826	2.631								0	level5						other
2.72_168.0899	1.3997	0.3788	Pyridoxamine	C8 H12 N2 O2	168.0899	2.72	C00534	MReference-568	HMDB0001431	0.0001	0.3817	15.7	51.9	0	level3	Organoheterocyclic compounds	Pyridines and derivatives	Pyridoxamines	Amines and derivatives	map00750 Vitamin B6 metabolism; map01100 Metabolic pathways; map04977 Vitamin digestion and absorption;	other
2.797_230.1267	1.2107	0.5074	Valyl-4-hydroxyproline	C10 H18 N2 O4	230.1267	2.797		S8418431		0.0001	0.3026			0	level4						other
2.817_327.1318	1.829	0.3644	(2s)-3-phenyl-2-({[(3s,4s,5r)-2,3,4-trihydroxy-5-(hydroxymethyl)tetrahydro-2-furanyl]methyl}amino)propanoic acid (non-preferred name)	C15 H21 N O7	327.1318	2.817		S35014483	HMDB0037846	0	-0.1426			0	level4	Organic acids and derivatives	Carboxylic acids and derivatives	Amino acids, peptides, and analogues	Amino acids, peptides, and analogues		other
2.891_297.1212	1.98	0.2432	N-(2-phenylethyl)-beta-d-glucopyranuronosylamine	C14 H19 N O6	297.1212	2.891		S166036	HMDB0010323	0	0.0572			3	level4	Organic oxygen compounds	Organooxygen compounds	Carbohydrates and carbohydrate conjugates	Carbohydrates		other
2.921_253.1526	0.9179	0.5337		C10 H23 N O6	253.1526	2.921								0	level5						other
2.996_244.0559	0.8717	0.3872		C7 H12 N6 S2	244.0559	2.996								0	level5						other
3.018_188.1163	0.9273	0.8496	Glycyl-l-leucine	C8 H16 N2 O3	188.1163	3.018	C02155	MReference-430	HMDB0000759	0.0002	1.2175		87.9	0	level2	Organic acids and derivatives	Carboxylic acids and derivatives	Amino acids, peptides, and analogues	Amino acids, peptides, and analogues	NULL	other
3.034_208.0948	0.8794	0.3281	Dambonitol	C8 H16 O6	208.0948	3.034		S10254650	HMDB0033942	0.0001	0.5183			0	level4	Organic oxygen compounds	Organooxygen compounds	Alcohols and polyols	Alcohols		other
3.084_383.1079	0.8454	0.2925	Succinyladenosine	C14 H17 N5 O8	383.1079	3.084		S17216022	HMDB0000912	0.0002	0.5436			14	level4	Nucleosides, nucleotides, and analogues	Purine nucleosides	null	Purines and derivatives		other
3.091_295.142	0.1918	0.2793		C15 H21 N O5	295.142	3.091								0	level5						other
3.206_209.0394	1.8963	0.5088		C13 H8 N P	209.0394	3.206								0	level5						other

3.218_187.1337	0.5496	0.6537	[similar to: dl-tryptophan; δ mass: -16.9562 da]		187.1337	3.218								5	level5						other
3.24_320.1372	1.7378	0.4339	Eterobarb	C16 H20 N2 O5	320.1372	3.24		S31271		0	0.0851			0	level4						other
3.242_147.0685	0.9249	0.4987	Indole-3-carbidol	C9 H9 N O	147.0685	3.242		S3581	HMDB0005785	0.0001	0.8203			0	level4	Organoheterocyclic compounds	Indoles and derivatives	Indoles	Indoles		other
3.303_283.1995	0.6975	0.2108		C13 H25 N5 O2	283.1995	3.303								0	level5						other
3.325_101.0842	0.2932	0.2431	Zv4	C5 H11 N O	101.0842	3.325	C01842	S11795		0.0001	1.1933			0	level4	FA Fatty acyls	FA08 Fatty amides	FA0801 Primary amides	Fatty acyls[FA]	NULL	other
3.385_161.0478	0.64	0.368	2-indolylformic acid	C9 H7 N O2	161.0478	3.385		S65731	HMDB0002285	0.0001	0.7047	16.2		0	level4	Organoheterocyclic compounds	Indoles and derivatives	Indolecarboxylic acids and derivatives	Indole and derivatives		other
3.428_180.0536	1.626	0.298	Nicotinuric acid	C8 H8 N2 O3	180.0536	3.428	C05380	MReference - 520	HMDB0003269	0.0001	0.6506		79.8	1	level2	Organic acids and derivatives	Carboxylic acids and derivatives	Amino acids, peptides, and analogues	Amino acids, peptides, and analogues	map00760 Nicotinate and nicotinamide metabolism;	other
3.43_327.2252	0.7881	0.1574		C18 H35 N P2	327.2252	3.43								4	level5						other
3.431_230.1995	2.5383	0.2036		C12 H26 N2 O2	230.1995	3.431								5	level5						other
3.434_315.1681	1.1262	0.306	Alizapride	C16 H21 N5 O2	315.1681	3.434		S39202	HMDB0015494	-0.0014	-4.5926			0	level4	Organoheterocyclic compounds	Benzotriazoles	null	Benzotriazoles		other
3.463_170.1058	2.0612	0.2435	Levetiracetam	C8 H14 N2 O2	170.1058	3.463	C07841	S4447633	HMDB0015333	0.0002	1.3138	24.9		0	level4	Organic acids and derivatives	Carboxylic acids and derivatives	Amino acids, peptides, and analogues	Amino acids, peptides, and analogues	NULL	other
3.489_283.1995	1.0688	0.3087		C13 H25 N5 O2	283.1995	3.489								0	level5						other
3.655_129.1519	0.9691	0.6383	Octylamine	C8 H19 N	129.1519	3.655	C01740	S7851		0.0001	1.0431			14	level4					NULL	other
3.731_297.1688	2.3539	0.3405		C14 H23 N3 O4	297.1688	3.731								0	level5						other
3.866_231.0531	0.8339	0.4179	Eglinazine	C7 H10 Cl N5 O2	231.0531	3.866	C19102	S98703		0.0008	3.4556			4	level4					NULL	other
4.02_415.1716	1.6092	0.2594	Sabeluzole	C22 H26 F N3 O2 S	415.1716	4.02		S53964		-0.0014	-3.2721			0	level4						other
4.104_385.2311	0.8378	0.3582		C17 H31 N5 O5	385.2311	4.104								0	level5						other
4.161_371.2238	0.5523	0.2837	Tamoxifen	C26 H29 N O	371.2238	4.161	C07108	S2015313	HMDB0014813	-0.0011	-3.0777			0	level4	Phenylpropanoids and polyketides	Stilbenes	null	Polyketides[PK]	map00982 Drug metabolism - cytochrome P450;	other
4.162_75.0321	0.4105	0.2934	Glycine	C2 H5 N O2	75.0321	4.162	C00037	S730	HMDB0000123	0.0001	1.3165			0	level4	Organic acids and derivatives	Carboxylic acids and derivatives	Amino acids, peptides, and analogues	Amino acids	map00120 Primary bile acid biosynthesis; map00230 Purine metabolism; map00260 Glycine, serine and threonine metabolism; map00310 Lysine degradation; map00440 Phosphonate and phosphinate metabolism; map00480 Glutathione metabolism; map00630 Glyoxylate and dicarboxylate metabolism; map00730 Thiamine metabolism; map00860 Porphyrin and chlorophyll [illegible]	other
4.31_201.0826	1.206	0.4113		C9 H15 N O2 S	201.0826	4.31								0	level5						other
4.321_376.1384	0.5801	0.3962	(+)-riboflavin	C17 H20 N4 O6	376.1384	4.321		S6501		0.0001	0.2809			12	level4						other
4.344_134.0369	0.1539	0.2514	Ortho-phthalaldehyde	C8 H6 O2	134.0369	4.344		S4642		0.0001	0.5662	80.3	82.9	33	level4						other
4.367_213.0403	1.8324	0.2651	N-lactoyl ethanolamine phosphate	C5 H12 N O6 P	213.0403	4.367		S9725045	HMDB0032357	0.0001	0.3606			34	level4	Organic acids and derivatives	Organic phosphoric acids and derivatives	Phosphate esters	Organic acids		other
4.399_279.1654	1.2994	0.2959	Drepamon	C16 H25 N O S	279.1654	4.399	C18898	S34422		-0.0003	-0.941			15	level4					NULL	other
4.404_278.1625	1.5649	0.2967	Ro 20-1724	C15 H22 N2 O3	278.1625	4.404		S4908	HMDB0062483	-0.0006	-2.0299			0	level4	Benzenoids	Phenol ethers	Anisoles	Benzene and derivatives		other
4.428_331.1988	2.5079	0.3845		C16 H29 N O6	331.1988	4.428								0	level5						other
4.457_193.0741	1.4894	0.415	Methylhippuric acid	C10 H11 N O3	193.0741	4.457		MReference - 490	HMDB0000859	0.0002	0.9947		82.1	8	level2	Organic acids and derivatives	Carboxylic acids and derivatives	Amino acids, peptides, and analogues	Amino acids, peptides, and analogues		other
4.715_178.0995	1.2127	0.5841	Benzyl butyrate	C11 H14 O2	178.0995	4.715		S7367	HMDB0033376	0.0001	0.8392			3	level4	Benzenoids	Benzene and substituted derivatives	Benzyloxycarbonyls	Benzene and derivatives		other
4.767_475.0203	1.5279	0.8261		C16 H19 N3 O6 P2 S2	475.0203	4.767								0	level5						other
4.77_309.1788	1.303	0.2222		C13 H27 N O7	309.1788	4.77								8	level5						other
4.791_364.1536	1.8074	0.286	Ganetespib	C20 H20 N4 O3	364.1536	4.791		S28189074		0.0001	0.2805			0	level4						other
4.841_200.0952	1.9944	0.2581	4,4'-oxydianiline	C12 H12 N2 O	200.0952	4.841	C14759	S7298		0.0002	1.0048			1	level4					NULL	other
4.841_246.1005	1.8935	0.2666	N-acetyl-d-tryptophan	C13 H14 N2 O3	246.1005	4.841		BGI415		0	0.1743	94.6	89.9	0	level1						other
4.842_180.1152	2.3303	0.1864	3-bha	C11 H16 O2	180.1152	4.842		S8145	HMDB0059925	0.0001	0.6976			71	level4	Benzenoids	Phenols	Methoxyphenols	Phenols and derivatives		other
4.844_120.094	1.8753	0.311	Cumene	C9 H12	120.094	4.844	C14396	S7128	HMDB0034029	0.0001	0.7169			0	level4	Benzenoids	Benzene and substituted derivatives	Cumenes	Benzene and derivatives	NULL	other
4.868_161.0842	2.0039	0.2567	Tryptophol	C10 H11 N O	161.0842	4.868	C00955	S10235	HMDB0003447	0.0001	0.7695			0	level4	Organoheterocyclic compounds	Indoles and derivatives	Indoles	Indoles	map00380 Tryptophan metabolism;	other
4.869_207.0897	2.0865	0.2523	N-acetyl-l-phenylalanine	C11 H13 N O3	207.0897	4.869	C03519	BGI422	HMDB0000512	0.0002	0.7811	94.6		0	level1	Organic acids and derivatives	Carboxylic acids and derivatives	Amino acids, peptides, and analogues	Amino acids, peptides, and analogues	map00360 Phenylalanine metabolism; map01100 Metabolic pathways;	other
4.872_119.0736	2.0237	0.2506		C8 H9 N	119.0736	4.872								3	level5						other

4.872_165.0791	1.8644	0.2513	L-phenylalanine	C9 H11 N O2	165.0791	4.872	C00079	MReference-8	HMDB0000159	0.0001	0.6556		94.3	0	level2	Organic acids and derivatives	Carboxylic acids and derivatives	Amino acids, peptides, and analogues	Amino acids	map00360 Phenylalanine metabolism; map00400 Phenylalanine, tyrosine and tryptophan biosynthesis; map00970 Aminoacyl-tRNA biosynthesis; map01100 Metabolic pathways; map01210 2-Oxocarboxylic acid metabolism; map01230 Biosynthesis of amino acids; map02010 ABC transporters; map04974 Protein digestion and absorption; map04978 Mineral absorption; [illegible]	other
4.893_568.3069	0.7185	0.1235	Protoporphyrinogen	C34 H40 N4 O4	568.3069	4.893	C01079	S108741	HMDB0001097	0.002	3.4479			0	level4	Organoheterocyclic compounds	Tetrapyrroles and derivatives	Porphyrins	Porphyrins	map00860 Porphyrin and chlorophyll metabolism; map01100 Metabolic pathways;	other
4.894_643.3089	0.7789	0.208		C27 H56 N3 O6 P3 S	643.3089	4.894								1	level5						other
4.932_502.2992	1.0811	0.2378		C23 H42 N4 O8	502.2992	4.932								0	level5						other
4.949_156.0788	1.4613	0.3289	2,5-dimethyl-4-ethoxy-3(2h)-furanone	C8 H12 O3	156.0788	4.949		S2285292	HMDB0032232	0.0001	0.9475	91.8		1	level4	Organoheterocyclic compounds	Dihydrofurans	Furanones	Furanones		other
4.973_166.0268	0.3301	0.3118	Phthalic acid	C8 H6 O4	166.0268	4.973	C01606	S992	HMDB0002107	0.0002	1.0139			1	level4	Benzenoids	Benzene and substituted derivatives	Benzoic acids and derivatives	Benzene and derivatives	map01100 Metabolic pathways; map02010 ABC transporters;	other
5.001_421.1982	1.7832	0.1361	Fenpyroximate	C24 H27 N3 O4	421.1982	5.001	C11098	S7850857		-0.002	-4.6486			0	level4					NULL	other
5.021_310.0839	1.0728	0.704	6-deoxyjacareubin	C18 H14 O5	310.0839	5.021	C10059	S4444948		-0.0002	-0.5902			0	level4	Polyketides	Pyrones	Xanthenes	Polyketides[PK]	NULL	other
5.099_247.1321	0.6884	0.3885	Parbendazole	C13 H17 N3 O2	247.1321	5.099		S24775		0	0.1715			10	level4						other
5.158_414.2581	1.3574	0.2336		C17 H38 N2 O9	414.2581	5.158								0	level5						other
5.158_461.2658	1.2456	0.128		C23 H45 N O2 P2 S	461.2658	5.158								0	level5						other
5.159_397.2313	1.3856	0.1667		C17 H35 N O9	397.2313	5.159								2	level5						other
5.178_651.4045	0.7044	0.1883		C29 H57 N5 O11	651.4045	5.178								6	level5						other
5.214_604.3306	0.8875	0.3066		C22 H48 N6 O13	604.3306	5.214								12	level5						other
5.216_458.2731	1.0829	0.3206		C21 H38 N4 O7	458.2731	5.216								19	level5						other
5.306_414.149	0.8292	0.2374	N-desisopropyl delavirdine	C19 H22 N6 O3 S	414.149	5.306		S411444	HMDB0061021	0.0016	3.9243			8	level4	Organoheterocyclic compounds	Diazinanes	Piperazines	Piperazines		other
5.315_441.2577	1.3158	0.3444		C20 H35 N5 O6	441.2577	5.315								0	level5						other
5.316_458.2844	1.3047	0.4402		C19 H42 N2 O10	458.2844	5.316								46	level5						other
5.336_324.1785	0.8986	0.3044		C14 H28 O8	324.1785	5.336								6	level5						other
5.34_414.2468	1.2242	0.3611		C18 H38 O10	414.2468	5.34								10	level5						other
5.341_509.2509	1.7285	0.3674		C23 H45 N O5 P2 S	509.2509	5.341								0	level5						other
5.448_354.7047	0.9126	0.2963	[similar to: (+/-)5(6)-dihet; δmass: 16.4590 da]		354.7047	5.448								0	level5						other
5.55_387.7088	0.9138	0.2581	[similar to: (+/-)5(6)-dihet; δmass: 49.4630 da]		387.7088	5.55								17	level5						other
5.552_384.2452	1.0591	0.427	Ibutilide	C20 H36 N2 O3 S	384.2452	5.552	C07753	S54755	HMDB0014453	0.0006	1.448			0	level4	Benzenoids	Benzene and substituted derivatives	Phenylbutylamines	Amines and derivatives	NULL	other
5.582_546.3393	1.3587	0.2714	3-î±-mycarosylerythronolide b	C28 H50 O10	546.3393	5.582	C06630	S389908		-0.0011	-1.9319			5	level4					map01100 Metabolic pathways;	other
5.603_597.3036	1.7774	0.2991		C24 H47 N5 O10 S	597.3036	5.603								0	level5						other
5.647_410.1872	1.079	0.3673	Benfuracarb	C20 H30 N2 O5 S	410.1872	5.647	C11073	S49560		-0.0004	-0.8699			4	level4					NULL	other
5.689_262.0351	1.4976	0.2703		C10 H6 N4 O5	262.0351	5.689								0	level5						other
5.689_430.1288	1.8657	0.292		C19 H26 O9 S	430.1288	5.689								0	level5						other
5.734_431.7348	0.9146	0.6894	[similar to: (+/-)5(6)-dihet; δmass: 93.4891 da]		431.7348	5.734								0	level5						other
5.739_427.7697	1.1037	0.4434	[similar to: (+/-)5(6)-dihet; δmass: 89.5240 da]		427.7697	5.739								0	level5						other
5.753_681.3972	1.2024	0.2539		C35 H60 N3 O6 P S	681.3972	5.753								0	level5						other
5.87_160.0526	1.236	0.3247	Qj4750000	C10 H8 O2	160.0526	5.87	C16213	S6824		0.0002	0.9632		87.2	0	level4						other
5.887_678.4159	1.345	0.175		C33 H64 N2 O8 P2	678.4159	5.887								0	level5						other
5.964_482.272	1.1147	0.4129		C18 H38 N6 O9	482.272	5.964								0	level5						other
6.21_233.0477	0.9386	0.4364		C7 H13 N3 O2 P2	233.0477	6.21								0	level5						other

6.442_362.2093	2.1084	0.1358	Cortisol	C21 H30 O5	362.2093	6.442	C00735	MReference-679	HMDB0000063	0	-0.0116		67.2	44	level1	Lipids and lipid-like molecules	Steroids and steroid derivatives	Hydroxysteroids	Steroids and derivatives	map00140 Steroid hormone biosynthesis; map01100 Metabolic pathways; map04080 Neuroactive ligand-receptor interaction; map04927 Cortisol synthesis and secretion; map04934 Cushing syndrome; map04960 Aldosterone-regulated sodium reabsorption; map04976 Bile secretion; map05200 Pathways in cancer; map05215 Prostate cancer;	other
6.504_330.0739	0.0699	0.199	Aflatoxin g2	C17 H14 O7	330.0739	6.504	C16754	MReference-302	HMDB0030475	0	-0.0671		69.5	0	level2	Phenylpropanoids and polyketides	Coumarins and derivatives	Furanocoumarins	Coumarins and derivatives	map01100 Metabolic pathways;	other
6.519_211.0127	0.0074	0.2084		C9 H9 N O S2	211.0127	6.519								0	level5						other
6.552_693.4516	1.1382	0.2752		C32 H63 N5 O11	693.4516	6.552								0	level5						other
6.605_330.1977	0.85	0.5462		C16 H30 N2 O3 S	330.1977	6.605								1	level5						other
6.616_737.4784	1.112	0.4463		C36 H74 N3 O6 P3	737.4784	6.616								3	level5						other
6.701_226.1207	1.4741	0.3259	Butopyronoxyl	C12 H18 O4	226.1207	6.701	C19142	S10306		0.0001	0.6381			0	level4					NULL	other
6.759_306.1944	0.2911	0.2532	Roxatidine	C17 H26 N2 O3	306.1944	6.759		S82423		0	0.1337			4	level4						other
6.81_134.0733	0.7656	0.1188	Cinnamyl alcohol	C9 H10 O	134.0733	6.81	C02394	S21105870	HMDB0029698	0.0001	0.7401			0	level4	Phenylpropanoids and polyketides	Cinnamyl alcohols	null	Cinnamyl alcohols		other
7.003_247.0633	0.8612	0.3481	Tinidazole	C8 H13 N3 O4 S	247.0633	7.003		S5279	HMDB0015047	0.0006	2.4993			8	level4	Organoheterocyclic compounds	Azoles	Imidazoles	Imidazoles		other
7.347_218.1308	0.2169	0.2001	Cis-3-hexenyl phenylacetate	C14 H18 O2	218.1308	7.347		S4519184	HMDB0038281	0.0001	0.622			22	level4	Benzenoids	Benzene and substituted derivatives	null	Benzene and derivatives		other
7.591_287.2823	0.8661	0.4697	Sphinganine (d17:0)	C17 H37 N O2	287.2823	7.591		S2497508		-0.0001	-0.3838			0	level4						other
7.817_144.9483	1.5726	0.274		C3 H N O2 P2	144.9483	7.817								0	level5						other
7.819_142.9529	1.5969	0.3572			142.9529	7.819								7	level5						other
7.831_148.0891	0.0981	0.3557	Cuminaldehyde	C10 H12 O	148.0891	7.831	C06577	MReference-359	HMDB0002214	0.0003	2.0132		38	19	level3	Lipids and lipid-like molecules	Prenol lipids	Monoterpenoids	Terpenoids	map01100 Metabolic pathways;	other
8.053_388.2613	0.1748	0.2818	3,7-diketo-5β-cholan-24-oic acid	C24 H36 O4	388.2613	8.053		S85006		0	-0.0979			10	level4						other
8.089_511.3276	0.5988	0.5117		C25 H46 N5 O4 P	511.3276	8.089								0	level5						other
8.099_264.0871	0.9349	0.5076			264.0871	8.099								0	level5						other
8.114_284.1987	2.1856	0.2232	(10s)-juvenile hormone iii diol	C16 H28 O4	284.1987	8.114	C16505	S9034333		-0.0001	-0.2352			0	level4						other
8.114_292.1651	2.1129	0.2296		C14 H31 P3	292.1651	8.114								5	level5						other
8.118_326.1023	1.9417	0.2544		C15 H19 Cl N2 O4	326.1023	8.118								1	level5						other
8.138_334.2119	0.4547	0.5205		C16 H34 N2 O S2	334.2119	8.138								0	level5						other
8.143_409.3192	0.1165	0.2325	Deoxycholate	C24 H40 O4	409.3192	8.143						79.3		0	level5						other
8.216_208.1101	1.4489	0.3306	Asarone	C12 H16 O3	208.1101	8.216	C17846	S552532	HMDB0031469	0.0002	0.9476			0	level4	Benzenoids	Phenol ethers	Anisoles	Benzene and derivatives	NULL	other
8.318_336.2277	0.4398	0.4295		C16 H28 N6 O2	336.2277	8.318							59.7	0	level5						other
8.331_453.3455	0.0609	0.3418		C20 H48 N5 O4 P	453.3455	8.331								2	level5						other
8.556_316.2014	0.2264	0.2867		C16 H24 N6 O	316.2014	8.556							61.2	0	level5						other
8.596_539.359	0.4867	0.3575		C27 H50 N5 O4 P	539.359	8.596								1	level5						other
8.6_412.2589	0.2809	0.3371		C22 H32 N6 O2	412.2589	8.6								0	level5						other
8.699_254.2246	0.5459	0.4092	Palmitoleic acid	C16 H30 O2	254.2246	8.699	C08362	MReference-1256	HMDB0003229	0	0.1185	72.5	84.5	1	level2	Lipids and lipid-like molecules	Fatty Acyls	Fatty acids and conjugates	Fatty acyls[FA]	map00061 Fatty acid biosynthesis;	other
8.781_318.217	0.6193	0.3583		C16 H26 N6 O	318.217	8.781							67.8	0	level5						other
8.842_766.5748	0.089	0.3195		C45 H85 O3 P3	766.5748	8.842								2	level5						other
8.86_436.2979	0.1627	0.3144	Bolmantalate	C29 H40 O3	436.2979	8.86		S10128607		0.0001	0.3427			0	level4						other
8.914_320.2327	1.2106	0.3473		C16 H36 N2 S2	320.2327	8.914							61.4	0	level5						other
9.116_511.3276	0.4664	0.6226		C25 H46 N5 O4 P	511.3276	9.116								2	level5						other
9.178_973.4962	0.9002	0.4482		C53 H86 N O3 P3 S3	973.4962	9.178								6	level5						other
9.184_582.2143	1.1043	0.5497		C33 H34 N4 O2 S2	582.2143	9.184								0	level5						other
9.193_708.3754	1.1618	0.2342		C36 H60 N4 O4 S3	708.3754	9.193								3	level5						other
9.196_183.0662	0.8237	0.4046		C5 H14 N O4 P	183.0662	9.196								2	level5						other
9.314_507.3327	0.7285	0.4779	Elacytarabine	C27 H45 N3 O6	507.3327	9.314		S4943338		0.0019	3.7417			0	level4						other
9.44_453.2859	0.7822	0.2034	Glycerophospho-n-palmitoyl ethanolamine	C21 H44 N O7 P	453.2859	9.44		MReference-2955		0.0003	0.7474		44.1	0	level3						other
9.492_523.7568	0.8412	0.4754	[similar to: cytidine 5'-diphosphocholine; δmass: 35.6495 da]		523.7568	9.492								0	level5						other
9.493_1003.4892	0.7587	0.253		C60 H77 N O6 S3	1003.4892	9.493								0	level5						other
9.494_510.2576	0.8092	0.3492		C30 H34 N6 S	510.2576	9.494								0	level5						other
9.502_915.4362	0.8793	0.5061		C47 H73 N5 O3 S5	915.4362	9.502								4	level5						other
9.516_739.3306	0.8322	0.4254		C40 H57 N3 O2 S4	739.3306	9.516								0	level5						other
9.524_334.1441	0.5434	0.2898	Pimobendan	C19 H18 N4 O2	334.1441	9.524		S4657		0.0011	3.4277			0	level4						other
9.525_651.2781	0.806	0.3561		C31 H49 N5 O2 S4	651.2781	9.525								10	level5						other

9.611_286.2296	0.678	0.3469	Retinol	C20 H30 O	286.2296	9.611	C00473	BGI501		0	-0.0887	21.4		1	level3	Vitamins and Cofactors	Vitamins	Fat-soluble vitamins [Fig]	Vitamins	map00830 Retinol metabolism; map01100 Metabolic pathways; map04977 Vitamin digestion and absorption;	other
9.715_334.1592	0.6967	0.3169	9-fluoro-16alpha-hydroxyandrost-4-ene-3,11,17-trione	C19 H23 F O4	334.1592	9.715	C15105	S206745		0.0011	3.3715			1	level4					NULL	other
9.955_358.3082	0.6131	0.3988	1-stearoylglycerol	C21 H42 O4	358.3082	9.955	D01947	MReference-939		-0.0001	-0.392		85.1	7	level2						other
9.958_380.2902	0.6107	0.3935		C19 H36 N6 O2	380.2902	9.958								0	level5						other
0.544_145.0226	1.1287	0.4732	[similar to: spermine; δ mass: -57.1931 da]		145.0226	0.544								0	level5						other
0.567_129.079	0.8716	0.2292	L-pipecolic acid	C6 H11 N O2	129.079	0.567	C00408	BGI375	HMDB0000716	0.0001	0.5458	98.8	97.3	0	level1	Alkaloids	Alkaloids derived from lysine	Piperidine alkaloids	Alkaloids	map00310 Lysine degradation; map01100 Metabolic pathways;	other
0.567_146.1056	0.8694	0.224	L-lysine	C6 H14 N2 O2	146.1056	0.567	C00047	BGI22	HMDB0000182	0.0001	0.3946	87	84.7	25	level1	Peptides	Amino acids	Common amino acids [Fig]	Amino acids	map00310 Lysine degradation; map00780 Biotin metabolism; map00970 Aminoacyl-tRNA biosynthesis; map01100 Metabolic pathways; map01210 2-Oxocarboxylic acid metabolism; map01230 Biosynthesis of amino acids; map02010 ABC transporters; map04974 Protein digestion and absorption;	other
0.574_219.9274	1.4183	0.4165		C2 H7 O4 P3 S	219.9274	0.574								10	level5						other
0.582_747.8491	1.1724	0.3902		C19 H7 N6 O17 P3 S2	747.8491	0.582								0	level5						other
0.604_843.7888	0.8833	0.3696			843.7888	0.604								2	level5						other
0.606_639.8254	0.961	0.537			639.8254	0.606								0	level5						other
0.631_147.928	0.8961	0.4813	[similar to: l-glutathione (reduced); δ mass: -159.1558 da]		147.928	0.631								0	level5						other
0.632_196.0936	1.0964	0.6251			196.0936	0.632								0	level5						other
0.645_323.8007	1.1325	0.4426			323.8007	0.645								0	level5						other
0.646_423.8642	1.0742	0.6695		C7 H Cl O17 S	423.8642	0.646								0	level5						other
0.653_163.0221	1.3202	0.3537		C5 H10 N O P S	163.0221	0.653								0	level5						other
0.655_220.9807	1.4778	0.4183		C5 H3 N O9	220.9807	0.655								0	level5						other
0.656_169.0852	0.7311	0.4167	N.pi.-methyl-l-histidine	C7 H11 N3 O2	169.0852	0.656	C01152	S58494	HMDB0000479	0.0001	0.5082			0	level4	Organic acids and derivatives	Carboxylic acids and derivatives	Amino acids, peptides, and analogues	Amino acids, peptides, and analogues	map00340 Histidine metabolism; map01100 Metabolic pathways;	other
0.657_119.0582	1.1652	0.5255	Dl-homoserine	C4 H9 N O3	119.0582	0.657	C00263	MReference-2604	HMDB0000719	0	-0.3089	71.9	73.8	0	level2	Organic acids and derivatives	Carboxylic acids and derivatives	Amino acids, peptides, and analogues	Amino acids, peptides, and analogues	map00260 Glycine, serine and threonine metabolism; map00270 Cysteine and methionine metabolism; map00920 Sulfur metabolism; map01100 Metabolic pathways; map01230 Biosynthesis of amino acids;	other
0.657_230.1377	1.0821	0.6402			230.1377	0.657								0	level5						other
0.663_375.9209	1.0428	0.6637		C3 H2 N6 O12 P2	375.9209	0.663								5	level5						other
0.665_146.0692	0.9606	0.5802	L-glutamine	C5 H10 N2 O3	146.0692	0.665	C00064	BGI357	HMDB0000641	0	0.1909	77.7	77.6	0	level1	Peptides	Amino acids	Common amino acids [Fig]	Amino acids	map00220 Arginine biosynthesis; map00230 Purine metabolism; map00240 Pyrimidine metabolism; map00250 Alanine, aspartate and glutamate metabolism; map00471 D-Glutamine and D-glutamate metabolism; map00630 Glyoxylate and dicarboxylate metabolism; map00910 Nitrogen metabolism; map00970 Aminoacyl-tRNA biosynthesis; map01100 Metabolic	other
0.685_116.0667	1.2734	0.4387			116.0667	0.685								6	level5						other

0.685_161.0689	1.129	0.4637	.alpha.-aminoadipic acid	C6 H11 N O4	161.0689	0.685		S456		0.0001	0.4639	8.4		0	level4						other
0.686_189.1116	0.6004	0.4646	Homocitrulline	C7 H15 N3 O3	189.1116	0.686	C02427	S58582	HMDB0000679	0.0002	1.3094			0	level4	Organic acids and derivatives	Carboxylic acids and derivatives	Amino acids, peptides, and analogues	Amino acids, peptides, and analogues	NULL	other
0.688_110.0482	1.0452	0.5192	Ni3400000	C5 H6 N2 O	110.0482	0.688	C02560	S16257		0.0002	1.3656			0	level4					NULL	other
0.688_334.9	1.0595	0.6472		C5 H2 Cl N O12 S	334.9	0.688								1	level5						other
0.692_228.1111	1.2405	0.5469	Tetraacetylethylenediamine	C10 H16 N2 O4	228.1111	0.692		S59725	HMDB0040573	0.0001	0.3116			1	level4	Organic acids and derivatives	Carboxylic acids and derivatives	Carboxylic acid derivatives	Organic acids		other
0.695_144.0899	1.2385	0.3782	4-amino-1-piperidinecarboxylic acid	C6 H12 N2 O2	144.0899	0.695	C16837	S392950	HMDB0060385	0	-0.0103			0	level4	Organoheterocyclic compounds	Piperidines	Piperidinecarboxylic acids and derivatives	Piperidinecarboxylic acids and derivatives	NULL	other
0.702_138.043	0.8943	0.3885	Urocanic acid	C6 H6 N2 O2	138.043	0.702	C00785	MReference-770	HMDB0000301	0	0.3485	36.3	48.6	2	level3	Organoheterocyclic compounds	Azoles	Imidazoles	Imidazoles	map00340 Histidine metabolism; map01100 Metabolic pathways;	other
0.708_124.0637	1.1505	0.6062	3183	C6 H8 N2 O	124.0637	0.708		S16906	HMDB0031852	0	0.3742			2	level4	Organoheterocyclic compounds	Diazines	Pyrazines	Pyrazines		other
0.711_200.164	1.6996	0.3206		C9 H20 N4 O	200.164	0.711								0	level5						other
0.712_117.0458	0.9398	0.2649		C3 H8 N3 P	117.0458	0.712								1	level5						other
0.717_797.4109	0.7135	0.3483			797.4109	0.717								0	level5						other
0.718_387.705	0.8352	0.7115			387.705	0.718								0	level5						other
0.72_82.0143	0.7648	0.8341			82.0143	0.72								31	level5						other
0.726_119.0582	1.4609	0.4068	L-allo-threonine	C4 H9 N O3	119.0582	0.726	C05519	BGI339		-0.0001	-0.7252	84.4	79.2	0	level1					map00260 Glycine, serine and threonine metabolism; map01100 Metabolic pathways;	other
0.753_161.043	1.0649	0.6451		C4 H7 N3 O4	161.043	0.753								0	level5						other
0.763_173.0066	0.4386	0.4121		C6 H8 N O P S	173.0066	0.763								8	level5						other
0.765_230.9652	0.4383	0.411		C2 H6 Cl N5 O2 S2	230.9652	0.765								1	level5						other
0.766_143.0475	0.0875	0.4698	[similar to: dl-stachydrine; δmass: -0.0471 da]		143.0475	0.766								0	level5						other
0.77_203.0558	1.182	0.4626	[similar to: acetyl-l-carnitine; δ mass: -0.0599 da]		203.0558	0.77								0	level5						other
0.794_149.0511	1.6249	0.2962	L-methionine	C5 H11 N O2 S	149.0511	0.794	C00073	BGI369	HMDB0000696	0.0001	0.6157	92.3	89.1	0	level1	Peptides	Amino acids	Common amino acids [Fig]	Amino acids	map00270 Cysteine and methionine metabolism; map00970 Aminoacyl-tRNA biosynthesis; map01100 Metabolic pathways; map01210 2-Oxocarboxylic acid metabolism; map01230 Biosynthesis of amino acids; map01523 Antifolate resistance; map04974 Protein digestion and absorption; map04978 Mineral absorption; map05230 Central carbon metabolism in [illegible]	other
0.852_147.0355	1.4029	0.3828	3-thiomorpholinecarboxylic acid	C5 H9 N O2 S	147.0355	0.852	C03901	S389150	HMDB0059611	0.0001	0.703			0	level4	Organic acids and derivatives	Carboxylic acids and derivatives	Amino acids, peptides, and analogues	Amino acids, peptides, and analogues	NULL	other
0.853_156.0035	1.2189	0.394			156.0035	0.853								0	level5						other
1.164_128.0111	1.2984	0.4137	5-hydroxy-2-furoic acid	C5 H4 O4	128.0111	1.164		S3373337	HMDB0059784	0.0001	0.7958			0	level4	Organoheterocyclic compounds	Furans	Furoic acid and derivatives	Furoic acid and derivatives		other
1.164_156.006	1.2872	0.4428	2,5-furandicarboxylic acid	C6 H4 O5	156.006	1.164	C20450	S69178	HMDB0004812	0.0001	0.6775			0	level4	Organoheterocyclic compounds	Furans	Furoic acid and derivatives	Furoic acid and derivatives		other
1.168_146.0217	1.2315	0.4879	Alpha-ketoglutaric acid	C5 H6 O5	146.0217	1.168	C00026	S50	HMDB0000208	0.0002	1.3851			0	level4	Organic acids and derivatives	Keto acids and derivatives	Gamma-keto acids and derivatives	Organic acids	map00020 Citrate cycle (TCA cycle); map00040 Pentose and glucuronate interconversions; map00053 Ascorbate and aldarate metabolism; map00220 Arginine biosynthesis; map00250 Alanine, aspartate and glutamate metabolism; map00310 Lysine degradation; map00340 Histidine metabolism; map00430 Taurine and hypotaurine metabolism; map00471 D-[illegible]	other
1.168_174.0166	1.2556	0.4633	Trans-aconitic acid	C6 H6 O6	174.0166	1.168	C02341	MReference-350	HMDB0000958	0.0002	0.9091		73.9	0	level2	Organic acids and derivatives	Carboxylic acids and derivatives	Tricarboxylic acids and derivatives	Organic acids	map01100 Metabolic pathways;	other
1.169_209.0537	1.0589	0.6075		C6 H11 N O7	209.0537	1.169								0	level5						other
1.17_137.9954	1.318	0.4189		C6 H2 O4	137.9954	1.17								8	level5						other
1.171_436.9659	1.1171	0.4576			436.9659	1.171								9	level5						other
1.175_392.9759	1.2215	0.4091		C12 H4 N5 O9 P	392.9759	1.175								17	level5						other

1.267_164.0475	1.3941	0.2072	2-hydroxycinnamic acid	C9 H8 O3	164.0475	1.267	C01772	MReference-168	HMDB0002641	0.0001	0.7289	63.7	89.6	3	level2	Phenylpropanoids and polyketides	Cinnamic acids and derivatives	Hydroxycinnamic acids and derivatives	Polyketides[PK]	map00360 Phenylalanine metabolism; map01100 Metabolic pathways;	other
1.467_261.0307	0.2844	0.3866	2-methoxyacetaminophen sulfate	C9 H11 N O6 S	261.0307	1.467		S34448633	HMDB0062550	-0.0001	-0.195			0	level4	Organic acids and derivatives	Organic sulfuric acids and derivatives	Arylsulfates	Organic acids		other
1.475_248.0467	0.6952	0.4528	Nimazone	C11 H9 Cl N4 O	248.0467	1.475		S16736691		0.0002	0.87			0	level4						other
1.547_270.0852	4.5458	0.275	1,4-dihydro-4-imino-1-î²-d-ribofuranosyl-3-pyridinecarboxylic acid	C11 H14 N2 O6	270.0852	1.547	C19977	S10446255		0	-0.0922			6	level4	Fungal toxins	Mushroom toxins	Others	Fungal toxins	NULL	other
1.563_131.0765	1.4558	0.4303	2-propylthiazolidine	C6 H13 N S	131.0765	1.563		S129544	HMDB0040058	-0.0004	-2.7888			57	level4	Organoheterocyclic compounds	Azolidines	Thiazolidines	Thiazolidines		other
1.735_134.0704	0.698	0.2115			134.0704	1.735								0	level5						other
2.403_162.0794	0.4968	0.4687	1-ebio	C9 H10 N2 O	162.0794	2.403	C13840	S74291		0.0001	0.81			0	level4					NULL	other
2.494_182.1057	1.168	0.5533		C9 H14 N2 O2	182.1057	2.494								0	level5						other
2.505_191.0695	0.2659	0.3763	Carbendazim	C9 H9 N3 O2	191.0695	2.505	C10897	MReference-2868	HMDB0031769	0	0.0722		38	0	level3	Organoheterocyclic compounds	Benzimidazoles	2-benzimidazolylcarbamic acid esters	Imidazole and derivatives	NULL	other
2.539_432.1861	1.8381	0.2543		C17 H28 N4 O9	432.1861	2.539								1	level5						other
2.606_119.0898	1.5182	0.3522	[similar to: microcystin lf; δmass: -866.4262 da]		119.0898	2.606								0	level5						other
2.618_165.1364	0.6297	0.1914	[similar to: l-phenylalanine; δmass: 0.0574 da]		165.1364	2.618								1	level5						other
2.636_102.047	0.8207	0.4918		C8 H6	102.047	2.636								0	level5						other
2.639_156.0794	0.8336	0.5498	N-acetyl-l-leucine	C8 H15 N O3	156.0794	2.639						23.9		0	level5						other
2.667_165.1351	0.9641	0.7482	[similar to: l-phenylalanine; δmass: 0.0561 da]		165.1351	2.667								0	level5						other
2.678_231.1371	2.0894	0.5125	Aminophenazone	C13 H17 N3 O	231.1371	2.678	C07539	S5787	HMDB0015493	0	-0.1165			0	level4	Organoheterocyclic compounds	Azoles	Pyrazoles	Pyrazoles	NULL	other
2.708_210.1005	0.8516	0.6201	Aprobarbital	C10 H14 N2 O3	210.1005	2.708	C07826	S6221	HMDB0015441	0.0001	0.4809			0	level4	Organoheterocyclic compounds	Diazines	Pyrimidines and pyrimidine derivatives	Pyrimidines and pyrimidine derivatives	NULL	other
2.759_302.0638	1.2543	0.357	Pyrogallol-2-o-glucuronide	C12 H14 O9	302.0638	2.759		S30778511	HMDB0060017	0	0.1155			0	level4	Organic oxygen compounds	Organooxygen compounds	Carbohydrates and carbohydrate conjugates	Carbohydrates		other
2.849_120.0212	0.7859	0.8933		C7 H4 O2	120.0212	2.849						44.1		0	level5						other
2.938_194.0693	0.9774	0.5311	Aminohippuric acid	C9 H10 N2 O3	194.0693	2.938		S2063	HMDB0001867	0.0002	1.0335			0	level4	Benzenoids	Benzene and substituted derivatives	Benzoic acids and derivatives	Benzene and derivatives		other
3.019_219.1108	0.7619	0.7968	Pantothenic acid	C9 H17 N O5	219.1108	3.019	C00864	BGI462	HMDB0000210	0.0001	0.4707	85.2	33.8	0	level1	Vitamins and Cofactors	Vitamins	Water-soluble vitamins [Fig]	Vitamins	map00410 beta-Alanine metabolism; map00770 Pantothenate and CoA biosynthesis; map01100 Metabolic pathways; map04977 Vitamin digestion and absorption;	other
3.034_162.0894	0.8578	0.3846	Cymarose	C7 H14 O4	162.0894	3.034	C08234	S390151		0.0001	0.9248			0	level4					NULL	other
3.087_283.1995	0.8316	0.287		C13 H25 N5 O2	283.1995	3.087								0	level5						other
3.108_236.1161	1.0772	0.3986	Hexobarbital	C12 H16 N2 O3	236.1161	3.108	C11723	S3482	HMDB0015444	0	-0.0068			0	level4	Organoheterocyclic compounds	Diazines	Pyrimidines and pyrimidine derivatives	Pyrimidines and pyrimidine derivatives	NULL	other
3.146_174.1158	1.3637	0.3016	N-methyltryptamine	C11 H14 N2	174.1158	3.146	C06213	MReference-524	HMDB0004370	0.0001	0.7407	17.1	32.9	0	level3	Organoheterocyclic compounds	Indoles and derivatives	Tryptamines and derivatives	Amines and derivatives	map00380 Tryptophan metabolism; map01100 Metabolic pathways;	other
3.147_254.1267	1.2185	0.3051	Midodrine	C12 H18 N2 O4	254.1267	3.147	C07890	S4050	HMDB0014356	0	0.054			0	level4	Benzenoids	Benzene and substituted derivatives	Methoxybenzenes	Benzene and derivatives	NULL	other
3.194_155.0584	0.1311	0.4267	(2s,3s)-2,3-dihydro-3-hydroxyanthranilic acid zwitterion	C7 H9 N O3	155.0584	3.194	C19830	S395510		0.0002	1.1908			0	level4					map01100 Metabolic pathways;	other
3.211_209.203	0.7586	0.8582			209.203	3.211								0	level5						other
3.214_192.1661	0.5566	0.8943			192.1661	3.214								0	level5						other
3.237_278.0725	1.5366	0.5253		C13 H14 N2 O3 S	278.0725	3.237								0	level5						other
3.237_322.153	1.5401	0.5104	Etifenin	C16 H22 N2 O5	322.153	3.237		S148943		0.0001	0.3967			0	level4						other
3.239_366.1429	1.7218	0.3483		C17 H22 N2 O7	366.1429	3.239								15	level5						other
3.249_192.0951	0.2595	0.8702			192.0951	3.249								0	level5						other
3.25_186.9943	1.2623	0.9541		C8 H2 N3 O P	186.9943	3.25								0	level5						other
3.274_258.1943	1.5185	0.277		C13 H26 N2 O3	258.1943	3.274								0	level5						other
3.306_255.1683	0.6596	0.1631		C11 H21 N5 O2	255.1683	3.306								2	level5						other
3.342_99.1049	1.3866	0.3242	Cyclohexylamine	C6 H13 N	99.1049	3.342	C00571	S7677	HMDB0031404	0.0001	1.0301			0	level4	Organic nitrogen compounds	Organonitrogen compounds	Cyclohexylamines	Amines and derivatives		other
3.406_218.0693	0.663	0.5269	N-(1h-indol-3-ylcarbonyl)glycine	C11 H10 N2 O3	218.0693	3.406		S23994542	HMDB0134937	0.0001	0.6873			0	level4	Organic acids and derivatives	Carboxylic acids and derivatives	Amino acids, peptides, and analogues	Amino acids, peptides, and analogues		other
3.428_134.0481	1.2201	0.4539		C7 H6 N2 O	134.0481	3.428								0	level5						other
3.464_215.1524	1.2324	0.3516	11343172	C11 H21 N O3	215.1524	3.464	C18830	S94028		0.0002	0.9395			6	level4					NULL	other
3.471_230.157	1.8609	0.3974		C10 H22 N4 S	230.157	3.471								0	level5						other

3.471_98.0845	1.8185	0.3842	2,4-dimethyl-2-imidazoline	C5 H10 N2	98.0845	3.471		S92597	HMDB0037787	0.0001	1.1655			15	level4	Organoheterocyclic compounds	Imidolactams	null	Imidolactams		other
3.593_218.1056	0.1206	0.277	N-acetylserotonin	C12 H14 N2 O2	218.1056	3.593	C00978	BGI426	HMDB0001238	0.0001	0.4471	91.3	90.5	4	level1	Organoheterocyclic compounds	Indoles and derivatives	Hydroxyindoles	Indole and derivatives	map00380 Tryptophan metabolism; map01100 Metabolic pathways;	other
3.652_179.0949	1.4794	0.4267	Phenacetin	C10 H13 N O2	179.0949	3.652	C07591	S4590		0.0002	1.2388			0	level4					NULL	other
3.68_265.1314	1.4246	0.3455	N-(2,6-dimethylphenyl)-n-(methoxyacetyl) alanine	C14 H19 N O4	265.1314	3.68		MReference-3120		0	0.0806		70.3	1	level2						other
3.731_450.115	1.3279	0.5266	Astilbin	C21 H22 O11	450.115	3.731	C17449	S106533	HMDB0033850	-0.0012	-2.717			0	level4	Phenylpropanoids and polyketides	Flavonoids	Flavonoid glycosides	Flavonoids	NULL	other
3.733_344.2239	1.1731	0.9486			344.2239	3.733								0	level5						other
3.742_240.1111	1.083	0.5001	(s)-2-hydrazino-3-(4-hydroxy-3-methoxyphenyl)-2-methylpropionic acid	C11 H16 N2 O4	240.1111	3.742		S8374147	HMDB0142637	0	0.2016			0	level4	Phenylpropanoids and polyketides	Phenylpropanoic acids	null	Phenylpropanoic acids		other
3.767_216.0899	1.0928	0.4213	1,2,3,4-tetrahydro-î²-carboline-3-carboxylic acid	C12 H12 N2 O2	216.0899	3.767		S88749	HMDB0035665	0.0001	0.3043			0	level4	Organoheterocyclic compounds	Indoles and derivatives	Pyridoindoles	Indole and derivatives		other
3.778_170.9992	0.5685	0.4938		C6 H5 N O3 S	170.9992	3.778								0	level5						other
3.798_279.1471	0.1795	0.3355	Metalaxyl	C15 H21 N O4	279.1471	3.798	C10947	S38839	HMDB0031802	0.0001	0.3019			0	level4	Organic acids and derivatives	Carboxylic acids and derivatives	Amino acids, peptides, and analogues	Amino acids, peptides, and analogues	NULL	other
3.834_238.1417	1.0955	0.2821		C10 H22 O6	238.1417	3.834								0	level5						other
3.835_313.209	1.03	0.6499		C17 H31 N O2 S	313.209	3.835								0	level5						other
3.904_371.2518	0.7571	0.2301		C17 H33 N5 O4	371.2518	3.904								0	level5						other
3.943_304.1423	1.2364	0.5287		C16 H20 N2 O4	304.1423	3.943								0	level5						other
4.018_808.362	1.4845	0.3889		C34 H56 N4 O18	808.362	4.018								0	level5						other
4.062_311.1944	1.1628	0.4308		C14 H25 N5 O3	311.1944	4.062								0	level5						other
4.078_751.3401	2.0751	0.2235		C31 H54 N5 O14 P	751.3401	4.078								0	level5						other
4.118_189.1367	1.5057	0.2861		C9 H19 N O3	189.1367	4.118								0	level5						other
4.132_262.0954	1.3324	0.4062	(5-benzyl-3,6-dioxo-2-piperazinyl)acetic acid	C13 H14 N2 O4	262.0954	4.132		S106716	HMDB0031360	0.0001	0.2711			0	level4	Organic acids and derivatives	Carboxylic acids and derivatives	Amino acids, peptides, and analogues	Amino acids, peptides, and analogues		other
4.16_387.2468	0.7322	0.1638		C17 H33 N5 O5	387.2468	4.16								0	level5						other
4.16_392.2019	0.7726	0.2462	(2s)-2-[(1r,3as,4e,7ar)-4-{[(6s)-6-hydroxy-2,2-dioxido-1,3,4,5,6,7-hexahydro-2-benzothiophen-1-yl]methylene}-7a-methyloctahydro-1h-inden-1-yl]propanal	C22 H32 O4 S	392.2019	4.16		S7826208		-0.0002	-0.5829			0	level4						other
4.161_193.074	0.4211	0.3191	Phenylacetylglycine	C10 H11 N O3	193.074	4.161	C05598	MReference-540	HMDB0000821	0.0002	0.8278		89.6	1	level2	Organic acids and derivatives	Carboxylic acids and derivatives	Amino acids, peptides, and analogues	Amino acids, peptides, and analogues	map00360 Phenylalanine metabolism;	other
4.162_90.0471	0.41	0.2983		C7 H6	90.0471	4.162								0	level5						other
4.286_245.0689	1.885	0.2492	Haplopine	C13 H11 N O4	245.0689	4.286	C10694	S10254899		0.0001	0.3356			12	level4	Alkaloids	Alkaloids derived from tryptophan and anthranilic acid	Quinoline alkaloids	Alkaloids	NULL	other
4.288_278.1268	1.5914	0.2579	7098783	C14 H18 N2 O4	278.1268	4.288	C18753	S48518		0.0001	0.3599			0	level4					NULL	other
4.313_229.168	1.5131	0.2746	1872050	C12 H23 N O3	229.168	4.313		S1361783	HMDB0013267	0.0002	1.0444			0	level4	Organic acids and derivatives	Carboxylic acids and derivatives	Amino acids, peptides, and analogues	Amino acids, peptides, and analogues		other
4.319_157.0377	1.2029	0.4082	Furaspor	C6 H7 N O4	157.0377	4.319	C14293	S10985		0.0002	1.073	95.6		0	level4					NULL	other
4.319_250.0954	1.2282	0.4192		C12 H14 N2 O4	250.0954	4.319								0	level5						other
4.321_190.0744	1.2893	0.3583	6-hydroxy-1h-indole-3-acetamide	C10 H10 N2 O2	190.0744	4.321		S252618	HMDB0031173	0.0002	0.937	91.2		4	level4	Organoheterocyclic compounds	Indoles and derivatives	Hydroxyindoles	Indole and derivatives		other
4.322_87.0321	1.1264	0.4189	Oxazolidinone	C3 H5 N O2	87.0321	4.322	C06695	S66579		0.0001	1.334			0	level4	Antibiotics	Others [Fig]	Cyclic lactones	Antibiotics	NULL	other
4.323_173.0479	1.2902	0.3578	2-quinolinecarboxylic acid	C10 H7 N O2	173.0479	4.323	C06325	BGI77	HMDB0000842	0.0002	1.022	90.7		0	level1	Organoheterocyclic compounds	Quinolines and derivatives	Quinoline carboxylic acids	Quinoline carboxylic acids	NULL	other
4.324_135.0685	0.7328	0.6361	Acetanilide	C8 H9 N O	135.0685	4.324	C07565	S880	HMDB0001250	0.0001	0.8526			0	level4	Benzenoids	Benzene and substituted derivatives	null	Benzene and derivatives	NULL	other
4.352_232.0849	1.0912	0.6114	Phenobarbital	C12 H12 N2 O3	232.0849	4.352	C07434	S4599	HMDB0015305	0.0001	0.3046			12	level4	Organoheterocyclic compounds	Diazines	Pyrimidines and pyrimidine derivatives	Pyrimidines and pyrimidine derivatives	NULL	other
4.352_430.0892	1.3109	0.3939	4-(7-hydroxy-4-oxo-4h-chromen-3-yl)phenyl beta-d-glucopyranosiduronic acid	C21 H18 O10	430.0892	4.352		S30777616	HMDB0041717	-0.0007	-1.7347			0	level4	Organic acids and derivatives	Organic sulfuric acids and derivatives	Arylsulfates	Organic acids		other
4.369_414.2469	0.7193	0.1825		C22 H40 O3 P2	414.2469	4.369								0	level5						other
4.437_341.2049	1.0851	0.4167		C15 H27 N5 O4	341.2049	4.437								0	level5						other
4.456_104.0263	1.4796	0.4403		C7 H4 O	104.0263	4.456						64.1		1	level5						other
4.463_218.1056	1.9811	0.249	Primidone	C12 H14 N2 O2	218.1056	4.463	C07371	S4740	HMDB0014932	0.0001	0.4662			0	level4	Organoheterocyclic compounds	Diazines	Pyrimidines and pyrimidine derivatives	Pyrimidines and pyrimidine derivatives	NULL	other
4.537_560.2119	0.9164	0.4437		C18 H38 N6 O10 P2	560.2119	4.537								0	level5						other

4.559_294.1499	1.1705	0.3399		C14 H30 S3	294.1499	4.559								2	level5						other
4.613_220.11	0.754	0.4371	Precocene ii	C13 H16 O3	220.11	4.613	C09018	S12046		0.0001	0.4028			0	level4					NULL	other
4.615_301.1162	0.4334	0.4484		C13 H19 N O7	301.1162	4.615								0	level5						other
4.62_634.2966	1.5118	0.2425		C22 H47 N6 O13 P	634.2966	4.62								0	level5						other
4.656_119.0736	1.0806	0.7182		C8 H9 N	119.0736	4.656								1	level5						other
4.725_599.2828	0.888	0.3594		C23 H45 N5 O11 S	599.2828	4.725								6	level5						other
4.735_173.1054	1.4448	0.3715	N-acetyl-l-leucine	C8 H15 N O3	173.1054	4.735	C02710	S64075	HMDB0011756	0.0002	0.9653			0	level4	Organic acids and derivatives	Carboxylic acids and derivatives	Amino acids, peptides, and analogues	Amino acids, peptides, and analogues	NULL	other
4.766_414.2468	1.0832	0.5449		C22 H40 O3 P2	414.2468	4.766								0	level5						other
4.872_505.3084	1.1768	0.5693		C24 H48 N3 O4 P S	505.3084	4.872								0	level5						other
4.957_110.0732	1.7068	0.2893	Trans,trans-2,4-heptadienal	C7 H10 O	110.0732	4.957		MReference-3242		0.0001	0.6705		56.1	0	level3						other
4.957_138.0682	1.148	0.4716	3-acetyl-2,5-dimethylfuran	C8 H10 O2	138.0682	4.957		MReference-3313	HMDB0029563	0.0001	0.8652		66.5	0	level2	Organic oxygen compounds	Organooxygen compounds	Carbonyl compounds	Carbonyl compounds		other
4.984_266.1267	1.5308	0.287	Threonylphenylalanine	C13 H18 N2 O4	266.1267	4.984		S3313819	HMDB0029068	0	0.1073			0	level4	Organic acids and derivatives	Carboxylic acids and derivatives	Amino acids, peptides, and analogues	Amino acids, peptides, and analogues		other
5.042_224.1525	2.0305	0.2353	Aspergillic acid	C12 H20 N2 O2	224.1525	5.042	C10571	S9853		0	0.1589			0	level4					NULL	other
5.05_177.0791	1.4654	0.4806	5-hydroxytryptophol	C10 H11 N O2	177.0791	5.05		S8708	HMDB0001855	0.0001	0.6078			3	level4	Organoheterocyclic compounds	Indoles and derivatives	Hydroxyindoles	Indole and derivatives		other
5.062_276.111	1.2972	0.4382	Oxagrelate	C14 H16 N2 O4	276.111	5.062		S38263		0	-0.1405			0	level4						other
5.078_560.3046	0.9403	0.5786		C24 H48 O14	560.3046	5.078								1	level5						other
5.083_295.1444	0.9623	0.8865		C12 H25 N O5 S	295.1444	5.083								0	level5						other
5.1_223.0482	0.6577	0.4365	2-(carboxyacetamido)benzoic acid	C10 H9 N O5	223.0482	5.1	C03147	S388954	HMDB0039495	0.0001	0.6273			3	level4	Benzenoids	Benzene and substituted derivatives	Benzoic acids and derivatives	Benzene and derivatives	NULL	other
5.117_192.0425	0.8119	0.7777	Scopoletin	C10 H8 O4	192.0425	5.117	C01752	S4444113	HMDB0034344	0.0002	1.0851			7	level4	Phenylpropanoids and polyketides	Coumarins and derivatives	Hydroxycoumarins	Coumarins and derivatives		other
5.122_238.1318	1.2838	0.3229	Seconal	C12 H18 N2 O3	238.1318	5.122		S5005	HMDB0014562	0.0001	0.3991			0	level4	Organoheterocyclic compounds	Diazines	Pyrimidines and pyrimidine derivatives	Pyrimidines and pyrimidine derivatives		other
5.129_628.3436	0.7345	0.5235		C30 H55 N4 O4 P3	628.3436	5.129								1	level5						other
5.168_237.1002	1.5513	0.3121	Ethopabate	C12 H15 N O4	237.1002	5.168		S5812		0.0001	0.2535			3	level4						other
5.183_731.362	0.7859	0.2188		C29 H57 N5 O14 S	731.362	5.183								2	level5						other
5.207_242.0943	1.3125	0.3463	(r)-equol	C15 H14 O3	242.0943	5.207		MReference-5829		0	0.0258		90.6	2	level2						other
5.208_435.153	1.3195	0.3505		C21 H25 N O9	435.153	5.208								2	level5						other
5.209_502.2994	0.6668	0.4547	[similar to: (+/-)5(6)-dihet; δmass: 164.0537 da]		502.2994	5.209								1	level5						other
5.212_382.1052	1.1135	0.4547	Mollicellin b	C21 H18 O7	382.1052	5.212		S134711	HMDB0033340	-0.0001	-0.1887			1	level4	Organoheterocyclic compounds	Benzopyrans	1-benzopyrans	1-benzopyrans		other
5.216_314.1488	1.0719	0.3065		C12 H27 O7 P	314.1488	5.216								0	level5						other
5.238_280.1099	0.8689	0.9925	Phenprocoumon	C18 H16 O3	280.1099	5.238		S10441592	HMDB0015081	0	-0.0454		41.3	0	level4	Phenylpropanoids and polyketides	Coumarins and derivatives	Hydroxycoumarins	Coumarins and derivatives		other
5.302_505.2921	1.1213	0.3511		C22 H43 N5 O6 S	505.2921	5.302								0	level5						other
5.339_634.378	1.0792	0.4647		C28 H58 O15	634.378	5.339								0	level5						other
5.441_591.347	0.8936	0.4578		C24 H54 N3 O11 P	591.347	5.441								0	level5						other
5.448_346.1916	0.9395	0.5696		C16 H30 N2 O4 S	346.1916	5.448								0	level5						other
5.448_502.2994	1.1504	0.5908		C25 H47 N2 O4 P S	502.2994	5.448								1	level5						other
5.461_485.2841	1.3495	0.3407		C21 H43 N O11	485.2841	5.461								0	level5						other
5.462_502.313	1.5331	0.2376		C20 H47 N4 O8 P	502.313	5.462								0	level5						other
5.477_553.2772	1.7827	0.4609	7-benzyl-4-[(2-hydroxy-4-methylpentanoyl)amino]-3-isopropyl-5,8-dioxo-2-oxa-6,9-diazabicyclo[10.2.2]hexadeca-1(14),12,15-triene-10-carboxylic acid	C30 H39 N3 O7	553.2772	5.477		S35013636	HMDB0033599	-0.0016	-2.9003			0	level4	Organic acids and derivatives	Carboxylic acids and derivatives	Amino acids, peptides, and analogues	Amino acids, peptides, and analogues		other
5.486_240.1362	0.2713	0.2122		C13 H20 O4	240.1362	5.486								0	level5						other
5.554_392.257	0.9645	0.823	5722	C23 H36 O5	392.257	5.554		S4942852		0.0008	1.9655			0	level4						other
5.555_590.3518	1.3663	0.2788		C37 H51 O4 P	590.3518	5.555								31	level5						other
5.595_214.1206	2.8647	0.3781	5-hexyl-2-oxotetrahydro-3-furancarboxylic acid	C11 H18 O4	214.1206	5.595		S8551389	HMDB0030984	0.0001	0.4593			1	level4	Organoheterocyclic compounds	Lactones	Gamma butyrolactones	Gamma butyrolactones		other
5.605_242.0804	0.8255	0.4454	Lumichrome	C12 H10 N4 O2	242.0804	5.605	C01727	S4483963		0	0.1365			11	level4					map00740 Riboflavin metabolism;	other
5.631_275.1634	1.3451	0.3334	(-)-physostigmine	C15 H21 N3 O2	275.1634	5.631	C06535	S5763	HMDB0015116	0	0.0878			11	level4	Organoheterocyclic compounds	Indoles and derivatives	Pyrroloindoles	Indole and derivatives		other
5.653_546.3254	1.394	0.0836		C25 H46 N4 O9	546.3254	5.653								0	level5						other
5.687_82.0783	1.597	0.3164	3-methylcyclopentene	C6 H10	82.0783	5.687		S13625	HMDB0031544	0.0001	0.9768			0	level4	Hydrocarbons	Unsaturated hydrocarbons	Olefins	Olefins		other
5.688_124.0889	1.3868	0.3953	3721	C8 H12 O	124.0889	5.688		S4446450	HMDB0031686	0.0001	0.9809			44	level4	Organic oxygen compounds	Organooxygen compounds	Carbonyl compounds	Carbonyl compounds		other
5.688_188.105	1.4174	0.3863	Azelaic acid	C9 H16 O4	188.105	5.688	C08261	BGI162	HMDB0000784	0.0002	0.8351	92.4		14	level1	FA Fatty acyls	FA01 Fatty Acids and Conjugates	FA0117 Dicarboxylic acids	Fatty acyls[FA]	NULL	other
5.688_273.0426	1.4404	0.3585		C7 H16 N O6 P S	273.0426	5.688								0	level5						other
5.688_432.1295	1.6721	0.3195		C19 H29 Cl N2 O3 S2	432.1295	5.688								7	level5						other
5.689_142.0995	1.4674	0.3689	2347	C8 H14 O2	142.0995	5.689		S20080	HMDB0031403	0.0002	1.133			0	level4	Organic acids and derivatives	Carboxylic acids and derivatives	Carboxylic acids	Organic acids		other

5.689_152.0839	1.4317	0.3764	2128	C9 H12 O2	152.0839	5.689		S56163	HMDB0041326	0.0001	0.8008			0	level4	Benzenoids	Benzene and substituted derivatives	Benzylethers	Benzene and derivatives		other
5.689_194.113	1.3681	0.3861		C12 H18 S	194.113	5.689								0	level5						other
5.689_260.0348	1.3732	0.3877	4-allyl-2-hydroxy-6-methoxyphenyl hydrogen sulfate	C10 H12 O6 S	260.0348	5.689		S74853778	HMDB0135261	-0.0007	-2.6361			1	level4	Organic acids and derivatives	Organic sulfuric acids and derivatives	Arylsulfates	Organic acids		other
5.689_96.094	1.389	0.3981		C7 H12	96.094	5.689								0	level5						other
5.69_244.0624	1.4207	0.3749	Carnidazole	C8 H12 N4 O3 S	244.0624	5.69		S2297829		-0.0006	-2.3857			4	level4						other
5.736_827.5106	1.1074	0.5122		C40 H79 N O12 P2	827.5106	5.736								0	level5						other
5.738_427.2231	1.1139	0.5196		C18 H37 N O8 S	427.2231	5.738								0	level5						other
5.817_434.2444	1.0403	0.6706	1-linoleyl-sn-glycerol 3-phosphate	C21 H39 O7 P	434.2444	5.817		S24766514		0.001	2.3656			0	level4						other
5.873_290.079	0.7485	0.8467	Cianidanol	C15 H14 O6	290.079	5.873	C17590	S1166	HMDB0127721	0	-0.0427			0	level4	Phenylpropanoids and polyketides	Flavonoids	Flavans	Flavonoids	NULL	other
6.003_208.0036	1.1848	0.654		C8 H5 N2 O3 P	208.0036	6.003								0	level5						other
6.004_187.0635	0.8302	0.4903	Trans-3-indoleacrylic acid	C11 H9 N O2	187.0635	6.004	C21283	MReference-928	HMDB0000734	0.0002	0.9152		60.7	0	level2	Organoheterocyclic compounds	Indoles and derivatives	Indoles	Indoles	NULL	other
6.016_136.089	0.7778	0.7807	4329	C9 H12 O	136.089	6.016		S10248	HMDB0032544	0.0002	1.182			0	level4	Benzenoids	Phenols	Cresols	Phenols and derivatives		other
6.114_234.1257	0.7072	0.606	Stiripentol	C14 H18 O3	234.1257	6.114		S4470940		0.0001	0.3961	32.4		0	level4						other
6.152_220.0737	0.9485	0.5193	Eugenitin	C12 H12 O4	220.0737	6.152		S2340764	HMDB0029467	0.0001	0.5648			3	level4	Organoheterocyclic compounds	Benzopyrans	1-benzopyrans	1-benzopyrans		other
6.209_172.0036	0.9796	0.8104		C5 H5 N2 O3 P	172.0036	6.209								6	level5						other
6.234_250.0842	1.0339	0.289	Citrinin	C13 H14 O5	250.0842	6.234	C16765	MReference-1774	HMDB0041857	0	0.185		51	0	level3	Organoheterocyclic compounds	Benzopyrans	null	Benzopyrans	NULL	other
6.403_421.195	0.7463	0.4131		C18 H31 N O10	421.195	6.403								0	level5						other
6.407_210.1258	0.5629	0.5794	Epi-jasmonic acid	C12 H18 O3	210.1258	6.407	C16317	S5584839		0.0002	1.042			0	level4	FA Fatty acyls	FA02 Octadecanoids	FA0202 jasmonic acids	Fatty acyls[FA]	map00592 alpha-Linolenic acid metabolism; map01100 Metabolic pathways;	other
6.408_588.3725	1.1115	0.442		C26 H57 N2 O10 P	588.3725	6.408								1	level5						other
6.442_344.1988	2.1379	0.1231	Nor-9-carboxy-δ9-thc	C21 H28 O4	344.1988	6.442		MReference-2472		0	0.0237		65.1	0	level2						other
6.584_178.0995	0.7124	0.9718	Isobutyl benzoate	C11 H14 O2	178.0995	6.584		MReference-2585	HMDB0040583	0.0002	0.8714		39.3	0	level3	Benzenoids	Benzene and substituted derivatives	Benzoic acids and derivatives	Benzene and derivatives		other
6.613_132.094	0.9361	0.4681	Tetralin	C10 H12	132.094	6.613	C14114	S8097		0.0001	1.0702			0	level4						other
6.701_671.3526	0.7981	0.2322		C33 H53 N O13	671.3526	6.701								0	level5						other
6.702_185.069	0.8094	0.2185	Eglumetad	C8 H11 N O4	185.069	6.702		S184747		0.0002	1.0382			1	level4						other
6.702_203.0796	0.8083	0.2093	N-acetyl-l-2-aminoadipic acid	C8 H13 N O5	203.0796	6.702	C12986	S392027		0.0002	0.9971	86.1		0	level4					NULL	other
6.702_221.0901	0.8262	0.3	N-acetyl-d-glucosamine	C8 H15 N O6	221.0901	6.702	C00140	BGI412	HMDB0000215	0.0001	0.5845	88.1	84.3	0	level2	Carbohydrates	Monosaccharides	Amino sugars [Fig]	Carbohydrates	map00520 Amino sugar and nucleotide sugar metabolism; map01100 Metabolic pathways; map02010 ABC transporters;	other
6.816_218.1672	0.7508	0.6271	Nootkatone	C15 H22 O	218.1672	6.816	C17914	MReference-842	HMDB0013687	0.0001	0.606		62.7	1	level2	Lipids and lipid-like molecules	Prenol lipids	Sesquiterpenoids	Terpenoids	NULL	other
6.898_346.2144	2.1272	0.7435	Corticosterone	C21 H30 O4	346.2144	6.898	C02140	MReference-354	HMDB0001547	0	0.0222		93.6	0	level1	Lipids and lipid-like molecules	Steroids and steroid derivatives	Hydroxysteroids	Steroids and derivatives	map00140 Steroid hormone biosynthesis; map01100 Metabolic pathways; map04923 Regulation of lipolysis in adipocytes; map04925 Aldosterone synthesis and secretion; map05020 Prion diseases;	other
7.023_344.21	1.2171	0.3015	Oxyphencyclimine	C20 H28 N2 O3	344.21	7.023	C07851	S4481	HMDB0014527	0	-0.1312			0	level4	Organoheterocyclic compounds	Diazines	Pyrimidines and pyrimidine derivatives	Pyrimidines and pyrimidine derivatives	NULL	other
7.059_164.1203	0.8344	0.5026	Fenipentol	C11 H16 O	164.1203	7.059		S3221		0.0002	1.0745			1	level4						other
7.06_250.1182	0.7997	0.6118		C11 H25 P3	250.1182	7.06							52.4	0	level5						other
7.061_122.1096	0.4369	0.6181	3-allylcyclohexene	C9 H14	122.1096	7.061		S123394	HMDB0061784	0.0001	0.8275			5	level4	Hydrocarbons	Unsaturated hydrocarbons	Olefins	Olefins		other
7.167_310.2145	0.8279	0.6398	13(s)-hpotre	C18 H30 O4	310.2145	7.167	C04785	S4593698		0.0001	0.2084			0	level4	FA Fatty acyls	FA01 Fatty Acids and Conjugates	FA0104 Hydroperoxy fatty acids	Fatty acyls[FA]	map00592 alpha-Linolenic acid metabolism; map01100 Metabolic pathways;	other
7.196_236.105	1.2102	0.3639	Ethylvanillin isobutyrate	C13 H16 O4	236.105	7.196		S586073	HMDB0037683	0.0002	0.7165			1	level4	Benzenoids	Phenol esters	null	Benzene and derivatives		other
7.225_334.2149	0.6076	0.1198	1886839	C20 H30 O4	334.2149	7.225	C14504	S6528		0.0005	1.4477		72.6	1	level4					NULL	other
7.253_295.1532	1.4864	0.3056	Butralin	C14 H21 N3 O4	295.1532	7.253	C18582	S33600		0	0.0078			8	level4					NULL	other
7.322_194.1309	1.2049	0.624	Sedanolide	C12 H18 O2	194.1309	7.322	C17002	MReference-6215	HMDB0034450	0.0002	1.1822		49.5	0	level3	Organoheterocyclic compounds	Isobenzofurans	null	Isobenzofurans	NULL	other
7.347_254.1518	0.2338	0.2698		C14 H22 O4	254.1518	7.347								42	level5						other
7.35_276.1337	0.1809	0.2727		C12 H16 N6 O2	276.1337	7.35								16	level5						other

7.365_254.1155	1.7157	0.2742	Hostmaniane	C13 H18 O5	254.1155	7.365		S9359446	HMDB0032796	0.0001	0.3797			0	level4	Benzenoids	Benzene and substituted derivatives	Benzoic acids and derivatives	Benzene and derivatives		other
7.436_178.1361	1.4719	0.3025	Propofol	C12 H18 O	178.1361	7.436	C07523	S4774	HMDB0014956	0.0003	1.7952			2	level4	Benzenoids	Benzene and substituted derivatives	Cumenes	Benzene and derivatives	NULL	other
7.436_224.1416	1.428	0.2998	Methyl jasmonate	C13 H20 O3	224.1416	7.436	C11512	BGI403	HMDB0036583	0.0004	1.6455	7.5		0	level3	FA Fatty acyls	FA02 Octadecanoids	FA0202 jasmonic acids	Fatty acyls[FA]	map00592 alpha-Linolenic acid metabolism;	other
7.437_264.1343	1.2681	0.3634		C9 H21 N4 O3 P	264.1343	7.437							75.2	0	level5						other
7.439_196.1468	0.8684	0.6781	Acetic acid geranyl ester	C12 H20 O2	196.1468	7.439	C09861	S1266019	HMDB0035157	0.0005	2.3532			1	level4	Terpenoids	Monoterpenoids (C10)	Linear monoterpenes	Terpenoids	NULL	other
7.483_299.1919	0.6754	0.3998		C16 H29 N O2 S	299.1919	7.483								0	level5						other
7.596_250.1205	1.4193	0.2999	Coenzyme q1	C14 H18 O4	250.1205	7.596		MReference-378	HMDB0002012	0	0.0091		58.5	0	level3	Lipids and lipid-like molecules	Prenol lipids	Quinone and hydroquinone lipids	Quinone		other
7.61_259.0718	0.904	0.4123		C10 H14 Cl N3 O3	259.0718	7.61								0	level5						other
7.622_160.089	0.8389	0.5784	2-phenyl-4-pentenal	C11 H12 O	160.089	7.622		S489034	HMDB0035207	0.0001	0.8948			1	level4	Benzenoids	Benzene and substituted derivatives	Phenylacetaldehydes	Benzene and derivatives		other
7.623_276.1726	0.8058	0.4788	Cyclandelate	C17 H24 O3	276.1726	7.623		S2790	HMDB0015586	0	0.116			0	level4	Benzenoids	Benzene and substituted derivatives	null	Benzene and derivatives		other
7.682_220.1474	0.7467	0.2569	2,6-di-tert-butyl-1,4-benzoquinone	C14 H20 O2	220.1474	7.682		MReference-607	HMDB0013817	0.0011	4.8415		48.6	0	level3	Lipids and lipid-like molecules	Prenol lipids	Monoterpenoids	Terpenoids		other
7.766_141.152	0.9237	0.8127	Isometheptene	C9 H19 N	141.152	7.766		S21106328	HMDB0015651	0.0002	1.5198			22	level4	Organic nitrogen compounds	Organonitrogen compounds	Amines	Amines		other
7.788_236.1428	0.9151	0.5022	[similar to: 12-oxo phytodienoic acid; δmass: -56.0610 da]		236.1428	7.788								18	level5						other
7.803_175.1457	1.8712	0.2463			175.1457	7.803								0	level5						other
7.803_256.1693	2.0917	0.26		C13 H25 N2 O P	256.1693	7.803								0	level5						other
7.804_192.153	1.9035	0.2706	[similar to: arachidonoyl amide; δ mass: -111.1032 da]		192.153	7.804								0	level5						other
7.804_210.1637	1.7841	0.3075	[similar to: 10-nitrooleate; δ mass: -117.0773 da]		210.1637	7.804								22	level5						other
7.804_238.1586	1.7549	0.3134	[similar to: estriol; δ mass: -50.0140 da]		238.1586	7.804								0	level5						other
7.804_312.0894	1.7933	0.2788	Quinacridone	C20 H12 N2 O2	312.0894	7.804		S13369	HMDB0034058	-0.0004	-1.4043			13	level4	Organoheterocyclic compounds	Quinolines and derivatives	Benzoquinolines	Quinone		other
7.805_224.1794	1.9327	0.2497	[similar to: (+/-)11(12)-dihet; δmass: -114.0663 da]		224.1794	7.805								0	level5						other
7.805_270.1852	1.8556	0.2799		C14 H27 N2 O P	270.1852	7.805								0	level5						other
7.814_110.1096	0.1731	0.352	1733342	C8 H14	110.1096	7.814		S12451	HMDB0061902	0.0001	0.881			0	level4	Hydrocarbons	Unsaturated hydrocarbons	Branched unsaturated hydrocarbons	Branched unsaturated hydrocarbons		other
7.817_243.2568	0.8818	0.6882		C15 H33 N O	243.2568	7.817								1	level5						other
7.83_83.953	1.5385	0.4082	Dichloromethane	C H2 Cl2	83.953	7.83	C02271	S6104	HMDB0031548	-0.0003	-4.0162			0	level4	Organohalogen compounds	Alkyl halides	Halomethanes	Halomethanes		other
7.863_272.1053	1.1568	0.3611	Deoxyshikonin	C16 H16 O4	272.1053	7.863	C18133	S89341		0.0005	1.7433			0	level4					map00130 Ubiquinone and other terpenoid-quinone biosynthesis;	other
7.871_274.1208	1.2539	0.4009	6,7-dimethoxy-3-(2-methyl-3-buten-2-yl)-2h-chromen-2-one	C16 H18 O4	274.1208	7.871		S158342	HMDB0034132	0.0003	1.0824			0	level4	Phenylpropanoids and polyketides	Coumarins and derivatives	null	Coumarins and derivatives		other
7.904_294.2195	0.7051	0.4679	13(s)-hotre	C18 H30 O3	294.2195	7.904	C16316	MReference-9864		0	-0.0266		49.7	5	level3	FA Fatty acyls	FA02 Octadecanoids	FA0200 Other Octadecanoids	Fatty acyls[FA]	map00592 alpha-Linolenic acid metabolism;	other
7.907_242.1309	0.8723	0.415	Nsc 1775	C16 H18 O2	242.1309	7.907	C14225	S59553		0.0002	0.8754			0	level4					NULL	other
7.942_244.1464	0.896	0.4809	Fenestrel	C16 H20 O2	244.1464	7.942		S22786		0.0001	0.4414			0	level4						other
7.947_296.1624	1.2242	0.4366	4-hydroxy-5-methoxy-4-[2-methyl-3-(3-methyl-2-buten-1-yl)-2-oxiranyl]-1-oxaspiro[2.5]octan-6-one	C16 H24 O5	296.1624	7.947	C09674	S251178	HMDB0038120	0	0.0756			0	level4	Organic oxygen compounds	Organooxygen compounds	Alcohols and polyols	Alcohols	NULL	other
8.071_317.1656	0.8138	0.4547	Pitrazepin	C19 H19 N5	317.1656	8.071	C13794	S128982		0.0016	4.9015			0	level4					NULL	other
8.073_437.17	0.8501	0.5147		C14 H33 Cl N3 O8 P	437.17	8.073								0	level5						other
8.074_317.0824	0.4764	0.6932		C17 H12 N5 P	317.0824	8.074								0	level5						other
8.077_417.1591	1.0423	0.8947			417.1591	8.077								0	level5						other
8.08_341.2524	0.8401	0.3898			341.2524	8.08								7	level5						other
8.082_317.0875	0.6978	0.617		C20 H15 N O S	317.0875	8.082								0	level5						other
8.098_315.3141	0.7908	0.5179		C19 H41 N O2	315.3141	8.098								0	level5						other
8.113_206.1672	1.6489	0.2858	513992	C14 H22 O	206.1672	8.113	C14205	S8483	HMDB0013825	0.0001	0.7005			0	level4	Benzenoids	Benzene and substituted derivatives	Phenylpropanes	Benzene and derivatives	NULL	other
8.113_252.1726	2.1135	0.2294	Cuauhtemone	C15 H24 O3	252.1726	8.113	C09645	S390809		0	0.1272			0	level4	Terpenoids	Sesquiterpenoids (C15)	Others	Terpenoids	NULL	other

8.113_270.1831	2.1083	0.23	3543	C15 H26 O4	270.1831	8.113		S54974	HMDB0040459	0	0.1013			18	level4	Phenylpropanoids and polyketides	Macrolides and analogues	null	Polyketides[PK]		other
8.208_422.2815	4.1548	0.8258	1-octadecylglycerone 3-phosphate	C21 H43 O6 P	422.2815	8.208		S25996866	HMDB0011142	0.0018	4.3013			17	level4	Organic oxygen compounds	Organooxygen compounds	Carbonyl compounds	Carbonyl compounds		other
8.218_315.3141	0.6684	0.3501		C19 H41 N O2	315.3141	8.218								9	level5						other
8.298_372.2664	0.1905	0.2424	(−)-nabilone	C24 H36 O3	372.2664	8.298		S4447641	HMDB0014629	0	-0.0642			0	level4	Organoheterocyclic compounds	Naphthopyrans	Naphthopyranones	Naphthopyranones		other
8.321_278.2245	0.539	0.6188	Gamma-linolenic acid	C18 H30 O2	278.2245	8.321	C06426	BGI279	HMDB0003073	-0.0001	-0.2722	87		0	level2	Lipids	Fatty acids [Fig]	Unsaturated fatty acids	Fatty acyls[FA]	map00591 Linoleic acid metabolism; map01040 Biosynthesis of unsaturated fatty acids; map01100 Metabolic pathways;	other
8.391_322.178	1.6322	0.2753	Taleranol	C18 H26 O5	322.178	8.391	C14752	S20916	HMDB0032702	-0.0001	-0.1822			0	level4	Phenylpropanoids and polyketides	Macrolides and analogues	null	Polyketides[PK]	NULL	other
8.541_442.3085	0.0313	0.2215	(1s,3r,5z,7e,9xi)-18-[(4-hydroxy-4-methyl-2-pentyn-1-yl)oxy]-20-methyl-9,10-secopregna-5,7,10-triene-1,3-diol	C28 H42 O4	442.3085	8.541		S7826625		0.0002	0.4049			0	level4						other
8.659_338.2432	0.7469	0.4747		C16 H30 N6 O2	338.2432	8.659							36.5	0	level5						other
8.71_294.2179	0.4578	0.3479		C14 H26 N6 O	294.2179	8.71								0	level5						other
8.76_597.3136	1.1088	0.6353			597.3136	8.76								0	level5						other
8.761_553.2873	1.1877	0.4392		C23 H48 N5 O4 P S2	553.2873	8.761								0	level5						other
8.768_444.3241	0.0128	0.2008	(1s,3r,5z,7e,22e)-9,10-secoergosta-5,7,10,22-tetraene-1,3,25,28-tetrol	C28 H44 O4	444.3241	8.768		S4946195		0.0001	0.278			10	level4						other
8.883_508.7609	1.0454	0.7131			508.7609	8.883								0	level5						other
9.173_517.2742	0.8573	0.4522		C25 H45 N O6 P2	517.2742	9.173								8	level5						other
9.173_519.7517	0.8361	0.3454	[similar to: dl-dipalmitoylphosphatidylcholine; δ mass: -213.8104 da]		519.7517	9.173								15	level5						other
9.179_538.1889	0.8958	0.5782		C20 H38 N6 O3 S4	538.1889	9.179								0	level5						other
9.179_885.4433	0.9829	0.9738		C47 H71 N3 O7 S3	885.4433	9.179								0	level5						other
9.185_665.3117	1.0085	0.8625		C32 H51 N5 O4 S3	665.3117	9.185								30	level5						other
9.186_621.2852	1.0264	0.8077		C30 H47 N5 O3 S3	621.2852	9.186								0	level5						other
9.186_648.2851	1.0031	0.8936		C32 H48 N4 O4 S3	648.2851	9.186								0	level5						other
9.2_649.3711	1.0481	0.6237		C32 H59 N O8 S2	649.3711	9.2								1	level5						other
9.205_620.3224	0.8958	0.4178	Terlakiren	C31 H48 N4 O7 S	620.3224	9.205		S58451		-0.002	-3.2385			34	level4						other
9.306_307.2511	1.0544	0.6891	Fingolimod	C19 H33 N O2	307.2511	9.306		S97087		0	-0.0793			0	level4						other
9.408_1016.6823	0.715	0.4163		C48 H102 N6 O10 P2 S	1016.6823	9.408								3	level5						other
9.492_524.2581	0.7956	0.5898	Dexamethasone phenylpropionate	C31 H37 F O6	524.2581	9.492		S56732		0.0007	1.311			0	level4						other
9.495_510.7584	0.819	0.378	[similar to: dl-dipalmitoylphosphatidylcholine; δ mass: -222.8038 da]		510.7584	9.495								26	level5						other
9.498_959.4628	0.8104	0.4343		C49 H77 N5 O4 S5	959.4628	9.498								5	level5						other
9.507_871.4096	0.8207	0.4985		C46 H69 N3 O5 S4	871.4096	9.507								32	level5						other
9.513_783.3568	0.8691	0.3984		C42 H61 N3 O3 S4	783.3568	9.513								2	level5						other
9.52_695.3041	0.8232	0.4361		C34 H50 Cl N3 O8 S	695.3041	9.52								3	level5						other
9.525_332.1438	0.4676	0.3564	9-fluoro-17?-oxa-dextro-homoandrosta-1,4-diene-3,11,17-trione	C19 H21 F O4	332.1438	9.525	C15387	S222020		0.0014	4.1398			0	level4					NULL	other
9.526_607.2518	0.8511	0.4197		C26 H49 N5 O S5	607.2518	9.526								3	level5						other
9.527_316.1714	0.5274	0.3067		C16 H30 O2 P2	316.1714	9.527								0	level5						other
9.606_308.1438	0.3635	0.3737		C17 H24 O3 S	308.1438	9.606								11	level5						other
9.713_324.2038	0.8192	0.2355		C19 H33 P S	324.2038	9.713								1	level5						other
9.716_356.2924	0.4116	0.2543	1-oleoyl-rac-glycerol	C21 H40 O4	356.2924	9.716		S4446588		-0.0002	-0.604			2	level4						other
0.542_108.9705	0.751	0.5342			108.9705	0.542								14	level5						other
0.542_142.9897	1.514	0.4656			142.9897	0.542								0	level5						other
0.544_124.9791	1.5217	0.4584			124.9791	0.544								1	level5						other
0.557_215.9435	1.5309	0.3881		C4 H8 Cl O2 P3	215.9435	0.557								1	level5						other
0.583_627.8502	1.2224	0.6607		C12 H3 N6 O17 P3 S	627.8502	0.583								0	level5						other
0.597_312.0284	1.1286	0.5445		C4 H12 N2 O14	312.0284	0.597								11	level5						other
0.598_258.0566	0.8817	0.241	4-(3-oxopentyl)phenyl hydrogen sulfate	C11 H14 O5 S	258.0566	0.598		S74853082	HMDB0133002	0.0004	1.6315			18	level4	Organic acids and derivatives	Organic sulfuric acids and derivatives	Arylsulfates	Organic acids		other
0.605_231.9006	0.9538	0.4944		C2 H2 O7 P2 S	231.9006	0.605								49	level5						other
0.606_299.8875	0.9344	0.3096			299.8875	0.606								0	level5						other

0.611_89.9694	0.8771	0.619			89.9694	0.611								97	level5						other
0.624_111.0796	1.0762	0.5253	Histamine	C5 H9 N3	111.0796	0.624	C00388	BGI310	HMDB0000870	0	-0.0123	93.7	92	4	level1	Peptides	Amines	Biogenic amines [Fig]	Amines	map00340 Histidine metabolism; map01100 Metabolic pathways; map04080 Neuroactive ligand-receptor interaction; map04664 Fc epsilon RI signaling pathway; map04721 Synaptic vesicle cycle; map04750 Inflammatory mediator regulation of TRP channels; map04971 Gastric acid secretion; map04974 Protein digestion and absorption; map05310 [illegible]	other
0.626_199.0334	0.9458	0.602		C5 H5 N5 O4	199.0334	0.626								26	level5						other
0.637_263.845	1.0877	0.66			263.845	0.637								2	level5						other
0.643_248.9288	0.9034	0.9805		C2 H4 Cl N3 O5 S2	248.9288	0.643								2	level5						other
0.649_142.9809	0.3842	0.4525		C2 H6 Cl N O2 S	142.9809	0.649								3	level5						other
0.653_382.1085	0.9491	0.9346		C10 H18 N6 O10	382.1085	0.653								54	level5						other
0.656_175.0958	0.8803	0.3555	L-citrulline	C6 H13 N3 O3	175.0958	0.656	C00327	BGI349	HMDB0000904	0.0001	0.7104	84.4	76.9	0	level1	Peptides	Amino acids	Other amino acids [Fig]	Amino acids	map00220 Arginine biosynthesis; map01100 Metabolic pathways; map01230 Biosynthesis of amino acids;	other
0.657_377.9183	0.9226	0.5771		C3 H8 Cl N2 O13 P S	377.9183	0.657								1	level5						other
0.66_259.0666	1.1514	0.6189	Azatepa	C8 H14 N5 O P S	259.0666	0.66		S29057		0.0009	3.405			11	level4						other
0.676_248.1483	1.241	0.8824		C11 H25 N2 P S	248.1483	0.676								1	level5						other
0.681_158.0693	0.8607	0.3925	Oxiracetam	C6 H10 N2 O3	158.0693	0.681		S4465		0.0001	0.823			0	level4						other
0.687_153.0193	1.3167	0.4471	Fosamine	C3 H8 N O4 P	153.0193	0.687	C18789	S30725		0.0002	1.3733			31	level4					NULL	other
0.687_159.0273	1.1386	0.6641		C4 H5 N3 O4	159.0273	0.687								18	level5						other
0.69_140.0586	1.3125	0.9594	Methylimidazoleacetic acid	C6 H8 N2 O2	140.0586	0.69	C05828	MReference-491	HMDB0002820	0	0.3442		36.1	0	level3	Organoheterocyclic compounds	Azoles	Imidazoles	Imidazoles	map00340 Histidine metabolism; map01100 Metabolic pathways;	other
0.69_149.0065	0.861	0.3566		C4 H8 N O P S	149.0065	0.69								2	level5						other
0.693_186.0506	1.1133	0.6342		C4 H6 N6 O3	186.0506	0.693						36		30	level5						other
0.695_159.126	0.8583	0.5982	Pregabalin	C8 H17 N O2	159.126	0.695		S4589156	HMDB0014375	0.0001	0.6328			0	level4	Organic acids and derivatives	Carboxylic acids and derivatives	Amino acids, peptides, and analogues	Amino acids, peptides, and analogues		other
0.696_73.0892	2.0226	0.8523	Diethylamine	C4 H11 N	73.0892	0.696		S7730	HMDB0041878	0.0001	0.7575			0	level4	Organic nitrogen compounds	Organonitrogen compounds	Amines	Amines		other
0.7_142.1109	2.3362	0.4851		C7 H14 N2 O	142.1109	0.7								3	level5						other
0.721_190.033	1.1601	0.6561		C6 H11 N2 O P S	190.033	0.721								0	level5						other
0.721_300.1331	0.7198	0.8461		C15 H27 P3	300.1331	0.721								17	level5						other
0.721_371.7307	1.1775	0.5064			371.7307	0.721								7	level5						other
0.722_313.772	0.9864	0.8907			313.772	0.722								9	level5						other
0.722_373.7279	0.9642	0.9399			373.7279	0.722								0	level5						other
0.724_202.1249	0.8582	0.3928	[similar to: ginsenoside rg2; δmass: -582.3724 da]		202.1249	0.724								0	level5						other
0.741_151.0244	0.8881	0.7096			151.0244	0.741								39	level5						other
0.755_71.0736	1.1317	0.4009	Pyrrolidine	C4 H9 N	71.0736	0.755		S29008	HMDB0031641	0.0001	1.718			1	level4	Organoheterocyclic compounds	Pyrrolidines	null	Pyrrolidines		other
0.781_96.0212	1.3626	0.3636	Furfural	C5 H4 O2	96.0212	0.781	C14279	S13863629	HMDB0032914	0.0001	1.2534			0	level4	Organic oxygen compounds	Organooxygen compounds	Carbonyl compounds	Carbonyl compounds	map01100 Metabolic pathways;	other
0.821_187.0281	1.058	0.6394	[similar to: nicotinic acid; δmass: 63.9960 da]		187.0281	0.821								10	level5						other
0.962_111.0434	0.6371	0.4456	Cytosine	C4 H5 N3 O	111.0434	0.962	C00380	S577	HMDB0000630	0.0001	1.1566			21	level4	Organoheterocyclic compounds	Diazines	Pyrimidines and pyrimidine derivatives	Pyrimidines and pyrimidine derivatives	map00240 Pyrimidine metabolism; map01100 Metabolic pathways;	other
1.005_191.0253	1.3955	0.3182		C6 H9 N O4 S	191.0253	1.005								5	level5						other
1.316_73.0529	0.8808	0.673	Dimethylformamide	C3 H7 N O	73.0529	1.316	C03134	S5993	HMDB0001888	0.0001	1.2929			15	level4	Organic acids and derivatives	Carboxylic acids and derivatives	Carboxylic acid derivatives	Organic acids	NULL	other

1.588_131.0948	1.0644	0.7323	L-isoleucine	C6 H13 N O2	131.0948	1.588	C00407	BGI366	HMDB0000172	0.0001	0.994	96.7	90.6	0	level1	Peptides	Amino acids	Common amino acids [Fig]	Amino acids	map00280 Valine, leucine and isoleucine degradation; map00290 Valine, leucine and isoleucine biosynthesis; map00970 Aminoacyl-tRNA biosynthesis; map01100 Metabolic pathways; map01210 2-Oxocarboxylic acid metabolism; map01230 Biosynthesis of amino acids; map02010 ABC transporters; map04974 Protein digestion and absorption; map04978 Mineral absorption; [illegible]	other
10.04_316.2377	0.6423	0.5206		C17 H28 N6	316.2377	10.04								0	level5						other
2.412_136.0719	0.6744	0.6354			136.0719	2.412								0	level5						other
2.577_102.047	0.8535	0.5908		C8 H6	102.047	2.577								2	level5						other
2.616_119.0381	1.0986	0.8992	4-aminobenzoate	C7 H7 N O2	119.0381	2.616						18.7		11	level5						other
2.638_109.0706	0.9396	0.5775			109.0706	2.638								14	level5						other
2.735_87.0144	1.2526	0.5661		C3 H5 N S	87.0144	2.735								0	level5						other
2.773_310.1165	0.9449	0.9827	Gamma-l-glutamyl-l-tyrosine	C14 H18 N2 O6	310.1165	2.773		S85142	HMDB0011741	0	0.1517			49	level4	Organic acids and derivatives	Carboxylic acids and derivatives	Amino acids, peptides, and analogues	Amino acids, peptides, and analogues		other
2.84_157.0741	1.6863	0.3564	N-acetylproline	C7 H11 N O3	157.0741	2.84		BGI621	HMDB0094701	0.0002	1.1131	29.7		0	level3	Organic acids and derivatives	Carboxylic acids and derivatives	Amino acids, peptides, and analogues	Amino acids, peptides, and analogues		other
3.035_207.101	1.0317	0.6878	Nnk	C10 H13 N3 O2	207.101	3.035	C16453	S43038	HMDB0011603	0.0002	0.9615			1	level4	Organic oxygen compounds	Organooxygen compounds	Carbonyl compounds	Carbonyl compounds	map00980 Metabolism of xenobiotics by cytochrome P450; map05204 Chemical carcinogenesis;	other
3.132_110.0369	1.4886	0.5711	Hydroquinone	C6 H6 O2	110.0369	3.132	C00530	S764	HMDB0002434	0.0001	0.7092			1	level4	Benzenoids	Phenols	Benzenediols	Phenols and derivatives	map00350 Tyrosine metabolism; map01100 Metabolic pathways;	other
3.21_192.0221	0.8098	0.8068		C5 H10 N2 O2 P2	192.0221	3.21								1	level5						other
3.242_292.0519	1.4821	0.4259		C5 H18 N4 O4 P2 S	292.0519	3.242								3	level5						other
3.247_142.0654	1.0828	0.6921		C6 H11 N2 P	142.0654	3.247								0	level5						other
3.248_220.0849	0.9483	0.8543	L-5-hydroxytryptophan	C11 H12 N2 O3	220.0849	3.248	C00643	MReference-1540	HMDB0000472	0.0001	0.3692	10.7	30.4	3	level3	Organoheterocyclic compounds	Indoles and derivatives	Tryptamines and derivatives	Amines and derivatives	map00380 Tryptophan metabolism; map01100 Metabolic pathways; map04726 Serotonergic synapse;	other
3.268_204.0112	1.4979	0.4963		C3 H8 O10	204.0112	3.268								2	level5						other
3.364_128.0475	1.4971	0.4142	Furaneol	C6 H8 O3	128.0475	3.364	C20717	S18218	HMDB0040594	0.0001	0.9105	52		0	level4	Organoheterocyclic compounds	Dihydrofurans	Furanones	Furanones	NULL	other
3.49_269.1839	1.0355	0.7107		C12 H23 N5 O2	269.1839	3.49								0	level5						other
3.611_159.0897	0.9773	0.9641	N-acetylvaline	C7 H13 N O3	159.0897	3.611		S198159	HMDB0011757	0.0001	0.8456			2	level4	Organic acids and derivatives	Carboxylic acids and derivatives	Amino acids, peptides, and analogues	Amino acids, peptides, and analogues		other
3.65_248.0797	1.2987	0.5375	Hydroxyphenobarbital	C12 H12 N2 O4	248.0797	3.65		S9402	HMDB0060537	0	0.0281			0	level4	Organoheterocyclic compounds	Diazines	Pyrimidines and pyrimidine derivatives	Pyrimidines and pyrimidine derivatives		other
3.677_226.1318	1.5749	0.4021	Pentobarbital	C11 H18 N2 O3	226.1318	3.677	C07422	S4575	HMDB0014457	0.0001	0.4687			1	level4	Organoheterocyclic compounds	Diazines	Pyrimidines and pyrimidine derivatives	Pyrimidines and pyrimidine derivatives	NULL	other
3.741_371.2518	0.8594	0.3009		C17 H33 N5 O4	371.2518	3.741								11	level5						other
3.771_304.1423	1.0286	0.8637		C16 H20 N2 O4	304.1423	3.771								0	level5						other
3.833_88.0524	1.005	0.906	Ethyl acetate	C4 H8 O2	88.0524	3.833	C00849	S8525	HMDB0031217	0	-0.3813			1	level4	Organic acids and derivatives	Carboxylic acids and derivatives	Carboxylic acid derivatives	Organic acids	NULL	other
3.834_132.0788	1.0444	0.5216	Paraldehyde	C6 H12 O3	132.0788	3.834	C07834	S21106173	HMDB0032456	0.0002	1.1682			0	level4	Organoheterocyclic compounds	Trioxanes	null	Trioxanes	NULL	other
3.93_250.0412	1.4158	0.6146		C11 H10 N2 O3 S	250.0412	3.93								3	level5						other
3.943_179.0582	0.897	0.8739	3-succinoylpyridine	C9 H9 N O3	179.0582	3.943	C19569	MReference-220	HMDB0000992	0	-0.0771	14.3	38.9	11	level3	Organic acids and derivatives	Keto acids and derivatives	Gamma-keto acids and derivatives	Organic acids	map00760 Nicotinate and nicotinamide metabolism; map00980 Metabolism of xenobiotics by cytochrome P450; map01100 Metabolic pathways; map05204 Chemical carcinogenesis;	other
3.99_415.278	0.9844	0.9345		C19 H37 N5 O5	415.278	3.99								6	level5						other
4.108_124.0526	1.083	0.6925	Guaiacol	C7 H8 O2	124.0526	4.108	C01502	S447	HMDB0001398	0.0001	1.0572			16	level4	Benzenoids	Phenols	Methoxyphenols	Phenols and derivatives		other
4.117_357.2355	1.0404	0.7456		C19 H37 N O P2	357.2355	4.117								0	level5						other
4.16_467.204	0.7677	0.2012		C19 H40 N3 O2 P3 S	467.204	4.16								17	level5						other

4.161_472.1596	0.7906	0.2779	Beta-l-fructofuranosyl 6-o-[(2e)-3-phenyl-2-propenoyl]-alpha-d-glucopyranoside	C21 H28 O12	472.1596	4.161		S30776758	HMDB0029281	0.0015	3.1253			44	level4	Phenylpropanoids and polyketides	Cinnamic acids and derivatives	Cinnamic acid esters	Polyketides[PK]		other
4.225_137.0478	1.1496	0.6524	Anthranilic acid	C7 H7 N O2	137.0478	4.225	C00108	MReference-113	HMDB0001123	0.0001	0.7901	93.9	94.1	38	level2	Benzenoids	Benzene and substituted derivatives	Benzoic acids and derivatives	Benzene and derivatives	map00380 Tryptophan metabolism; map00400 Phenylalanine, tyrosine and tryptophan biosynthesis; map01100 Metabolic pathways; map01230 Biosynthesis of amino acids;	other
4.306_332.126	0.4504	0.7955	10-ethyl-4-propyl-5,9,10,11-tetrahydro-1h-furo [3',4':5,6]cyclonona[1,2-c]furan-1,3,6,8(4h)-tetrone	C18 H20 O6	332.126	4.306		S3739279	HMDB0034262	0	0.0164			2	level4	Phenylpropanoids and polyketides	Coumarins and derivatives	Pyranocoumarins	Coumarins and derivatives		other
4.36_310.1628	1.0869	0.8591		C14 H22 N4 O4	310.1628	4.36								0	level5						other
4.363_415.278	1.0359	0.6799		C19 H37 N5 O5	415.278	4.363								24	level5						other
4.502_233.1165	0.8966	0.6202	Pardoprunox	C12 H15 N3 O2	233.1165	4.502		S5293722		0.0001	0.3247			0	level4						other
4.608_354.1891	1.0736	0.8402		C16 H26 N4 O5	354.1891	4.608								0	level5						other
4.727_604.2384	0.8178	0.4713		C20 H40 N6 O13 S	604.2384	4.727								1	level5						other
4.736_215.0947	0.7795	0.6438	Atrazine	C8 H14 Cl N5	215.0947	4.736	C06551	S2169	HMDB0041830	0.001	4.4411			3	level4	Organoheterocyclic compounds	Triazines	1,3,5-triazines	1,3,5-triazines	map01100 Metabolic pathways;	other
4.763_234.1619	0.595	0.8779	3138020	C15 H22 O2	234.1619	4.763	C09743	S4945176	HMDB0030016	-0.0001	-0.2211			0	level4	Terpenoids	Sesquiterpenoids (C15)	Others	Terpenoids	NULL	other
4.766_503.3306	1.0595	0.4961		C23 H45 N5 O7	503.3306	4.766								0	level5						other
4.92_546.3257	0.8275	0.3398		C24 H50 O13	546.3257	4.92								0	level5						other
4.975_226.0478	0.3738	0.3544	Prephenic acid	C10 H10 O6	226.0478	4.975		S1001		0.0001	0.4708			0	level4						other
5.046_687.3352	0.9119	0.5522		C29 H60 N3 O7 P3 S	687.3352	5.046								0	level5						other
5.047_133.0892	1.3928	0.4996	Nx4900000	C9 H11 N	133.0892	5.047		S6779	HMDB0012489	0	0.1886			0	level4	Organoheterocyclic compounds	Tetrahydroisoquinolines	null	Tetrahydroisoquinolines		other
5.082_176.1048	1.0333	0.8005	Ethanal tetramer	C8 H16 O4	176.1048	5.082	C18744	S54981		-0.0001	-0.4257			0	level4					NULL	other
5.167_503.2944	1.0254	0.9806		C21 H45 N O12	503.2944	5.167								0	level5						other
5.17_486.2679	1.0224	0.964		C21 H42 O12	486.2679	5.17								0	level5						other
5.183_392.1358	0.8605	0.376	Sn-38	C22 H20 N2 O5	392.1358	5.183	C11173	S94634	HMDB0060510	-0.0014	-3.5151			1	level4	Alkaloids and derivatives	Camptothecins	null	Alkaloids and derivatives	map00983 Drug metabolism - other enzymes;	other
5.213_302.1654	0.9246	0.4326	(2e,6e)-farnesyl monophosphate	C15 H27 O4 P	302.1654	5.213	C20121	S8533001		0.0006	2.1508			0	level4					map00900 Terpenoid backbone biosynthesis;	other
5.22_317.1575	0.9746	0.792		C13 H23 N3 O6	317.1575	5.22								0	level5						other
5.238_298.1205	0.8876	0.9381	Benzyl succinate	C18 H18 O4	298.1205	5.238	C07332	S7370		0	0.0408			0	level4					NULL	other
5.239_316.1309	0.8721	0.8694	Combretastatin a-4	C18 H20 O5	316.1309	5.239	C20268	S4508364		-0.0001	-0.4425			0	level4					NULL	other
5.24_333.1575	0.8461	0.9088	Seneciphylline	C18 H23 N O5	333.1575	5.24	C10391	S4445064		-0.0002	-0.4565			5	level4	Alkaloids	Alkaloids derived from ornithine	Pyrrolizidine alkaloids	Alkaloids	NULL	other
5.339_339.7177	1.0626	0.5915	[similar to: (+/-)5(6)-dihet; δmass: 1.4720 da]		339.7177	5.339								0	level5						other
5.364_140.0475	0.814	0.96	Lv1850000	C7 H8 O3	140.0475	5.364		S11485	HMDB0059862	0.0001	0.8041			0	level4	Organoheterocyclic compounds	Furans	Furoic acid and derivatives	Furoic acid and derivatives		other
5.431_549.3187	1.1747	0.491		C27 H53 N O4 P2 S	549.3187	5.431								0	level5						other
5.453_370.2437	0.9624	0.8187		C14 H34 N4 O7	370.2437	5.453								0	level5						other
5.55_753.4367	0.8993	0.402		C43 H64 N O8 P	753.4367	5.55								0	level5						other
5.649_405.7568	1.1143	0.4333	[similar to: (+/-)5(6)-dihet; δmass: 67.5111 da]		405.7568	5.649								0	level5						other
5.65_406.2585	1.088	0.5705		C21 H34 N4 O4	406.2585	5.65								0	level5						other
5.669_679.3993	0.9366	0.6001		C29 H61 N O16	679.3993	5.669								0	level5						other
5.735_412.2313	1.0501	0.6477	[similar to: (+/-)5(6)-dihet; δmass: 73.9855 da]		412.2313	5.735								0	level5						other
5.773_171.1261	0.9337	0.9047	Gabapentin	C9 H17 N O2	171.1261	5.773	C07018	S3328	HMDB0005015	0.0001	0.8035			0	level4	Organic acids and derivatives	Carboxylic acids and derivatives	Amino acids, peptides, and analogues	Amino acids, peptides, and analogues	NULL	other
5.786_266.1131	0.3955	0.8983		C10 H14 N6 O3	266.1131	5.786								1	level5						other
6.071_210.0894	1.0834	0.8425	3-(3,5-dimethoxyphenyl)propanoic acid	C11 H14 O4	210.0894	6.071		S3095008	HMDB0127493	0.0002	0.8876			17	level4	Phenylpropanoids and polyketides	Phenylpropanoic acids	null	Phenylpropanoic acids		other
6.209_219.0321	0.9848	0.8093		C6 H11 N3 O2 P2	219.0321	6.209								2	level5						other
6.32_148.0891	1.1899	0.5451	Trans-anethole	C10 H12 O	148.0891	6.32	C10428	S553166		0.0003	2.0135			0	level4	Phenylpropanoids	Monolignols	Others	Phenylpropanoids		other
6.483_632.3988	1.1184	0.477		C28 H61 N2 O11 P	632.3988	6.483								33	level5						other
6.702_137.0478	0.8523	0.3276	Trigonelline	C7 H7 N O2	137.0478	6.702	C01004	MReference-806	HMDB0000875	0.0001	1.0078	23.8	61	13	level2	Alkaloids and derivatives	null	null	Alkaloids and derivatives	map00760 Nicotinate and nicotinamide metabolism;	other
7.259_375.2523	0.8562	0.3772		C21 H33 N3 O3	375.2523	7.259								40	level5						other
7.483_236.1414	0.7735	0.479	4-(heptyloxy)benzoic acid	C14 H20 O3	236.1414	7.483	C14274	S76800		0.0002	0.7525			0	level4					NULL	other

7.621_316.1649	0.8809	0.6355		C15 H20 N6 O2	316.1649	7.621								7	level5						other
7.689_243.2569	0.9029	0.8165		C15 H33 N O	243.2569	7.689								0	level5						other
7.7_474.3352	0.6864	0.4976	18-acetoxy-1alpha,25-dihydroxyvitamin d3	C29 H46 O5	474.3352	7.7		S7826460		0.0007	1.471			4	level4						other
7.795_220.1481	1.1433	0.4983	2,6-di-tert-butyl-1,4-benzoquinone	C14 H20 O2	220.1481	7.795							54.5	1	level5						other
7.815_126.0691	1.1824	0.4493			126.0691	7.815								9	level5						other
7.951_250.157	0.7612	0.9748	Gemfibrozil	C15 H22 O3	250.157	7.951	C07020	S3345	HMDB0015371	0.0001	0.3424			3	level4	Benzenoids	Phenol ethers	null	Benzene and derivatives	NULL	other
8.073_435.1699	1.016	0.9776			435.1699	8.073								7	level5						other
8.087_317.0783	0.7774	0.7459		C14 H15 N5 S2	317.0783	8.087								4	level5						other
8.243_342.2883	0.8627	0.6605		C19 H38 N2 O3	342.2883	8.243								10	level5						other
8.336_446.3033	1.0788	0.6146	Carpipramine	C28 H38 N4 O	446.3033	8.336		S2482		-0.0012	-2.7614			12	level4						other
8.758_508.7608	1.1124	0.5451			508.7608	8.758								6	level5						other
8.847_430.3084	1.1255	0.5294	Hecogenin	C27 H42 O4	430.3084	8.847	C08902	S82581		0.0001	0.1502			4	level4	Terpenoids	Steroids	Spirostan	Terpenoids		other
8.888_473.2476	1.0647	0.6717		C21 H39 N5 O3 S2	473.2476	8.888								32	level5						other
8.889_188.087	1.1773	0.4645		C9 H16 O2 S	188.087	8.889								3	level5						other
9.171_522.2608	0.9165	0.6808	2-(2,4-dihydroxy-5-methoxyphenyl)-3-[(2z)-3,7-dimethyl-2,6-octadien-1-yl]-5,7-dihydroxy-6-(3-methyl-2-buten-1-yl)-2,3-dihydro-4h-chromen-4-one	C31 H38 O7	522.2608	9.171		S74852676	HMDB0129835	-0.0009	-1.8076			1	level4	Phenylpropanoids and polyketides	Flavonoids	Flavans	Flavonoids		other
9.172_500.2477	0.8746	0.5281		C22 H36 N4 O9	500.2477	9.172								14	level5						other
9.172_517.7752	0.8754	0.5622			517.7752	9.172								34	level5						other
9.175_634.3771	1.0248	0.8957		C24 H54 N6 O13	634.3771	9.175								1	level5						other
9.176_929.4701	0.9214	0.5826		C49 H75 N3 O8 S3	929.4701	9.176								0	level5						other
9.177_475.7256	0.9072	0.6161			475.7256	9.177								0	level5						other
9.178_478.2344	1.0051	0.8689	Ethyl 3,5,6-tri-o-benzyl-d-glucofuranoside	C29 H34 O6	478.2344	9.178		S23489		-0.0012	-2.476			0	level4						other
9.181_841.4168	0.8784	0.5687		C45 H67 N3 O6 S3	841.4168	9.181								1	level5						other
9.182_780.3638	0.9139	0.7047	Bis(glutathionyl)spermine	C30 H56 N10 O10 S2	780.3638	9.182	C16563	S30791789		0.0015	1.9629			0	level4					map00480 Glutathione metabolism;	other
9.185_577.2591	1.0204	0.8709		C28 H43 N5 O2 S3	577.2591	9.185								0	level5						other
9.234_484.2828	1.0405	0.8378	Stigmatellin y	C29 H40 O6	484.2828	9.234	C12150	S4445302		0.0003	0.5468			1	level4					NULL	other
9.485_567.7832	0.8642	0.591	[similar to: cytidine; δ mass: 324.6977 da]		567.7832	9.485								0	level5						other
9.49_354.0626	0.8351	0.6076		C6 H21 N4 O7 P3	354.0626	9.49								0	level5						other
9.51_827.3833	0.8763	0.6842		C44 H65 N3 O4 S4	827.3833	9.51								0	level5						other
9.671_330.2766	1.041	0.8647	1728235	C19 H38 O4	330.2766	9.671		S14201		-0.0004	-1.2211			0	level4						other
0.564_83.0735	0.856	0.2382	1-piperideine	C5 H9 N	83.0735	0.564	C06181	S61469		0	0.4242			0	level4	Alkaloids	Alkaloids derived from lysine	Piperidine alkaloids	Alkaloids	map01100 Metabolic pathways;	other
0.603_168.0875	0.9766	0.7153	[similar to: mjn110; δ mass: -293.0034 da]		168.0875	0.603								0	level5						other
0.604_435.8624	0.9839	0.7442		C3 H7 N2 O13 P3 S2	435.8624	0.604								0	level5						other
0.637_265.8421	1.0571	0.7217		C2 H Cl O5 P2 S2	265.8421	0.637								0	level5						other
0.64_111.0296	0.8875	0.6451			111.0296	0.64								0	level5						other
0.648_197.0775	0.9206	0.4349			197.0775	0.648								0	level5						other
0.649_157.0851	1.0602	0.8205		C6 H11 N3 O2	157.0851	0.649								1	level5						other
0.649_174.1117	1.0601	0.8216	L-arginine	C6 H14 N4 O2	174.1117	0.649	C00062	BGI343	HMDB0000517	0	0.0963	95.7	94.3	1	level1	Peptides	Amino acids	Common amino acids [Fig]	Amino acids	map00220 Arginine biosynthesis; map00330 Arginine and proline metabolism; map00472 D-Arginine and D-ornithine metabolism; map00970 Aminoacyl-tRNA biosynthesis; map01100 Metabolic pathways; map01230 Biosynthesis of amino acids; map02010 ABC transporters; map04150 mTOR signaling pathway; map04974 Protein digestion and absorption; map05014	other
0.649_365.905	0.9885	0.9659		C5 H5 Cl N2 O11 P2	365.905	0.649								0	level5						other
0.652_363.9087	0.9604	0.7401		C2 H6 O17 P2	363.9087	0.652								0	level5						other
0.653_305.95	0.8658	0.5377		C4 H7 Cl N4 O6 S2	305.95	0.653								0	level5						other
0.658_161.1052	1.0139	0.7644	L(-)-carnitine	C7 H15 N O3	161.1052	0.658	C00318	MReference-1837	HMDB0000062	0	-0.0096		97.5	14	level2	Organic nitrogen compounds	Organonitrogen compounds	Quaternary ammonium salts	Quaternary ammonium salts	map04714 Thermogenesis; map04976 Bile secretion;	other

0.66_117.0789	1.1782	0.7903	L-(+)-valine	C5 H11 N O2	117.0789	0.66	C00183	S6050	HMDB0000883	0	-0.3193			23	level4	Organic acids and derivatives	Carboxylic acids and derivatives	Amino acids, peptides, and analogues	Amino acids	map00280 Valine, leucine and isoleucine degradation; map00290 Valine, leucine and isoleucine biosynthesis; map00770 Pantothenate and CoA biosynthesis; map00970 Aminoacyl-tRNA biosynthesis; map01100 Metabolic pathways; map01210 2-Oxocarboxylic acid metabolism; map01230 Biosynthesis of amino acids; map02010 ABC transporters; map04974 Protein digestion [illegible]	other
0.682_172.0349	1.1093	0.5708		C3 H4 N6 O3	172.0349	0.682						21.9		2	level5						other
0.684_116.0605	1.0185	0.7625			116.0605	0.684								1	level5						other
0.686_114.0295	1.2964	0.6077		C H2 N6 O	114.0295	0.686								0	level5						other
0.722_139.8963	0.9892	0.8514			139.8963	0.722								0	level5						other
0.724_115.111	0.9155	0.526		C5 H13 N3	115.111	0.724								0	level5						other
0.752_219.0015	1.0249	0.8182		C11 H7 Cl N P	219.0015	0.752								6	level5						other
0.752_276.96	0.9976	0.9828		C5 H12 Cl N3 S4	276.96	0.752								0	level5						other
0.779_133.9957	1.2299	0.5374			133.9957	0.779								0	level5						other
0.781_84.0211	0.6995	0.7414	Lu3453000	C4 H4 O2	84.0211	0.781	C17601	S9917	HMDB0032330	0	0.1612			3	level4	Organoheterocyclic compounds	Dihydrofurans	Furanones	Furanones	NULL	other
0.79_290.0627	1.3037	0.79		C11 H14 O9	290.0627	0.79								0	level5						other
0.844_165.0461	1.1616	0.5722	L-methionine sulfoxide	C5 H11 N O3 S	165.0461	0.844	C02989	MReference-2246	HMDB0002005	0.0001	0.7636		86.4	1	level2	Organic acids and derivatives	Carboxylic acids and derivatives	Amino acids, peptides, and analogues	Amino acids, peptides, and analogues	map00270 Cysteine and methionine metabolism;	other
0.993_112.0138	0.8842	0.6668	[similar to: uracil; δ mass: -0.0135 da]		112.0138	0.993								26	level5						other
1.026_145.0199	1.2277	0.5407	3,4-dehydrothiomorpholine-3-carboxylic acid	C5 H7 N O2 S	145.0199	1.026	C04445	S163623		0.0002	1.272	48.8		0	level4					NULL	other
1.148_175.1209	1.343	0.9312		C8 H17 N O3	175.1209	1.148								0	level5						other
1.531_204.057	2.0383	0.9295		C7 H12 N2 O3 S	204.057	1.531								21	level5						other
1.589_85.0892	1.0116	0.9843	2908	C5 H11 N	85.0892	1.589	C01746	S7791	HMDB0034301	0.0001	1.0093			0	level4	Organoheterocyclic compounds	Piperidines	null	Piperidines	map04974 Protein digestion and absorption;	other
1.672_134.1134	1.0023	0.8406			134.1134	1.672								0	level5						other
2.285_172.1214	1.1966	0.8964		C8 H16 N2 O2	172.1214	2.285								0	level5						other
2.337_208.0849	0.9408	0.9602	L-kynurenine	C10 H12 N2 O3	208.0849	2.337	C00328		HMDB0000684	0.0001	0.3798	89.9	89	0	level1	Organic oxygen compounds	Organooxygen compounds	Carbonyl compounds	Carbonyl compounds	map00380 Tryptophan metabolism; map01100 Metabolic pathways; map05143 African trypanosomiasis;	other
2.341_164.0687	0.994	0.9011	A-l-fucopyranose	C6 H12 O5	164.0687	2.341	C20835	S388645	HMDB0059624	0.0002	1.2271	23.2		0	level4	Organic oxygen compounds	Organooxygen compounds	Carbohydrates and carbohydrate conjugates	Carbohydrates	NULL	other
2.403_149.0479	0.5844	0.6211	Dihydroxyindole	C8 H7 N O2	149.0479	2.403	C05578	S102690	HMDB0004058	0.0002	1.1893			0	level4	Organoheterocyclic compounds	Indoles and derivatives	Hydroxyindoles	Indole and derivatives	map00350 Tyrosine metabolism; map01100 Metabolic pathways;	other
2.409_119.0375	0.776	0.8336	Phenylisocyanate	C7 H5 N O	119.0375	2.409		S7389	HMDB0062270	0.0004	3.0295	33.7		4	level4	Benzenoids	Benzene and substituted derivatives	null	Benzene and derivatives		other
2.413_150.0893	1.0067	0.967		C6 H14 O4	150.0893	2.413								0	level5						other
2.482_366.1428	1.054	0.8123		C15 H27 O8 P	366.1428	2.482								0	level5						other
2.585_119.039	0.9114	0.3787	[similar to: phenylethanolamine; δ mass: -18.0451 da]		119.039	2.585								1	level5						other
2.603_165.0203	0.9497	0.6083		C5 H4 N5 P	165.0203	2.603								0	level5						other
2.622_165.0216	0.92	0.7447		C11 H3 N O	165.0216	2.622								0	level5						other
2.664_375.1644	1.1362	0.9384		C13 H30 N O9 P	375.1644	2.664								25	level5						other
2.73_118.0567	1.2272	0.6322		C4 H10 N2 S	118.0567	2.73							34	0	level5						other
2.948_334.1165	0.8636	0.8249		C16 H18 N2 O6	334.1165	2.948								39	level5						other
3.04_167.0584	0.8092	0.6227	Pyridoxal	C8 H9 N O3	167.0584	3.04	C00250	BGI486	HMDB0001545	0.0002	1.136	7.9		0	level3	Vitamins and Cofactors	Vitamins	Water-soluble vitamins [Fig]	Vitamins	map00750 Vitamin B6 metabolism; map01100 Metabolic pathways; map04977 Vitamin digestion and absorption;	other
3.083_238.1417	1.0887	0.8815		C10 H22 O6	238.1417	3.083								5	level5						other
3.083_255.1683	1.0566	0.869		C11 H21 N5 O2	255.1683	3.083								0	level5						other
3.086_260.1236	1.1218	0.9422		C8 H16 N6 O4	260.1236	3.086								0	level5						other
3.136_133.0562	1.034	0.7724	(1e)-n-hydroxy-4-(methylsulfanyl)-1-butanimine	C5 H11 N O S	133.0562	3.136	C17241	S24784893		0.0001	0.7134			0	level4					map01210 2-Oxocarboxylic acid metabolism;	other
3.154_290.1268	1.179	0.7004		C16 H14 N6	290.1268	3.154								7	level5						other

3.206_186.1373	1.0088	0.9762	Capuride	C9 H18 N2 O2	186.1373	3.206		S201505		0.0004	2.4071			6	level4						other
3.217_211.1257	0.7249	0.9014			211.1257	3.217								0	level5						other
3.223_413.2116	0.907	0.697	Delanzomib	C21 H28 B N3 O5	413.2116	3.223		S23325665		-0.0006	-1.5396			0	level4						other
3.246_143.0737	1.05	0.782	6-methylquinoline	C10 H9 N	143.0737	3.246		MReference-2933	HMDB0033115	0.0002	1.3683		81.5	2	level2	Organoheterocyclic compounds	Quinolines and derivatives	null	Quinolines and derivatives		other
3.247_117.0579	1.0595	0.7521	Indole	C8 H7 N	117.0579	3.247	C00463	MReference-444	HMDB0000738	0.0001	0.6799		78.3	3	level2	Organoheterocyclic compounds	Indoles and derivatives	Indoles	Indoles	map00380 Tryptophan metabolism; map00400 Phenylalanine, tyrosine and tryptophan biosynthesis; map01100 Metabolic pathways; map04974 Protein digestion and absorption;	other
3.247_169.053	1.0641	0.7427		C11 H7 N O	169.053	3.247								0	level5						other
3.247_204.09	1.0543	0.7713	Dl-tryptophan	C11 H12 N2 O2	204.09	3.247	C00806	MReference-415	HMDB0030396	0.0001	0.6495	87.2	96.2	0	level2	Organoheterocyclic compounds	Indoles and derivatives	Indolyl carboxylic acids and derivatives	Indole and derivatives	NULL	other
3.249_442.2322	0.8364	0.2965		C22 H30 N6 O4	442.2322	3.249								2	level5						other
3.252_173.0841	1.0163	0.8845	Pyroquilon	C11 H11 N O	173.0841	3.252	C18487	S82768	HMDB0037113	0.0001	0.5182			1	level4	Organoheterocyclic compounds	Quinolines and derivatives	Quinolones and derivatives	Quinolones and derivatives	NULL	other
3.253_189.0675	1.1164	0.646		C6 H12 N3 O2 P	189.0675	3.253								26	level5						other
3.291_292.0882	1.2031	0.6388		C14 H16 N2 O3 S	292.0882	3.291								0	level5						other
3.299_150.0318	0.7802	0.7076	Phenylglyoxylic acid	C8 H6 O3	150.0318	3.299	C02137	S11421	HMDB0001587	0.0001	0.8914			0	level4	Benzenoids	Benzene and substituted derivatives	Benzoyl derivatives	Benzene and derivatives	map00360 Phenylalanine metabolism; map01100 Metabolic pathways;	other
3.439_283.1708	0.8179	0.4521	[similar to: (+/-)5(6)-dihet; δmass: -55.0749 da]		283.1708	3.439								6	level5						other
3.458_297.1788	0.7065	0.5346		C12 H27 N O7	297.1788	3.458								62	level5						other
3.488_238.2399	0.9576	0.9166		C15 H30 N2	238.2399	3.488								0	level5						other
3.517_291.053	1.0565	0.7569	1-(6-oxo-6h-benzo[c]chromen-3-yl)-1h-pyrrole-2,5-dione	C17 H9 N O4	291.053	3.517	C15433	S217643		-0.0002	-0.5848			0	level4					NULL	other
3.569_146.0401	1.2007	0.865	Thta	C6 H10 O2 S	146.0401	3.569	C11072	S391364		0	-0.3307			3	level4					NULL	other
3.616_220.1213	1.0687	0.8087	Phenylacetylglycine dimethylamide	C12 H16 N2 O2	220.1213	3.616	C12958	S18406		0.0001	0.4688	8.1		1	level4					NULL	other
3.687_250.0954	0.8264	0.607		C12 H14 N2 O4	250.0954	3.687								4	level5						other
3.729_423.1776	1.0789	0.8228		C18 H35 N O4 P2 S	423.1776	3.729								0	level5						other
3.743_348.1757	0.9926	0.7568	Efinaconazole	C18 H22 F2 N4 O	348.1757	3.743		S428538		-0.0004	-1.2708			62	level4						other
3.769_259.1321	1.1748	0.9157	(4r)-4-{[3-(2-aminoethyl)-1h-indol-5-yl]methyl}-1,3-oxazolidin-2-one	C14 H17 N3 O2	259.1321	3.769		S30778612	HMDB0060966	0	-0.0653			0	level4	Organoheterocyclic compounds	Indoles and derivatives	Tryptamines and derivatives	Amines and derivatives		other
3.786_260.1371	0.9403	0.9168	L-gamma-glutamyl-l-leucine	C11 H20 N2 O5	260.1371	3.786		S133115	HMDB0011171	-0.0001	-0.4596			10	level4	Organic acids and derivatives	Carboxylic acids and derivatives	Amino acids, peptides, and analogues	Amino acids, peptides, and analogues		other
3.796_133.0528	1.1368	0.6729	5-indolol	C8 H7 N O	133.0528	3.796		S15244	HMDB0059805	0.0001	0.5339			15	level4	Organoheterocyclic compounds	Indoles and derivatives	Hydroxyindoles	Indole and derivatives		other
3.797_104.0263	1.0209	0.9798		C7 H4 O	104.0263	3.797						97.5		1	level5						other
3.797_179.0583	1.1197	0.7493	Hippurate	C9 H9 N O3	179.0583	3.797	C01586	BGI309	HMDB0000714	0.0001	0.4846	95.7	91	0	level1	Benzenoids	Benzene and substituted derivatives	Benzoic acids and derivatives	Benzene and derivatives	map00360 Phenylalanine metabolism; map01100 Metabolic pathways;	other
3.797_76.0314	1.0275	0.9333		C6 H4	76.0314	3.797								0	level5						other
3.798_185.0664	1.0361	0.9567			185.0664	3.798								1	level5						other
3.8_278.983	1.0639	0.9639		C11 H5 N O6 S	278.983	3.8								0	level5						other
3.8_282.0065	1.041	0.9293		C8 H12 O7 P2	282.0065	3.8								0	level5						other
3.803_412.0358	0.9996	0.7366	Imazosulfuron	C14 H13 Cl N6 O5 S	412.0358	3.803	C18443	S83451		0.0001	0.3515			0	level4					NULL	other
3.835_327.2252	0.983	0.8504		C18 H35 N P2	327.2252	3.835								45	level5						other
4.01_303.1583	0.8484	0.8323	N(6)-[(indol-3-yl)acetyl]-l-lysine	C16 H21 N3 O3	303.1583	4.01	C04211	S141640		0	0.0383			1	level4					NULL	other
4.024_189.0427	1.1014	0.6052	4-hydroxy-2-quinolinecarboxylic acid	C10 H7 N O3	189.0427	4.024	C01717	BGI117	HMDB0000715	0.0001	0.7253	98.1	89.7	24	level1	Organoheterocyclic compounds	Quinolines and derivatives	Quinoline carboxylic acids	Quinoline carboxylic acids	map00380 Tryptophan metabolism; map01100 Metabolic pathways;	other
4.117_142.0631	1.0853	0.7105	4-oxocyclohexanecarboxylic acid	C7 H10 O3	142.0631	4.117	C03767	S167248		0.0001	0.7557	54.6		0	level4					NULL	other
4.118_318.1584	1.3038	0.6042	Imiprothrin	C17 H22 N2 O4	318.1584	4.118		S110211		0.0004	1.3861			16	level4						other
4.119_371.2518	1.0042	0.9394		C17 H33 N5 O4	371.2518	4.119								11	level5						other
4.121_182.0557	1.0465	0.8429		C5 H6 N6 O2	182.0557	4.121								28	level5						other
4.126_343.0488	0.6936	0.1398	[similar to: (+/-)5(6)-dihet; δmass: 4.8031 da]		343.0488	4.126								0	level5						other
4.218_256.1722	0.6611	0.8938		C12 H24 N4 S	256.1722	4.218								0	level5						other
4.305_431.2731	0.9058	0.4159	Istamycin c1	C19 H37 N5 O6	431.2731	4.305	C17996	S30791521		-0.0013	-3.0035			0	level4					NULL	other
4.318_511.2303	0.9149	0.7251	Verruculogen	C27 H33 N3 O7	511.2303	4.318	C20045	S10405461		-0.0016	-3.0472			0	level4	Fungal toxins	Mycotoxins	Others	Fungal toxins	map01100 Metabolic pathways;	other

4.47_146.037	1.035	0.7378	Coumarin	C9 H6 O2	146.037	4.47	C05851	MReference-356	HMDB0001218	0.0002	1.2965		77.6	0	level2	Phenylpropanoids and polyketides	Coumarins and derivatives	null	Coumarins and derivatives		other
4.471_164.0475	1.0506	0.7047	4-coumarate	C9 H8 O3	164.0475	4.471	C00811	BGI115	HMDB0002035	0.0002	1.1341	91.4	87	0	level2	Phenylpropanoids	Monolignols	Paracoumaryl alcohol derivatives	Phenylpropanoids	map00130 Ubiquinone and other terpenoid-quinone biosynthesis; map00350 Tyrosine metabolism; map01100 Metabolic pathways;	other
4.535_130.042	1.0207	0.6943		C9 H6 O	130.042	4.535						62.2		0	level5						other
4.575_459.3043	1.0363	0.6735		C21 H41 N5 O6	459.3043	4.575								0	level5						other
4.584_127.1001	1.043	0.8669	Lq1825000	C7 H13 N O	127.1001	4.584	C11519	S12476		0.0004	3.0958			1	level4						other
4.7_461.2838	1.0064	0.9431		C20 H39 N5 O7	461.2838	4.7								1	level5						other
4.753_502.2994	0.963	0.5302		C21 H47 N2 O9 P	502.2994	4.753								6	level5						other
4.757_582.2562	0.8539	0.3423		C25 H49 N2 O5 P3 S	582.2562	4.757								5	level5						other
4.819_398.2153	0.9995	0.8596		C21 H36 O3 P2	398.2153	4.819								2	level5						other
4.861_166.0995	0.9947	0.8523	Tbhq	C10 H14 O2	166.0995	4.861		S15235	HMDB0032062	0.0001	0.8074			0	level4	Benzenoids	Benzene and substituted derivatives	Phenylpropanes	Benzene and derivatives		other
4.895_563.3518	0.8791	0.4613		C25 H49 N5 O9	563.3518	4.895								7	level5						other
4.932_547.3569	1.0609	0.5081		C25 H49 N5 O8	547.3569	4.932								8	level5						other
4.973_180.0424	0.3683	0.415	Aspirin	C9 H8 O4	180.0424	4.973	C01405	S2157	HMDB0001879	0.0001	0.8184			0	level4	Benzenoids	Benzene and substituted derivatives	Benzoic acids and derivatives	Benzene and derivatives	map04976 Bile secretion;	other
5.006_442.2415	1.0175	0.9479		C17 H41 N4 O3 P3	442.2415	5.006								0	level5						other
5.044_607.3782	0.9381	0.5584		C27 H53 N5 O10	607.3782	5.044								0	level5						other
5.052_590.3517	0.8982	0.4421		C26 H54 O14	590.3517	5.052								0	level5						other
5.08_458.2731	1.006	0.9261		C24 H44 O4 P2	458.2731	5.08								0	level5						other
5.137_130.042	0.7689	0.3377		C9 H6 O	130.042	5.137						69.7		1	level5						other
5.174_275.1192	1.0662	0.7103		C12 H21 N O4 S	275.1192	5.174								0	level5						other
5.215_590.3516	1.0074	0.949		C27 H50 N4 O10	590.3516	5.215								0	level5						other
5.216_635.4091	1.0037	0.9514		C34 H58 N3 O6 P	635.4091	5.216								0	level5						other
5.303_695.4306	0.8556	0.3871		C33 H68 N3 O6 P3	695.4306	5.303								0	level5						other
5.312_530.2944	0.9341	0.5144		C22 H47 N2 O10 P	530.2944	5.312								0	level5						other
5.314_547.321	0.9283	0.4928		C22 H50 N3 O10 P	547.321	5.314								54	level5						other
5.339_679.4355	1.0479	0.6742		C34 H67 N O8 P2	679.4355	5.339								24	level5						other
5.34_339.1706	0.963	0.7789		C14 H29 N O6 S	339.1706	5.34								0	level5						other
5.416_436.1623	0.8653	0.4734		C24 H24 N2 O6	436.1623	5.416								40	level5						other
5.45_696.434	1.0611	0.3797		C32 H66 N4 O8 P2	696.434	5.45								0	level5						other
5.451_678.4041	1.0566	0.5526		C30 H62 O16	678.4041	5.451								1	level5						other
5.452_358.1749	1.0101	0.8771		C17 H22 N6 O3	358.1749	5.452								0	level5						other
5.452_361.1837	1.0107	0.8881		C15 H27 N3 O7	361.1837	5.452								0	level5						other
5.452_361.7307	1.0436	0.6528			361.7307	5.452								0	level5						other
5.453_546.3258	0.9464	0.5754		C27 H53 N2 O3 P3	546.3258	5.453								0	level5						other
5.454_362.2324	1.0465	0.6981		C17 H35 N2 O4 P	362.2324	5.454								0	level5						other
5.532_383.2278	1.0432	0.741		C15 H33 N3 O8	383.2278	5.532								1	level5						other
5.555_388.2101	1.0358	0.8856	Ilomastat	C20 H28 N4 O4	388.2101	5.555		S117009		-0.001	-2.539			61	level4						other
5.559_635.3734	0.994	0.8482		C25 H60 N5 O7 P3	635.3734	5.559								0	level5						other
5.62_115.0457	0.9529	0.8941	Butyl isothiocyanate	C5 H9 N S	115.0457	5.62		S11124	HMDB0031328	0.0001	0.9961			1	level4	Organosulfur compounds	Isothiocyanates	null	Isothiocyanates		other
5.622_165.0283	0.9806	0.9612	8-oxoguanine	C5 H3 N5 O2	165.0283	5.622		S106574	HMDB0041820	-0.0003	-1.9724			0	level4	Organoheterocyclic compounds	Imidazopyrimidines	Purines and purine derivatives	Purines and derivatives		other
5.678_162.0534	1.092	0.6494	Diethylpyrocarbonate	C6 H10 O5	162.0534	5.678	C11592	S2943	HMDB0032873	0.0005	3.3934	82		0	level4	Organic acids and derivatives	Organic carbonic acids and derivatives	null	Organic acids	NULL	other
5.707_410.2228	0.8169	0.9283	[similar to: (+/-)5(6)-dihet; δmass: 71.9771 da]		410.2228	5.707								6	level5						other
5.787_240.1363	0.6094	0.5349		C13 H20 O4	240.1363	5.787								0	level5						other
6.016_206.0945	0.3709	0.679	2210033	C12 H14 O3	206.0945	6.016	C10476	S4445096	HMDB0030762	0.0002	0.8479			10	level4	Phenylpropanoids and polyketides	Cinnamic acids and derivatives	Cinnamic acid esters	Polyketides[PK]	NULL	other
6.32_138.1046	1.094	0.7658	Isophorone	C9 H14 O	138.1046	6.32	C14743	MReference-774	HMDB0031195	0.0001	0.8947		84.1	0	level2	Organic oxygen compounds	Organooxygen compounds	Carbonyl compounds	Carbonyl compounds	NULL	other
6.321_202.1207	1.1572	0.6593	Sebacic acid	C10 H18 O4	202.1207	6.321	C08277	MReference-916	HMDB0000792	0.0002	0.7923		69.1	15	level2	Lipids and lipid-like molecules	Fatty Acyls	Fatty acids and conjugates	Fatty acyls[FA]	NULL	other
6.323_224.1025	1.0958	0.7365		C8 H12 N6 O2	224.1025	6.323								5	level5						other
6.375_217.1138	1.0035	0.984		C10 H19 N O2 S	217.1138	6.375								0	level5						other
6.632_140.1203	1.0361	0.9931	(e)-non-2-enal	C9 H16 O	140.1203	6.632		S4446456		0.0001	1.0647			0	level4						other
6.793_256.2151	1.1618	0.7091		C14 H28 N2 O2	256.2151	6.793								0	level5						other
6.859_152.1202	1.1692	0.6284	D-(+)-camphor	C10 H16 O	152.1202	6.859	C00808	MReference-2727	HMDB0059838	0.0001	0.812		90.3	0	level2	Lipids and lipid-like molecules	Prenol lipids	Monoterpenoids	Terpenoids	map04750 Inflammatory mediator regulation of TRP channels;	other
6.87_308.126	1.0348	0.9986	Mono(2-ethyl-5-carboxypentyl) phthalate	C16 H20 O6	308.126	6.87		S130805	HMDB0094647	0	-0.0814			2	level4	Benzenoids	Benzene and substituted derivatives	Benzoic acids and derivatives	Benzene and derivatives		other
7.02_181.1105	0.7924	0.9917	3,4-dimethoxyphenethylamine	C10 H15 N O2	181.1105	7.02		S8114	HMDB0041806	0.0002	1.2551			1	level4	Benzenoids	Benzene and substituted derivatives	Methoxybenzenes	Benzene and derivatives		other
7.062_182.1309	0.4069	0.8139	Yq3683000	C11 H18 O2	182.1309	7.062		S28797		0.0002	1.1638			0	level4						other
7.321_166.136	1.0865	0.7647	Dihydrojasmone	C11 H18 O	166.136	7.321		S56166	HMDB0031565	0.0002	1.0866			0	level4	Organic oxygen compounds	Organooxygen compounds	Carbonyl compounds	Carbonyl compounds		other
7.673_230.1315	0.8771	0.757	Dehydrocostus lactone	C15 H18 O2	230.1315	7.673	C09387	S65938		0.0008	3.576			0	level4	Terpenoids	Sesquiterpenoids (C15)	Guaianolide	Terpenoids	NULL	other

7.675_221.1507	0.8908	0.5555			221.1507	7.675								0	level5						other
7.79_301.2095	1.6336	0.7868		C16 H33 N P2	301.2095	7.79								0	level5						other
7.813_148.0173	1.1224	0.5774	[similar to: silibinin; δ mass: -334.1040 da]		148.0173	7.813								0	level5						other
7.83_303.2792	1.1183	0.6148		C16 H38 N3 P	303.2792	7.83								0	level5						other
7.961_354.2559	0.926	0.885	Bufa-20,22-dienolide	C24 H34 O2	354.2559	7.961	C16921	S30791731		0	0.0154			0	level4					NULL	other
8.074_451.1861	0.8899	0.6496	Doxazosin	C23 H25 N5 O5	451.1861	8.074	C06970	S3045	HMDB0014728	0.0006	1.2281			0	level4	Organoheterocyclic compounds	Diazinanes	Piperazines	Piperazines	NULL	other
8.078_656.4516	0.8959	0.6308		C33 H70 O8 P2	656.4516	8.078								0	level5						other
8.098_273.0873	0.928	0.6323		C11 H16 Cl N3 O3	273.0873	8.098								0	level5						other
8.205_188.1566	1.1036	0.6981		C14 H20	188.1566	8.205								0	level5						other
8.663_298.2508	0.8401	0.704	Ricinelaidic acid	C18 H34 O3	298.2508	8.663		S393218		0	-0.1198			1	level4						other
8.735_292.2038	1.0925	0.6494	12-oxo phytodienoic acid	C18 H28 O3	292.2038	8.735	C01226	MReference-9879		0	-0.1071		33.9	0	level3	FA Fatty acyls	FA02 Octadecanoids	FA0201 12-oxophytodienoic acid metabolites	Fatty acyls[FA]	map00592 alpha-Linolenic acid metabolism; map01100 Metabolic pathways;	other
8.855_334.2119	1.0581	0.6932		C14 H31 N4 O3 P	334.2119	8.855							57.1	3	level5						other
9.095_308.2328	0.9984	0.8976		C15 H28 N6 O	308.2328	9.095								0	level5						other
9.166_222.0565	0.9889	0.8503	3-(3-methylthio)propylmalic acid	C8 H14 O5 S	222.0565	9.166	C17215	S24808248		0.0003	1.5274			0	level4					map01210 2-Oxocarboxylic acid metabolism;	other
9.171_539.7883	0.9494	0.8565			539.7883	9.171								0	level5						other
9.172_541.765	0.88	0.7321			541.765	9.172								0	level5						other
9.177_544.2735	0.9134	0.7217		C27 H41 N6 O2 P S	544.2735	9.177								0	level5						other
9.179_298.0544	0.9758	0.9653	Quinalphos	C12 H15 N2 O3 P S	298.0544	9.179	C11030	S24335		0.0003	0.9569			1	level4					NULL	other
9.18_476.2265	0.9447	0.7581		C15 H36 N6 O9 S	476.2265	9.18								0	level5						other
9.181_516.2065	0.9654	0.8841		C34 H24 N6	516.2065	9.181								0	level5						other
9.183_753.3639	0.9228	0.7304		C41 H59 N3 O4 S3	753.3639	9.183								0	level5						other
9.183_797.3904	0.9108	0.6948		C38 H63 N5 O7 S3	797.3904	9.183								0	level5						other
9.185_709.3379	1.002	0.925		C31 H59 N5 O5 S4	709.3379	9.185								0	level5						other
9.185_824.3902	0.8854	0.5932		C45 H64 N2 O6 S3	824.3902	9.185								0	level5						other
9.186_692.3114	0.9921	0.9657		C34 H52 N4 O5 S3	692.3114	9.186								0	level5						other
9.187_626.2403	0.972	0.8774		C32 H42 N4 O3 S3	626.2403	9.187								0	level5						other
9.191_708.8766	0.8248	0.4879		C26 H6 N3 O14 P3 S	708.8766	9.191								0	level5						other
9.2_664.8508	1.0314	0.7568		C19 H2 N5 O15 P3 S	664.8508	9.2								0	level5						other
9.231_529.3407	0.9535	0.878		C25 H48 N5 O5 P	529.3407	9.231								0	level5						other
0.582_128.1314	3.2213	0.6163		C7 H16 N2	128.1314	0.582								0	level5						other
0.605_163.9132	0.9916	0.7847			163.9132	0.605								0	level5						other
0.635_205.8866	1.0032	0.9629			205.8866	0.635								0	level5						other
0.635_331.832	0.974	0.877			331.832	0.635								0	level5						other
0.646_439.7182	1.1766	0.6462			439.7182	0.646								0	level5						other
0.652_247.9915	0.9722	0.7508		C3 H12 Cl N4 O P3	247.9915	0.652								0	level5						other
0.655_202.0453	1.0047	0.9591	D-fructose	C6 H12 O6	202.0453	0.655	C02336		HMDB0000660			59.2		0	level5	Organic oxygen compounds	Organooxygen compounds	Carbohydrates and carbohydrate conjugates	Carbohydrates	map00520 Amino sugar and nucleotide sugar metabolism;	other
0.656_219.0594	0.9586	0.559		C7 H14 N3 O P S	219.0594	0.656								1	level5						other
0.659_192.1475	1.4146	0.6673		C8 H20 N2 O3	192.1475	0.659								0	level5						other
0.66_317.9624	1.0085	0.9463		C5 H11 Cl N6 S4	317.9624	0.66								1	level5						other
0.663_75.0685	1.168	0.5568	Trimethylamine n-oxide	C3 H9 N O	75.0685	0.663	C01104	S1113	HMDB0000925	0.0001	0.6725			0	level4	Organic nitrogen compounds	Organonitrogen compounds	Aminoxides	Aminoxides	map01100 Metabolic pathways;	other
0.668_243.0719	1.128	0.6077		C6 H17 N3 O3 S2	243.0719	0.668								2	level5						other
0.671_128.0586	1.0184	0.6509	Dihydrothymine	C5 H8 N2 O2	128.0586	0.671	C00906	MReference-398	HMDB0000079	0.0001	0.5822		38.9	3	level3	Organoheterocyclic compounds	Diazines	Pyrimidines and pyrimidine derivatives	Pyrimidines and pyrimidine derivatives		other
0.682_216.061	1.0227	0.7635		C5 H8 N6 O4	216.061	0.682								0	level5						other
0.692_100.1001	1.9326	0.7078		C5 H12 N2	100.1001	0.692								0	level5						other
0.706_154.0354	0.9854	0.6242	[similar to: sulfamerazine; δmass: -110.0327 da]		154.0354	0.706								0	level5						other
0.779_139.0609	0.9779	0.9638			139.0609	0.779								0	level5						other
0.788_115.111	0.9111	0.7486		C5 H13 N3	115.111	0.788								0	level5						other
0.854_133.9957	1.0578	0.8159		C4 H7 O P S	133.9957	0.854								0	level5						other
1.347_100.0161	1.2192	0.5167	Succinic anhydride	C4 H4 O3	100.0161	1.347	C19524	S7634	HMDB0032523	0.0001	0.9067			4	level4	Organic acids and derivatives	Carboxylic acids and derivatives	Dicarboxylic acids and derivatives	Organic acids	NULL	other
1.372_127.0206	1.1083	0.6646		C4 H5 N3 S	127.0206	1.372								0	level5						other
2.366_150.0893	0.8193	0.7498		C6 H14 O4	150.0893	2.366								0	level5						other
2.414_93.058	0.8271	0.7361	Aniline	C6 H7 N	93.058	2.414	C00292	S5889	HMDB0003012	0.0001	1.1764			0	level4	Benzenoids	Benzene and substituted derivatives	Aniline and substituted anilines	Benzene and derivatives		other
2.634_106.0419	1.0075	0.9217	Benzaldehyde	C7 H6 O	106.0419	2.634	C00193	S235	HMDB0006115	0.0001	0.6908	87.4		0	level4	Benzenoids	Benzene and substituted derivatives	Benzoyl derivatives	Benzene and derivatives		other

2.639_148.0525	0.9467	0.9184	Cinnamic acid	C9 H8 O2	148.0525	2.639	C00423	S392447	HMDB0000930	0.0001	0.6711	49.2		2	level4	Phenylpropanoids and polyketides	Cinnamic acids and derivatives	Cinnamic acids	Polyketides[PK]	map00130 Ubiquinone and other terpenoid-quinone biosynthesis; map00360 Phenylalanine metabolism; map01100 Metabolic pathways;	other
2.734_134.0515	0.9363	0.738		C4 H10 N2 O S	134.0515	2.734								0	level5						other
3.045_194.1156	0.9432	0.7552	Peg-4	C8 H18 O5	194.1156	3.045		S7908	HMDB0094708	0.0002	0.934			0	level4	Organic oxygen compounds	Organooxygen compounds	Ethers	Ethers		other
3.092_205.1317	6.4358	0.86	Panthenol	C9 H19 N O4	205.1317	3.092	C05944	S4516	HMDB0004231	0.0002	1.2025			0	level4	Organic oxygen compounds	Organooxygen compounds	Alcohols and polyols	Alcohols	map00770 Pantothenate and CoA biosynthesis;	other
3.113_213.1956	1.0813	0.9738		C10 H23 N5	213.1956	3.113								0	level5						other
3.235_322.0623	1.2851	0.5678		C8 H17 N6 O2 P3	322.0623	3.235								2	level5						other
3.243_308.0832	1.3769	0.4969	Alprazolam	C17 H13 Cl N4	308.0832	3.243	C06817	S2034	HMDB0014548	0.0003	0.9558			0	level4	Organoheterocyclic compounds	Benzodiazepines	1,4-benzodiazepines	1,4-benzodiazepines	NULL	other
3.246_114.0465	1.0418	0.7874		C9 H6	114.0465	3.246								0	level5						other
3.247_114.047	1.0614	0.7335		C9 H6	114.047	3.247								0	level5						other
3.247_141.058	1.0467	0.7815		C10 H7 N	141.058	3.247								0	level5						other
3.247_145.0529	1.043	0.7882	8-hydroxyquinoline	C9 H7 N O	145.0529	3.247	C19434	MReference-2633		0.0002	1.1717		90.9	0	level2					NULL	other
3.248_131.0736	1.0634	0.7276	Skatole	C9 H9 N	131.0736	3.248	C08313	MReference-215	HMDB0000466	0.0001	0.8984		64.8	0	level2	Organoheterocyclic compounds	Indoles and derivatives	Indoles	Indoles	map00380 Tryptophan metabolism;	other
3.252_159.0685	0.8354	0.6689	Indole-3-acetaldehyde	C10 H9 N O	159.0685	3.252	C00637	BGI320	HMDB0001190	0.0001	0.8031	81.2		0	level2	Organoheterocyclic compounds	Indoles and derivatives	Indoles	Indoles	map00380 Tryptophan metabolism; map01100 Metabolic pathways;	other
3.302_201.0792	0.9861	0.8591	Carbaryl	C12 H11 N O2	201.0792	3.302	C07491	S5899		0.0002	1.1358			0	level4					NULL	other
3.352_246.1368	1.1478	0.9105	Hypaphorine	C14 H18 N2 O2	246.1368	3.352	C09213	S390633	HMDB0061115	0	0.0484			0	level4	Organic acids and derivatives	Carboxylic acids and derivatives	Amino acids, peptides, and analogues	Amino acids, peptides, and analogues	NULL	other
3.363_114.0682	1.0192	0.8707	Trans-3-hexenoic acid	C6 H10 O2	114.0682	3.363		MReference-2539		0.0002	1.3647		61.1	0	level2						other
3.371_175.0668	1.1574	0.5938		C7 H13 N O2 S	175.0668	3.371							37.3	2	level5						other
3.436_304.1492	1.0534	0.8391		C10 H20 N6 O5	304.1492	3.436								1	level5						other
3.438_299.1939	0.9167	0.4477		C16 H31 N P2	299.1939	3.438								0	level5						other
3.456_206.1157	1.0077	0.8994		C9 H18 O5	206.1157	3.456								0	level5						other
3.693_222.1104	1.1182	0.8543		C9 H18 O6	222.1104	3.693								0	level5						other
3.795_104.0395	1.0897	0.6974		C4 H9 O P	104.0395	3.795								0	level5						other
3.806_116.095	1.0307	0.937	Tetramethylurea	C5 H12 N2 O	116.095	3.806		S11930	HMDB0062789	0	-0.0273			6	level4	Organic acids and derivatives	Organic carbonic acids and derivatives	Ureas	Organic acids		other
3.806_71.0372	1.0426	0.9133	6185892	C3 H5 N O	71.0372	3.806	C01659	S6331	HMDB0004296	0.0001	1.0554			0	level4	Organic acids and derivatives	Carboximidic acids and derivatives	Carboximidic acids	Organic acids		other
3.907_294.1216	1.0162	0.9644	Methyl alpha-aspartylphenylalaninate	C14 H18 N2 O5	294.1216	3.907		S2155		0	-0.0306			17	level4						other
4.063_266.1366	1.1094	0.727		C11 H22 O7	266.1366	4.063								1	level5						other
4.372_494.2036	0.8646	0.3347		C20 H35 N2 O10 P	494.2036	4.372								0	level5						other
4.709_176.0475	1.0765	0.8831	Hymecromone	C10 H8 O3	176.0475	4.709	C03081	S4444190	HMDB0059622	0.0002	0.939	89		15	level4	Phenylpropanoids and polyketides	Coumarins and derivatives	Hydroxycoumarins	Coumarins and derivatives	NULL	other
4.711_194.0581	0.9255	0.5708	Ferulate	C10 H10 O4	194.0581	4.711	C01494	BGI265	HMDB0000954	0.0002	0.9176	92.5		5	level3	Phenylpropanoids	Monolignols	Coniferyl alcohol derivatives	Phenylpropanoids	map01100 Metabolic pathways;	other
4.715_224.105	0.961	0.6342	Ethylvanillin propylene glycol acetal	C12 H16 O4	224.105	4.715		S98408	HMDB0036203	0.0001	0.6265			0	level4	Benzenoids	Phenol ethers	null	Benzene and derivatives		other
4.755_205.0741	1.0013	0.9822	Indole-3-lactic acid	C11 H11 N O3	205.0741	4.755	C02043	MReference-450	HMDB0000671	0.0002	0.8058		90.4	0	level2	Organoheterocyclic compounds	Indoles and derivatives	Indolyl carboxylic acids and derivatives	Indole and derivatives		other
4.766_475.2993	0.9015	0.4314	Netilmicin	C21 H41 N5 O7	475.2993	4.766	C07657	S20152952	HMDB0015090	-0.0013	-2.7898			36	level4	Organic oxygen compounds	Organooxygen compounds	Carbohydrates and carbohydrate conjugates	Carbohydrates	NULL	other
4.892_648.2644	0.9882	0.7728		C34 H40 N4 O7 S	648.2644	4.892								1	level5						other
4.919_626.2824	0.947	0.509		C26 H47 N2 O13 P	626.2824	4.919								0	level5						other
5.041_612.333	0.8397	0.3217		C26 H55 N4 O6 P3	612.333	5.041								0	level5						other
5.066_670.3084	0.958	0.5304		C25 H55 N2 O14 P S	670.3084	5.066								0	level5						other
5.12_175.0635	0.9266	0.4791	Indole-3-acetic acid	C10 H9 N O2	175.0635	5.12	C00954	BGI323	HMDB0000197	0.0001	0.8078	97.1	91.3	0	level1	Alkaloids	Alkaloids derived from tryptophan and anthranilic acid	Indole alkaloids	Alkaloids	map00380 Tryptophan metabolism; map01100 Metabolic pathways;	other
5.215_414.2468	1.0216	0.835		C18 H38 O10	414.2468	5.215								0	level5						other
5.333_546.3258	0.9233	0.4649	[similar to: (+/-)5(6)-dihet; δmass: 208.0801 da]		546.3258	5.333								0	level5						other
5.512_203.0982	1.0764	0.6984		C9 H17 N O2 S	203.0982	5.512								1	level5						other
5.555_380.1882	1.0266	0.694		C16 H33 N2 O4 P S	380.1882	5.555								1	level5						other
5.555_383.1968	1.0253	0.7862		C16 H33 N O7 S	383.1968	5.555								9	level5						other
5.624_179.044	0.9872	0.9275	181594	C6 H5 N5 O2	179.044	5.624	C03975	S10277	HMDB0000704	-0.0003	-1.8858			0	level4	Organoheterocyclic compounds	Pteridines and derivatives	Pterins and derivatives	Pteridines and derivatives	NULL	other
5.649_405.2098	0.9973	0.9352		C17 H31 N3 O8	405.2098	5.649								2	level5						other
6.71_252.1838	0.9899	0.7681		C14 H24 N2 O2	252.1838	6.71								0	level5						other
7.043_192.1153	0.7273	0.8317	Ibufenac	C12 H16 O2	192.1153	7.043		S14516		0.0002	1.2192			0	level4						other

7.323_176.1203	1.1236	0.7397	2740	C12 H16 O	176.1203	7.323		S190439	HMDB0031569	0.0002	0.9863		81	0	level4	Benzenoids	Benzene and substituted derivatives	null	Benzene and derivatives		other
7.819_140.9526	1.0788	0.6752			140.9526	7.819								2	level5						other
7.829_414.2069	0.0968	0.9343		C25 H26 N4 O2	414.2069	7.829								0	level5						other
8.852_294.2195	0.8069	0.9129	9-oxo-10(e),12(e)-octadecadienoic acid	C18 H30 O3	294.2195	8.852	C14766	MReference-5838	HMDB0004669	0	-0.0266		39.7	0	level3	Lipids and lipid-like molecules	Fatty Acyls	Lineolic acids and derivatives	Fatty acyls[FA]	map00591 Linoleic acid metabolism;	other
9.176_520.2527	0.9398	0.8091	[similar to: dl-dipalmitoylphosphatidylcholine; δ mass: -213.3095 da]		520.2527	9.176								0	level5						other
9.183_533.2332	0.959	0.8433		C26 H37 Cl N5 O3 P	533.2332	9.183								5	level5						other
9.184_736.3374	0.9385	0.7868		C30 H65 N4 O4 P S5	736.3374	9.184								0	level5						other

Compound.ID is consisted of Retention time and Molecular Weight. The metabolite ID in the column starting with BGI is the ID in the BGI Library database; the metabolite ID starting with M is the ID in the mzCloud Library database; the metabolite ID starting with S is the ID in the ChemSpider Library database. The mzVault.Best.Match refers to compound's secondary spectrum matching score in the BGI self-built standard library. The mzCloud.Best.Match refers to compound's secondary spectrum matching score in the mzCloud standard library. The meanings of different levels are as follows: Level 1: Substances can be accurately identified from the standard database and laboratory data. Level 2: Structural formula can match the standard database. Level 3: The structural formula partly can match the standard database, but needs further verification. Level 4: The accurate MS1 molecular weight can match the database. Level 5: There are no matches and no identification results in the database. The Super.class, Class and Sub.class refer to the second, third and fourth level of HMDB database classification respectively. The lable means differential metabolite filter labels, up or down are differential metabolites, other is not a differential metabolite.
