# Supplementary material for: Metabolic Profiling of Serum for Osteoarthritis Biomarkers
Source: Dis Markers. 2022 Jul 28;2022:1800812. doi: 10.1155/2022/1800812 (PMC9356247; doi:10.1155/2022/1800812)
Supplement: Supplementary 2 — Table S2. Metabolites detected in negative ion mode. [file 1800812.f2.pdf]

Table S2 Metabolites detected in negative ion mode																					
Compound.ID	Fold-change	t.test_p.value	Name	Formula	Molecular Weight	Retention time	KEGG.ID	mzCloud.ID..ChemSpider.ID	HMDB.ID	DeltaMass..Da.	DeltaMass..ppm.	mzVault.Best.Match	mzCloud.Best.Match	ChemSpider.Results	level	Super.class	Class	Sub.class	Family	Pathway	label
0.564_234.0375	2.3573	0.0749	2-(2-acetoxy-2-oxoethyl)-2-hydroxysuccinic acid	C8 H10 O8	234.0375	0.564		S67159455	HMDB0059808	-0.0001	-0.2491			1	level4	Organic acids and derivatives	Carboxylic acids and derivatives	Tetracarboxylic acids and derivatives	Organic acids		other
0.572_225.9783	0.1397	0.8391		C5 H6 O8 S	225.9783	0.572								0	level5						other
0.578_239.9938	1.3386	0.7814		C6 H8 O8 S	239.9938	0.578								0	level5						other
0.582_176.0321	2.2196	0.0004	Ascorbic acid	C6 H8 O6	176.0321	0.582	C00072	MReference-325	HMDB0000044	0	0.1701		49.3	21	level3	Organoheterocyclic compounds	Dihydrofurans	Furanones	Furanones	map00053 Ascorbate and aldarate metabolism; map00480 Glutathione metabolism; map01100 Metabolic pathways; map04066 HIF-1 signaling pathway; map04977 Vitamin digestion and absorption;	up
0.59_289.9942	1.3857	0.3668		C6 H10 O11 S	289.9942	0.59								0	level5						other
0.591_387.0407	0.9036	0.7247		C10 H19 N3 O7 P2 S	387.0407	0.591								0	level5						other
0.593_146.0216	1.4616	0.3353	2-oxoglutaric acid	C5 H6 O5	146.0216	0.593	C00026	MReference-305	HMDB0000208	0.0001	0.3632	87.3	88.3	7	level2	Organic acids and derivatives	Keto acids and derivatives	Gamma-keto acids and derivatives	Organic acids	map00020 Citrate cycle (TCA cycle); map00040 Pentose and glucuronate interconversions; map00053 Ascorbate and aldarate metabolism; map00220 Arginine biosynthesis; map00250 Alanine, aspartate and glutamate metabolism; map00310 Lysine degradation; map00340 Histidine metabolism; map00430 Taurine and hypotaurine metabolism; map00471 D-Glutamine and D-glutamate metabolism; map00630 Glyoxylate and dicarboxylate [illegible]	other
0.593_97.9673	0.9811	0.7358	Sulfuric acid	H2 O4 S	97.9673	0.593	C00059	S1086	HMDB0001448	0	-0.3802			1	level4	Homogeneous non-metal compounds	Non-metal oxoanionic compounds	Non-metal sulfates	Non-metal sulfates	map00230 Purine metabolism; map00270 Cysteine and methionine metabolism; map00920 Sulfur metabolism; map01100 Metabolic pathways; map02010 ABC transporters;	other
0.596_192.0271	1.4393	0.3459	Citrate	C6 H8 O7	192.0271	0.596	C00158	BGI188	HMDB0000094	0.0001	0.3537	98.2	95.2	13	level1	Organic acids	Carboxylic acids [Fig]	Tricarboxylic acids	Organic acids	map00020 Citrate cycle (TCA cycle); map00250 Alanine, aspartate and glutamate metabolism; map00630 Glyoxylate and dicarboxylate metabolism; map01100 Metabolic pathways; map01200 Carbon metabolism; map01210 2-Oxocarboxylic acid metabolism; map01230 Biosynthesis of amino acids; map04742 Taste transduction; map04922 Glucagon signaling pathway; map05230 Central carbon metabolism in cancer;	other
0.598_112.0161	1.4003	0.3323	3-furoic acid	C5 H4 O3	112.0161	0.598		MReference-1735	HMDB0000444	0	0.3855		73.4	4	level2	Organoheterocyclic compounds	Furans	Furoic acid and derivatives	Furoic acid and derivatives		other
0.601_134.0215	1.3948	0.1395	Dl-malic acid	C4 H6 O5	134.0215	0.601	C03668	MReference-1796	HMDB0000744	0	0.1641	96.2	96.4	5	level2						other
0.601_148.0372	1.1137	0.4691	(+/-)-2-hydroxyglutaric acid	C5 H8 O5	148.0372	0.601	C02630	S42	HMDB0059655	0.0001	0.5236	88.4	95.4	20	level4	Organic acids and derivatives	Hydroxy acids and derivatives	Short-chain hydroxy acids and derivatives	Organic acids	map00650 Butanoate metabolism; map01100 Metabolic pathways;	other
0.601_174.0165	0.9651	0.9871	Trans-aconitic acid	C6 H6 O6	174.0165	0.601	C02341	MReference-350	HMDB0000958	0	0.2074	37.2	84.9	6	level2	Organic acids and derivatives	Carboxylic acids and derivatives	Tricarboxylic acids and derivatives	Organic acids	map01100 Metabolic pathways;	other

0.603_116.011	1.4747	0.0857	Fumaric acid	C4 H4 O4	116.011	0.603	C00122	MReference - 1274	HMDB0000134	0	0.044	39.4	39.3	3	level3	Organic acids and derivatives	Carboxylic acids and derivatives	Dicarboxylic acids and derivatives	Organic acids	map00020 Citrate cycle (TCA cycle); map00190 Oxidative phosphorylation; map00220 Arginine biosynthesis; map00250 Alanine, aspartate and glutamate metabolism; map00350 Tyrosine metabolism; map00360 Phenylalanine metabolism; map00620 Pyruvate metabolism; map00650 Butanoate metabolism; map00760 Nicotinate and nicotinamide metabolism; map01100 Metabolic pathways; map01200 Carbon metabolism; [illegible]	other
0.604_88.0161	1.4406	0.0767	Pyruvic acid	C3 H4 O3	88.0161	0.604	C00022	MReference - 1264	HMDB0000243	0	0.1409		85.3	6	level2	Organic acids and derivatives	Keto acids and derivatives	Alpha-keto acids and derivatives	Organic acids	map00010 Glycolysis / Gluconeogenesis; map00020 Citrate cycle (TCA cycle); map00030 Pentose phosphate pathway; map00040 Pentose and glucuronate interconversions; map00053 Ascorbate and aldarate metabolism; map00250 Alanine, aspartate and glutamate metabolism; map00260 Glycine, serine and threonine metabolism; map00270 Cysteine and methionine metabolism; map00290 Valine, leucine and isoleucine biosynthesis; map00330 Arginine [illegible]	other
0.606_147.0532	0.5233	0.2394	L-glutamic acid	C5 H9 N O4	147.0532	0.606	C00025	MReference - 470	HMDB0000148	0.0001	0.4274	78.2	88.8	13	level1	Organic acids and derivatives	Carboxylic acids and derivatives	Amino acids, peptides, and analogues	Amino acids	map00220 Arginine biosynthesis; map00250 Alanine, aspartate and glutamate metabolism; map00330 Arginine and proline metabolism; map00340 Histidine metabolism; map00430 Taurine and hypotaurine metabolism; map00471 D-Glutamine and D-glutamate metabolism; map00480 Glutathione metabolism; map00524 Neomycin, kanamycin and gentamicin biosynthesis; map00630 Glyoxylate and [illegible]	other
0.606_235.8665	1.4671	0.2341		C H O6 P S3	235.8665	0.606								0	level5						other
0.609_133.0376	0.4811	0.0555	L-aspartate	C4 H7 N O4	133.0376	0.609	C16433	BGI345		0.0001	0.5485	97.5	62.3	4	level1					NULL	other
0.609_177.908	1.0665	0.7646		C H4 Cl O2 P S2	177.908	0.609								0	level5						other
0.61_136.0372	0.9932	0.9495	Threonic acid, l-	C4 H8 O5	136.0372	0.61		S388628		0	0.2749	99.2	94	5	level4						other
0.61_245.8952	0.9743	0.7032		C7 H2 Cl O2 P3	245.8952	0.61								0	level5						other
0.611_166.0478	0.7054	0.5351	D-xylonic acid	C5 H10 O6	166.0478	0.611	C00502	S5034782	HMDB0059750	0	0.0688			9	level4	Organic oxygen compounds	Organooxygen compounds	Carbohydrates and carbohydrate conjugates	Carbohydrates	map00040 Pentose and glucuronate interconversions; map01100 Metabolic pathways;	other
0.611_205.0118	0.5576	0.3724		C8 H3 N3 O4	205.0118	0.611								0	level5						other
0.612_226.9938	0.5092	0.3283		C4 H6 Cl N3 O6	226.9938	0.612								0	level5						other
0.613_293.8252	1.3054	0.2709			293.8252	0.613								0	level5						other
0.622_327.767	1.0078	0.944			327.767	0.622								0	level5						other
0.623_271.8051	0.9894	0.8745			271.8051	0.623								0	level5						other
0.625_213.8467	0.9926	0.8944			213.8467	0.625								0	level5						other
0.627_395.7545	1.0432	0.5255	[similar to: d-(+)-maltose; δ mass: 53.6383 da]		395.7545	0.627								0	level5						other
0.627_453.7129	1.063	0.2817			453.7129	0.627								0	level5						other
0.627_455.71	1.069	0.351			455.71	0.627								0	level5						other
0.628_337.7958	1.0157	0.8133		C3 H2 Cl N2 O P3 S4	337.7958	0.628								0	level5						other
0.629_335.7988	1.0661	0.1985			335.7988	0.629								0	level5						other
0.63_277.8398	1.0738	0.0623			277.8398	0.63								0	level5						other
0.63_279.8369	1.0431	0.507			279.8369	0.63								0	level5						other
0.63_405.7834	1.0862	0.2616	1,2,3,4,6,7,8-heptachlorodibenzofuran	C12 H Cl7 O	405.7834	0.63	C18111	S35019		-0.0014	-3.3542			2	level4						other
0.63_463.7417	1.0912	0.3772			463.7417	0.63								0	level5						other
0.631_117.991	0.8037	0.6591	[similar to: docosahexaenoyl ethanolamide; δmass: -253.2914 da]		117.991	0.631								0	level5						other
0.631_295.8111	0.8393	0.1501		C3 H4 Cl P3 S4	295.8111	0.631								0	level5						other

0.632_347.8246	1.0409	0.8008		C4 H2 Cl N4 O P S5	347.8246	0.632								0	level5						other
0.633_219.8813	0.8784	0.0277			219.8813	0.633								0	level5						other
0.633_221.8784	0.9866	0.7822		C5 H2 Cl P3 S	221.8784	0.633								0	level5						other
0.633_345.8275	1.089	0.4018			345.8275	0.633								0	level5						other
0.633_521.6998	1.1824	0.0732			521.6998	0.633								0	level5						other
0.634_163.0013	0.7337	0.099		C6 H N3 O3	163.0013	0.634								0	level5						other
0.635_182.9734	0.4567	0.03			182.9734	0.635								0	level5						down
0.635_289.8657	0.8453	0.1707		C2 H Cl N4 O3 P2 S2	289.8657	0.635								0	level5						other
0.636_161.9227	0.9718	0.6484			161.9227	0.636								0	level5						other
0.636_381.822	0.5541	0.2781		C5 H6 Cl N2 O2 P3 S4	381.822	0.636								0	level5						other
0.637_163.9199	0.9563	0.621	Chloral hydrate	C2 H3 Cl3 O2	163.9199	0.637	C06899	S2606	HMDB0060451	0	-0.0205			1	level4	Organohalogen compounds	Halohydrins	Chlorohydrins	Chlorohydrins	map00980 Metabolism of xenobiotics by cytochrome P450; map05204 Chemical carcinogenesis;	other
0.637_287.8687	0.9694	0.6967			287.8687	0.637								0	level5						other
0.638_323.8633	0.6037	0.2595		C9 H5 Cl O S5	323.8633	0.638								0	level5						other
0.639_321.8662	0.7635	0.4398			321.8662	0.639								0	level5						other
0.639_389.8537	0.6872	0.2884		C H N2 O16 P3	389.8537	0.639								0	level5						other
0.64_125.0147	0.4968	0.0899	Taurine	C2 H7 N O3 S	125.0147	0.64	C00245	S1091	HMDB0000251	0	-0.0216			1	level4	Organic acids and derivatives	Organic sulfonic acids and derivatives	Organosulfonic acids and derivatives	Organic acids	map00120 Primary bile acid biosynthesis; map00430 Taurine and hypotaurine metabolism; map00920 Sulfur metabolism; map01100 Metabolic pathways; map02010 ABC transporters; map04080 Neuroactive ligand-receptor interaction;	other
0.64_263.9074	0.5851	0.3934		C3 H6 O6 P2 S2	263.9074	0.64								0	level5						other
0.64_391.8507	0.849	0.5107		C7 H5 Cl N2 O5 S5	391.8507	0.64								0	level5						other
0.642_205.9488	0.6605	0.3362			205.9488	0.642								0	level5						other
0.642_331.8951	0.6497	0.312		C3 H3 N4 O7 P3 S	331.8951	0.642								0	level5						other
0.643_377.9188	0.4594	0.3966		C4 H6 Cl N6 O7 P3	377.9188	0.643								0	level5						other
0.644_204.0278	1.0022	0.8387	1-oxo-1,2,4-butanetricarboxylic acid	C7 H8 O7	204.0278	0.644	C05533	S389591		0.0008	4.0421			2	level4					map01210 2-Oxocarboxylic acid metabolism;	other
0.644_258.0827	0.6609	0.0556		C8 H15 N6 P S	258.0827	0.644								0	level5						other
0.644_273.9362	0.6934	0.3237		C5 H7 O7 P S2	273.9362	0.644								0	level5						other
0.644_399.8826	0.7119	0.4766		C8 H15 Cl O2 P2 S5	399.8826	0.644								0	level5						other
0.645_146.0692	0.8441	0.6675	L-glutamine	C5 H10 N2 O3	146.0692	0.645	C00064	BGI357	HMDB0000641	0.0001	0.6337	77.9	71.7	6	level1	Peptides	Amino acids	Common amino acids [Fig]	Amino acids	map00220 Arginine biosynthesis; map00230 Purine metabolism; map00240 Pyrimidine metabolism; map00250 Alanine, aspartate and glutamate metabolism; map00471 D-Glutamine and D-glutamate metabolism; map00630 Glyoxylate and dicarboxylate metabolism; map00910 Nitrogen metabolism; map00970 Aminoacyl-tRNA biosynthesis; map01100 Metabolic pathways; map01230 Biosynthesis of [illegible]	other
0.645_317.9629	0.5239	0.4265		C5 H11 Cl N6 S4	317.9629	0.645								0	level5						other
0.646_147.9904	0.7136	0.3719			147.9904	0.646								0	level5						other
0.647_163.9643	0.4865	0.1927			163.9643	0.647								0	level5						other
0.647_202.0454	0.5986	0.3309			202.0454	0.647								0	level5						other
0.647_229.91	0.9163	0.4612			229.91	0.647								0	level5						other
0.647_260.0037	0.4979	0.2664		C5 H9 Cl N2 O8	260.0037	0.647								0	level5						other
0.647_341.924	0.7645	0.3903		C5 H11 Cl N2 O5 S4	341.924	0.647								0	level5						other
0.647_467.8695	0.6595	0.1023		C3 H7 N2 O17 P3 S	467.8695	0.647								0	level5						other
0.648_215.9779	0.701	0.3052		C5 H10 Cl O3 P S	215.9779	0.648								0	level5						other
0.648_327.9915	0.4991	0.2967		C6 H12 Cl N6 O2 P3	327.9915	0.648								0	level5						other
0.649_150.0528	1.2886	0.4546	D-ribose	C5 H10 O5	150.0528	0.649	C00121	BGI247	HMDB0000283	0	-0.0994	85.2	82.2	39	level1	Carbohydrates	Monosaccharides	Aldoses [Fig]	Carbohydrates	map00030 Pentose phosphate pathway; map01100 Metabolic pathways; map02010 ABC transporters;	other
0.649_177.0169	1.504	0.2994		C7 H3 N3 O3	177.0169	0.649								0	level5						other

0.649_270.0327	0.5112	0.2423		C10 H12 N2 O3 P2	270.0327	0.649								0	level5						other
0.649_297.8975	1.0602	0.8842		C5 H12 Cl P S5	297.8975	0.649								0	level5						other
0.649_409.9114	0.6287	0.16		C10 H16 Cl O3 P S5	409.9114	0.649								0	level5						other
0.649_463.9663	0.5377	0.3005		C8 H11 Cl N6 O11 P2	463.9663	0.649								0	level5						other
0.649_521.9243	0.615	0.4561		C7 H13 Cl N4 O15 P2 S	521.9243	0.649								0	level5						other
0.65_231.0718	1.1702	0.6172		C7 H14 N5 P S	231.0718	0.65								0	level5						other
0.65_247.9884	0.697	0.2832		C4 H7 N6 O P3	247.9884	0.65								0	level5						other
0.65_283.965	0.6986	0.2847		C4 H6 Cl N6 O3 P S	283.965	0.65								0	level5						other
0.65_338.0203	0.517	0.2792		C7 H13 N6 O4 P3	338.0203	0.65								0	level5						other
0.65_395.9791	0.5093	0.2878		C9 H10 Cl N6 O6 P S	395.9791	0.65								0	level5						other
0.651_237.9595	0.7143	0.3132		C3 H4 Cl N6 O P S	237.9595	0.651								0	level5						other
0.651_90.0091	0.4768	0.1606			90.0091	0.651								0	level5						other
0.652_305.9472	0.7348	0.2757		C5 H8 Cl N2 O7 P S	305.9472	0.652								0	level5						other
0.652_477.8987	0.7053	0.084		C5 H7 Cl N4 O16 S2	477.8987	0.652								0	level5						other
0.653_158.0191	0.7085	0.3079	[similar to: muramic acid; δmass: -93.0814 da]		158.0191	0.653								0	level5						other
0.653_230.0765	1.3626	0.7146		C6 H10 N6 O4	230.0765	0.653								0	level5						other
0.653_315.9761	0.7171	0.1579	Lythidathion	C7 H13 N2 O4 P S3	315.9761	0.653	C19004	S16622		-0.0014	-4.4925			1	level4					NULL	other
0.653_373.9348	0.6712	0.1852		C5 H5 Cl N6 O8 P2	373.9348	0.653								0	level5						other
0.654_180.0011	0.692	0.1928		C5 H9 O3 P S	180.0011	0.654								0	level5						other
0.654_180.0634	1.2237	0.215	D-tagatose	C6 H12 O6	180.0634	0.654	C00795	BGI252		0	0.2099	95.7	95.4	58	level1	Carbohydrates	Monosaccharides	Ketoses [Fig]	Carbohydrates	map00052 Galactose metabolism; map01100 Metabolic pathways;	other
0.654_226.0065	0.6655	0.1118		C4 H6 N2 O9	226.0065	0.654								0	level5						other
0.654_293.9939	0.6353	0.2083		C5 H7 N6 O5 P S	293.9939	0.654								0	level5						other
0.654_419.9402	0.6168	0.1314		C6 H7 Cl N6 O10 P2	419.9402	0.654								0	level5						other
0.655_187.0457	1.0837	0.7385		C11 H9 N S	187.0457	0.655								0	level5						other
0.655_270.0949	1.0537	0.704		C9 H18 O9	270.0949	0.655								0	level5						other
0.656_487.9275	0.7539	0.1034		C5 H13 Cl N2 O18 S2	487.9275	0.656								0	level5						other
0.658_119.0583	1.4923	0.2982	L-threonine	C4 H9 N O3	119.0583	0.658	C00188	BGI380	HMDB0000167	0.0001	0.4675	68.2	58.3	10	level1	Peptides	Amino acids	Common amino acids [Fig]	Amino acids	map00260 Glycine, serine and threonine metabolism; map00290 Valine, leucine and isoleucine biosynthesis; map00860 Porphyrin and chlorophyll metabolism; map00970 Aminoacyl-tRNA biosynthesis; map01100 Metabolic pathways; map01230 Biosynthesis of amino acids; map02010 ABC transporters; map04974 Protein digestion and absorption; map04978 Mineral absorption;	other
0.658_351.9527	0.6918	0.128		C6 H10 Cl N2 O9 P S	351.9527	0.658								0	level5						other
0.659_175.0957	1.1018	0.7539	Dl-citrulline	C6 H13 N3 O3	175.0957	0.659		S810		0	0.175			3	level4						other
0.662_120.0423	1.3556	0.085	D-(−)-threose	C4 H8 O4	120.0423	0.662		S388736		0	0.0297			11	level4						other
0.663_361.9816	0.7167	0.2455		C7 H11 N2 O11 P S	361.9816	0.663								0	level5						other
0.665_158.0439	0.518	0.2801	Allantoin	C4 H6 N4 O3	158.0439	0.665	C01551	S199	HMDB0000462	-0.0001	-0.6795			3	level4	Organoheterocyclic compounds	Azoles	Imidazoles	Imidazoles	NULL	other
0.667_72.0211	1.5646	0.0681	Acrylic acid	C3 H4 O2	72.0211	0.667	C00511	MReference-2696	HMDB0031647	0	0.0589		100	5	level2	Organic acids and derivatives	Carboxylic acids and derivatives	Acrylic acids and derivatives	Organic acids		other
0.668_216.0028	0.5368	0.157	D-4-phosphoerythronic acid	C4 H9 O8 P	216.0028	0.668	C03393	S395873		-0.0007	-3.2694			3	level4					map00750 Vitamin B6 metabolism; map01100 Metabolic pathways;	other
0.671_132.0423	1.3477	0.1873	Glutaric acid	C5 H8 O4	132.0423	0.671	C00489	S723	HMDB0000661	0	0.1246			17	level4	Organic acids and derivatives	Carboxylic acids and derivatives	Dicarboxylic acids and derivatives	Organic acids	map00071 Fatty acid degradation; map00310 Lysine degradation; map01100 Metabolic pathways;	other
0.673_156.0036	2.3301	0.13			156.0036	0.673								0	level5						other
0.673_187.0012	0.4517	0.1081		C3 H10 Cl N3 S2	187.0012	0.673								0	level5						other
0.673_200.0298	1.6537	0.1125		C4 H4 N6 O4	200.0298	0.673								0	level5						other
0.674_129.0426	0.5221	0.2119	L-pyroglutamic acid	C5 H7 N O3	129.0426	0.674	C01879	S7127	HMDB0000267	0	0.3796			10	level4	Organic acids and derivatives	Carboxylic acids and derivatives	Amino acids, peptides, and analogues	Amino acids, peptides, and analogues	map00480 Glutathione metabolism; map01100 Metabolic pathways;	other
0.674_197.0301	0.4933	0.2104		C4 H13 N3 P2 S	197.0301	0.674								0	level5						other
0.675_145.9747	1.775	0.0996	[similar to: d-(-)-glutamine; δ mass: -0.0944 da]		145.9747	0.675								0	level5						other
0.675_223.0568	0.9937	0.9691		C6 H16 N3 P3	223.0568	0.675								0	level5						other

0.675_268.0806	2.5174	0.2205	2-(alpha-d-mannosyl)-d-glyceric acid	C9 H16 O9	268.0806	0.675	C11544	S4573823		0.0011	4.1684			4	level4					map00051 Fructose and mannose metabolism; map01100 Metabolic pathways;	other
0.676_155.0696	1.0011	0.8557	L-histidine	C6 H9 N3 O2	155.0696	0.676	C00135	MReference-473	HMDB0000177	0.0001	0.587		82.8	5	level2	Organic acids and derivatives	Carboxylic acids and derivatives	Amino acids, peptides, and analogues	Amino acids	map00340 Histidine metabolism; map00410 beta-Alanine metabolism; map00970 Aminoacyl-tRNA biosynthesis; map01100 Metabolic pathways; map01230 Biosynthesis of amino acids; map02010 ABC transporters; map04974 Protein digestion and absorption; map05230 Central carbon metabolism in cancer;	other
0.676_189.0282	0.664	0.287		C6 H7 N O6	189.0282	0.676								0	level5						other
0.676_199.057	0.7334	0.4324			199.057	0.676								0	level5						other
0.676_213.0282	1.1334	0.1869		C8 H7 N O6	213.0282	0.676								0	level5						other
0.678_89.0478	0.9956	0.954	B-alanine	C3 H7 N O2	89.0478	0.678	C00099	MReference-599	HMDB0000056	0.0001	1.0746		37.1	8	level3	Organic acids and derivatives	Carboxylic acids and derivatives	Amino acids, peptides, and analogues	Amino acids, peptides, and analogues	map00240 Pyrimidine metabolism; map00410 beta-Alanine metabolism; map00640 Propanoate metabolism; map00770 Pantothenate and CoA biosynthesis; map01100 Metabolic pathways; map04080 Neuroactive ligand-receptor interaction; map04974 Protein digestion and absorption;	other
0.681_174.1005	0.9343	0.7296	N-acetylornithine	C7 H14 N2 O3	174.1005	0.681		MReference-513		0	0.1181		39.2	6	level3						other
0.682_214.0454	1.9662	0.1367		C13 H10 O S	214.0454	0.682								0	level5						other
0.685_103.9642	1.0713	0.7111	[similar to: 4-hydroxybutyric acid (ghb); δ mass: -0.0832 da]		103.9642	0.685								0	level5						other
0.685_190.0478	0.9285	0.5791	3-dehydroquinic acid	C7 H10 O6	190.0478	0.685	C00944	S388474	HMDB0012710	0.0001	0.3274			5	level4	Organic acids and derivatives	Hydroxy acids and derivatives	Alpha hydroxy acids and derivatives	Organic acids	map00400 Phenylalanine, tyrosine and tryptophan biosynthesis; map01100 Metabolic pathways; map01230 Biosynthesis of amino acids;	other
0.685_257.0665	0.9341	0.5143		C8 H16 Cl N O6	257.0665	0.685								0	level5						other
0.686_183.0507	1.457	0.3076			183.0507	0.686								0	level5						other
0.687_172.0349	1.4724	0.0724		C3 H4 N6 O3	172.0349	0.687								0	level5						other
0.687_173.022	1.4104	0.3698		C8 H3 N3 O2	173.022	0.687								0	level5						other
0.688_162.006	1.4939	0.0547	2-chloro-1-ethoxy-1,1,2-trifluoroethane	C4 H6 Cl F3 O	162.006	0.688	C14700	S119928		0	0.2755			1	level4					NULL	other
0.69_115.0633	1.2542	0.4942	L-proline	C5 H9 N O2	115.0633	0.69	C00148	S128566	HMDB0000162	0	-0.2218			6	level4	Organic acids and derivatives	Carboxylic acids and derivatives	Amino acids, peptides, and analogues	Amino acids	map00330 Arginine and proline metabolism; map00970 Aminoacyl-tRNA biosynthesis; map01100 Metabolic pathways; map01230 Biosynthesis of amino acids; map02010 ABC transporters; map04974 Protein digestion and absorption; map04978 Mineral absorption; map05230 Central carbon metabolism in cancer;	other
0.69_202.0649	0.1398	0.4823	[similar to: lignoceric acid; δmass: -166.3006 da]		202.0649	0.69								0	level5						other
0.69_216.0609	1.419	0.0159		C5 H8 N6 O4	216.0609	0.69								0	level5						up
0.69_284.1106	1.974	0.0912		C10 H20 O9	284.1106	0.69								0	level5						other

0.69_311.1217	0.667	0.4823	N2-dimethylguanosine	C12 H17 N5 O5	311.1217	0.69		S83878	HMDB0004824	-0.0013	-4.1812			2	level4	Nucleosides, nucleotides, and analogues	Purine nucleosides	null	Purines and derivatives		other
0.694_355.8563	0.9502	0.9647			355.8563	0.694								0	level5						other
0.695_169.0852	1.1556	0.85	N.pi.-methyl-l-histidine	C7 H11 N3 O2	169.0852	0.695	C01152	S58494	HMDB0000479	0	0.2733			6	level4	Organic acids and derivatives	Carboxylic acids and derivatives	Amino acids, peptides, and analogues	Amino acids, peptides, and analogues	map00340 Histidine metabolism; map01100 Metabolic pathways;	other
0.695_204.0455	1.0415	0.9357		C8 H12 O4 S	204.0455	0.695								0	level5						other
0.697_214.0566	1.1404	0.4625	1-methyl-3-(2-thiazolyl)-1h-indole	C12 H10 N2 S	214.0566	0.697		S9450932	HMDB0034965	0.0001	0.6877			1	level4	Organoheterocyclic compounds	Indoles and derivatives	N-alkylindoles	Indole and derivatives		other
0.702_278.0808	0.1519	0.1045		C15 H10 N4 O2	278.0808	0.702								0	level5						other
0.703_187.0529	0.1754	0.0684			187.0529	0.703								0	level5						other
0.704_188.0493	0.1724	0.1524			188.0493	0.704								0	level5						other
0.706_113.059	1.0102	0.9193	Creatinine	C4 H7 N3 O	113.059	0.706	C00791	BGI197	HMDB0000562	0.0001	0.608	87.1		2	level1	Organic acids and derivatives	Carboxylic acids and derivatives	Amino acids, peptides, and analogues	Amino acids, peptides, and analogues	map00330 Arginine and proline metabolism; map01100 Metabolic pathways;	other
0.706_229.91	1.0928	0.7443			229.91	0.706								0	level5						other
0.708_297.8975	0.881	0.6715		C5 H12 Cl P S5	297.8975	0.708								0	level5						other
0.71_239.9389	0.818	0.0348		C2 H4 Cl N6 P3	239.9389	0.71								0	level5						down
0.711_171.9516	0.898	0.554	2-bromophenol	C6 H5 Br O	171.9516	0.711	C14841	S6974	HMDB0032059	-0.0008	-4.4938			4	level4	Benzenoids	Phenols	Halophenols	Phenols and derivatives	map00980 Metabolism of xenobiotics by cytochrome P450;	other
0.712_116.0281	0.2163	0.1374	[similar to: fumaric acid; δmass: 0.0171 da]		116.0281	0.712								0	level5						other
0.713_147.9904	0.7461	0.353	[similar to: sucrose; δ mass: -194.1258 da]		147.9904	0.713								0	level5						other
0.713_251.842	0.9612	0.7124			251.842	0.713								0	level5						other
0.713_365.8853	0.6045	0.0084		C4 H8 Cl N6 P S5	365.8853	0.713								0	level5						down
0.715_216.0401	1.1204	0.8402	2-c-methylerythritol 4-phosphate	C5 H13 O7 P	216.0401	0.715	C11434	S391470		0.0002	0.9683	58.4		1	level4					map00900 Terpenoid backbone biosynthesis; map01100 Metabolic pathways;	other
0.716_175.0376	1.285	0.2949		C8 H5 N3 O2	175.0376	0.716								0	level5						other
0.716_317.9552	0.8382	0.2684	Mitotane	C14 H10 Cl4	317.9552	0.716		S4066	HMDB0014786	0.0015	4.7928			3	level4	Benzenoids	Benzene and substituted derivatives	Diphenylmethanes	Benzene and derivatives		other
0.716_341.9239	0.7233	0.4755		C5 H11 Cl N2 O5 S4	341.9239	0.716								0	level5						other
0.716_385.9423	0.9655	0.9208		C6 H8 N6 O6 P2 S2	385.9423	0.716								0	level5						other
0.717_259.8737	0.92	0.4099			259.8737	0.717								0	level5						other
0.717_297.1423	1.8166	0.1503		C11 H23 N O8	297.1423	0.717								0	level5						other
0.717_307.9264	1.1159	0.5729		C5 H7 Cl O9 P2	307.9264	0.717								0	level5						other
0.718_249.9676	1.3104	0.2969		C3 H5 N6 O2 P3	249.9676	0.718								0	level5						other
0.718_433.8725	0.7854	0.0335		C4 H7 Cl N4 O10 S4	433.8725	0.718								0	level5						down
0.719_229.0927	1.148	0.4297		C14 H15 N S	229.0927	0.719								0	level5						other
0.719_269.9026	1.3251	0.0821		C2 H7 Cl N2 O3 S4	269.9026	0.719								0	level5						other
0.721_218.037	2.4667	0.0913		C7 H12 N2 O2 P2	218.037	0.721								0	level5						other
0.721_375.9139	1.1729	0.2267		C8 H7 Cl O11 P2	375.9139	0.721								0	level5						other
0.721_443.9012	0.7882	0.0086		C6 H5 Cl N2 O17 S	443.9012	0.721								0	level5						down
0.722_158.0191	0.808	0.5037	[similar to: lignoceric acid; δmass: -210.3463 da]		158.0191	0.722								0	level5						other
0.722_90.0317	0.9135	0.7144	Dl-lactic acid	C3 H6 O3	90.0317	0.722	C01432	MReference-2603	HMDB0144295	0	0.2401		99.7	11	level2	Organic acids and derivatives	Hydroxy acids and derivatives	Alpha hydroxy acids and derivatives	Organic acids	NULL	other
0.724_180.0634	1.57	0.2029	D-(-)-fructose	C6 H12 O6	180.0634	0.724	C02336	MReference-1478	HMDB0000660	0.0001	0.2817	94.5	95.8	58	level2	Organic oxygen compounds	Organooxygen compounds	Carbohydrates and carbohydrate conjugates	Carbohydrates	map00520 Amino sugar and nucleotide sugar metabolism;	other
0.724_181.9804	1.1352	0.5416		C4 H7 O4 P S	181.9804	0.724								0	level5						other
0.725_185.0664	1.2924	0.2266	[similar to: n-acetylvaline; δmass: 25.9769 da]		185.0664	0.725								0	level5						other
0.726_226.0688	1.0371	0.9347	Glucoheptonic acid	C7 H14 O8	226.0688	0.726		S55750	HMDB0240292	0	-0.1661		56.1	1	level4	Organic oxygen compounds	Organooxygen compounds	Carbohydrates and carbohydrate conjugates	Carbohydrates		other
0.726_270.0949	0.9776	0.876		C9 H18 O9	270.0949	0.726								0	level5						other
0.726_279.9313	1.7567	0.0265		C5 H3 Cl N4 O4 P2	279.9313	0.726								0	level5						up
0.733_162.0529	1.4476	0.1979	Diethylpyrocarbonate	C6 H10 O5	162.0529	0.733	C11592	S2943	HMDB0032873	0.0001	0.3539	75.9		31	level4	Organic acids and derivatives	Organic carbonic acids and derivatives	null	Organic acids	NULL	other
0.735_216.061	0.3518	0.1434		C13 H12 O S	216.061	0.735								0	level5						other
0.737_132.0899	1.6792	0.5414	Ornithine	C5 H12 N2 O2	132.0899	0.737	C01602	MReference-531		0	0.0763		35.5	4	level3					NULL	other
0.74_114.0317	1.1849	0.7787	4-hydroxy-5-methylfuran-3(2h)-one	C5 H6 O3	114.0317	0.74		S3757733	HMDB0031859	0	0.2002			4	level4	Organoheterocyclic compounds	Dihydrofurans	Furanones	Furanones		other

0.744_112.0273	0.4062	0.3546	Uracil	C4 H4 N2 O2	112.0273	0.744	C00106	S1141	HMDB0000300	0	0.3133			3	level4	Organoheterocyclic compounds	Diazines	Pyrimidines and pyrimidine derivatives	Pyrimidines and pyrimidine derivatives	map00240 Pyrimidine metabolism; map00410 beta-Alanine metabolism; map00770 Pantothenate and CoA biosynthesis; map01100 Metabolic pathways;	other
0.744_174.1117	0.975	0.888	Dl-arginine	C6 H14 N4 O2	174.1117	0.744	C02385	MReference-408		0	0.1825		94.5	3	level2					NULL	other
0.744_221.9728	0.9042	0.4567		C2 H3 N6 O3 P S	221.9728	0.744								0	level5						other
0.745_117.079	2.2286	0.0345	L-(+)-valine	C5 H11 N O2	117.079	0.745	C00183	S6050	HMDB0000883	0	0.0175			15	level4	Organic acids and derivatives	Carboxylic acids and derivatives	Amino acids, peptides, and analogues	Amino acids	map00280 Valine, leucine and isoleucine degradation; map00290 Valine, leucine and isoleucine biosynthesis; map00770 Pantothenate and CoA biosynthesis; map00970 Aminoacyl-tRNA biosynthesis; map01100 Metabolic pathways; map01210 2-Oxocarboxylic acid metabolism; map01230 Biosynthesis of amino acids; map02010 ABC transporters; map04974 Protein digestion and absorption; map04978 Mineral [illegible]	up
0.746_147.0897	1.289	0.5074	Afegostat	C6 H13 N O3	147.0897	0.746		S394649		0.0001	0.6974			7	level4						other
0.754_226.0065	0.7375	0.6436		C4 H6 N2 O9	226.0065	0.754								0	level5						other
0.757_277.0845	0.1852	0.35			277.0845	0.757								0	level5						other
0.757_289.9602	1.1652	0.3351		C4 H7 N2 O9 P S	289.9602	0.757								0	level5						other
0.764_102.0317	3.1596	0.2087	2-oxobutyric acid	C4 H6 O3	102.0317	0.764	C00109	MReference-180	HMDB0000005	0	0.1913		55.1	8	level3	Organic acids and derivatives	Keto acids and derivatives	Short-chain keto acids and derivatives	Organic acids	map00260 Glycine, serine and threonine metabolism; map00270 Cysteine and methionine metabolism; map00290 Valine, leucine and isoleucine biosynthesis; map00640 Propanoate metabolism; map01100 Metabolic pathways; map01210 2-Oxocarboxylic acid metabolism; map01230 Biosynthesis of amino acids;	other
0.765_278.0808	0.1433	0.2236		C7 H14 N6 O4 S	278.0808	0.765								0	level5						other
0.765_329.1146	1.8036	0.2623		C11 H25 N O6 P2	329.1146	0.765								0	level5						other
0.771_294.0562	1.3409	0.038	2-hydroxy-1,2-diphenylethyl hydrogen sulfate	C14 H14 O5 S	294.0562	0.771		S74853754	HMDB0135202	0	0.0842			1	level4	Phenylpropanoids and polyketides	Stilbenes	null	Polyketides[PK]		up
0.774_188.0493	0.0586	0.0603			188.0493	0.774								0	level5						other
0.775_249.0612	1.361	0.221		C9 H18 N O P3	249.0612	0.775								0	level5						other
0.777_217.0385	1.7874	0.2488			217.0385	0.777								0	level5						other
0.782_367.1165	0.3667	0.4417			367.1165	0.782								0	level5						other
0.784_368.1127	0.4073	0.5447		C16 H21 N2 O6 P	368.1127	0.784								0	level5						other
0.816_159.9858	3.7374	0.0108		C H4 O9	159.9858	0.816								0	level5						up
0.861_309.1092	7.7855	0.0339	4-carboxy-2-(tyrosylamino)butanoate	C14 H17 N2 O6	309.1092	0.861		S35031938		0.0005	1.7606			2	level4						up
0.932_149.0512	2.0544	0.15	L-(-)-methionine	C5 H11 N O2 S	149.0512	0.932	C00073	S5907	HMDB0000696	0.0001	0.7216			5	level4	Organic acids and derivatives	Carboxylic acids and derivatives	Amino acids, peptides, and analogues	Amino acids	map00270 Cysteine and methionine metabolism; map00970 Aminoacyl-tRNA biosynthesis; map01100 Metabolic pathways; map01210 2-Oxocarboxylic acid metabolism; map01230 Biosynthesis of amino acids; map01523 Antifolate resistance; map04974 Protein digestion and absorption; map04978 Mineral absorption; map05230 Central carbon metabolism in cancer;	other
0.94_178.03	1.3976	0.1245	Uf7990000	C6 H10 O4 S	178.03	0.94		S7805	HMDB0031162	0.0001	0.3967			1	level4	Organic acids and derivatives	Carboxylic acids and derivatives	Dicarboxylic acids and derivatives	Organic acids		other
0.966_189.1001	3.8233	0.0587	(+)-castanospermine	C8 H15 N O4	189.1001	0.966	C02256	S49177		0	-0.1549			12	level4	Alkaloids	Alkaloids derived from lysine	Indolizidine alkaloids	Alkaloids	NULL	other

0.966_279.1316	4.2177	0.0785	(2s)-3-methyl-2-({[(3s,4s,5r)-2,3,4-trihydroxy-5-(hydroxymethyl)tetrahydro-2-furanyl]methyl}amino)butanoic acid (non-preferred name)	C11 H21 N O7	279.1316	0.966		S35014481	HMDB0037844	-0.0002	-0.7568			1	level4	Organic acids and derivatives	Carboxylic acids and derivatives	Amino acids, peptides, and analogues	Amino acids, peptides, and analogues		other
0.973_159.9858	0.991	0.3834		C H4 O9	159.9858	0.973								0	level5						other
0.975_131.9909	0.8501	0.3346		C3 H5 N2 P S	131.9909	0.975								0	level5						other
1.009_220.0726	1.9512	0.3734	Eugenitin	C12 H12 O4	220.0726	1.009		S2340764	HMDB0029467	-0.001	-4.4975			5	level4	Organoheterocyclic compounds	Benzopyrans	1-benzopyrans	1-benzopyrans		other
1.029_181.0739	1.1701	0.5459	L-tyrosine	C9 H11 N O3	181.0739	1.029	C00082	MReference-2255	HMDB0000158	0	0.0595		92	15	level1	Organic acids and derivatives	Carboxylic acids and derivatives	Amino acids, peptides, and analogues	Amino acids	map00130 Ubiquinone and other terpenoid-quinone biosynthesis; map00350 Tyrosine metabolism; map00360 Phenylalanine metabolism; map00400 Phenylalanine, tyrosine and tryptophan biosynthesis; map00730 Thiamine metabolism; map00970 Aminoacyl-tRNA biosynthesis; map01100 Metabolic pathways; map01210 2-Oxocarboxylic acid metabolism; map01230 Biosynthesis of amino acids; [illegible]	other
1.033_159.9858	1.3955	0.1694		C H4 O9	159.9858	1.033								0	level5						other
1.037_131.9909	1.1853	0.4705		C3 H5 N2 P S	131.9909	1.037								0	level5						other
1.052_136.0385	0.5361	0.3915	Hypoxanthine	C5 H4 N4 O	136.0385	1.052	C00262	BGI318	HMDB0000157	0	0.2833	97	94.6	3	level1	Alkaloids	Others	Purine alkaloids	Alkaloids	map00230 Purine metabolism; map01100 Metabolic pathways;	other
1.125_152.0334	0.3813	0.2171	Xanthine	C5 H4 N4 O2	152.0334	1.125	C00385	MReference-781	HMDB0000292	0	-0.1948		88.7	3	level1	Organoheterocyclic compounds	Imidazopyrimidines	Purines and purine derivatives	Purines and derivatives	map00230 Purine metabolism; map00232 Caffeine metabolism; map01100 Metabolic pathways;	other
1.145_259.9987	0.6675	0.7264	Caffeic acid 3-sulfate	C9 H8 O7 S	259.9987	1.145		S30777605	HMDB0041706	-0.0004	-1.4306			7	level4	Phenylpropanoids and polyketides	Cinnamic acids and derivatives	Hydroxycinnamic acids and derivatives	Polyketides[PK]		other
1.147_228.0633	1.5211	0.3017	2-hydroxy-3,4,5-trimethoxybenzoic acid	C10 H12 O6	228.0633	1.147		S255670	HMDB0141334	-0.0001	-0.2885			8	level4	Benzenoids	Benzene and substituted derivatives	Benzoic acids and derivatives	Benzene and derivatives		other
1.164_147.0354	6.117	0.3106	3-thiomorpholinecarboxylic acid	C5 H9 N O2 S	147.0354	1.164	C03901	S389150	HMDB0059611	0	0.2035			1	level4	Organic acids and derivatives	Carboxylic acids and derivatives	Amino acids, peptides, and analogues	Amino acids, peptides, and analogues	NULL	other
1.203_182.0579	1.0255	0.8472	Dl-4-hydroxyphenyllactic acid	C9 H10 O4	182.0579	1.203	C03672	MReference-312	HMDB0000755	0	0.0701		89.2	32	level2	Phenylpropanoids and polyketides	Phenylpropanoic acids	null	Phenylpropanoic acids	NULL	other
1.215_137.9809	2.6792	0.192		C3 H6 O2 S2	137.9809	1.215								0	level5						other
1.218_280.046	2.4824	0.225		C9 H13 Cl N2 O6	280.046	1.218						91.9		0	level5						other
1.225_137.0476	0.6988	0.4374	Anthranilic acid	C7 H7 N O2	137.0476	1.225	C00108	MReference-113	HMDB0001123	-0.0001	-0.4666	76.7	76.8	11	level2	Benzenoids	Benzene and substituted derivatives	Benzoic acids and derivatives	Benzene and derivatives	map00380 Tryptophan metabolism; map00400 Phenylalanine, tyrosine and tryptophan biosynthesis; map01100 Metabolic pathways; map01230 Biosynthesis of amino acids;	other
1.239_131.0946	1.1848	0.4571	L-(+)-leucine	C6 H13 N O2	131.0946	1.239	C00123	S5880	HMDB0000687	0	-0.2119			18	level4	Organic acids and derivatives	Carboxylic acids and derivatives	Amino acids, peptides, and analogues	Amino acids	map00280 Valine, leucine and isoleucine degradation; map00290 Valine, leucine and isoleucine biosynthesis; map00970 Aminoacyl-tRNA biosynthesis; map01100 Metabolic pathways; map01210 2-Oxocarboxylic acid metabolism; map01230 Biosynthesis of amino acids; map02010 ABC transporters; map04150 mTOR signaling pathway; map04974 Protein digestion and absorption; map04978 Mineral absorption; [illegible]	other
1.24_134.1134	1.0622	0.6395			134.1134	1.24								0	level5						other

1.289_244.0693	3.0755	0.1725	Uridine	C9 H12 N2 O6	244.0693	1.289	C00299	BGI558	HMDB0000296	-0.0002	-1.0134	96.5	92	3	level1	Nucleic acids	Nucleosides [Fig]	Ribonucleosides	Nucleic acids	map00240 Pyrimidine metabolism; map01100 Metabolic pathways; map02010 ABC transporters;	other
1.292_219.1106	0.7539	0.2969	Pantothenic acid	C9 H17 N O5	219.1106	1.292	C00864	MReference-536	HMDB0000210	-0.0001	-0.3595		87	2	level2	Organic oxygen compounds	Organooxygen compounds	Alcohols and polyols	Alcohols	map00410 beta-Alanine metabolism; map00770 Pantothenate and CoA biosynthesis; map01100 Metabolic pathways; map04977 Vitamin digestion and absorption;	other
1.295_280.046	3.0357	0.1817		C9 H13 Cl N2 O6	280.046	1.295						92.7		0	level5						other
1.322_116.0473	0.9741	0.8904	Methyl acetoacetate	C5 H8 O3	116.0473	1.322		MReference-488	HMDB0000310	0	-0.1113		46	15	level3	Organic acids and derivatives	Keto acids and derivatives	Beta-keto acids and derivatives	Organic acids		other
1.703_164.0474	1.105	0.6284	4-coumaric acid	C9 H8 O3	164.0474	1.703	C00811	MReference-539	HMDB0002035	0	0.2918		90	21	level2	Phenylpropanoids and polyketides	Cinnamic acids and derivatives	Hydroxycinnamic acids and derivatives	Polyketides[PK]	map00130 Ubiquinone and other terpenoid-quinone biosynthesis; map00350 Tyrosine metabolism; map01100 Metabolic pathways;	other
1.736_152.0474	0.2066	0.2046	Dl-mandelic acid	C8 H8 O3	152.0474	1.736	C05852	MReference-1359	HMDB0000669	0	0.1141	58.3	98.2	37	level2	Benzenoids	Benzene and substituted derivatives	Phenylacetic acids	Benzene and derivatives	map00360 Phenylalanine metabolism; map01100 Metabolic pathways;	other
1.811_203.1158	2.8622	0.1758	Acetylcarnitine	C9 H17 N O4	203.1158	1.811		S21243783		0.0001	0.434			3	level4						other
1.817_293.1474	2.7147	0.1863	(2s)-3-methyl-2-({[(3s,4s,5r)-2,3,4-trihydroxy-5-(hydroxymethyl)tetrahydro-2-furanyl]methyl}amino)pentanoic acid (non-preferred name)	C12 H23 N O7	293.1474	1.817		S35014874	HMDB0039780	-0.0001	-0.3397			2	level4	Organic acids and derivatives	Carboxylic acids and derivatives	Amino acids, peptides, and analogues	Amino acids, peptides, and analogues		other
1.836_163.9897	0.848	0.9671		C3 H6 N2 O2 P2	163.9897	1.836								0	level5						other
1.866_189.0096	1.0247	0.8486	Lanthionine ketimine	C6 H7 N O4 S	189.0096	1.866		S118424	HMDB0004823	0	0.1514			3	level4	Organic acids and derivatives	Carboxylic acids and derivatives	Amino acids, peptides, and analogues	Amino acids, peptides, and analogues		other
1.98_268.0806	2.1751	0.2481	Inosine	C10 H12 N4 O5	268.0806	1.98	C00294	BGI328	HMDB0000195	-0.0002	-0.8028	98.8		9	level1	Nucleosides, nucleotides, and analogues	Purine nucleosides	null	Purines and derivatives	map00230 Purine metabolism; map01100 Metabolic pathways; map02010 ABC transporters;	other
10.01_280.2402	0.6334	0.2833	Linoleic acid	C18 H32 O2	280.2402	10.01	C01595	MReference-2706	HMDB0000673	-0.0001	-0.2017		93.6	87	level2	Lipids and lipid-like molecules	Fatty Acyls	Lineolic acids and derivatives	Fatty acyls[FA]	map00591 Linoleic acid metabolism; map01040 Biosynthesis of unsaturated fatty acids; map01100 Metabolic pathways;	other
10.012_111.9831	0.7001	0.2848	Methyl bisulfate	C H4 O4 S	111.9831	10.012	C02704	S6172		0.0001	0.4616			1	level4					NULL	other
10.055_525.3426	0.5678	0.2168		C25 H52 N O8 P	525.3426	10.055								0	level5						other
10.096_555.3546	0.4923	0.1606		C27 H50 N5 O5 P	555.3546	10.096								0	level5						other
10.096_593.3698	0.2926	0.1621		C29 H56 N O9 P	593.3698	10.096								0	level5						other
10.12_509.3115	0.4665	0.0993	18-amino-15-hydroxy-15-oxido-9-oxo-10,14,16-trioxa-15lambda~5~-phosphaoctadecan-12-yl decanoate	C24 H48 N O8 P	509.3115	10.12		S7825745		-0.0002	-0.4813			2	level4						other
10.135_527.3583	0.4912	0.202		C26 H50 N5 O4 P	527.3583	10.135								0	level5						other
10.16_463.3063	0.3818	0.1524		C23 H46 N O6 P	463.3063	10.16								0	level5						other
10.197_306.256	0.4296	0.147	8z,11z,14z-eicosatrienoic acid	C20 H34 O2	306.256	10.197	C03242	MReference-349	HMDB0002925	0.0001	0.374		86.6	26	level2	Lipids and lipid-like molecules	Fatty Acyls	Fatty acids and conjugates	Fatty acyls[FA]	map00591 Linoleic acid metabolism; map01040 Biosynthesis of unsaturated fatty acids; map01100 Metabolic pathways;	other
10.203_637.3584	0.3766	0.0779		C31 H52 N5 O7 P	637.3584	10.203								0	level5						other
10.207_569.37	0.3758	0.0982		C27 H56 N O9 P	569.37	10.207								0	level5						other
10.223_553.3736	0.4487	0.1452		C27 H56 N O8 P	553.3736	10.223								0	level5						other
10.333_637.3585	0.7702	0.5437		C30 H56 N O11 P	637.3585	10.333								0	level5						other
10.337_569.37	0.4153	0.1358		C27 H56 N O9 P	569.37	10.337								0	level5						other

10.338_282.2558	0.5988	0.3431	Trans-petroselinic acid	C18 H34 O2	282.2558	10.338		MReference-9851		-0.0001	-0.2545		92.6	32	level2						other
10.359_549.3037	0.586	0.1445		C25 H50 N3 O4 P3	549.3037	10.359								0	level5						other
10.368_332.2717	0.5294	0.176	(7e,10e,13e,16e)-7,10,13,16-docosatetraenoic acid	C22 H36 O2	332.2717	10.368		S4445971		0.0002	0.5826			11	level4						other
2.154_132.0786	1.5526	0.3027	6-hydroxycaproic acid	C6 H12 O3	132.0786	2.154	C06103	MReference-1203	HMDB0012843	0	-0.0335		87.7	20	level2	Organic acids and derivatives	Hydroxy acids and derivatives	Medium-chain hydroxy acids and derivatives	Organic acids		other
2.189_207.0531	3.1098	0.1807	4-(2-aminophenyl)-2,4-dioxobutanoic acid	C10 H9 N O4	207.0531	2.189	C01252	S459	HMDB0000978	0	-0.1991			5	level4	Organic oxygen compounds	Organooxygen compounds	Carbonyl compounds	Carbonyl compounds	map00380 Tryptophan metabolism; map01100 Metabolic pathways;	other
2.242_208.0848	1.6021	0.1462	L-kynurenine	C10 H12 N2 O3	208.0848	2.242	C00328	BGI19	HMDB0000684	0	-0.1615	95.8		5	level1	Organic oxygen compounds	Organooxygen compounds	Carbonyl compounds	Carbonyl compounds	map00380 Tryptophan metabolism; map01100 Metabolic pathways; map05143 African trypanosomiasis;	other
2.27_147.0685	0.7099	0.9855	Indole-3-carbidol	C9 H9 N O	147.0685	2.27		S3581	HMDB0005785	0.0001	0.3434			6	level4	Organoheterocyclic compounds	Indoles and derivatives	Indoles	Indoles		other
2.421_194.069	1.3195	0.5713	4-aminohippuric acid	C9 H10 N2 O3	194.069	2.421	D01421	MReference-223	HMDB0001867	-0.0001	-0.5708		83.1	2	level2	Benzenoids	Benzene and substituted derivatives	Benzoic acids and derivatives	Benzene and derivatives		other
2.448_165.079	1.8376	0.2479	L-phenylalanine	C9 H11 N O2	165.079	2.448	C00079	MReference-8	HMDB0000159	0	0.0935	96.3	96.5	17	level1	Organic acids and derivatives	Carboxylic acids and derivatives	Amino acids, peptides, and analogues	Amino acids	map00360 Phenylalanine metabolism; map00400 Phenylalanine, tyrosine and tryptophan biosynthesis; map00970 Aminoacyl-tRNA biosynthesis; map01100 Metabolic pathways; map01210 2-Oxocarboxylic acid metabolism; map01230 Biosynthesis of amino acids; map02010 ABC transporters; map04974 Protein digestion and absorption; map04978 Mineral absorption; map05230 Central carbon metabolism [illegible]	other
2.45_174.1091	1.0083	0.9936	Dl-arginine	C6 H14 N4 O2	174.1091	2.45							57.5	0	level5						other
2.451_148.0525	1.1178	0.5661	Trans-cinnamic acid	C9 H8 O2	148.0525	2.451	C00423	MReference-1403	HMDB0000930	0.0001	0.6263		73.1	15	level2	Phenylpropanoids and polyketides	Cinnamic acids and derivatives	Cinnamic acids	Polyketides[PK]	map00130 Ubiquinone and other terpenoid-quinone biosynthesis; map00360 Phenylalanine metabolism; map01100 Metabolic pathways;	other
2.459_110.0367	1.6651	0.3327	Catechol	C6 H6 O2	110.0367	2.459	C00090	MReference-2991	HMDB0000957	-0.0001	-0.5186	93.2	94.2	7	level2	Benzenoids	Phenols	Benzenediols	Phenols and derivatives	map01100 Metabolic pathways;	other
2.459_189.9936	1.9538	0.2659	Mfcd12546417	C6 H6 O5 S	189.9936	2.459	C06674	S164229		0	-0.1063			4	level4						other
2.473_274.072	0.844	0.3942		C8 H18 O8 S	274.072	2.473								0	level5						other
2.51_188.1049	0.7999	0.1861	Azelaic acid	C9 H16 O4	188.1049	2.51	C08261	BGI162	HMDB0000784	0	0.1533	98.5	96.3	11	level2	FA Fatty acyls	FA01 Fatty Acids and Conjugates	FA0117 Dicarboxylic acids	Fatty acyls[FA]	NULL	other
2.568_252.1207	0.9366	0.5796		C10 H20 O7	252.1207	2.568								0	level5						other
2.583_274.072	1.5632	0.5429		C8 H18 O8 S	274.072	2.583								0	level5						other
2.63_182.0579	1.8818	0.2684	3,4-dihydroxyphenylpropionic acid	C9 H10 O4	182.0579	2.63	C10447	MReference-142	HMDB0000423	0	0.0645		82.6	32	level2	Phenylpropanoids and polyketides	Phenylpropanoic acids	null	Phenylpropanoic acids	map00350 Tyrosine metabolism;	other
2.654_234.0197	1.1576	0.5747	4-ethyl-2,6-dihydroxyphenyl hydrogen sulfate	C8 H10 O6 S	234.0197	2.654		S74852096	HMDB0128030	-0.0001	-0.4757			1	level4	Organic acids and derivatives	Organic sulfuric acids and derivatives	Arylsulfates	Organic acids		other
2.688_252.1207	0.7121	0.2042		C10 H20 O7	252.1207	2.688								0	level5						other
2.717_278.0668	0.531	0.2289	1-(2-deoxy-5-o-phosphono-beta-d-erythro-pentofuranosyl)-1,2-dihydropyrimidine	C9 H15 N2 O6 P	278.0668	2.717		S390164		0	0.1578			1	level4						other
2.721_242.0901	0.4376	0.1663	Thymidine	C10 H14 N2 O5	242.0901	2.721	C00214	MReference-1230	HMDB0000273	-0.0001	-0.5858		76.2	3	level1	Nucleosides, nucleotides, and analogues	Pyrimidine nucleosides	Pyrimidine 2'-deoxyribonucleosides	Nucleic acids and analogues	map00240 Pyrimidine metabolism; map01100 Metabolic pathways;	other
2.772_243.0201	2.7485	0.6616		C9 H9 N O5 S	243.0201	2.772								0	level5						other
2.802_166.063	3.7769	0.1664	3-(2-hydroxyphenyl)propanoate	C9 H10 O3	166.063	2.802	C01198	BGI78	HMDB0033752	0	-0.0758	81.5	75	44	level2	Phenylpropanoids and polyketides	Phenylpropanoic acids	null	Phenylpropanoic acids	map00360 Phenylalanine metabolism; map01100 Metabolic pathways;	other
2.836_159.0896	1.3532	0.3181	N-acetylvaline	C7 H13 N O3	159.0896	2.836		S198159	HMDB0011757	0	0.2784			17	level4	Organic acids and derivatives	Carboxylic acids and derivatives	Amino acids, peptides, and analogues	Amino acids, peptides, and analogues		other
2.866_369.9795	0.387	0.6071		C11 H17 O4 P3 S2	369.9795	2.866								0	level5						other
2.875_173.9988	1.0619	0.5177	4-phenolsulfonic acid	C6 H6 O4 S	173.9988	2.875	C12849	S4601		0.0001	0.7698			2	level4					NULL	other
2.88_357.052	0.7058	0.7016		C14 H15 N O8 S	357.052	2.88								0	level5						other

2.891_380.0985	1.0906	0.9887	L-gamma-glutamyl-s-[hydroxy(methyl)carbamoyl]-l-cysteinylglycine	C12 H20 N4 O8 S	380.0985	2.891	C04572	S112646		-0.0017	-4.3963			1	level4					NULL	other
2.893_135.0685	1.181	0.7955	Acetanilide	C8 H9 N O	135.0685	2.893	C07565	S880	HMDB0001250	0	0.2907			14	level4	Benzenoids	Benzene and substituted derivatives	null	Benzene and derivatives	NULL	other
2.894_179.0582	1.1205	0.9421	Hippurate	C9 H9 N O3	179.0582	2.894	C01586	BGI309	HMDB0000714	0	-0.1812	98	87.8	8	level1	Benzenoids	Benzene and substituted derivatives	Benzoic acids and derivatives	Benzene and derivatives	map00360 Phenylalanine metabolism; map01100 Metabolic pathways;	other
2.895_138.0317	0.742	0.4411	Salicylic acid	C7 H6 O3	138.0317	2.895	C00805	MReference-643	HMDB0001895	0	-0.0966		95.5	10	level2	Benzenoids	Benzene and substituted derivatives	Benzoic acids and derivatives	Benzene and derivatives	map00360 Phenylalanine metabolism; map01100 Metabolic pathways; map04976 Bile secretion;	other
2.905_130.0631	1.2416	0.4025	(hydroxyethyl)methacrylate	C6 H10 O3	130.0631	2.905	C14530	S12791		0.0001	0.4205			23	level4					NULL	other
2.907_184.0563	0.8283	0.2258	3-((2-methyl-3-furyl)thio)-2-butanone	C9 H12 O2 S	184.0563	2.907		S15528527	HMDB0032401	0.0005	2.9439			1	level4	Organosulfur compounds	Thioethers	Aryl thioethers	Aryl thioethers		other
2.908_139.0633	0.835	0.2886	Deferiprone	C7 H9 N O2	139.0633	2.908		S2866		0	0.0951			5	level4						other
2.912_183.0531	0.8342	0.2765	4-pyridoxic acid	C8 H9 N O4	183.0531	2.912	C00847	MReference-1	HMDB0000017	-0.0001	-0.4057		93.6	5	level2	Organoheterocyclic compounds	Pyridines and derivatives	Pyridinecarboxylic acids and derivatives	Pyridine and derivatives	map00750 Vitamin B6 metabolism; map01100 Metabolic pathways;	other
2.935_180.0606	0.8591	0.8467	[similar to: 5-methyldeoxycytidine; δ mass: -61.0456 da]		180.0606	2.935								0	level5						other
2.94_327.1319	1.7038	0.3806	(2s)-3-phenyl-2-({[(3s,4s,5r)-2,3,4-trihydroxy-5-(hydroxymethyl)tetrahydro-2-furanyl]methyl}amino)propanoic acid (non-preferred name)	C15 H21 N O7	327.1319	2.94		S35014483	HMDB0037846	0.0001	0.3299			1	level4	Organic acids and derivatives	Carboxylic acids and derivatives	Amino acids, peptides, and analogues	Amino acids, peptides, and analogues		other
2.944_420.1302	0.734	0.2776	9-bromo-16alpha-methyl-pregn-4-ene-3,11,20-trione	C22 H29 Br O3	420.1302	2.944	C14918	S10128379		0.0002	0.5651			1	level4					NULL	other
2.944_440.2244	0.6834	0.2264		C24 H34 N4 P2	440.2244	2.944								0	level5						other
2.945_237.1	1.604	0.3951	Ethopabate	C12 H15 N O4	237.1	2.945		S5812		-0.0001	-0.5005			6	level4						other
2.957_209.1211	0.8906	0.3747			209.1211	2.957								0	level5						other
2.958_180.0536	1.7946	0.2434	Nicotinuric acid	C8 H8 N2 O3	180.0536	2.958	C05380	MReference-520	HMDB0003269	0.0001	0.4724		60.4	3	level2	Organic acids and derivatives	Carboxylic acids and derivatives	Amino acids, peptides, and analogues	Amino acids, peptides, and analogues	map00760 Nicotinate and nicotinamide metabolism;	other
2.96_136.0637	1.9539	0.2591	N-methylnicotinamide	C7 H8 N2 O	136.0637	2.96		MReference-523	HMDB0003152	0.0001	0.3852		38.2	6	level3	Organoheterocyclic compounds	Pyridines and derivatives	Pyridinecarboxylic acids and derivatives	Pyridine and derivatives		other
2.962_218.0247	1.2434	0.471	4-(2-hydroxyethyl)phenyl hydrogen sulfate	C8 H10 O5 S	218.0247	2.962		S30777652	HMDB0041785	-0.0002	-0.8689			2	level4	Organic acids and derivatives	Organic sulfuric acids and derivatives	Arylsulfates	Organic acids		other
2.966_108.0688	1.9334	0.11	Phenylhydrazine	C6 H8 N2	108.0688	2.966	C02304	S7235		0	0.4615			9	level4					NULL	other
2.971_435.1931	0.932	0.6759		C21 H30 N3 O5 P	435.1931	2.971								0	level5						other
2.974_413.2112	0.9754	0.9636	Delanzomib	C21 H28 B N3 O5	413.2112	2.974		S23325665		-0.001	-2.3856			1	level4						other
2.979_166.063	1.37	0.3548	3-phenyllactic acid	C9 H10 O3	166.063	2.979	C05607	MReference-311	HMDB0000748	0	-0.0228	59	94.9	44	level2	Phenylpropanoids and polyketides	Phenylpropanoic acids	null	Phenylpropanoic acids	map00360 Phenylalanine metabolism; map01100 Metabolic pathways;	other
2.993_117.0579	1.2355	0.4696	Indole	C8 H7 N	117.0579	2.993	C00463	S776	HMDB0000738	0.0001	0.513			2	level4	Organoheterocyclic compounds	Indoles and derivatives	Indoles	Indoles	map00380 Tryptophan metabolism; map00400 Phenylalanine, tyrosine and tryptophan biosynthesis; map01100 Metabolic pathways; map04974 Protein digestion and absorption;	other

2.993_204.09	1.243	0.4513	L-tryptophan	C11 H12 N2 O2	204.09	2.993	C00078	BGI382	HMDB0000929	0.0001	0.487	98.5	93.6	12	level1	Peptides	Amino acids	Common amino acids [Fig]	Amino acids	map00260 Glycine, serine and threonine metabolism; map00380 Tryptophan metabolism; map00400 Phenylalanine, tyrosine and tryptophan biosynthesis; map00970 Aminoacyl-tRNA biosynthesis; map01100 Metabolic pathways; map01210 2-Oxocarboxylic acid metabolism; map01230 Biosynthesis of amino acids; map04726 Serotonergic synapse; map04974 Protein digestion and absorption; map04978 Mineral [illegible]	other
2.998_430.1618	1.3739	0.3605	Sesartemin	C23 H26 O8	430.1618	2.998	C10884	S303816	HMDB0038931	-0.001	-2.3501			5	level4	Phenylpropanoids	Lignans	Lignans	Lignans	NULL	other
2.999_404.2048	1.2414	0.3604	Flunarizine	C26 H26 F2 N2	404.2048	2.999		S819216	HMDB0015589	-0.0016	-4.0048			1	level4	Benzenoids	Benzene and substituted derivatives	Diphenylmethanes	Benzene and derivatives		other
3.005_318.0984	1.1053	0.932		C11 H18 N4 O5 S	318.0984	3.005								0	level5						other
3.006_275.0462	0.7837	0.7628	Methyl o-sulfo-l-tyrosinate	C10 H13 N O6 S	275.0462	3.006	C04201	S389236		-0.0001	-0.5113			1	level4					NULL	other
3.01_184.0736	0.825	0.5982	Mhpg	C9 H12 O4	184.0736	3.01	C05594	S10348	HMDB0001490	0	0.0862			17	level4	Benzenoids	Phenols	Methoxyphenols	Phenols and derivatives	map00350 Tyrosine metabolism; map01100 Metabolic pathways;	other
3.012_213.0095	0.2964	0.1452	3-indoxyl sulphate	C8 H7 N O4 S	213.0095	3.012		MReference-1303	HMDB0000682	-0.0001	-0.477		94.8	1	level2	Organic acids and derivatives	Organic sulfuric acids and derivatives	Arylsulfates	Organic acids		other
3.013_315.0989	1.3187	0.5052	Flusilazole	C16 H15 F2 N3 Si	315.0989	3.013	C18733	S66326	HMDB0039815	-0.0014	-4.473			1	level4	Benzenoids	Benzene and substituted derivatives	Halobenzenes	Benzene and derivatives	NULL	other
3.022_448.0011	0.0542	0.1604		C15 H19 N2 O4 P3 S2	448.0011	3.022								0	level5						other
3.066_189.0426	1.6615	0.3565	Kynurenic acid	C10 H7 N O3	189.0426	3.066	C01717	MReference-458	HMDB0000715	0	-0.0166		89.7	6	level2	Organoheterocyclic compounds	Quinolines and derivatives	Quinoline carboxylic acids	Quinoline carboxylic acids	map00380 Tryptophan metabolism; map01100 Metabolic pathways;	other
3.076_400.0672	1.8117	0.34		C17 H12 N4 O8	400.0672	3.076								0	level5						other
3.101_202.0855	2.3473	0.2535	Metamiton	C10 H10 N4 O	202.0855	3.101	C10930	S35563		0.0001	0.3863			2	level4					NULL	other
3.132_362.072	0.7139	0.305		C12 H18 N4 O5 S2	362.072	3.132								0	level5						other
3.136_296.1471	1.1001	0.3019		C12 H24 O8	296.1471	3.136								0	level5						other
3.166_205.0739	1.1272	0.7209	Indole-3-lactic acid	C11 H11 N O3	205.0739	3.166	C02043	MReference-450	HMDB0000671	0	-0.076		89.7	8	level2	Organoheterocyclic compounds	Indoles and derivatives	Indolyl carboxylic acids and derivatives	Indole and derivatives		other
3.17_254.1265	1.4836	0.2847	Midodrine	C12 H18 N2 O4	254.1265	3.17	C07890	S4050	HMDB0014356	-0.0002	-0.5946			5	level4	Benzenoids	Benzene and substituted derivatives	Methoxybenzenes	Benzene and derivatives	NULL	other
3.187_231.02	1.3737	0.3811	Paracetamol sulfate	C8 H9 N O5 S	231.02	3.187		S75741	HMDB0059911	-0.0001	-0.4445			2	level4	Organic acids and derivatives	Organic sulfuric acids and derivatives	Arylsulfates	Organic acids		other
3.199_366.1429	2.0143	0.2507		C18 H18 N6 O3	366.1429	3.199								0	level5						other
3.209_232.0041	1.6845	0.2822	4-formyl-2-methoxyphenyl hydrogen sulfate	C8 H8 O6 S	232.0041	3.209		S10798458	HMDB0041789	-0.0001	-0.4854			5	level4	Organic acids and derivatives	Organic sulfuric acids and derivatives	Arylsulfates	Organic acids		other
3.241_232.9308	2.4124	0.0602		C3 H7 N O3 S4	232.9308	3.241								0	level5						other
3.245_464.1563	1.254	0.6583		C20 H34 O6 P2 S	464.1563	3.245								0	level5						other
3.287_260.1621	21.4495	0.0553		C13 H24 O5	260.1621	3.287								0	level5						other
3.294_188.0699	2.1861	0.265		C9 H8 N4 O	188.0699	3.294								0	level5						other
3.294_209.0147	1.713	0.2729		C9 H7 N O3 S	209.0147	3.294								0	level5						other
3.305_220.0041	0.5604	0.4166		C7 H8 O6 S	220.0041	3.305								0	level5						other
3.33_166.03	0.711	0.3566	3-methyl-2-buten-1-yl hydrogen sulfate	C5 H10 O4 S	166.03	3.33		S74854048	HMDB0136691	0	0.0338			1	level4	Organic acids and derivatives	Organic sulfuric acids and derivatives	Sulfuric acid esters	Organic acids		other
3.34_193.0739	0.4939	0.3436	Phenylacetylglycine	C10 H11 N O3	193.0739	3.34	C05598	MReference-540	HMDB0000821	0	0.183		96.2	15	level2	Organic acids and derivatives	Carboxylic acids and derivatives	Amino acids, peptides, and analogues	Amino acids, peptides, and analogues	map00360 Phenylalanine metabolism;	other
3.341_75.0321	0.4754	0.3261	Glycine	C2 H5 N O2	75.0321	3.341	C00037	MReference-2524	HMDB0000123	0	0.3797		92.3	5	level2	Organic acids and derivatives	Carboxylic acids and derivatives	Amino acids, peptides, and analogues	Amino acids	map00120 Primary bile acid biosynthesis; map00230 Purine metabolism; map00260 Glycine, serine and threonine metabolism; map00310 Lysine degradation; map00440 Phosphonate and phosphinate metabolism; map00480 Glutathione metabolism; map00630 Glyoxylate and dicarboxylate metabolism; map00730 Thiamine metabolism; map00860 Porphyrin and chlorophyll metabolism; map00970 [illegible]	other
3.347_252.0665	1.154	0.326		C9 H16 O6 S	252.0665	3.347								0	level5						other
3.35_195.053	0.6701	0.9972	2-((4-nitrophenoxy)methyl)oxirane	C9 H9 N O4	195.053	3.35	C04274	S20045		-0.0001	-0.6231			14	level4					NULL	other

3.365_216.0093	1.3674	0.4095	2-hydroxy-5-vinylphenyl hydrogen sulfate	C8 H8 O5 S	216.0093	3.365		S74851573	HMDB0124978	0	0.1466			1	level4	Organic acids and derivatives	Organic sulfuric acids and derivatives	Arylsulfates	Organic acids		other
3.388_394.0442	0.4214	0.2625	7-chloro-2-(3,4-dimethoxyphenyl)-3,5,6-trihydroxy-8-methoxy-4h-chromen-4-one	C18 H15 Cl O8	394.0442	3.388		S74854007	HMDB0135845	-0.0014	-3.4833			2	level4	Phenylpropanoids and polyketides	Flavonoids	Flavones	Flavonoids		other
3.395_173.106	1.8416	0.2696	N-acetyl-d-alloisoleucine	C8 H15 N O3	173.106	3.395		MReference-1480		0.0008	4.3629		78.5	8	level2						other
3.418_302.079	0.0551	0.2108	Hematoxylin	C16 H14 O6	302.079	3.418	C09931	MReference-1818		0	0.0523		51	52	level3	PK Polyketides	PK12 Flavonoids	PK1210 Neoflavonoids	Polyketides[PK]	NULL	other
3.425_278.0724	1.634	0.3819		C13 H14 N2 O3 S	278.0724	3.425								0	level5						other
3.425_362.1248	0.8632	0.4847		C12 H26 O10 S	362.1248	3.425								0	level5						other
3.426_200.095	1.6695	0.3798	4,4'-oxydianiline	C12 H12 N2 O	200.095	3.426	C14759	S7298		0.0001	0.3347			2	level4					NULL	other
3.433_284.0895	0.492	0.3385	Mfcd08277028	C13 H16 O7	284.0895	3.433		S135751	HMDB0011686	-0.0001	-0.4513			2	level4	Organic oxygen compounds	Organooxygen compounds	Carbohydrates and carbohydrate conjugates	Carbohydrates		other
3.444_191.0583	1.0694	0.5824	5-hydroxyindole-3-acetic acid	C10 H9 N O3	191.0583	3.444	C05635	MReference-256	HMDB0000763	0	0.2208		68.9	4	level2	Organoheterocyclic compounds	Indoles and derivatives	Indolyl carboxylic acids and derivatives	Indole and derivatives	map00380 Tryptophan metabolism; map01100 Metabolic pathways; map04726 Serotonergic synapse;	other
3.446_145.0528	1.0712	0.5862	Fg7175000	C9 H7 N O	145.0528	3.446	C06338	S5816		0	0.1212		65.9	6	level4					NULL	other
3.449_250.0951	1.2388	0.3847		C12 H14 N2 O4	250.0951	3.449								0	level5						other
3.466_161.0477	0.6027	0.1822	Indole-2-carboxylic acid	C9 H7 N O2	161.0477	3.466		MReference-1302	HMDB0002285	0	0.1117		68.2	8	level2	Organoheterocyclic compounds	Indoles and derivatives	Indolecarboxylic acids and derivatives	Indole and derivatives		other
3.479_154.0266	0.3758	0.0009	Gentisic acid	C7 H6 O4	154.0266	3.479	C00628	MReference-110	HMDB0000152	0	0.0722	78.9	81.9	8	level2	Benzenoids	Benzene and substituted derivatives	Benzoic acids and derivatives	Benzene and derivatives	map00350 Tyrosine metabolism; map01100 Metabolic pathways;	down
3.482_258.1466	68.0249	0.0001		C13 H22 O5	258.1466	3.482								0	level5						up
3.482_340.1734	1.1999	0.3636		C14 H28 O9	340.1734	3.482								0	level5						other
3.505_248.0352	0.7135	0.2713	4-ethyl-2-hydroxy-6-methoxyphenyl hydrogen sulfate	C9 H12 O6 S	248.0352	3.505		S74852093	HMDB0128019	-0.0002	-0.839			1	level4	Organic acids and derivatives	Organic sulfuric acids and derivatives	Arylsulfates	Organic acids		other
3.507_508.1823	0.8221	0.3178		C22 H38 O7 P2 S	508.1823	3.507								0	level5						other
3.57_193.074	1.5414	0.4567	2-methylhippuric acid	C10 H11 N O3	193.074	3.57		MReference-165	HMDB0011723	0.0001	0.427		88.8	15	level2	Benzenoids	Benzene and substituted derivatives	Benzoic acids and derivatives	Benzene and derivatives		other
3.589_264.1206	2.2217	0.0888		C11 H20 O7	264.1206	3.589								0	level5						other
3.627_246.0196	0.7855	0.9605	3-(sulfooxy)benzenepropanoic acid	C9 H10 O6 S	246.0196	3.627		S162993	HMDB0094710	-0.0002	-0.944			10	level4	Organic acids and derivatives	Organic sulfuric acids and derivatives	Arylsulfates	Organic acids		other
3.643_232.0847	1.0745	0.8409	Phenobarbital	C12 H12 N2 O3	232.0847	3.643	C07434	S4599	HMDB0015305	-0.0001	-0.4904			3	level4	Organoheterocyclic compounds	Diazines	Pyrimidines and pyrimidine derivatives	Pyrimidines and pyrimidine derivatives	NULL	other
3.666_286.1205	0.4058	0.2184	Diphenolic acid	C17 H18 O4	286.1205	3.666	C14294	S60518		0	-0.0491			17	level4					NULL	other
3.667_330.1105	0.4465	0.271	Acetylshikonin	C18 H18 O6	330.1105	3.667	C17413	S30089		0.0002	0.4707			27	level4					NULL	other
3.677_319.0575	2.1406	0.1267		C8 H17 N O10 S	319.0575	3.677								0	level5						other
3.68_287.08	0.928	0.4633	1,3,5-trihydroxy-4-methoxy-10-methyl-9(10h)-acridinone	C15 H13 N O5	287.08	3.68		S8192468	HMDB0030373	0.0006	2.1863		34.9	2	level4	Organoheterocyclic compounds	Quinolines and derivatives	Benzoquinolines	Quinone		other
3.701_242.1154	3.9776	0.1376		C12 H18 O5	242.1154	3.701								0	level5						other
3.703_204.0093	0.7702	0.5387	Guaiacol sulfate	C7 H8 O5 S	204.0093	3.703		S21078	HMDB0060013	0.0001	0.3682			1	level4	Organic acids and derivatives	Organic sulfuric acids and derivatives	Arylsulfates	Organic acids		other
3.713_241.0044	1.7459	0.7201	Indole-3-carboxilic acid-o-sulphate	C9 H7 N O5 S	241.0044	3.713		S30778505	HMDB0060002	-0.0001	-0.4116			1	level4	Organoheterocyclic compounds	Indoles and derivatives	Indolecarboxylic acids and derivatives	Indole and derivatives		other
3.724_207.0895	2.1342	0.2416	N-acetyl-l-phenylalanine	C11 H13 N O3	207.0895	3.724	C03519	BGI422	HMDB0000512	-0.0001	-0.3249	98.4	88.8	10	level2	Organic acids and derivatives	Carboxylic acids and derivatives	Amino acids, peptides, and analogues	Amino acids, peptides, and analogues	map00360 Phenylalanine metabolism; map01100 Metabolic pathways;	other
3.728_406.151	0.958	0.6186		C15 H26 N4 O7 S	406.151	3.728								0	level5						other
3.76_156.0787	1.7508	0.2227	2,5-dimethyl-4-ethoxy-3(2h)-furanone	C8 H12 O3	156.0787	3.76		S2285292	HMDB0032232	0.0001	0.4672			3	level4	Organoheterocyclic compounds	Dihydrofurans	Furanones	Furanones		other
3.769_228.9901	1.1519	0.8637		C5 H11 N O3 S3	228.9901	3.769								0	level5						other
3.784_384.1998	1.1468	0.2332		C16 H32 O10	384.1998	3.784								0	level5						other
3.794_240.1361	16.1512	0.0785		C13 H20 O4	240.1361	3.794								0	level5						other
3.795_257.9833	1.5324	0.2396		C9 H6 O7 S	257.9833	3.795								0	level5						other
3.804_348.0912	2.0377	0.1573		C11 H24 O8 S2	348.0912	3.804								0	level5						other
3.806_246.1003	2.3395	0.2215	N-acetyl-dl-tryptophan	C13 H14 N2 O3	246.1003	3.806		MReference-814		-0.0002	-0.7553	77.8	84.6	6	level2						other
3.821_244.1309	0.5082	0.8853	6-hydroxy-5-methyl-4,11-dioxoundecanoic acid	C12 H20 O5	244.1309	3.821		S21258154	HMDB0032472	-0.0002	-0.7762			1	level4	Organic acids and derivatives	Keto acids and derivatives	Medium-chain keto acids and derivatives	Organic acids		other
3.86_246.1465	1.983	0.1347	Dibutyl malate	C12 H22 O5	246.1465	3.86		S86081	HMDB0031696	-0.0002	-0.8232			2	level4	Organic acids and derivatives	Hydroxy acids and derivatives	Beta hydroxy acids and derivatives	Organic acids		other
3.879_243.0201	1.4237	0.7619		C9 H9 N O5 S	243.0201	3.879								0	level5						other

3.922_122.0368	1.9018	0.038	Benzoic acid	C7 H6 O2	122.0368	3.922	C00180	S238	HMDB0001870	0	-0.1063			6	level4	Benzenoids	Benzene and substituted derivatives	Benzoic acids and derivatives	Benzene and derivatives	map00360 Phenylalanine metabolism; map01100 Metabolic pathways;	up
3.949_596.2354	1.0694	0.82		C22 H44 O16 S	596.2354	3.949								0	level5						other
3.949_808.3638	1.8678	0.1956		C38 H57 N4 O13 P	808.3638	3.949								0	level5						other
3.967_231.053	1.015	0.571	Eglinazine	C7 H10 Cl N5 O2	231.053	3.967	C19102	S98703		0.0007	3.1834			1	level4					NULL	other
3.983_130.0994	2.1251	0.2279	Heptanoic acid	C7 H14 O2	130.0994	3.983	C17714	MReference-2836	HMDB0000666	0	0.3359		68.8	29	level2	Lipids and lipid-like molecules	Fatty Acyls	Fatty acids and conjugates	Fatty acyls[FA]	NULL	other
3.987_450.1771	0.9407	0.5734		C16 H34 O12 S	450.1771	3.987								0	level5						other
3.993_176.1048	2.1862	0.2212	Ethanal tetramer	C8 H16 O4	176.1048	3.993	C18744	S54981		0	-0.03			4	level4					NULL	other
4.005_152.0586	3.9173	0.1823	5-nitro-o-toluidine	C7 H8 N2 O2	152.0586	4.005	C16398	S7166		0	0.1957			5	level4						other
4.015_177.079	1.8754	0.3648	5-hydroxytryptophol	C10 H11 N O2	177.079	4.015		S8708	HMDB0001855	0	-0.0408			6	level4	Organoheterocyclic compounds	Indoles and derivatives	Hydroxyindoles	Indole and derivatives		other
4.039_428.2259	1.2185	0.0737		C18 H36 O11	428.2259	4.039								0	level5						other
4.046_146.0943	1.2849	0.44	N-butyl lactate	C7 H14 O3	146.0943	4.046		S8409	HMDB0040254	0	0.2411			13	level4	Organic acids and derivatives	Carboxylic acids and derivatives	Carboxylic acid derivatives	Organic acids		other
4.053_276.0666	1.2022	0.4592	4-(3-hydroxybutyl)-2-methoxyphenyl hydrogen sulfate	C11 H16 O6 S	276.0666	4.053		S74853929	HMDB0135717	-0.0002	-0.5621			2	level4	Organic acids and derivatives	Organic sulfuric acids and derivatives	Arylsulfates	Organic acids		other
4.053_518.1241	0.0546	0.2451	[5,7,7'-trihydroxy-2'-(4-methyl-3-penten-1-yl)-4-oxo-3,4-dihydro-2h,2'h-2,6'-bichromen-2'-yl]methyl hydrogen sulfate	C25 H26 O10 S	518.1241	4.053		S74853005	HMDB0132814	-0.0006	-1.0993			3	level4	Phenylpropanoids and polyketides	Flavonoids	Pyranoflavonoids	Flavonoids		other
4.065_398.0108	0.2515	0.2009		C13 H21 O4 P3 S2	398.0108	4.065								0	level5						other
4.072_108.0572	0.3013	0.2314	4-methylphenol	C7 H8 O	108.0572	4.072	C01468	MReference-3199	HMDB0001858	-0.0003	-3.1925		89	5	level2	Benzenoids	Phenols	Cresols	Phenols and derivatives	map01100 Metabolic pathways; map04974 Protein digestion and absorption;	other
4.075_188.0143	0.286	0.2622	P-cresylsulfate	C7 H8 O4 S	188.0143	4.075		S3806481	HMDB0011635	0	-0.0172			3	level4	Organic acids and derivatives	Organic sulfuric acids and derivatives	Arylsulfates	Organic acids		other
4.107_390.1528	1.1964	0.3555	Loganin	C17 H26 O10	390.1528	4.107	C01433	S79111		0.0002	0.5592			1	level4	Terpenoids	Monoterpenoids (C10)	Iridoids	Terpenoids	map01100 Metabolic pathways;	other
4.108_242.1154	0.6494	0.6956		C12 H18 O5	242.1154	4.108								0	level5						other
4.113_392.1175	3.7296	0.1273		C9 H26 N6 O5 P2 S	392.1175	4.113								0	level5						other
4.116_363.0838	4.4417	0.1235		C11 H17 N5 O7 S	363.0838	4.116								0	level5						other
4.124_196.0848	1.1249	0.3955	5-nitro-2-propoxyaniline	C9 H12 N2 O3	196.0848	4.124		S10647	HMDB0037688	0	0.1813			2	level4	Benzenoids	Benzene and substituted derivatives	Nitrobenzenes	Benzene and derivatives		other
4.125_152.095	1.3653	0.3714	Isopropyl methoxy pyrazine	C8 H12 N2 O	152.095	4.125		S30649	HMDB0040340	0	0.308			5	level4	Organoheterocyclic compounds	Diazines	Pyrazines	Pyrazines		other
4.13_254.0248	4.635	0.183		C11 H10 O5 S	254.0248	4.13								0	level5						other
4.141_640.2632	2.2319	0.1372		C23 H49 N2 O14 P S	640.2632	4.141								0	level5						other
4.217_584.1901	3.1301	0.7781		C31 H28 N4 O8	584.1901	4.217								0	level5						other
4.224_494.2031	1.0147	0.7831		C16 H41 N4 O5 P3 S	494.2031	4.224								0	level5						other
4.229_169.9967	0.8227	0.4555	Heptafluoro propane	C3 H F7	169.9967	4.229		S61257		0	0.1571			1	level4						other
4.229_213.9865	0.82	0.3641	Benzothiazole-2-sulfonamide	C7 H6 N2 O2 S2	213.9865	4.229	C14181	S61261		-0.0005	-2.5355			1	level4					NULL	other
4.264_304.0981	1.4705	0.2582		C13 H20 O6 S	304.0981	4.264								0	level5						other
4.27_472.2519	1.0539	0.6114	Sulukast	C25 H36 N4 O3 S	472.2519	4.27		S4940793		0.0011	2.3649			1	level4						other
4.328_266.1265	1.5727	0.2563	Threonylphenylalanine	C13 H18 N2 O4	266.1265	4.328		S3313819	HMDB0029068	-0.0002	-0.7379			5	level4	Organic acids and derivatives	Carboxylic acids and derivatives	Amino acids, peptides, and analogues	Amino acids, peptides, and analogues		other
4.332_964.4668	3.665	0.1933		C45 H68 N6 O17	964.4668	4.332								0	level5						other
4.359_436.1437	2.3147	0.1086		C15 H32 O10 S2	436.1437	4.359								0	level5						other
4.394_270.1465	0.6912	0.6144		C14 H22 O5	270.1465	4.394								0	level5						other
4.408_245.0686	2.455	0.1592	Haplopine	C13 H11 N O4	245.0686	4.408	C10694	S10254899		-0.0002	-0.9918			2	level4	Alkaloids	Alkaloids derived from tryptophan and anthranilic acid	Quinoline alkaloids	Alkaloids	NULL	other
4.425_538.2289	0.9741	0.6734		C21 H38 N4 O10 S	538.2289	4.425								0	level5						other
4.431_292.098	1.1048	0.3871	6-chloro-12-methyl-2-oxa-12,15-diazatetracyclo[7.5.3.0~1,10~.0~3,8~]heptadeca-3,5,7-trien-16-one	C15 H17 Cl N2 O2	292.098	4.431		S2315437		0.0002	0.5329			1	level4						other
4.451_407.11	2.4382	0.0695		C16 H27 N O5 P2 S	407.11	4.451								0	level5						other
4.476_516.2778	1.0535	0.6368		C22 H44 O13	516.2778	4.476								0	level5						other
4.494_206.1154	2.1651	0.2068		C9 H18 O5	206.1154	4.494								0	level5						other
4.496_316.1311	0.9957	0.7168	Combretastatin a-4	C18 H20 O5	316.1311	4.496	C20268	S4508364		0.0001	0.1847			17	level4					NULL	other
4.52_394.0724	0.9838	0.7493	Diflufenican	C19 H11 F5 N2 O2	394.0724	4.52	C18549	S82834		-0.0016	-4.1453			1	level4					NULL	other
4.555_277.1061	2.4436	0.189		C13 H15 N3 O4	277.1061	4.555								0	level5						other
4.56_214.1205	0.9989	0.4259	5-hexyl-2-oxotetrahydro-3-furancarboxylic acid	C11 H18 O4	214.1205	4.56		S8551389	HMDB0030984	0	0.086			3	level4	Organoheterocyclic compounds	Lactones	Gamma butyrolactones	Gamma butyrolactones		other
4.583_480.1696	1.5739	0.4977		C20 H29 N6 O4 P S	480.1696	4.583								0	level5						other

4.607_418.1266	1.488	0.3308	Aloin a	C21 H22 O9	418.1266	4.607	C10305	S24534069		0.0003	0.6001			15	level4	Polyketides	Anthraquinones	Anthrone type	Quinone	NULL	other
4.612_582.2563	0.9421	0.5463		C21 H47 N2 O12 P S	582.2563	4.612								0	level5						other
4.66_560.3052	1.0383	0.681		C24 H48 O14	560.3052	4.66								0	level5						other
4.661_604.3072	0.5554	0.0056		C27 H51 N4 O5 P3	604.3072	4.661								0	level5						down
4.694_200.0144	1.2045	0.5956	4-vinylphenol sulfate	C8 H8 O4 S	200.0144	4.694		S4932200	HMDB0062775	0	0.1121			2	level4	Organic acids and derivatives	Organic sulfuric acids and derivatives	Arylsulfates	Organic acids		other
4.695_260.9619	1.2583	0.9963		C5 H11 N O3 S4	260.9619	4.695								0	level5						other
4.728_451.136	2.5249	0.0368		C17 H32 N3 O3 P3 S	451.136	4.728								0	level5						up
4.751_332.0534	1.8897	0.2573	Patuletin	C16 H12 O8	332.0534	4.751	C10118	S4444997	HMDB0030802	0.0002	0.5601			27	level4	Phenylpropanoids and polyketides	Flavonoids	Flavones	Flavonoids	NULL	other
4.772_524.1956	1.525	0.4543		C17 H43 N4 O4 P3 S2	524.1956	4.772								0	level5						other
4.782_626.2838	0.9093	0.4887		C23 H51 N2 O13 P S	626.2838	4.782								0	level5						other
4.827_139.027	0.8578	0.2616	6-hydroxynicotinic acid	C6 H5 N O3	139.027	4.827	C01020	MReference-172	HMDB0002658	0	0.3449		71	6	level2	Organoheterocyclic compounds	Pyridines and derivatives	Pyridinecarboxylic acids and derivatives	Pyridine and derivatives	map00760 Nicotinate and nicotinamide metabolism; map01100 Metabolic pathways;	other
4.829_604.331	1.0517	0.6311		C26 H52 O15	604.331	4.829								0	level5						other
4.83_324.0669	0.6353	0.2587	3-[2-(3-hydroxy-5-methoxyphenyl)ethyl]phenyl hydrogen sulfate	C15 H16 O6 S	324.0669	4.83		S74852692	HMDB0129925	0.0001	0.3971			2	level4	Phenylpropanoids and polyketides	Stilbenes	null	Polyketides[PK]		other
4.86_190.1206	2.1472	0.2279		C9 H18 O4	190.1206	4.86								0	level5						other
4.896_364.0255	1.8835	0.2462	5-(7-hydroxy-4-oxo-4h-chromen-2-yl)-2-methoxyphenyl hydrogen sulfate	C16 H12 O8 S	364.0255	4.896		S74853269	HMDB0133321	0.0002	0.5631			1	level4	Phenylpropanoids and polyketides	Flavonoids	O-methylated flavonoids	Flavonoids		other
4.907_402.1923	1.7312	0.0058		C19 H27 N6 O2 P	402.1923	4.907								0	level5						up
4.919_404.1685	0.6963	0.3379		C18 H28 O10	404.1685	4.919								0	level5						other
4.932_670.3094	0.9302	0.5474		C26 H54 O17 S	670.3094	4.932								0	level5						other
4.944_568.2229	1.3964	0.422	Formocortal	C29 H38 Cl F O8	568.2229	4.944		S254985		-0.001	-1.8138			1	level4						other
4.958_495.162	2.3477	0.0525		C16 H35 N O12 P2	495.162	4.958								0	level5						other
4.975_388.2099	0.999	0.6075	Ilomastat	C20 H28 N4 O4	388.2099	4.975		S117009		-0.0011	-2.9414			6	level4						other
4.981_648.3585	1.0343	0.731		C28 H56 O16	648.3585	4.981								0	level5						other
5.076_714.3372	0.9076	0.5693		C28 H58 O18 S	714.3372	5.076								0	level5						other
5.083_474.1526	0.6138	0.2453		C24 H26 O10	474.1526	5.083								0	level5						other
5.102_426.042	1.4938	0.5045		C14 H14 N6 O6 S2	426.042	5.102								0	level5						other
5.104_612.2503	1.3561	0.9708		C23 H48 O14 S2	612.2503	5.104								0	level5						other
5.106_202.03	1.6123	0.4869	4-ethylphenylsulfonic acid	C8 H10 O4 S	202.03	5.106		S34448630	HMDB0062551	0	0.0954			2	level4	Organic acids and derivatives	Organic sulfuric acids and derivatives	Arylsulfates	Organic acids		other
5.109_548.3037	1.1961	0.193	Belfosdil	C27 H50 O7 P2	548.3037	5.109		S65099		0.0005	0.9722			2	level4						other
5.112_538.275	0.697	0.8603		C23 H43 Cl N4 O8	538.275	5.112								0	level5						other
5.116_446.2184	1.6843	0.0108		C21 H41 N2 P3 S	446.2184	5.116								0	level5						up
5.118_278.163	1.6695	0.3265	Ro 20-1724	C15 H22 N2 O3	278.163	5.118		S4908	HMDB0062483	-0.0001	-0.3439			4	level4	Benzenoids	Phenol ethers	Anisoles	Benzene and derivatives		other
5.121_692.3861	1.0032	0.9211		C29 H61 N2 O14 P	692.3861	5.121								0	level5						other
5.127_230.0247	0.8278	0.7869	4-vinylguaiacol sulfate	C9 H10 O5 S	230.0247	5.127		S58170415	HMDB0127980	-0.0002	-0.6808			14	level4	Organic acids and derivatives	Organic sulfuric acids and derivatives	Arylsulfates	Organic acids		other
5.136_474.1526	0.6979	0.4494		C24 H26 O10	474.1526	5.136								0	level5						other
5.203_232.131	0.6237	0.2937		C11 H20 O5	232.131	5.203								0	level5						other
5.203_253.0407	3.2148	0.267	Actinoquinol	C11 H11 N O4 S	253.0407	5.203		S22136		-0.0001	-0.571			2	level4						other
5.204_338.0461	0.4991	0.4482	2-hydroxy-5-[3-(2-hydroxyphenyl)propanoyl]phenyl hydrogen sulfate	C15 H14 O7 S	338.0461	5.204		S74853828	HMDB0135421	0.0001	0.2787			15	level4	Phenylpropanoids and polyketides	Linear 1,3-diarylpropanoids	Chalcones and dihydrochalcones	Polyketides[PK]		other
5.205_758.3648	0.9156	0.5838		C29 H63 N2 O16 P S	758.3648	5.205								0	level5						other
5.217_322.0512	0.9636	0.488	2-hydroxy-5-(3-phenylpropanoyl)phenyl hydrogen sulfate	C15 H14 O6 S	322.0512	5.217		S74853827	HMDB0135416	0.0001	0.3862		31	9	level4	Phenylpropanoids and polyketides	Linear 1,3-diarylpropanoids	Chalcones and dihydrochalcones	Polyketides[PK]		other
5.251_656.2761	1.565	0.4317		C28 H45 N6 O8 P S	656.2761	5.251								0	level5						other
5.264_211.1209	1.638	0.2432	Isoprenaline	C11 H17 N O3	211.1209	5.264	C07056	S3647	HMDB0015197	0	0.1658			5	level4	Benzenoids	Phenols	Benzenediols	Phenols and derivatives	map04261 Adrenergic signaling in cardiomyocytes;	other
5.265_200.0507	7.9055	0.7632		C9 H12 O3 S	200.0507	5.265								0	level5						other
5.266_582.3025	1.1191	0.4937		C25 H47 Cl N4 O9	582.3025	5.266								0	level5						other
5.267_592.3311	1.2212	0.131		C29 H54 O8 P2	592.3311	5.267								0	level5						other
5.287_368.0568	0.4852	0.5972	3,5-dihydroxy-2-(4-hydroxy-3-methoxyphenyl)-3,4-dihydro-2h-chromen-7-yl hydrogen sulfite	C16 H16 O8 S	368.0568	5.287		S74849512	HMDB0029177	0.0002	0.6771			13	level4	Phenylpropanoids and polyketides	Flavonoids	Flavans	Flavonoids		other
5.293_490.2444	1.7804	0.0022		C20 H44 O9 P2	490.2444	5.293								0	level5						up
5.322_802.3903	0.8905	0.5095		C31 H67 N2 O17 P S	802.3903	5.322								0	level5						other
5.326_276.103	1.925	0.2903		C12 H20 O5 S	276.103	5.326								0	level5						other

5.33_170.0943	1.0362	0.8124	2-(diethoxymethyl)furan	C9 H14 O3	170.0943	5.33	C14280	S75385		0	0.1037			3	level4					NULL	other
5.34_583.2153	2.3486	0.0508		C20 H41 N O16 S	583.2153	5.34								0	level5						other
5.359_434.1215	0.8302	0.4312	Prunin	C21 H22 O10	434.1215	5.359	C09099	S83766		0.0002	0.4071			9	level4	Flavonoids	Flavonoids	Flavanones	Flavonoids		other
5.371_700.3036	1.6202	0.4307		C26 H57 N2 O13 P S2	700.3036	5.371								0	level5						other
5.374_186.0892	2.2751	0.2306	Cis-2-carboxycyclohexyl-acetic acid	C9 H14 O4	186.0892	5.374	C14112	S571146		0	0.0304			3	level4						other
5.406_626.3302	1.218	0.152		C27 H51 Cl N4 O10	626.3302	5.406								0	level5						other
5.41_636.3588	1.2105	0.094		C27 H56 O16	636.3588	5.41								0	level5						other
5.43_378.0775	0.6149	0.091		C18 H18 O7 S	378.0775	5.43								0	level5						other
5.431_846.418	1.0324	0.8447		C33 H71 N2 O18 P S	846.418	5.431								0	level5						other
5.432_160.11	0.7908	0.1043	8-hydroxyoctanoic acid	C8 H16 O3	160.11	5.432		S63018	HMDB0061914	0	0.2452			23	level4	Organic acids and derivatives	Hydroxy acids and derivatives	Medium-chain hydroxy acids and derivatives	Organic acids		other
5.454_534.2701	1.662	0.0067		C23 H42 N4 O8 S	534.2701	5.454								0	level5						up
5.458_144.115	2.024	0.2395	Caprylic acid	C8 H16 O2	144.115	5.458	C06423	MReference-2704	HMDB0000482	0	-0.0888		81.5	39	level2	Lipids and lipid-like molecules	Fatty Acyls	Fatty acids and conjugates	Fatty acyls[FA]	map00061 Fatty acid biosynthesis; map00785 Lipoic acid metabolism; map01100 Metabolic pathways;	other
5.496_627.2428	2.2267	0.0504		C21 H46 N3 O14 P S	627.2428	5.496								0	level5						other
5.538_890.4434	0.9871	0.6918		C34 H76 N4 O16 P2 S	890.4434	5.538								0	level5						other
5.542_680.3864	1.1903	0.101		C30 H56 N4 O13	680.3864	5.542								0	level5						other
5.545_670.3558	1.1653	0.2391		C32 H61 Cl O8 P2	670.3558	5.545								0	level5						other
5.587_354.0411	0.6789	0.5895	Dihydronaringenin-o-sulphate	C15 H14 O8 S	354.0411	5.587		S30778503	HMDB0059996	0.0002	0.5161			11	level4	Phenylpropanoids and polyketides	Linear 1,3-diarylpropanoids	Chalcones and dihydrochalcones	Polyketides[PK]		other
5.592_568.269	1.9433	0.0037		C27 H51 Cl O4 P2 S	568.269	5.592								0	level5						up
5.594_578.2976	1.7771	0.0011		C33 H42 N2 O7	578.2976	5.594								0	level5						up
5.596_214.03	1.2073	0.902		C9 H10 O4 S	214.03	5.596								0	level5						other
5.631_540.2751	0.0022	0.1857		C28 H44 O8 S	540.2751	5.631								0	level5						other
5.638_671.2685	2.4087	0.069		C23 H50 N3 O15 P S	671.2685	5.638								0	level5						other
5.663_714.3835	1.117	0.1932		C33 H66 Cl N2 O6 P3	714.3835	5.663								0	level5						other
5.663_724.412	1.2154	0.0607		C31 H64 O18	724.412	5.663								0	level5						other
5.687_232.0404	0.7288	0.5342	4-ethyl-2-methoxyphenyl hydrogen sulfate	C9 H12 O5 S	232.0404	5.687		S74852087	HMDB0127988	-0.0001	-0.6135			1	level4	Organic acids and derivatives	Organic sulfuric acids and derivatives	Arylsulfates	Organic acids		other
5.722_622.3252	1.7395	0.0076		C26 H54 O14 S	622.3252	5.722								0	level5						up
5.723_612.2966	1.9059	0.0072		C25 H50 Cl N6 O5 P S	612.2966	5.723								0	level5						up
5.761_715.2961	2.4127	0.0564		C27 H49 N5 O15 S	715.2961	5.761								0	level5						other
5.774_768.4397	1.198	0.1897		C32 H69 N2 O16 P	768.4397	5.774								0	level5						other
5.779_758.4112	1.0851	0.5725		C35 H70 Cl N2 O7 P3	758.4112	5.779								0	level5						other
5.838_656.3224	1.7462	0.0136	N-(5-amino-2-[(2,6-diamino-2,6-dideoxyhexopyranosyl)oxy]-3-{[3-o-(2,6-diamino-2,6-dideoxyhexopyranosyl)-beta-d-ribofuranosyl]oxy}-4-hydroxycyclohexyl)acetamide	C25 H48 N6 O14	656.3224	5.838	C02725	S388846		-0.0004	-0.6837			1	level4					NULL	up
5.839_666.351	1.7371	0.0059		C28 H58 O15 S	666.351	5.839								0	level5						up
5.874_759.3238	2.4836	0.0574		C32 H58 N O15 P S	759.3238	5.874								0	level5						other
5.877_812.4652	1.1898	0.2269		C34 H73 N2 O17 P	812.4652	5.877								0	level5						other
5.905_580.2771	1.0819	0.8287		C26 H51 N2 O4 P3 S	580.2771	5.905								0	level5						other
5.932_216.0457	0.1707	0.6411	3-phenylpropyl hydrogen sulfate	C9 H12 O4 S	216.0457	5.932		S74853792	HMDB0135313	0	0.1772			1	level4	Benzenoids	Benzene and substituted derivatives	null	Benzene and derivatives		other
5.938_275.1632	1.6104	0.2833	(-)-physostigmine	C15 H21 N3 O2	275.1632	5.938	C06535	S5763	HMDB0015116	-0.0002	-0.5647			1	level4	Organoheterocyclic compounds	Indoles and derivatives	Pyrroloindoles	Indole and derivatives		other
5.941_254.1154	1.9821	0.2385	Hostmaniane	C13 H18 O5	254.1154	5.941		S9359446	HMDB0032796	-0.0001	-0.2198			2	level4	Benzenoids	Benzene and substituted derivatives	Benzoic acids and derivatives	Benzene and derivatives		other
5.944_710.3786	1.7704	0.0022		C33 H65 N2 O6 P3 S	710.3786	5.944								0	level5						up
5.946_700.35	1.9139	0.0065		C32 H64 Cl N2 O4 P3 S	700.35	5.946								0	level5						up
5.957_526.2772	1.9398	0.328		C23 H38 N6 O8	526.2772	5.957								0	level5						other
5.97_856.4929	1.1635	0.2831		C35 H78 N4 O15 P2	856.4929	5.97								0	level5						other
5.976_254.1516	0.2827	0.1469		C14 H22 O4	254.1516	5.976								0	level5						other
5.977_803.3492	2.3715	0.0716		C28 H63 N5 O15 P2 S	803.3492	5.977								0	level5						other
6.041_230.1517	1.1232	0.6995	Dodecanedioic acid	C12 H22 O4	230.1517	6.041	C02678	MReference-1182	HMDB0000623	-0.0002	-0.6611		90.2	5	level2	Lipids and lipid-like molecules	Fatty Acyls	Fatty acids and conjugates	Fatty acyls[FA]	NULL	other
6.042_252.1336	1.0905	0.7738		C11 H27 P3	252.1336	6.042								0	level5						other
6.042_754.4062	1.7905	0.0039		C33 H62 N4 O13 S	754.4062	6.042								0	level5						up

6.07_847.3771	2.6554	0.0467	D-tryptophyl-d-alanyl-d-allothreonylglycyl-d-histidyl-l-phenylalanyl-d-methioninamide	C40 H53 N11 O8 S	847.3771	6.07		S30776676	HMDB0013017	-0.0028	-3.3509			1	level4	Organic acids and derivatives	Carboxylic acids and derivatives	Amino acids, peptides, and analogues	Amino acids, peptides, and analogues		up
6.111_527.2548	1.5031	0.3955		C27 H37 N5 O4 S	527.2548	6.111								0	level5						other
6.179_196.9203	2.588	0.2003	Tcpy	C5 H2 Cl3 N O	196.9203	6.179		S21541	HMDB0039853	0.0001	0.3409			1	level4	Organoheterocyclic compounds	Pyridines and derivatives	Halopyridines	Pyridine and derivatives		other
6.179_198.9173	2.4733	0.2065		C3 H4 Cl N O P2 S	198.9173	6.179								0	level5						other
6.254_346.0691	1.7817	0.2732	Syringetin	C17 H14 O8	346.0691	6.254	C11620	S4445230		0.0002	0.6879			29	level4	Flavonoids	Flavonoids	Flavonols	Flavonoids		other
6.271_424.1556	1.6864	0.2222		C21 H28 O7 S	424.1556	6.271								0	level5						other
6.288_346.2357	0.9289	0.8259	Sorbitan, monododecanoate	C18 H34 O6	346.2357	6.288		S17215359		0.0002	0.5138			4	level4						other
6.329_320.0356	0.4538	0.6709	2-hydroxy-5-[(2e)-3-phenyl-2-propenoyl]phenyl hydrogen sulfate	C15 H12 O6 S	320.0356	6.329		S74853826	HMDB0135412	0.0001	0.3367			1	level4	Phenylpropanoids and polyketides	Linear 1,3-diarylpropanoids	Chalcones and dihydrochalcones	Polyketides[PK]		other
6.427_254.1516	1.2581	0.458		C14 H22 O4	254.1516	6.427								0	level5						other
6.449_245.9154	3.8194	0.1662			245.9154	6.449								0	level5						other
6.449_247.9123	3.9013	0.1638		C6 H Cl N2 O3 S2	247.9123	6.449								0	level5						other
6.454_304.1344	1.0631	0.3579	Flupirtine	C15 H17 F N4 O2	304.1344	6.454		S48119		0.0009	2.9246			1	level4						other
6.463_428.187	1.6107	0.7792		C21 H32 O7 S	428.187	6.463								0	level5						other
6.568_242.1517	1.5603	0.2524	5-octyl-2-oxotetrahydro-3-furancarboxylic acid	C13 H22 O4	242.1517	6.568		S35013298	HMDB0030987	-0.0001	-0.3636			2	level4	Organoheterocyclic compounds	Lactones	Gamma butyrolactones	Gamma butyrolactones		other
6.596_195.1259	0.4349	0.1367	4-(2-aminopropoxy)-3,5-dimethylphenol	C11 H17 N O2	195.1259	6.596		S84217	HMDB0060954	0	0.0796			2	level4	Benzenoids	Phenols	4-alkoxyphenols	Phenols and derivatives		other
6.599_671.3531	0.8339	0.2379		C34 H49 N5 O9	671.3531	6.599								0	level5						other
6.613_426.1714	1.5761	0.7053		C21 H30 O7 S	426.1714	6.613								0	level5						other
6.63_295.1531	1.6691	0.2752	Butralin	C14 H21 N3 O4	295.1531	6.63	C18582	S33600		-0.0001	-0.2092			1	level4					NULL	other
6.64_186.1256	1.0157	0.867	Queen bee acid	C10 H18 O3	186.1256	6.64		S4472163		0	0.0763			32	level4						other
6.691_408.215	2.1119	0.2329	Cascarillin	C22 H32 O7	408.215	6.691	C09071	S390570	HMDB0036836	0.0002	0.5809			3	level4	Terpenoids	Diterpenoids (C20)	Clerodanes	Terpenoids	NULL	other
6.695_348.2514	0.8709	0.6571	9,10,12,13-tetrahydroxyoctadecanoic acid	C18 H36 O6	348.2514	6.695		S83772		0.0002	0.5998			1	level4						other
6.724_316.0618	0.0013	0.176	7-chloro-5-(4-hydroxyphenyl)-1-methyl-1h-1,5-benzodiazepine-2,4(3h,5h)-dione	C16 H13 Cl N2 O3	316.0618	6.724		S30778586	HMDB0060771	0.0003	0.8879			1	level4	Organoheterocyclic compounds	Benzodiazepines	null	Benzodiazepines		other
6.727_330.0741	0.0848	0.1921	Tricin	C17 H14 O7	330.0741	6.727	C10193	S4445019	HMDB0124861	0.0001	0.3847			30	level4	Phenylpropanoids and polyketides	Flavonoids	O-methylated flavonoids	Flavonoids	NULL	other
6.887_470.2339	1.1455	0.6541		C18 H39 N4 O6 P S	470.2339	6.887								0	level5						other
6.942_270.056	0.0749	0.0889	4-[(1e)-4-methyl-3-oxo-1-penten-1-yl]phenyl hydrogen sulfate	C12 H14 O5 S	270.056	6.942		S74853062		-0.0002	-0.743		43.1	1	level4						other
6.964_370.2358	0.4071	0.0606	Thromboxane b2	C20 H34 O6	370.2358	6.964	C05963	MReference-4408	HMDB0003252	0.0003	0.6955		85.1	9	level2	Lipids and lipid-like molecules	Fatty Acyls	Eicosanoids	Fatty acyls[FA]	map00590 Arachidonic acid metabolism; map01100 Metabolic pathways; map04726 Serotonergic synapse; map04976 Bile secretion;	other
7.006_188.1413	0.451	0.3595	3-hydroxydecanoic acid	C10 H20 O3	188.1413	7.006		MReference-200	HMDB0002203	0	0.0804		84.5	23	level2	Organic acids and derivatives	Hydroxy acids and derivatives	Medium-chain hydroxy acids and derivatives	Organic acids		other
7.171_382.1914	2.2934	0.7983		C21 H31 Cl O4	382.1914	7.171								0	level5						other
7.172_392.2201	2.3057	0.6765	Isodomedin	C22 H32 O6	392.2201	7.172	C09115	S390594		0.0002	0.5823			6	level4	Terpenoids	Diterpenoids (C20)	Kaurenes	Terpenoids	NULL	other
7.18_188.1413	0.712	0.2546	10-hydroxydecanoic acid	C10 H20 O3	188.1413	7.18	C02774	MReference-280	-	0	0.1039		80	23	level2	FA Fatty acyls	FA01 Fatty Acids and Conjugates	FA0105 Hydroxy fatty acids	Fatty acyls[FA]	NULL	other
7.195_278.1492	2.2562	0.2148		C13 H29 P3	278.1492	7.195							40.7	0	level5						other
7.222_258.1829	0.4182	0.1133	Tetradecanedioic acid	C14 H26 O4	258.1829	7.222		MReference-1652	HMDB0000872	-0.0002	-0.6523		85.4	9	level2	Lipids and lipid-like molecules	Fatty Acyls	Fatty acids and conjugates	Fatty acyls[FA]		other
7.222_280.1649	0.4308	0.1191		C13 H31 P3	280.1649	7.222							64.9	0	level5						other
7.275_296.1624	1.2567	0.4221	4-hydroxy-5-methoxy-4-[2-methyl-3-(3-methyl-2-buten-1-yl)-2-oxiranyl]-1-oxaspiro[2.5]octan-6-one	C16 H24 O5	296.1624	7.275	C09674	S251178	HMDB0038120	0	0.0802			4	level4	Organic oxygen compounds	Organooxygen compounds	Alcohols and polyols	Alcohols	NULL	other
7.365_376.1922	5.169	0.1386		C18 H32 O6 S	376.1922	7.365								0	level5						other
7.401_522.301	0.4223	0.5899		C29 H46 O6 S	522.301	7.401								0	level5						other
7.472_698.3729	1.3757	0.2362		C36 H55 N6 O4 P S	698.3729	7.472								0	level5						other
7.545_500.3135	0.9327	0.9084	11-deoxocucurbitacin i	C30 H44 O6	500.3135	7.545	C08807	S4444701		-0.0003	-0.5042			1	level4	Terpenoids	Triterpenoids (C30)	Cucurbitanes	Terpenoids	NULL	other
7.609_514.2961	1.6302	0.3521		C21 H47 N4 O6 P S	514.2961	7.609								0	level5						other

7.676_214.157	0.606	0.3799	3-oxolauric acid	C12 H22 O3	214.157	7.676	C02367	S388783	HMDB0010727	0.0001	0.4023			11	level4	Organic acids and derivatives	Keto acids and derivatives	Medium-chain keto acids and derivatives	Organic acids	NULL	other
7.683_250.1568	0.7916	0.8543	Gemfibrozil	C15 H22 O3	250.1568	7.683	C07020	S3345	HMDB0015371	-0.0001	-0.5457			32	level4	Benzenoids	Phenol ethers	null	Benzene and derivatives	NULL	other
7.69_474.3345	0.6252	0.1402	18-acetoxy-1alpha,25-dihydroxyvitamin d3	C29 H46 O5	474.3345	7.69		S7826460		0	-0.0807			1	level4						other
7.692_514.2961	2.7051	0.0607		C23 H42 N6 O5 S	514.2961	7.692								0	level5						other
7.698_360.1972	2.3433	0.3005		C18 H32 O5 S	360.1972	7.698							41.3	0	level5						other
7.7_516.3116	2.3401	0.0132	Probucol	C31 H48 O2 S2	516.3116	7.7	C07373	S4743	HMDB0015537	0.0021	4.0162			1	level4	Benzenoids	Benzene and substituted derivatives	Phenylpropanes	Benzene and derivatives	NULL	up
7.701_270.1829	2.2401	0.2198	3543	C15 H26 O4	270.1829	7.701		S54974	HMDB0040459	-0.0002	-0.6891			2	level4	Phenylpropanoids and polyketides	Macrolides and analogues	null	Polyketides[PK]		other
7.703_292.1649	2.2868	0.2158		C14 H31 P3	292.1649	7.703								0	level5						other
7.891_382.1792	2.4399	0.2707		C16 H26 N6 O3 S	382.1792	7.891								0	level5						other
7.892_360.1973	2.6006	0.2655		C18 H32 O5 S	360.1973	7.892							47.4	0	level5						other
7.895_568.3255	0.4331	0.3151	(3î±,5î²,6î±)-23-carboxy-3-hydroxy-24-norcholan-6-yl î²-d-glucopyranosiduronic acid	C30 H48 O10	568.3255	7.895		S169791		0.0008	1.3487			3	level4						other
7.897_465.3091	0.7408	0.2205	Glycocholic acid	C26 H43 N O6	465.3091	7.897	C01921	MReference-429	HMDB0000138	0.0001	0.1445		79.6	4	level2	Lipids and lipid-like molecules	Steroids and steroid derivatives	Bile acids, alcohols and derivatives	Bile acids, alcohols and derivatives	map00120 Primary bile acid biosynthesis; map01100 Metabolic pathways; map04976 Bile secretion; map04979 Cholesterol metabolism;	other
7.952_388.2616	0.1393	0.3516	3,7-diketo-5β-cholan-24-oic acid	C24 H36 O4	388.2616	7.952		S85006		0.0003	0.7242			34	level4						other
7.957_346.1394	1.5341	0.0615		C13 H23 N4 O5 P	346.1394	7.957								0	level5						other
7.957_470.3032	0.0041	0.0972	(2r,4ar,6bs,12as)-1-hydroxy-1,2,6b,9,9,12a-hexamethyl-10,13-dioxo-1,3,4,5,6,6a,6b,7,8,8a,9,10,11,12,12a,12b,13,14b-octadecahydro-4a(2h)-picenecarboxylic acid	C29 H42 O5	470.3032	7.957		S35014633	HMDB0038683	0	0.0252			1	level4	Organic oxygen compounds	Organooxygen compounds	Carbonyl compounds	Carbonyl compounds		other
7.957_538.29	0.0047	0.1264		C29 H47 O5 P S	538.29	7.957								0	level5						other
7.958_278.1517	1.0964	0.6407	Mono(2-ethylhexyl) phthalate (mehp)	C16 H22 O4	278.1517	7.958	C03343	MReference-2914	HMDB0013248	-0.0001	-0.3632		95.3	15	level2	Benzenoids	Benzene and substituted derivatives	Benzoic acids and derivatives	Benzene and derivatives	NULL	other
7.988_360.1973	2.6083	0.2538		C18 H32 O5 S	360.1973	7.988							55.7	0	level5						other
8.023_570.2869	4.3128	0.0444		C29 H46 O9 S	570.2869	8.023								0	level5						up
8.049_460.28	0.3625	0.3518		C21 H41 N4 O5 P	460.28	8.049								0	level5						other
8.087_554.3019	1.9479	0.6844		C28 H46 N2 O7 S	554.3019	8.087								0	level5						other
8.111_472.3189	0.0094	0.0988		C23 H45 N4 O4 P	472.3189	8.111								0	level5						other
8.113_540.3056	0.0136	0.0748		C29 H51 O3 P3	540.3056	8.113								0	level5						other
8.214_460.2801	0.4299	0.1655		C21 H41 N4 O5 P	460.2801	8.214								0	level5						other
8.214_806.5706	0.1233	0.1472		C49 H79 N2 O5 P	806.5706	8.214								0	level5						other
8.25_390.2773	0.299	0.2592	Bis(2-ethylhexyl) phthalate	C24 H38 O4	390.2773	8.25	C03690	S21106505		0.0003	0.8305			62	level4					NULL	other
8.252_376.283	0.0289	0.1483		C20 H40 O6	376.283	8.252								0	level5						other
8.348_408.2878	0.1574	0.2748	Cholic acid	C24 H40 O5	408.2878	8.348	C00695	MReference-344	HMDB0000619	0.0002	0.4812		85.1	84	level2	Lipids and lipid-like molecules	Steroids and steroid derivatives	Bile acids, alcohols and derivatives	Bile acids, alcohols and derivatives	map00120 Primary bile acid biosynthesis; map01100 Metabolic pathways; map04976 Bile secretion;	other
8.352_476.2749	0.1887	0.2569		C21 H41 N4 O6 P	476.2749	8.352								0	level5						other
8.383_595.3726	0.7164	0.4209		C31 H54 N3 O6 P	595.3726	8.383								0	level5						other
8.388_280.2038	0.5138	0.0884	12-hhtre	C17 H28 O3	280.2038	8.388		S4472190		-0.0001	-0.295			11	level4						other
8.391_472.3189	0.1925	0.2611		C29 H44 O5	472.3189	8.391								0	level5						other
8.42_556.3191	0.7699	0.4224		C27 H49 N4 O4 P S	556.3191	8.42								0	level5						other
8.439_498.3013	2.3195	0.1565		C21 H47 N4 O5 P S	498.3013	8.439								0	level5						other
8.461_458.3396	0.1332	0.1606	18-acetoxy-1alpha-hydroxyvitamin d3	C29 H46 O4	458.3396	8.461		S7826457		0	0.0837			6	level4						other
8.465_526.3266	0.1393	0.1606		C28 H42 N6 O4	526.3266	8.465								0	level5						other
8.488_672.4099	5.151	0.141		C39 H62 O5 P2	672.4099	8.488								0	level5						other
8.503_449.3143	0.3323	0.057	Glycodeoxycholic acid	C26 H43 N O5	449.3143	8.503	C05464	MReference-1291	HMDB0000631	0.0001	0.312	84.4	91	6	level2	Lipids and lipid-like molecules	Steroids and steroid derivatives	Bile acids, alcohols and derivatives	Bile acids, alcohols and derivatives	NULL	other
8.554_342.2772	0.1109	0.1501	Icosanedioic acid	C20 H38 O4	342.2772	8.554		S68030		0.0002	0.6591			1	level4						other
8.573_628.3842	1.8768	0.226		C34 H52 N4 O7	628.3842	8.573								0	level5						other
8.585_294.2195	0.266	0.3347	13(s)-hotre	C18 H30 O3	294.2195	8.585	C16316	MReference-9864		0	-0.1002		76.6	40	level2	FA Fatty acyls	FA02 Octadecanoids	FA0200 Other Octadecanoids	Fatty acyls[FA]	map00592 alpha-Linolenic acid metabolism;	other
8.594_360.2878	0.1307	0.1705		C20 H40 O5	360.2878	8.594								0	level5						other
8.639_312.2301	0.7071	0.7087	(+/-)9-hpode	C18 H32 O4	312.2301	8.639		MReference-9923		0.0001	0.301		79.3	35	level2						other

8.745_316.2615	0.7502	0.539	9,10-dihydroxystearic acid	C18 H36 O4	316.2615	8.745	C19622	S80658		0.0002	0.482			22	level4						other
8.75_344.2929	0.1271	0.1599	Mfcd00273074	C20 H40 O4	344.2929	8.75		S96316		0.0002	0.6711			10	level4						other
8.856_266.155	1.2379	0.797	Laurilsulfate	C12 H26 O4 S	266.155	8.856		S8448		-0.0002	-0.788			1	level4						other
8.93_314.2458	0.0927	0.1689	(+/-)12(13)-dihome	C18 H34 O4	314.2458	8.93	C14829	MReference -6638	HMDB0004705	0.0001	0.3545		73.8	10	level2	Lipids and lipid-like molecules	Fatty Acyls	Fatty acids and conjugates	Fatty acyls[FA]		other
8.961_806.5706	0.2035	0.1905		C50 H78 O8	806.5706	8.961								0	level5						other
8.963_392.2931	0.2602	0.229	Deoxycholic acid	C24 H40 O4	392.2931	8.963	C04483	MReference -388	HMDB0000626	0.0004	1.0929		92.2	54	level2	Lipids and lipid-like molecules	Steroids and steroid derivatives	Bile acids, alcohols and derivatives	Bile acids, alcohols and derivatives	map04976 Bile secretion;	other
8.963_460.2801	0.5771	0.1766		C21 H41 N4 O5 P	460.2801	8.963								0	level5						other
8.992_251.152	0.9429	0.4461	Lt9970000	C14 H21 N O3	251.152	8.992	C18912	S39512		-0.0002	-0.6004			1	level4					NULL	other
9.037_298.2508	1.1123	0.7307	Ricinelaidic acid	C18 H34 O3	298.2508	9.037		S393218		0	0.1061			45	level4						other
9.056_320.2352	0.3405	0.0785	(+/-)11(12)-eet	C20 H32 O3	320.2352	9.056		MReference -9807		0.0001	0.3392		86.4	61	level2						other
9.059_388.2227	0.3687	0.0285		C19 H28 N6 O3	388.2227	9.059								0	level5						down
9.101_310.1815	1.063	0.6879		C14 H30 O5 S	310.1815	9.101								0	level5						other
9.135_456.3241	0.7226	0.1187	Callystatin a	C29 H44 O4	456.3241	9.135	C16891	S4581330		0.0001	0.2419			7	level4					NULL	other
9.147_280.1708	2.4847	0.9678		C13 H28 O4 S	280.1708	9.147							48.3	0	level5						other
9.184_322.2509	0.0567	0.0947	15-oxoede	C20 H34 O3	322.2509	9.184		MReference -9905		0.0001	0.4308		71.2	13	level2						other
9.221_354.2078	1.1014	0.811		C16 H34 O6 S	354.2078	9.221								0	level5						other
9.287_398.2341	1.4177	0.8525		C18 H38 O7 S	398.2341	9.287								0	level5						other
9.332_442.2602	1.8234	0.6244		C21 H38 N4 O4 S	442.2602	9.332								0	level5						other
9.363_486.2861	2.0596	0.6211		C22 H46 O9 S	486.2861	9.363								0	level5						other
9.372_526.3266	0.1429	0.1488		C26 H47 N4 O5 P	526.3266	9.372								0	level5						other
9.419_482.3606	0.8517	0.575		C22 H51 N4 O5 P	482.3606	9.419								0	level5						other
9.422_513.3063	0.5601	0.2181		C24 H44 N5 O5 P	513.3063	9.422								0	level5						other
9.455_563.3232	0.3354	0.225		C28 H46 N5 O5 P	563.3232	9.455								0	level5						other
9.546_272.235	0.3667	0.2419	16-hydroxyhexadecanoic acid	C16 H32 O3	272.235	9.546	C18218	MReference -2551	HMDB0006294	-0.0002	-0.5557		92.3	21	level2	Lipids and lipid-like molecules	Fatty Acyls	Fatty acids and conjugates	Fatty acyls[FA]	map01100 Metabolic pathways;	other
9.554_539.3217	0.5046	0.2615		C26 H46 N5 O5 P	539.3217	9.554								0	level5						other
9.594_434.2436	0.3834	0.1523	1-linoleyl-sn-glycerol 3-phosphate	C21 H39 O7 P	434.2436	9.594		S24766514		0.0003	0.6467			5	level4						other
9.604_565.3389	0.5947	0.2501		C27 H52 N O9 P	565.3389	9.604								0	level5						other
9.608_633.3272	0.7051	0.2602		C30 H52 N O11 P	633.3272	9.608								0	level5						other
9.664_595.3103	0.5804	0.114		C31 H50 N O6 P S	595.3103	9.664								0	level5						other
9.667_527.3219	0.5399	0.1958		C24 H50 N O9 P	527.3219	9.667								0	level5						other
9.682_589.3386	0.5225	0.2349		C30 H48 N5 O5 P	589.3386	9.682								0	level5						other
9.707_565.3389	0.5829	0.2692		C27 H52 N O9 P	565.3389	9.707								0	level5						other
9.715_633.3272	1.0951	0.3578		C30 H52 N O11 P	633.3272	9.715								0	level5						other
9.778_541.3374	0.5445	0.173		C26 H48 N5 O5 P	541.3374	9.778								0	level5						other
9.778_542.3402	0.534	0.1824		C22 H53 N6 O3 P3	542.3402	9.778								0	level5						other
9.783_609.3257	0.5962	0.0661		C32 H54 N O4 P3	609.3257	9.783								0	level5						other
9.857_591.3542	0.3598	0.2519		C30 H50 N5 O5 P	591.3542	9.857								0	level5						other
9.87_567.3544	0.5803	0.2095		C28 H50 N5 O5 P	567.3544	9.87								0	level5						other
9.884_541.3374	0.515	0.1602		C25 H52 N O9 P	541.3374	9.884								0	level5						other
9.885_1036.6733	0.494	0.1314		C49 H102 N2 O16 P2	1036.6733	9.885								0	level5						other
9.896_453.2858	0.5038	0.1218	Glycerophospho-n-palmitoyl ethanolamine	C21 H44 N O7 P	453.2858	9.896		MReference -2955		0.0003	0.5554		50.2	2	level3						other
9.897_609.3257	1.3117	0.3962		C28 H52 N O11 P	609.3257	9.897								0	level5						other
9.917_328.2405	0.8937	0.3146	Docosahexaenoic acid	C22 H32 O2	328.2405	9.917	C06429	MReference -6801	HMDB0002183	0.0002	0.6936		82.3	15	level2	Lipids and lipid-like molecules	Fatty Acyls	Fatty acids and conjugates	Fatty acyls[FA]	map01040 Biosynthesis of unsaturated fatty acids;	other
9.972_372.2279	0.6833	0.1001		C17 H33 N4 O3 P	372.2279	9.972								0	level5						other
9.974_567.3544	0.5088	0.2316		C27 H54 N O9 P	567.3544	9.974								0	level5						other
9.975_635.3429	0.6819	0.1705		C30 H54 N O11 P	635.3429	9.975								0	level5						other
9.978_304.2403	0.6264	0.1302	Arachidonic acid	C20 H32 O2	304.2403	9.978	C00219	MReference -2742	HMDB0001043	0.0001	0.2429		87.9	36	level2	Lipids and lipid-like molecules	Fatty Acyls	Fatty acids and conjugates	Fatty acyls[FA]	map00590 Arachidonic acid metabolism; map00591 Linoleic acid metabolism; map01040 Biosynthesis of unsaturated fatty acids; map01100 Metabolic pathways; map04216 Ferroptosis; map04217 Necroptosis; map04270 Vascular smooth muscle contraction; map04611 Platelet activation; map04664 Fc epsilon RI signaling pathway; map04666 Fc gamma R-mediated phagocytosis; map04723 Retrograde endocannabinoid signaling; map04726 [illegible]	other

Compound.ID is consisted of Retention time and Molecular Weight. The metabolite ID in the column starting with BGI is the ID in the BGI Library database; the metabolite ID starting with M is the ID in the mzCloud Library database; the metabolite ID starting with S is the ID in the ChemSpider Library database. The mzVault.Best.Match refers to compound's secondary spectrum matching score in the BGI self-built standard library. The mzCloud.Best.Match refers to compound's secondary spectrum matching score in the mzCloud standard library. The meanings of different levels are as follows: Level 1: Substances can be accurately identified from the standard database and laboratory data. Level 2: Structural formula can match the standard database. Level 3: The structural formula partly can match the standard database, but needs further verification. Level 4: The accurate MS1 molecular weight can match the database. Level 5: There are no matches and no identification results in the database. The Super.class, Class and Sub.class refer to the second, third and fourth level of HMDB database classification respectively. The lable means differential metabolite filter labels, up or down are differential metabolites, other is not a differential metabolite.
