# Supplementary material for: Metabolic Profiling of Serum for Osteoarthritis Biomarkers
Source: Dis Markers. 2022 Jul 28;2022:1800812. doi: 10.1155/2022/1800812 (PMC9356247; doi:10.1155/2022/1800812)
Supplement: Supplementary 3 — Table S3. Cluster analysis results in positive ion mode. [file 1800812.f3.pdf]

Table S3 Cluster analysis results in positive ion mode									
Compound.ID	control-1	control-2	control-3	case-5	case-1	case-3	case-4	case-2	case-6
9.693_348.2037	0.559581496	1.098523275	0.775660645	0.585978664	0.568148126	-0.915426591	-0.833786691	-1.916517043	0.077838119
9.098_263.2613	0.141366744	1.460910594	1.448121624	0.359908602	-0.109332033	-0.303226051	-0.4267939	-1.404234642	-1.166720938
1.021_165.0651	0.686922522	1.277962702	1.207264791	0.912535195	-0.63285414	-0.760935035	-0.94878479	-1.193193139	-0.548918106
5.884_260.0685	0.518145156	1.504866936	1.348274239	0.595392878	-0.762788578	-0.691914884	-1.04502756	-0.551923435	-0.915024753
7.215_266.1155	0.232869609	1.727653148	1.372311014	0.410047195	-0.504533112	-0.762797811	-0.7384313	-0.966345908	-0.770772835
8.918_365.2929	0.153701854	1.496868537	1.027442781	0.679456718	0.328198774	-1.640892545	-0.705200731	-0.778595464	-0.560979924
9.684_342.1867	0.442152259	1.423704057	0.929220197	0.724697468	0.031104567	-1.431885534	-0.76810837	-1.321674764	-0.029209878
1.926_183.0533	0.707163427	1.365431561	0.513711944	1.20287071	0.110059401	-1.262985772	-0.885275264	-0.895509498	-0.855466508
9.103_475.3664	0.213022438	1.080355305	1.263174184	1.173638676	0.145235875	-0.929440078	-0.986751655	-0.844551538	-1.114683206
5.453_414.2469	1.6208717	1.378865959	0.335855125	0.133629888	-0.591770675	-1.493771306	-0.598502917	-0.608047618	-0.177130155
8.44_475.3664	0.796166794	1.265216544	1.2414719	-0.625419819	0.481639236	-1.440038279	-0.094138344	-0.497609634	-1.127288398
0.609_188.1527	1.066326895	1.560101473	1.20832474	-0.748137115	-0.582484216	-0.668127042	-0.838720338	-0.958452502	-0.038831895
3.162_230.163	1.304650813	1.0400339	1.149467651	-1.435572392	0.194933351	-0.745450475	-0.428222123	-1.026733553	-0.053107171
0.712_101.1206	0.435175547	1.705084316	1.121330133	0.023073203	-1.453656519	-0.291352273	-0.393780513	-0.009103604	-1.13677029
8.555_400.259	0.362864704	1.578543914	1.470213662	-0.478015181	-0.118006329	-0.386640522	-1.127585627	-0.057034253	-1.244340368
8.555_342.2559	0.343862231	1.569842654	1.519146765	-0.606566206	-0.133372573	-0.440442427	-1.057037086	-0.006959655	-1.188473702
8.555_360.2665	0.350593822	1.56839708	1.519885478	-0.62243868	-0.150066759	-0.403299628	-1.07161896	-0.014169807	-1.177282545
4.455_242.1267	0.453336902	1.892958262	1.097231843	-0.602316888	0.100217507	-0.254385585	-0.940260729	-1.156253727	-0.590527585
0.702_175.121	0.219568722	1.532279573	1.564868633	-0.107821272	-0.065224727	-0.161651054	-1.064750987	-1.22455702	-0.692711869
0.708_157.1104	0.23767975	1.540589076	1.572374758	-0.215491289	-0.048800842	-0.144160094	-1.050755579	-1.205793999	-0.68564178
5.729_129.058	0.467932506	0.721387278	1.663721179	-1.683106667	0.358094677	0.404467429	-0.37360826	-0.697748296	-0.861139846
5.729_189.0791	0.393048792	0.692943257	1.576952527	-1.843458163	0.416641937	0.440564429	-0.216994085	-0.736591874	-0.723106822
3.819_327.1526	0.506825749	1.099473845	1.480633308	-0.376317563	0.493788353	0.04091537	-0.691901108	-0.947926949	-1.605491007
4.112_371.179	0.889459458	0.668887634	1.277989313	-0.222133573	0.381767328	0.467756658	-0.47636788	-1.60598608	-1.381372857
0.538_178.974	1.074559547	0.851576275	0.87180349	-0.594279774	-1.456525824	1.115786397	-0.106853283	-1.086815739	-0.669251088
5.101_604.3073	0.874610306	0.969756367	1.153675459	-1.248127641	-1.335840491	0.001327344	0.681037863	-1.075697617	-0.02074159
0.595_530.0068	0.467442647	1.313172761	0.75164556	-1.969651331	-0.055368588	0.579620139	-1.128087513	0.02838586	0.012840465
6.649_108.094	1.088229436	1.141662456	0.324041451	-1.989630884	-0.569610175	0.366432385	-0.895766813	0.416789923	0.117852221
7.941_194.1672	0.166416884	1.296546575	1.323806122	-1.67152875	-1.298234558	0.188213221	-0.115856315	0.102335777	0.008301044
0.61_105.9433	0.582683836	1.971219309	0.789201709	-1.380732771	-0.605986649	0.078447599	-0.436603577	-0.19639034	-0.801839116
0.606_257.8919	0.761884947	1.765043232	0.635326143	-1.684507118	-0.544178558	-0.093509957	-0.85940714	-0.088580413	0.107928864
0.602_377.8928	0.620108884	1.965514918	0.759222625	-1.445620254	-0.860655114	-0.215797538	-0.492438924	-0.193687446	-0.136647151
0.607_173.9308	0.530395532	1.957186784	0.677927952	-1.580658411	-0.750062286	0.019452711	-0.529367671	-0.292935245	-0.031939365
1.134_334.9702	-0.669972703	-0.86116435	-0.91212138	-1.110545526	0.829744496	1.495498818	1.105560524	-0.481378625	0.604378745
6.322_131.0406	-0.693421792	-1.280868973	-0.616590494	-0.386237838	1.747629238	1.320375082	0.572361847	-0.286720017	-0.376527055
5.655_131.0406	-0.757136091	-1.291313297	-0.618460171	-0.39446125	1.477138742	1.343620395	0.97986077	-0.353255709	-0.38599339
6.017_131.0406	-0.775539399	-1.184836791	-0.59844539	-0.616426122	1.384807305	1.289163863	1.19762831	-0.357693399	-0.338658377
0.646_139.0609	-0.427280138	-1.127245893	-1.115159941	-0.035768454	0.888324659	1.676062419	1.090042467	-0.294054875	-0.654920244
6.014_562.3137	-0.790264001	-1.4501116	-0.764145946	-0.201600866	1.343224741	1.266620721	1.057653826	-0.315690014	-0.145686861
5.492_386.2086	-0.765817834	-1.430700697	-0.942735193	-0.340235512	1.086344166	1.439351893	1.0474049	-0.11311043	0.019498706

5.306_131.0406	-0.649915984	-1.29639178	-0.8384263	-0.522405653	1.216442024	1.514241064	1.02326571	-0.195456799	-0.251352283
5.494_131.0406	-0.748637235	-1.286137362	-0.868255617	-0.378388121	1.171759367	1.494933388	1.081985661	-0.251557367	-0.215702715
5.907_518.2876	-1.008375761	-1.444741385	-0.715513414	-0.186277626	0.999779038	1.436925663	1.119752045	-0.027382138	-0.174166424
5.789_474.2613	-0.761908461	-1.470307489	-0.53842985	-0.42340432	1.139129526	1.407796535	1.170871956	-0.28319568	-0.240552217
5.908_131.0406	-0.791673185	-1.257767702	-0.745398219	-0.386734448	1.030764169	1.392566289	1.373377678	-0.24043022	-0.374704362
4.224_158.0369	-0.242742771	-1.169494229	-1.722711405	-0.299863225	0.73970394	1.004933594	1.121348929	-0.234937371	0.803762539
7.461_253.1681	-0.29564058	-1.379091221	-1.672695215	-0.446975654	0.936912715	0.639484249	0.745915966	0.528262217	0.943827523
0.664_174.0503	-1.772035941	-0.332988906	-1.216421278	-0.323549077	1.131729791	0.729224486	0.824470753	0.154268472	0.8053017
2.215_338.1478	-1.359467453	-0.520623623	-1.669546249	-0.153264743	0.361699865	0.670206888	0.94560132	0.631108584	1.094285412
0.667_251.1004	-1.506421584	-0.867993817	-0.322363094	-1.164967522	0.778026958	1.039139528	0.956627493	0.946543137	0.141408902
0.568_308.1583	-0.607599186	-0.561553874	-1.300393117	-1.516349113	0.510783233	0.90473572	0.74168237	1.02284537	0.805848597
0.659_262.0774	-0.866380245	-0.3999731	-1.372744716	-1.393524099	0.827955336	0.738352841	0.843276242	0.85709182	0.765945922
0.66_222.0852	-0.699159661	-0.490090138	-1.547790588	-1.279111288	0.821977479	0.735327414	0.833133884	0.873144786	0.752568112
0.653_336.1644	-1.089184434	-0.625255994	-0.802882177	-1.42678974	0.303156709	0.648525171	0.725606181	1.392394841	0.874429442
0.679_281.111	-0.923949686	-1.012118403	-0.843035334	-1.318919453	0.774409507	0.682943091	0.836729866	1.275023484	0.528916926
5.182_517.3102	-1.303016489	-1.689897017	-0.605212219	0.026071907	0.414350125	0.430450481	0.790224453	1.301655484	0.635373274
0.721_213.007	-0.786274814	-1.310140591	-1.56999643	0.036662035	1.236146143	0.335683211	0.435525408	0.973316211	0.649078827
0.726_191.0253	-0.610099406	-0.818023684	-1.766902247	-0.347372942	0.581040251	-0.013342884	0.628388225	1.51282918	0.833483508
3.476_232.1215	-1.022288418	-0.468656573	-1.222504517	0.610077937	1.635128507	-0.692673944	0.349706602	-0.389318293	1.200528699
4.62_385.2313	-0.802707943	-1.352861019	-0.779936601	0.477468071	0.932919457	0.578839823	-0.009086381	-0.752625103	1.707989695
4.609_589.7004	-0.630046211	-0.651610946	-1.446398987	1.500501826	-0.502089579	1.416671462	0.587960919	-0.447582805	0.172594321
4.608_589.1976	-0.707683224	-0.700297932	-1.528840059	1.691633825	-0.429996262	0.985635049	0.493039882	-0.343911299	0.540420019
4.609_577.2753	-0.564362395	-0.675300609	-1.393645291	1.856048732	-0.329978131	1.120438836	0.436960724	-0.526555478	0.076393612
4.613_553.7362	-0.917450944	-0.436793426	-1.379255562	1.851582921	-0.326770449	1.037107723	0.329922011	-0.487236096	0.328893824
3.057_225.1002	-1.152692861	-0.464030907	-1.756141215	1.516290543	0.331992256	0.413201023	0.449305622	-0.099545562	0.761621101
4.93_279.1093	-1.369004236	-0.455307983	-1.330214907	0.821413436	0.319020598	1.013337729	-0.235137859	-0.255751556	1.491644778
3.772_437.1933	-1.146855085	-1.6900147	-0.232256112	0.650526528	1.308534997	1.06196359	0.599180023	-0.258885933	-0.292193309
4.506_417.2575	-1.282564126	-1.636825672	-0.467698478	1.179034384	0.845237007	1.132507647	-0.102607895	0.121611154	0.211305978
0.742_131.98	-0.162536654	-1.312848787	-1.644765168	1.225517066	0.919015011	0.913077886	0.491125457	-0.339828976	-0.088755835
0.699_185.1053	-0.442531483	-1.089494394	-1.112324472	2.148545689	0.213934983	0.681980342	0.102978203	-0.491996576	-0.011092291
5.184_465.2244	-0.98249478	-1.315986293	-0.411257138	1.923513438	0.857340197	0.338568407	0.356852431	-0.668797767	-0.097738495
1.272_122.0369	-0.413033308	-0.95435767	-1.283067748	0.883037763	1.444452511	-1.061088802	0.565419488	0.968537442	-0.149899676
2.701_170.1533	-0.982693986	-1.301898447	-0.282540456	1.170412842	0.602695836	-0.630263086	1.586695529	0.465793547	-0.628201779
2.745_189.0396	-0.453099959	-0.820231442	-1.030089788	1.130076321	-0.303261207	-0.762595685	1.221941063	1.533250811	-0.515990113
2.822_189.0395	-0.503136005	-1.200536659	-0.969712849	1.016326688	-0.119008307	-0.743139427	1.318923187	1.408221292	-0.207937919
3.163_216.1408	-0.686743175	-0.723728546	-1.315697068	1.663098768	0.179910165	-0.194628361	-0.763003935	1.204089371	0.63670278
6.139_459.277	-1.173816066	-0.744955489	-0.39337807	1.737085441	-0.924202934	-0.494757334	0.107725071	0.995023467	0.891275912
4.797_373.2133	-0.856858275	-0.863229265	-0.480521098	1.836724382	-1.246610385	0.362876528	-0.073262569	0.240484967	1.080395715
4.981_364.1917	-0.90871338	-1.068451241	-0.33079829	1.87325141	-1.04548191	-0.194500922	0.310862118	0.323700994	1.040131223
5.559_540.2972	-0.869379904	-1.171389269	-0.481361992	1.887503679	-0.764904847	-0.135569673	0.076230566	0.279867227	1.179004213
3.194_159.0717	-0.580010684	-1.15764173	-1.203857762	-0.543584999	-0.206535597	0.065909902	0.898186865	1.414921112	1.312612892
5.285_324.2024	-0.453838034	-1.10535625	-1.007786382	-0.05636623	-0.686044932	-0.362963763	0.721930106	1.278592086	1.671833399

5.285_631.378	-0.303998939	-1.191715544	-0.942708971	0.037929555	-0.681298842	-0.518862664	0.655875787	1.251214845	1.693564773
5.023_560.3523	-0.417099906	-1.143708772	-1.117897962	-0.014311731	-0.629526574	-0.29172125	0.66893491	1.443595607	1.501735679
5.601_390.2416	-0.60501489	-1.271091125	-0.905156112	-0.019423929	-0.611688487	-0.233230338	0.778106572	1.270474626	1.597023684
4.05_368.2524	-0.577162214	-1.21137093	-1.216850646	0.082095296	-0.401030576	-0.281183273	0.903104402	1.301025868	1.401372074
3.763_324.2259	-0.336596677	-1.240295762	-1.231132404	0.004985893	-0.474054019	-0.294799982	0.821766377	1.196003334	1.554123241
4.869_499.2996	-0.441071127	-1.250133942	-1.189447651	0.068690268	-0.454599828	-0.281253586	0.721632449	1.305986633	1.520196784
5.021_271.6629	-0.479549336	-1.285002106	-1.102125412	-0.075710493	-0.42608893	-0.245508088	0.806781839	1.222668161	1.584534365
5.16_293.6759	-0.472479318	-1.181547771	-1.12972702	-0.089054004	-0.512566672	-0.275549511	0.808163113	1.292979916	1.559781266
5.022_543.3258	-0.484812011	-1.259478937	-1.042412144	-0.022302195	-0.543680299	-0.272937227	0.744840914	1.347583354	1.533198544
5.161_587.3518	-0.472294608	-1.264631553	-1.008678185	-0.076864815	-0.524582572	-0.342968582	0.873870017	1.280618143	1.535532155
3.763_279.1681	-0.224501443	-1.631124315	-1.0177397	0.048786672	-0.401610739	-0.155930372	0.737115556	1.213210041	1.431794301
3.424_235.1421	-0.326605114	-1.446376295	-1.149209974	0.106273512	-0.457650941	-0.171360086	0.740219629	1.240803723	1.463905547
4.051_323.1944	-0.358714333	-1.325795102	-1.212138395	0.100457328	-0.483706427	-0.21568493	0.744062725	1.29011436	1.461404774
4.508_411.247	-0.397499632	-1.328788623	-1.223102084	0.046777571	-0.45002931	-0.156563627	0.778719114	1.273244752	1.45724184
4.295_367.2207	-0.291511435	-1.439923213	-1.262028606	0.06282785	-0.348437542	-0.145328776	0.812075986	1.181946872	1.430378864
4.697_455.2732	-0.336251313	-1.367101878	-1.253108016	-0.054549652	-0.309820003	-0.195645387	0.818433542	1.327856447	1.37018626
5.391_460.2633	-0.242231859	-1.599986823	-1.593890668	1.018816095	0.316078817	0.155743001	1.17467508	0.415443501	0.355352855
5.698_503.2582	-0.319164397	-1.762903465	-1.437679286	0.968998187	0.228134515	0.151129138	1.008384283	0.671827893	0.491273132
3.286_244.1358	-1.027300393	-1.629934308	-0.599306208	0.989681435	0.13843212	-0.481258123	1.280243022	0.961223443	0.368219012
4.493_289.1395	-0.397713738	-1.521733919	-1.111031109	1.094305024	-0.36734065	-0.437487614	1.177094063	1.064325205	0.499582738
5.549_368.2285	-1.013213519	-1.155450576	-1.059534279	0.811373641	-0.981510952	0.868895182	0.822212649	0.800216536	0.907011316
5.384_533.3416	-1.138056703	-1.779905452	-0.473163171	1.224964712	-0.163647613	0.25909201	0.938417347	0.252497638	0.879801232
5.701_520.2848	-1.632594572	-1.240676123	-0.963717103	0.930440043	0.352563168	0.28962569	0.974965295	0.718450816	0.570942786
Compound.ID is the ID of the differential metabolites, consisting of Retention time and Molecular Weight. Others are the samples names. The metabolite order in the table is the metabolite order in the heatmap map (from top to bottom). The order of the samples in the table is the order of the samples in the heatmap (from left to right).									
