# Supplementary material for: Metabolic Profiling of Serum for Osteoarthritis Biomarkers
Source: Dis Markers. 2022 Jul 28;2022:1800812. doi: 10.1155/2022/1800812 (PMC9356247; doi:10.1155/2022/1800812)
Supplement: Supplementary 4 — Table S4. Cluster analysis results in negative ion mode. [file 1800812.f4.pdf]

Table S4 Cluster analysis results in negative ion mode									
Compound.ID	control-2	control-1	control-3	case-5	case-2	case-6	case-1	case-3	case-4
0.861_309.1092	-0.552085463	-1.891045407	-0.810266679	-0.602041051	0.978755006	0.669812778	0.541158488	0.750997811	0.914714518
0.745_117.079	-1.455222579	-0.389857762	-1.711457948	0.042167596	0.889461397	0.429454065	0.823925687	1.005529691	0.365999852
8.023_570.2869	-1.435238773	-0.142447402	-1.340023808	-0.549675301	1.469646778	0.017627745	0.962820092	0.838309246	0.178981423
0.771_294.0562	-1.911483151	-1.10312368	-0.453852493	0.629966017	0.253396151	0.714043212	-0.052402753	0.738283897	1.185172799
5.293_490.2444	-1.523281077	-1.092225512	-0.754534944	1.5857148	-0.10914735	0.550219536	0.257926751	1.007795427	0.077532369
6.042_754.4062	-1.433408855	-1.102589831	-0.653069171	1.79699949	-0.11075097	0.574877553	-0.081771416	0.832523576	0.177189625
4.907_402.1923	-1.565028832	-0.817890839	-0.783864751	1.691560093	-0.25975209	0.369821083	0.026145652	1.059712474	0.279297208
5.454_534.2701	-1.483919594	-0.854938516	-0.708686194	1.542946368	-0.273735524	0.546923357	-0.338270524	1.266354216	0.303326411
5.723_612.2966	-1.728872832	-0.759794514	-1.002478479	1.462878486	0.121206765	0.642097026	0.048275915	0.852536192	0.364151441
5.838_656.3224	-1.811926746	-0.949396595	-0.732397726	1.308425741	-0.114405513	0.669726958	0.219647358	0.882370757	0.527955766
5.946_700.35	-1.720434413	-0.79893636	-0.94022644	1.370413833	-0.220289895	0.59401744	0.180168395	0.884578573	0.650708868
5.592_568.269	-1.58608974	-1.118241503	-0.702943153	1.538271373	-0.140054313	0.50824874	0.037323944	0.623972546	0.839512107
5.839_666.351	-1.621642029	-1.007340297	-0.682838749	1.555218368	-0.18241292	0.807411841	-0.063728526	0.780573752	0.414758559
5.594_578.2976	-1.482563199	-0.928992758	-0.969610773	1.644180259	-0.139911504	0.689160699	-0.025634948	0.785842628	0.427529596
5.944_710.3786	-1.397402931	-1.047064528	-0.785156294	1.688987635	-0.302362485	0.708479146	-0.1384118	0.808324135	0.464607121
5.116_446.2184	-1.317696849	-1.350058157	-0.445664206	1.7405599	0.01800166	0.87567576	-0.057885467	0.654635439	-0.117568082
5.722_622.3252	-1.371768982	-0.812049974	-0.74259593	1.629182359	0.075193731	0.787475211	-0.787876806	0.971386411	0.25105398
3.922_122.0368	-1.563262283	-0.21885144	-1.148031479	1.527929686	0.803605372	0.357289804	0.537266735	0.464352042	-0.760298437
4.728_451.136	-1.786530207	-0.42424354	-0.818428515	1.162594761	0.956804351	0.757206288	0.103766511	-0.719251352	0.768081702
6.07_847.3771	-1.679851884	-0.206977227	-1.069445831	1.412179671	0.887183663	0.485651791	0.175857083	-0.689104089	0.684506823
0.69_216.0609	-0.590258898	-1.258946486	-0.833548153	1.662498794	-0.927084233	0.277432839	1.258785703	0.240473025	0.17064741
7.7_516.3116	-0.843445907	-0.569518142	-1.422085881	1.010467874	-0.149998662	0.149239159	1.886745197	0.365789004	-0.427192642
0.726_279.9313	-0.589509548	-0.628638005	-1.637414764	1.168984983	-0.846426032	-0.037732958	0.422699796	1.01891215	1.129124377
0.816_159.9858	-1.332218796	-0.496017736	-1.522951435	0.751597637	-0.33075771	0.628928894	0.052435418	0.953884399	1.295099328
0.582_176.0321	-1.161877921	-0.961143647	-1.383681294	0.243467063	0.074609656	0.214156573	1.415773852	1.270153851	0.288541868
3.482_258.1466	-1.069701917	-1.351852113	-1.488677836	1.059173711	0.373621994	0.66603088	0.517429654	0.67948847	0.614487158
9.059_388.2227	1.409879228	0.323679065	0.956438801	0.623939175	-0.578515533	-0.54313406	0.304034222	-1.866818876	-0.629502023
0.718_433.8725	1.445035289	1.275479078	0.219124023	-0.363444876	0.291147782	0.496246537	-1.095635258	-1.105080706	-1.162871868
0.721_443.9012	1.115396121	0.555848262	1.227909786	-0.161180199	-0.055943781	0.765925338	-1.630935237	-0.828885215	-0.988135075
0.635_182.9734	1.764154809	0.390134084	1.084740911	0.024556702	-0.354878421	-0.652949121	-0.592501869	-0.034215375	-1.62904172
3.479_154.0266	1.009526793	1.279999785	1.137044814	-1.115987644	-0.350466975	-0.103077864	-0.048438487	-0.185676283	-1.622924138
4.661_604.3072	1.185692747	1.009470168	0.809635818	-1.069875084	-1.235361921	-0.178239912	-1.270950448	-0.025675827	0.775304459
0.71_239.9389	0.42913402	0.841205149	1.110827273	0.141179844	-1.289702302	-1.966783252	-0.062075328	0.429318944	0.366895653
0.713_365.8853	0.534953633	1.162417549	1.233122598	0.326088437	-0.193062096	-1.799516064	-0.454897085	-1.084956161	0.275849189
Compound.ID is the ID of the differential metabolites, consisting of Retention time and Molecular Weight. Others are the samples names. The metabolite order in the table is the metabolite order in the heatmap map (from top to bottom). The order of the samples in the table is the order of the samples in the heatmap (from left to right).									
