# Supplementary material for: Metabolic Profiling of Serum for Osteoarthritis Biomarkers
Source: Dis Markers. 2022 Jul 28;2022:1800812. doi: 10.1155/2022/1800812 (PMC9356247; doi:10.1155/2022/1800812)
Supplement: Supplementary 5 — Table S5. Data used for ROC analysis. [file 1800812.f5.pdf]

Table S5 Data used for ROC analysis									
ESI-									
	case-1	case-2	case-3	case-4	case-5	case-6	control-1	control-2	control-3
Ascorbic acid	5610905.901	3112211.636	5263093.889	3418995.363	3351937.369	3309039.335	1974210.863	1807519.539	1639650.871
L-(+)-valine	70101967.83	72268788.04	76272030.09	56669910.29	48755726.05	58365093.52	39890810.91	24319582.22	21590643.09
2-hydroxy-1,2-diphenylethyl hydrogen sulfate	6258487.268	6594152.597	7163687.282	7732052.836	7032336.858	7134080.616	5230077.735	4555432.465	5843629.425
4-carboxy-2-(tyrosylamino)butanoate	1984487.777	3504100.874	2606496.468	3224331.283	449325.0528	2345556.11	84177.82407	479457.0526	342817.1198
Gentisic acid	21196909.42	17667398.35	19513292.93	8202035.128	11134893.81	20509861.59	47226387.73	40118901.46	43325685.76
Benzoic acid	3402535.824	3816792.228	3297181.743	1944093.625	5216668.654	3148370.785	2455566.609	1374960.218	1644672.256
N-(5-amino-2-[(2,6-diamino-2,6-dideoxyhexopyranosyl)oxy]-3-{[3-o-(2,6-diamino-2,6-dideoxyhexopyranosyl)-beta-d-ribofuranosyl]oxy}-4-hydroxycyclohex	1574532.659	1415290.514	1945439.516	1737357.041	2228830.552	1817779.748	1084176.428	823259.7584	1161930.438
D-tryptophyl-d-alanyl-d-allothreonylglycyl-d-histidyl-l-phenylalanyl-d-methioninamide	762713.4871	1217431.715	431932.302	1065566.434	1719216.472	934991.3093	593010.6979	225194.593	336378.6416
Probucol	3396819.284	1120526.912	1483870.506	963543.5046	2107899.82	1318822.029	891690.4732	768131.4714	560529.5396
ESI+									
Fructoselysine	4255500.181	6106582.497	5618502.197	5008138.885	1018657.456	5239994.764	1933675.365	1997503.329	1186251.428
ε, ε, ε trimethyllysine	5440003.419	4403052.627	5184149.125	4709808.485	4956006.01	7385977.902	13752677.42	18155635.43	14896214.3
(2s)-3-hydroxy-2-({[(3s,4s,5r)-2,3,4-trihydroxy-5-(hydroxymethyl)tetrahydro-2-furanyl]methyl}amino)butanoic acid (non-preferred name)	5979392.207	10129633.41	5430356.833	6384940.844	659709.8548	4617324.346	999947.7639	911290.2368	1088880.957
Otonecine	2618597.291	1793056.742	3366033.199	2467271.506	7392865.508	2320810.654	1841275.961	1301317.533	1285476.411
Tranexamic acid	27652118.13	3697767.015	23426683.99	4842038.751	20693778.56	9136311.956	45507752.49	438605043	463531152.5
Triethylamine	1967886.654	2816113.776	2625657.006	2559772.58	2838685.228	2128847.102	3144279.427	4308770.865	3727812.602
Carmustine	3926681.722	3379990.522	2349395.935	2487080.699	1980981.528	2809266.531	1238843.041	918840.8463	792258.3763
Epiguanine	2016445.048	1637562.566	1922762.374	1793178.414	3579923.201	2080300.835	3292153.278	4100350.243	3994078.984
4-hydroxybenzaldehyde	107152165.5	88670134.4	39547583.7	75531985.13	85704896.39	56825805.35	51178098.54	41262908.12	36205018.45
4-pyridoxate	6152964.813	4543273.085	4066628.903	4557318.536	8555148.801	4598475.943	7367103.709	8985055.958	6949560.185
Bayer e 39	12839429.95	17336780.76	18109073.43	24615954.01	7231820.543	29053316.77	1885020.86	4801827.366	1334150.325
Etilevodopa	3756427.855	2135363.845	4177700.747	4379863.848	17700154.12	6591633.97	538048.3445	1325207.841	244229.0856
Leu-val	2077819.885	1469698.692	1591668.068	1741411.222	1308888.922	1936759.318	2845818.572	2640185.944	2723360.463
Melatonin	1712717.062	150416.4588	104471.8531	365536.6559	499800.9301	1016024.457	70307.38448	136739.9184	55274.50048
1,4-naphthoquinone	1139780.035	623891.5568	1342891.277	1443114.692	599342.5821	1185829.206	620887.8942	350074.6796	248662.3955
Arg-asp	732561.2321	2194231.47	694224.5964	2392270.853	2245222.092	1423455.819	715708.5247	302466.9233	414337.3447
Istamycin a1	2738137.25	2333917.79	2917387.531	2221217.173	2947503.713	2380587.001	1711889.444	1583123.479	2049236.033
Deferoxamine	2188157.937	7165998.778	2654775.009	4600058.788	3111484.389	7408414.97	2470981.419	1630413.048	1654672.188
Codonocarpine	2255579.485	1374362.025	1905991.63	1917338.07	3188369.736	1654283.177	1241297.037	1113934.449	1494202.89
Sophoranone	3678984.439	3872459.319	3386965.343	5728805.677	5286275.077	3754272.733	2758414.21	1369322.18	1373634.711
Anecortave	1219468.539	537397.403	1552054.861	1187455.715	460159.2392	588356.2165	344063.8037	218459.0205	304893.432
Compactin	2019068.31	5226257.591	2444575.602	4075124.451	2723473.712	6163846.951	2025888.457	1446922.991	1740803.222
Quinoline	17832425.25	9184575.158	18359720.03	11259536.38	4944743.491	8288342.579	19106730.71	22405622.82	40507036.13
Indole-3-methyl acetate	23334068.68	10518467.38	23722946.38	15061297.18	4895842.547	10616925.59	22956788.86	28242109.16	52017793.87
12-deoxyphorbol 20-acetate 13-(2-methylbutanoate	1939849.547	726252.5691	2335419.501	1982852.426	659214.5508	747963.1555	521770.6368	319866.3033	608864.3087
Khellin	1018508.744	1086011.469	1040715.457	934677.4504	1539830.989	972397.8553	1504053.793	2030843.862	1936332.665
Epelsiban	2093969.178	1076603.799	2779255.916	2263162.027	971320.6884	978969.6155	570331.4128	429921.0546	689448.8317

4-vinylcyclohexen	2852914.948	3469296.575	3434823.622	2674231.19	2152737.78	3269608.494	3963404.543	4005624.75	3406070.001
Nevirapine	208040.8019	125942.6174	157125.7953	161342.3918	562122.2172	155769.8045	463663.4546	2353646.477	1599630.282
(1r,5r)-3,3,5-trimethylcyclohexyl 5-oxo-l-	8056561.653	4861020.496	5577600.626	6362023.17	1455726.165	8125733.913	1755246.404	459818.2137	319843.3867
Theaspirane	1055684.361	3161593.961	3381546.348	2664974.377	788073.2678	2937124.306	3324312.313	8055446.483	8229270.327
2475675 2,2-Dimethyl-N-(5-{[2-(1-pyrrolidinyl)ethyl]sulfanyl}-1,3,4-thiadiazol-2-yl)propanamide	4115110.475	4673931.096	3020300.857	1622981.886	2554920.664	1421723.659	6655082.381	22880888.61	21741794.07
Drostanolone propionate	4695034.431	5387153.39	3633744.575	1847814.186	2911070.35	1660442.853	7792134.634	26719348.5	25439420.32
N-linoleoyl-4-aminobutyric	1007754.49	310484.0352	124072.7215	335695.9368	1464294.606	391356.9394	837029.6937	3493456.898	2120267.443
Roxane	1802899.667	835640.3708	1139124.695	1168272.358	1812875.907	1549070.913	1798126.155	2124500.242	1922482.252
